# The global epidemiology of hepatitis E virus infection: A systematic review and meta‐analysis

**DOI:** 10.1111/liv.14468

**Published:** 2020-04-24

**Authors:** Pengfei Li, Jiaye Liu, Yang Li, Junhong Su, Zhongren Ma, Wichor M. Bramer, Wanlu Cao, Robert A. de Man, Maikel P. Peppelenbosch, Qiuwei Pan

**Affiliations:** ^1^ Department of Gastroenterology and Hepatology Erasmus MC‐University Medical Center Rotterdam The Netherlands; ^2^ Biomedical Research Center Northwest Minzu University Lanzhou China; ^3^ Medical Library Erasmus MC‐University Medical Center Rotterdam The Netherlands

**Keywords:** epidemiology, hepatitis E virus, prevalence, risk factors, seroprevalence

## Abstract

**Background and aims:**

Hepatitis E virus (HEV), as an emerging zoonotic pathogen, is a leading cause of acute viral hepatitis worldwide, with a high risk of developing chronic infection in immunocompromised patients. However, the global epidemiology of HEV infection has not been comprehensively assessed. This study aims to map the global prevalence and identify the risk factors of HEV infection by performing a systematic review and meta‐analysis.

**Methods:**

A systematic searching of articles published in Medline, Embase, Web of science, Cochrane and Google scholar databases till July 2019 was conducted to identify studies with HEV prevalence data. Pooled prevalence among different countries and continents was estimated. HEV IgG seroprevalence of subgroups was compared and risk factors for HEV infection were evaluated using odd ratios (OR).

**Results:**

We identified 419 related studies which comprised of 1 519 872 individuals. A total of 1 099 717 participants pooled from 287 studies of general population estimated a global anti‐HEV IgG seroprevalence of 12.47% (95% CI 10.42‐14.67; *I* ^2^ = 100%). Notably, the use of ELISA kits from different manufacturers has a substantial impact on the global estimation of anti‐HEV IgG seroprevalence. The pooled estimate of anti‐HEV IgM seroprevalence based on 98 studies is 1.47% (95% CI 1.14‐1.85; *I* ^2^ = 99%). The overall estimate of HEV viral RNA‐positive rate in general population is 0.20% (95% CI 0.15‐0.25; *I* ^2^ = 98%). Consumption of raw meat (*P* = .0001), exposure to soil (*P* < .0001), blood transfusion (*P* = .0138), travelling to endemic areas (*P* = .0244), contacting with dogs (*P* = .0416), living in rural areas (*P* = .0349) and receiving education less than elementary school (*P* < .0001) were identified as risk factors for anti‐HEV IgG positivity.

**Conclusions:**

Globally, approximately 939 million corresponding to 1 in 8 individuals have ever experienced HEV infection. 15‐110 million individuals have recent or ongoing HEV infection. Our study highlights the substantial burden of HEV infection and calls for increasing routine screening and preventive measures.

**Abbreviations**
95% CI: 95% confidential interval
GT: genotype
HEV: hepatitis E virus
HIV: human immunodeficiency virus
IDU: intravascular drug use
MSM: man having sex with man
OR: odd ratio

**Key points**

- This meta‐analysis reports the latest estimation that approximately 939 million of the global population have ever experienced hepatitis E virus (HEV) infection, and 15‐110 million individuals have recent or ongoing infection.
- These findings indicate that HEV infection has emerged as a global health burden requiring implementation of specific control measures.

## 1 INTRODUCTION

Hepatitis E virus (HEV) as a positive‐sense single‐stranded RNA virus is a leading cause of acute viral hepatitis worldwide. The infection is usually asymptomatic or self‐limiting in the general population. However, acute infection in pregnant women may cause severe clinical outcomes, including fulminant hepatic failure with high mortality rate reaching up to 20%‐30%.^1^ These patients are mostly from resource‐limited regions. In European countries, HEV infection has been frequently reported to bear high risk of developing into chronic hepatitis in immunocompromised individuals, in particular organ transplant patients.^2^, ^3^ Thus, HEV is truly imposing a global health burden in both developing and developed countries.

Currently, eight distinct genotypes (GTs) of HEV have been classified.^4^ GT 1‐4 are known to be the main threat to humans. GT 1 and GT 2 are restricted to human and mainly transmit through contaminated water causing acute hepatitis. GT 3 and GT 4 are zoonotic and have been identified in a wide spectrum of hosts, including human, swine, wild boar, goat, cattle, deer, camel and yak.^5^ Both GT 3 and GT 4 can cause chronic infection in organ transplant patients,^2^, ^6^ and consumption of raw or undercooked animal meat has been recognized as the main routes of causing sporadic cases in developed countries.^7^ In fact, the host range of HEV is ever expanding and the implications of the rare GTs and the newly discovered strains in human health remain largely uncertain.^7^ This further complicates the transmission and the risk of HEV infection. In addition to the classical waterborne and foodborne transmission routes, blood transfusion‐mediated transmission has been reported in organ transplant patients.^8^ Person‐to‐person transmission has also been proposed.^9^ Intriguingly, recent evidence has indicated that pet animals including dogs, cats, rabbits and horses might be accidental hosts for HEV and constitute a potential source for transmitting to human.^10^, ^11^ Thus, there is an urgent need to comprehensively understand the risks for HEV infection, in order to device preventive measures.

Globally, it has been roughly estimated that one‐third of the population are living in HEV endemic areas.^12^ More recently, substantial efforts have been dedicated to systematically evaluate HEV prevalence in different continents (eg the Americas and Europe),^13^, ^14^ different countries (eg industrialized countries, China, Iran, Brazil and Somalia)^15^, ^16^, ^17^ and special populations or settings (eg blood donors, swine workers and outbreak setting).^18^, ^19^, ^20^ Most of these studies are based on seropositivity of anti‐HEV IgG antibody. Anti‐HEV IgG antibody developed post‐infection usually persists for many years, and is thus regarded as a marker for past infection.^21^, ^22^ In contrast, anti‐HEV IgM antibody is short‐lived up to a few months, thus considered as evidence of recent or current infection. Detection of HEV RNA is a bona fide marker for active ongoing infection. In this study, we aimed to systematically estimate the global burden of HEV infection. More specifically, we have mapped the global prevalence of past, recent and ongoing HEV infection and evaluated the key risk factors of infection.

## 2 MATERIALS AND METHODS

### 2.1 Data sources and searches

A systematic search was conducted in Medline, Embase, Web of science, Cochrane CENTRAL and Google scholar. Databases were searched for articles in the English language from inception until July 2019. All searches from database were performed by a biomedical information specialist of the medical library, with an exhaustive set of search terms related to hepatitis E virus infection and epidemiology (the full search strategies are provided in the Supporting Information S1). This study is reported in accordance with the Preferred Reporting Items for Systematic Reviews and Meta‐Analysis.^23^ No institutional review board approval was required for this meta‐analysis because our study only included data which had been published previously.

### 2.2 Study selection

Studies were included if they met the following criteria: (a) Studies which contained data about seroprevalence of anti‐HEV IgG, anti‐HEV IgM or HEV RNA positivity, (b) Studies contained mixed population were excluded unless they clearly and explicitly reported the prevalence for each group, (c) Studies contained information of risk factors related to HEV infection and (d) Studies which focused on the HEV prevalence in human beings.

Studies were excluded if they met the following criteria: (a) Studies are systematic review, meta‐analysis, case reports, perspectives and abstracts, (b) None human studies, (c) No primary data or incomplete data, (d) Duplicate data, (e) Studies with <50 individuals were excluded in order to decrease bias caused by small population and (f) Studies concerning about HEV outbreaks, since the prevalence and outcome of HEV infection in these studies would dramatically differ from those of the general population.

Two reviewers (PL and JL) worked independently to determine whether a study met inclusion criteria, abstracted information to assess the methodological validity of each candidate study and extracted data with structured data collection forms. The reviewers resolved discrepancies by jointly reviewing the study in question. If no consensus was reached, a third reviewer (QP), unaware of prior determinations, functioned as an arbiter.

### 2.3 Data extraction and quality assessment

Eligible studies were further divided into three study populations: general population, occupational population and special population. General population included people without apparent risk factors and could be comprised of blood donors, pregnant women, healthy individuals and hospital attendants. For general population, individuals were further divided into subgroups by gender, different age ranges, study period (1993‐2006 or 2007‐2019), country development classification (developing and developed countries), gross national income classification of each country (high, upper middle, lower middle and low income) and ELISA kit manufacturers. More importantly, OR analysis of anti‐HEV IgG seropositivity was conducted in possible risk factors including living area (urban or rural), consumption of raw meat, exposure to soil, contacting with cat or dog, education level (elementary school or above elementary school), intravascular drug use (IDU), water source (tap, well or river), man having sex with man (MSM), transfusion history and travelling history to endemic areas. Occupational population represents people who had been in frequent contact with pigs or pig products, including veterinarians, swine workers, slaughterhouse workers and pork sellers. Special populations are further categorized into four groups as followings: patients with acute hepatitis (caused by hepatitis B virus, hepatitis C virus or other unknown hepatitis), individuals with human immunodeficiency virus (HIV) infection, people who underwent haemodialysis and organ transplant recipients. Two independent reviewers (PL and JY) extracted data, with discrepancies and disagreements resolved by discussion. We extracted data on first author, country, continent, publication date, anti‐HEV IgG prevalence, anti‐HEV IgM prevalence, HEV RNA positivity, subgroup information of anti‐HEV IgG prevalence and HEV‐related risk factors using data extracting forms. When multiple publications were identified that reported on the same populations and outcomes, only the most representative and comprehensive study was included for further meta‐analysis in order to avoid duplicate data. The quality of studies was assessed using the Joanna Briggs Institute checklist for prevalence studies, which enabled assessment of included studies in relation to risk of bias, rigour and transparency.^24^ Studies scoring 1‐3 were defined as low quality, 4‐6 as average quality and 7‐9 as high quality (Table S1). Studies were not excluded on the basis of their quality score to increase transparency and to ensure all available evidence in this area was reported.

### 2.4 Statistics analysis

After checking for consistency, the Metaprop module in the R‐3.5.3 statistical software package was used for meta‐analysis. A 95% confidence interval (95% CI) was estimated using Wilson score method, and pooled seroprevalence was calculated with the DerSimonian‐Laird random effects model with Freeman‐Tukey double arcsine transformation. Heterogeneity across the included studies was assessed using the Cochran *Q* statistics and *I* ^2^ statistics, with *I* ^2^ statistics 25%‐50%, 50%‐75% and >75% considered as mild, moderate and severe heterogeneity respectively. When heterogeneity was higher than 50%, a random effect model will be used. ORs were used to report the risk factors for HEV infection. ORs and their 95% CI were extracted directly from studies when available, with adjusted ORs extracted preferentially over unadjusted ORs. If included studies did not report ORs, crude ORs were calculated from extracted data. We then pooled the ORs using the DerSimonian and Laird random effect models, with the heterogeneity estimated from the Mantel‐Haenszel model. Funnel plots and Egger regression test were used to assess potential publication biases. Additionally, we performed sensitivity analyses using “metainf” in a random model to investigate the effects of population source and potentially unrepresentative samples. The estimated prevalence of anti‐HEV IgG, IgM and HEV RNA infection was based on the global population of 7 530 000 000 on 20 July 2019 (https://population.io).

## 3 RESULTS

### 3.1 Global prevalence of HEV infection

**FIGURE 1:**
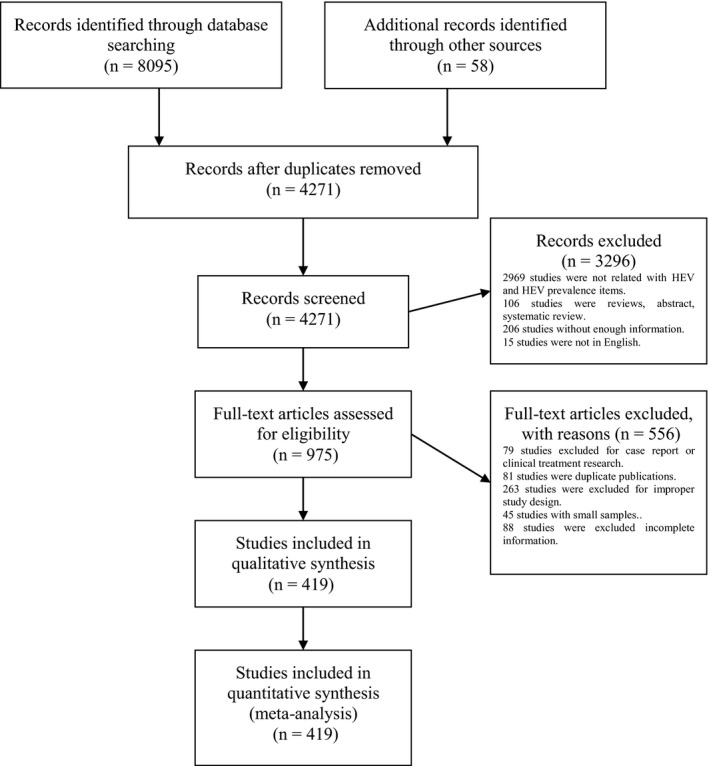
Study selection

**FIGURE 2:**
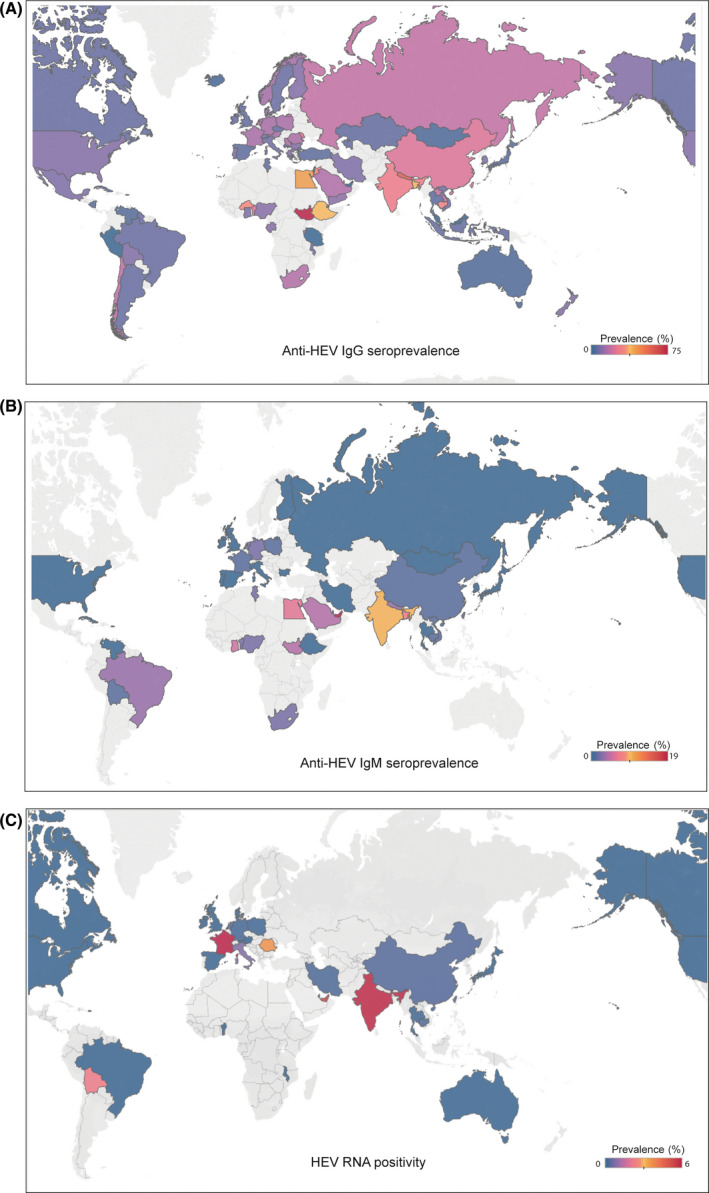
(A) The global seroprevalence of anti‐HEV IgG antibody (B) The global seroprevalence of anti‐HEV IgM antibody (C) The global prevelence of HEV RNA prositivity

**FIGURE 3:**
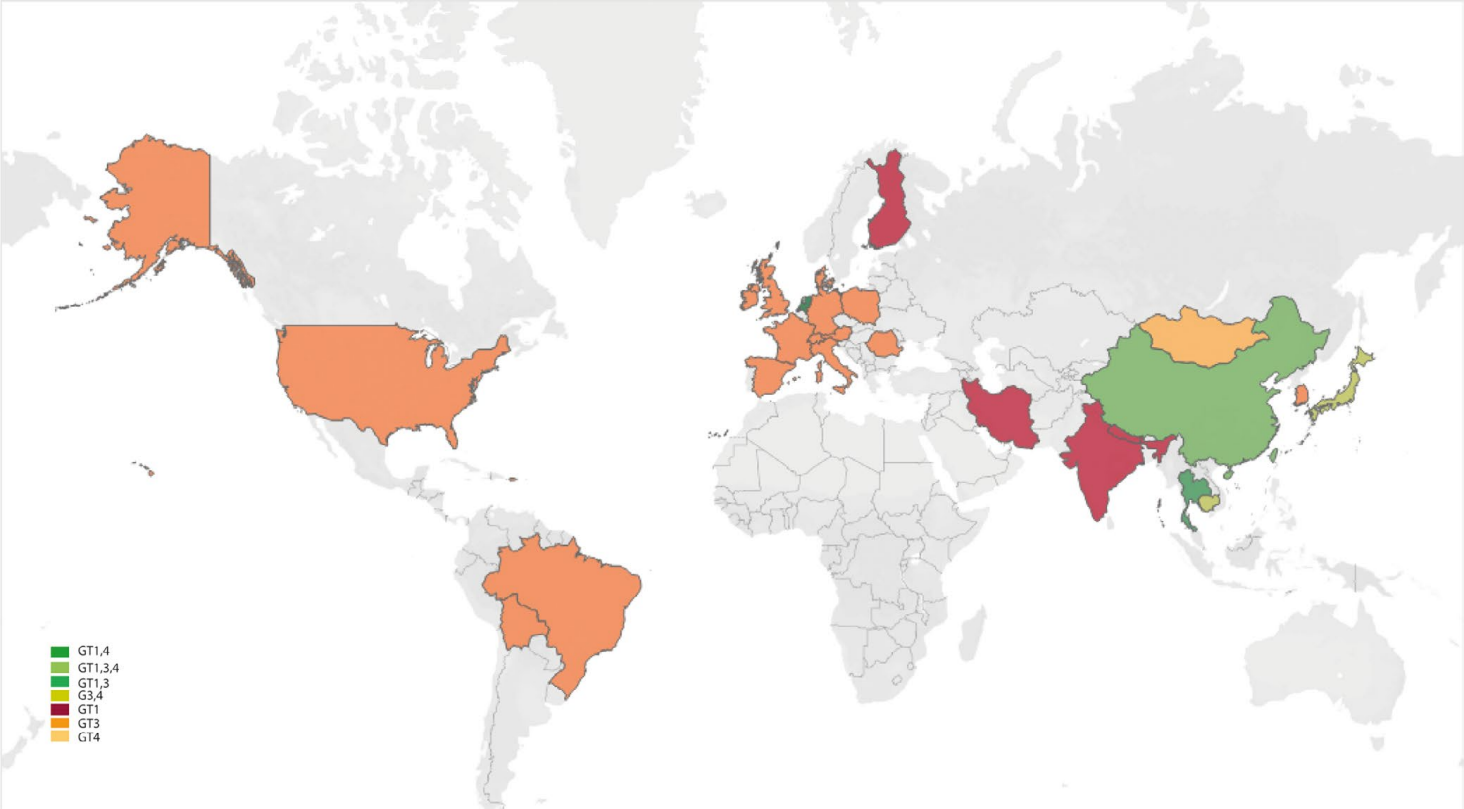
Hepatitis E virus genotype distribution in our study

Our search retuned 8153 records, of which 419 met the inclusion criteria (Figure 1). In total, participants from 302 studies related to general population, and 287 studies were pooled to estimate a global anti‐HEV IgG seroprevalence of 12.47% (1 099 717 individuals included; 95% CI 10.42‐14.67; *I* ^2^ = 100%; Figure 2A; Figure S1). The pooled estimate of anti‐HEV IgM seroprevalence based on 98 studies is 1.47% (479 001 individuals; 95% CI 1.14‐1.85; *I* ^2^ = 99%; Figure 2B; Figure S2). The overall estimate of HEV RNA‐positive rate in the general population is 0.20% (3 444 752; 95% CI 0.15‐0.25; *I* ^2^ = 98%; Figure 2C; Figure S3). We also stratified data to estimate the HEV prevalence in 75 countries among six continents (excluding Antarctica). The highest anti‐HEV IgG seropositivity rate was found in Africa (22 377; 21.76%, 95% CI 13.05‐31.98; *I* ^2^ = 100%), followed by Asia (681 373; 15.80%, 95% CI 13.29‐18.49; *I* ^2^ = 100%), Europe (132 419; 9.31%, 95% CI 7.35‐11.48; *I* ^2^ = 99%), North America (71 989; 8.05%, 95% CI 5.47‐11.09; *I* ^2^ = 99%), South America (14 586; 7.28%, 95% CI 4.83‐10.19; *I* ^2^ = 97%) and Oceania (1563; 5.99%, 95% CI 1.22‐14.03; *I* ^2^ = 96%; Figure S4). Besides, the anti‐HEV IgM seroprevalence was 3.09% (5001; 95% CI 1.49‐5.24; *I* ^2^ = 93%), 1.86% (141 565; 95% CI 1.34‐2.46; *I* ^2^ = 98%), 0.79% (146 322; 95% CI 0.30‐1.51; *I* ^2^ = 99%), 0.22% (12 197; 95% CI 0.00‐0.74; *I* ^2^ = 91%) and 2.43% (2680; 95% CI 0.43‐6.00; *I* ^2^ = 96%) for Africa, Asia, Europe, North America and South America respectively (Figure S5). In addition, the HEV RNA positivity rate was 0.00% (278; 95% CI 0.00‐0.35), 0.93% (727 744; 95% CI 0.48‐1.52; *I* ^2^ = 99%), 0.08% (2 441 774; 95% CI 0.05‐0.11; *I* ^2^ = 95%), 0.00% (34 761; 95% CI 0.00‐0.02; *I* ^2^ = 45%), 0.00% (74 131; 95% CI 0.00‐0.01) and 0.18% (1054; 95% CI 0.00‐1.36; *I* ^2^ = 81%) for Africa, Asia, Europe, North America, Oceania and South America respectively (Figure S6). HEV prevalence varies substantially among countries, from 0.25% (Tanzania, 95% CI 0.00‐0.97) to 74.76% (South Sudan, 95% CI 68.61‐80.44) of anti‐HEV IgG, from 0.00% (Mongolia, 95% CI 0.00‐0.08; Bulgaria, 95% CI 0.00‐0.13) to 19.83% (United Arab, 95% CI 16.35‐23.56) of anti‐HEV IgM and from 0.00% (Benin, Malawi, Australia, Canada, Brazil) to 6.75% (France, 95% CI 0.14‐22.04) of HEV RNA positivity (Table 1; Figures S1‐S3). We also collected data of HEV GTs, with the finding that HEV GT 1 infection occasionally occurred in China and frequently in India, and GT 3 was widely distributed in European countries. GT 3 was also prevalent in Japan and Korea, whereas GT 4 infection mainly emerged in China (Figure 3; Table S2). Based on our comprehensive estimates, approximately 938 991 000 individuals corresponding to 1/8 of the global population have ever experienced HEV infection based on anti‐HEV IgG positivity. Importantly, we estimated approximately 110 691 000 global individuals with current or recent HEV infection and 15 060 000 individuals with ongoing HEV infection based on anti‐HEV IgM or viral RNA positivity respectively.

**TABLE 1:** Hepatitis E virus (HEV) prevalence in general population

Continent	Country	Anti‐HEV IgG							Anti‐HEV IgM Anti‐HEV IgM					HEV RNA				
		No. of studies	Events	Tested (n)	Prevalence (%)		95% CI		No. of studies	Events	Tested (n)	Prevalence (%)	95% CI	No. of studies	Events	Tested (n)	Prevalence (%)	95% CI
Asia	China	42	200 221	579 696		22.68		19.67; 25.83	18	1186	88 587	1.76	1.29; 2.31	5	106	56 319	0.41	0.07;1.06
	Thailand	4	121	2632		4.82		0.30; 14.31	2	5	2057	0.12	0.00; 1.05	1	26	30 115	0.09	0.06;0.12
	Israel	3	133	7115		3.36		0.19; 10.22	··	··	··	··	··	··	··	··	··	··
	Saudi Arabia	3	428	2911		15.41		10.77; 20.70	1	39	900	4.33	3.10; 5.76	··	··	··	··	··
	Japan	8	1901	40 936		4.26		3.27; 5.37	5	32	27 478	0.30	0.06; 0.73	2	41	623 325	0.05	0.00;0.29
	Jordan	1	139	450		30.89		26.71; 35.23	··	··	··	··	··	··	··	··	··	··
	Kazakhstan	1	11	199		5.53		2.79; 9.12	··	··	··	··	··	··	··	··	··	··
	Korea	3	328	3969		9.30		4.87; 14.96	1	9	1030	0.87	0.40; 1.53	··	··	··	··	··
	Vietnam	2	44	833		7.25		0.39; 21.55	1	1	187	0.53	0.00; 2.08	··	··	··	··	··
	Yemen	1	38	356		10.67		7.68; 14.09	··	··	··	··	··	··	··	··	··	··
	Bangladesh	3	509	1707		36.87		17.16; 59.20	1	20	273	7.33	4.54; 10.71	··	··	··	··	··
	India	7	3160	14 136		27.15		19.31; 35.78	5	348	5354	10.18	2.08; 23.38	4	510	8462	6.59	0.22;20.71
	Indonesia	2	50	858		5.83		4.36; 7.49	··	··	··	··	··	··	··	··	··	··
	Mongolia	3	36	1486		2.70		0.06; 9.08	2	0	1237	0	0.00; 0.08	··	··	··	··	··
	Iran	20	1208	12 547		8.98		5.74; 12.86	5	38	4148	0.80	0.19; 1.82	2	7	2031	0.24	0.00;2.16
	Cambodia	3	1074	3173		28.93		14.46; 46.04	2	55	2305	1.31	0.01; 4.74	2	3	1169	0.25	0.05;0.63
	Singapore	1	30	219		13.70		9.48; 18.56	··	··	··	··	··	··	··	··	··	··
	Laos	1	38	210		18.10		13.20; 23.58	··	··	··	··	··	··	··	··	··	··
	Nepal	2	1344	2602		59.19		26.14; 88.13	1	54	1686	3.20	2.42; 4.10	··	··	··	··	··
	Qatar	1	1198	5854		20.46		19.44; 21.51	1	34	5854	0.58	0.40; 0.79	1	4	5854	0.07	0.02;0.15
	United Arab Emirates	··	··	··		··		··	1	93	469	19.83	16.35; 23.56	1	28	469	5.97	4.01;8.29
	Total	111	212 011	681 373		15.80		13.29; 18.49	46	1920	141 565	1.86	1.34; 2.46	18	725	727 744	0.93	0.48;1.52
Africa	Nigeria	2	56	588		10.17		5.32; 16.35	2	17	927	2.83	0.00; 17.56	··	··	··	··	··
	Burkina Faso	1	56	178		31.46		24.86; 38.46	··	··	··	··	··	··	··	··	··	··
	Burundi	1	18	129		13.95		8.54; 20.44	··	··	··	··	··	··	··	··	··	··
	Djibouti	1	14	112		12.50		7.05; 19.23	··	··	··	··	··	··	··	··	··	··
	Egypt	6	6670	14 052		42.43		20.40; 66.19	1	6	100	6.00	2.22; 11.47	··	··	··	··	··
	Ethiopia	2	481	1232		37.09		26.85; 47.95	2	10	1232	0.80	0.38; 1.37	··	··	··	··	··
	Gabon	2	135	1083		10.23		4.02; 18.87	··	··	··	··	··	··	··	··	··	··
	Ghana	3	75	789		8.74		4.39; 14.38	3	44	789	5.87	0.06; 20.10	··	··	··	··	··
	Benin	1	62	278		22.30		17.61; 27.38	1	7	278	2.52	1.01; 4.68	1	0	278	0	0.00;1.32
	Malawi	1	80	397		20.15		16.36; 24.23	··	··	··	··	··	··	··	··	··	··
	South Africa	3	418	2243		16.00		1.55; 41.31	1	16	782	2.05	1.17; 3.16	··	··	··	··	··
	South Sudan	1	154	206		74.76		68.61; 80.44	1	9	206	4.37	2.01; 7.58	··	··	··	··	··
	Tanzania	1	1	403		0.25		0.00; 0.97	··	··	··	··	··	··	··	··	··	··
	Tunisia	1	37	687		5.39		3.82; 7.20	1	20	687	2.91	1.79; 4.30	··	··	··	··	··
	Total	26	8257	22 377		21.76		13.05; 31.98	12	129	5001	3.09	1.49; 5.24	1	0	278	0	0.00;0.35
Europe	Finland	1	37	385		9.61		6.87; 12.75	1	1	385	0.26	0.00; 1.02	··	··	··	··	··
	France	8	3978	17 778		14.51		6.80; 24.49	5	230	15 027	1.16	0.30; 2.57	3	32	917	6.75	0.14;22.04
	Germany	11	2146	11 045		14.35		6.69; 24.30	2	60	2183	2.86	0.00; 23.32	2	16	17 144	0.14	0.02;0.37
	Moldova	1	63	255		24.71		19.62; 30.18	··	··	··	··	··	··	··	··	··	··
	Greece	1	25	265		9.43		6.22; 13.24	··	··	··	··	··	··	··	··	··	··
	Turkey	10	225	4656		4.93		2.68; 7.81	··	··	··	··	··	··	··	··	··	··
	UK	7	706	9672		5.60		3.11; 8.76	3	25	96 341	0.36	0.00; 1.66	4	600	2 201 609	0.02	0.01;0.04
	Iceland	1	6	291		2.06		0.75; 4.01	··	··	··	··	··	··	··	··	··	··
	Ireland	2	73	1274		6.17		3.92; 8.90	1	2	1076	0.19	0.02; 0.53	1	10	24 985	0.04	0.02;0.07
	Italy	14	1690	19 488		7.28		4.54; 10.60	4	49	10 559	0.44	0.19; 0.78	2	12	10 363	0.83	0.00;7.27
	Czech	1	13	230		5.65		3.05; 9.00	··	··	··	··	··	··	··	··	··	··
	Denmark	2	296	1459		11.09		0.00; 57.44	··	··	··	··	··	1	11	25 637	0.04	0.02;0.07
	Austria	2	306	2200		13.91		12.49; 15.38	··	··	··	··	··	1	7	58 915	0.01	0.00;0.02
	Bulgaria	1	67	741		9.04		7.08; 11.21	1	0	741	0	0.00; 0.13	··	··	··	··	··
	Montenegro	1	24	400		6.00		3.89; 8.53	00B7·	··	··	··	··	··	··	··	··	··
	Netherlands	8	3521	25 786		16.07		6.09; 29.62	3	491	13 503	2.00	0.05; 6.63	4	96	67 041	0.40	0.03;1.18
	Norway	1	177	1263		14.01		12.16; 15.98	··	··	··	··	··	··	··	··	··	··
	Poland	4	1561	4497		14.17		2.07; 34.48	3	41	3470	1.02	0.52; 1.67	1	10	12 664	0.08	0.04;0.14
	Portugal	3	380	2812		8.72		2.20; 18.99	1	8	1656	0.48	0.21; 0.87	··	··	··	··	··
	Romania	1	22	148		14.86		9.61; 21.03	··	··	··	··	··	1	6	148	4.05	1.49;7.81
	Russia	1	62	341		18.18		14.27; 22.45	1	2	341	0.59	0.06; 1.67	··	··	··	··	··
	San Marino	1	33	2233		1.48		1.02; 2.02	··	··	··	··	··	··	··	··	··	··
	Serbia	2	50	726		8.46		0.95; 22.35	··	··	··	··	··	··	··	··	··	··
	Spain	7	2282	18 534		5.08		0.85; 12.58	1	7	1040	0.67	0.27; 2.16	3	7	22 351	0.03	0.01;0.06
	Sweden	2	14	205		6.51		2.40; 12.45	··	··	··	··	··	··	··	··	··	··
	Switzerland	4	803	5228		7.25		0.97; 18.62	··	··	··	··	··	··	··	··	··	··
	Total	97	18 560	132 419		9.31		7.35; 11.48	26	916	146 322	0.79	0.30; 1.51	23	807	2 441 744	0.08	0.05;0.11
Oceania	New Zealand	1	98	1013		9.67		7.93; 11.57	··	··	··	··	··	··	··	··	··	··
	Australia	1	17	550		3.09		1.81; 4.70	··	··	··	··	··	1	1	74 131	0	0.00;0.01
	Total	2	115	1563		5.99		1.22; 14.03	··	··	··	··	··	1	1	74 131	0	0.00;0.01
North America	Cuba	3	74	1827		4.89		0.70; 12.55	1	5	1149	0.44	0.14; 0.90	··	··	··	··	··
	Mexico	6	535	4977		8.97		3.75; 16.14	··	··	··	··	··	··	··	··	··	··
	USA	12	7747	60 291		9.65		5.68; 14.53	3	12	11 048	0.16	0.00; 0.78	2	2	20 768	0.01	0.00;0.03
	Canada	2	252	4495		4.35		1.82; 7.91	··	··	··	··	··	1	0	13 993	0	0.00;0.01
	Nicaragua	1	17	399		4.26		2.50;6.46	··	··	··	··	··	··	··	··	··	··
	Total	24	8625	71 989		8.05		5.47; 11.09	4	17	12 197	0.22	0.00; 0.74	3	2	34 761	0	0.00;0.02
South America	Chile	1	82	469		17.48		14.18; 21.05	··	··	··	··	··	··	··	··	··	··
	Argentina	3	145	5831		5.92		1.29; 13.60	··	··	··	··	··	··	··	··	··	··
	Venezuela	1	23	611		3.76		2.40; 5.42	1	3	611	0.49	0.09; 1.20	··	··	··	··	··
	Bolivia	5	209	1940		9.50		3.19; 18.70	1	10	574	1.74	0.83; 2.97	1	3	123	2.44	0.47;5.89
	Brazil	10	315	4739		6.39		3.25; 10.49	4	50	1495	3.46	0.12; 11.09	2	0	931	0	0.00;0.10
	Guyana	1	64	996		6.43		4.99; 8.03	··	··	··	··	··	··	··	··	··	··
	Total	21	838	14 586		7.28		4.83; 10.19	6	63	2680	2.43	0.43; 6.00	3	3	1054	0.18	0.00;1.36
Multiple		6	3158	175 410		14.64		0.40; 43.67	4	18	171 236	0.15	0.00; 0.56	1	16	165 010	0.01	0.01;0.02
Total		287	251 564	1 099 717		12.47		10.42; 14.67	98	3063	479 001	1.47	1.14; 1.85	50	1554	3 444 752	0.20	0.15;0.25

Multiple: studies contained more than one countries.

**FIGURE 4:**
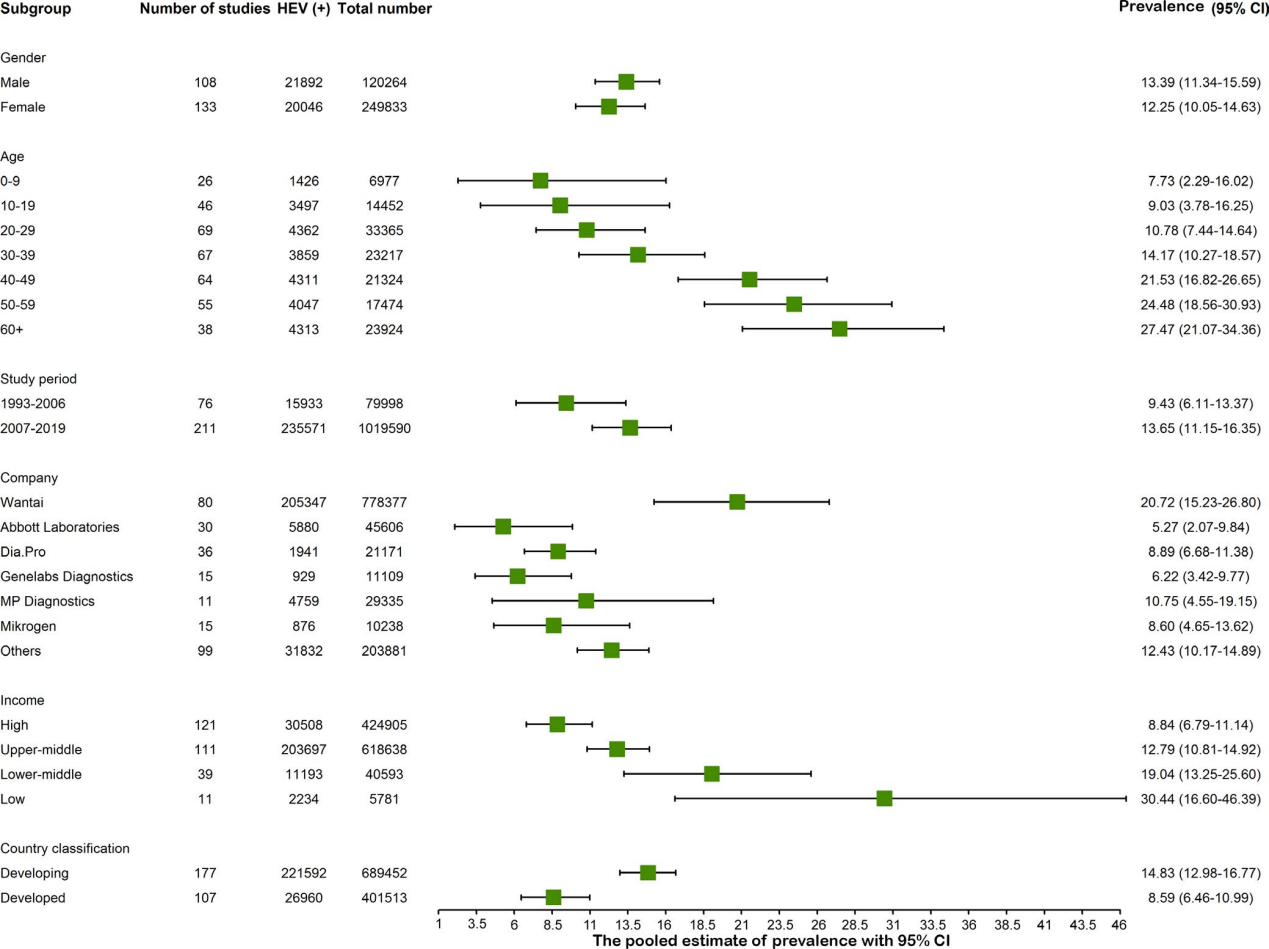
Anti‐hepatitis E virus (HEV) IgG seroprevalence among six subgroups

We next performed subgroup analysis of anti‐HEV IgG positivity rate in general population. General population of six different continents were further divided into seven age groups, including age range of 0‐9, 10‐19, 20‐29, 30‐39, 40‐49, 50‐59 and above 60‐year‐old. The corresponding pooled anti‐HEV IgG‐positive rates are 7.73% (6977 individuals included; 95% CI 2.29‐16.02; *I* ^2^ = 99%), 9.03% (14 452 individuals; 95% CI 3.78‐16.25; *I* ^2^ = 99%), 10.78% (33 365; 95% CI 7.44‐14.64; *I* ^2^ = 99%), 14.17% (23 217; 95% CI 10.27‐18.57; *I* ^2^ = 99%), 21.53% (21 324; 95% CI 16.82‐26.65; *I* ^2^ = 99%), 24.48% (17 474; 95% CI 18.56‐30.93; *I* ^2^ = 99%) and 27.47% (23 924; 95% CI 21.07‐34.36; *I* ^2^ = 99%) respectively (Figure 4; Figures S7 and S8). The positive rate is slightly higher in male (129 569; 13.39%, 95% CI 11.34‐15.59; *I* ^2^ = 99%) compared to female (120 264; 12.25%, 95% CI 10.05‐14.63; *I* ^2^ = 99%; Figure 4; Figure S9). To clarify the HEV prevalence among regions with different levels of economic development, we firstly calculated the anti‐HEV IgG prevalence in high‐income countries, upper middle income countries, lower middle income countries and low income countries. We estimated the anti‐HEV IgG positivity of 8.84% (424 905; 95% CI 6.79‐11.14; *I* ^2^ = 100%) in high‐income countries, 12.79% (618 638; 95% CI 10.81‐14.92; *I* ^2^ = 100%) in upper middle income countries, 19.04% (40 593; 95% CI 13.25%‐25.60%; *I* ^2^ = 100%) in lower middle income countries and 30.44% (5781; 95% CI 16.60‐46.39; *I* ^2^ = 99%) in low income countries (Figure 4; Figure S10). The pooled estimate of anti‐HEV IgG seroprevalence was 14.83% (689 452; 95% CI 12.98‐16.77; *I* ^2^ = 100%) in developing countries compared to 8.59% (401 513; 95% CI 6.46‐10.99; *I* ^2^ = 100%) in developed countries (Figure 4; Figure S11). Of the global HEV prevalence during the period of 1993‐2019, we estimated anti‐HEV IgG‐positive rate of 9.43% (79 998; 95% CI 6.11‐13.37; *I* ^2^ = 100%) during 1993‐2006 and 13.65% (1 019 719; 95% CI 11.15‐16.35; *I* ^2^ = 100%) during 2007‐2019 (Figure 4, Figure S12).

### 3.2 Prevalence of HEV infection in occupational population and special population

Anti‐HEV IgG seroprevalence data from veterinarians, swine workers, slaughters and pork sellers were collected to estimate the overall anti‐HEV seroprevalence in occupational population. Based on 43 studies with 8776 occupational individuals, the overall seropositivity of anti‐HEV IgG is 24.04% (95% CI 18.55‐29.99; *I* ^2^ = 97%; Figure S13). In total, data from 126 studies were extracted to analyse the prevalence in special populations. The overall anti‐HEV IgG, anti‐HEV IgM and viral RNA‐positive rates are 15.43% (95% CI 12.82‐18.24; *I* ^2^ = 98%), 3.21% (95% CI 1.77‐5.06; *I* ^2^ = 98%) and 1.10% (95% CI 0.53‐1.87; *I* ^2^ = 97%) respectively (Figures S14‐S16). Among these special populations, patients with acute hepatitis have the highest positive rate of anti‐HEV IgG (21.49%, 95% CI 12.65‐31.92; *I* ^2^ = 99%), anti‐HEV IgM (8.62%, 95% CI 4.16‐14.51; *I* ^2^ = 99%) and viral RNA (5.57%, 95% CI 2.26‐10.21; *I* ^2^ = 99%; Figures S17‐S19). The anti‐HEV IgG‐positive rates in two special groups are higher than that in general population, with 16.91% (95% CI 12.64‐21.67; *I* ^2^ = 98%) in the HIV population and 13.10% (95% CI 9.34‐17.39; *I* ^2^ = 96%) in haemodialysis population, while it is slightly lower in organ transplant recipients with 11.68% (95% CI 7.91‐16.06; *I* ^2^ = 97%) seropositivity (Figures S20‐S27; Table S5).

### 3.3 Risk factors of HEV

We investigated the potential risk factors for HEV in the general population (Figure S28). Significant rising effects on anti‐HEV IgG seropositivity were observed in consumption of raw meat (OR 1.45, 95% CI 1.20‐1.76, *P* = .0001), exposure to soil (OR 1.52, 95% CI 1.24‐1.86, *P* < .0001), blood transfusion (OR 1.61, 95% CI 1.10‐2.36, *P* = .0138), travelling to endemic areas (OR 1.39, 95% CI 1.04‐1.84, *P* = .0244), contacting with dogs (OR 1.45, 95% CI 1.01‐2.07, *P* = .0416), living in rural areas (OR 0.80, 95% CI 0.65‐0.98, *P* = .0349) and receiving education less than elementary school (OR 1.71, 95% CI 1.41‐2.07, *P* < .0001). No statistically significant differences were identified for anti‐HEV IgG positivity in respect to different water source (*P* = .0909), IDU experience (*P* = .4321), MSM experience (*P* = .5576) and contacting with cats (*P* = .4791; Figure S29‐S39). Sensitivity analysis detected no study having an obvious effect influence to the pooled estimates of HEV prevalence in the general population (Table S3).

### 3.4 Anti‐HEV IgG detection rate of different ELISA kits

We finally analysed the detection rates of the ELISA kits from six manufacturers. The detection rates of anti‐HEV IgG seropositivity vary dramatically, with the highest of Wantai assay (20.72%, 95% CI 2.07‐9.84; *I* ^2^ = 100%) followed by MP Diagnostics (10.75%, 95% CI 4.55‐19.15; *I* ^2^ = 100%), Dia.Pro (8.89%, 95% CI 6.68‐11.38; *I* ^2^ = 97%), Mikrogen (8.60%, 95% CI 4.65‐13.62; *I* ^2^ = 98%), Genelabs Diagnostics (6.22%; 95% CI 3.42‐9.77; *I* ^2^ = 98%) and Abbott Laboratories (5.27%, 95% CI 2.07‐9.84; *I* ^2^ = 100%) (Figure S40; Table S4).

## 4 DISCUSSION

It has been estimated that one‐third of the global population, representing over two billion people, live in HEV endemic areas at risk of infection.^12^ This has been widely misinterpreted as that 2.3 billion of the population have been infected with HEV.^25^ In fact, the true burden of hepatitis E remains largely unknown.^26^ In this study, we have systematically and comprehensively assessed the global HEV prevalence by retrieving data from 75 countries of the six continents. We estimated that 12.47% of the global population, corresponding to approximately 939 million individuals, have experienced past infection of HEV based on their seropositivity of anti‐HEV IgG antibody. Africa and Asia have been previously recognized for the high prevalence of HEV.^27^, ^28^ Our estimates confirm the high seroprevalence rates of 21. 76% and 15.80% in Africa and Asia respectively. For Europe, we estimated a prevalence rate of 9.31%, which is substantially lower than a previous estimation of 16.90% from a meta‐analysis performed in 2016.^14^ A possible explanation for the disparity could be that they collected fewer studies and included small size populations, and thus is prone to cause more bias. In Americas, we observed a slightly higher seroprevalence rate in North (8.05%) compared to South (7.28%) America, which is consistent with the results from a recent meta‐analysis.^13^

Of a technical note, it has been well‐realized that there are substantial differences in sensitivity and specificity of the anti‐HEV IgG ELISA kits from different manufacturers.^29^, ^30^ Our results largely agree with the literature that the Wantai assay has the highest sensitivity and has been most widely used.^31^ Thus, the use of different anti‐HEV IgG ELISA kits may partially explain the disparities in estimates among different studies, and caution should be taken when interpreting the seroprevalence rate in this respect.

The bona fide disease burden of HEV lies in the actively infected patients. The global burden caused by GT 1 and GT 2 HEV in Africa and Asia has been mathematically modelled for 2005. Among the 4.7 billion people in these regions corresponding to 72.8% of the global population in 2005, it has been estimated as 20 million incident HEV infections, 3.4 million symptomatic cases, 70 000 deaths and 3000 stillbirths.^32^ In 2011, WHO reported 14 million symptomatic cases annually worldwide with 300 000 deaths and 5200 stillbirths.^33^ Hypothetically, if both estimates are accurate, there would be about 10 million symptomatic cases annually from developed countries, which are mainly caused by the zoonotic GT 3 strains. This clearly disagrees with the vast majority of the current literature that we do not expect the burden in respect to symptomatic infection would be three times in developed compared to developing countries. In this study, we have estimated approximately 110 million individuals with recent/current infection based on anti‐HEV IgM antibody positivity and 15 million with ongoing infection based on HEV RNA positivity. As viral RNA persists for a few weeks and anti‐HEV IgM antibody for a few months,^34^ the annually global infections are probably at a range of hundred(s) millions. However, the available data regarding anti‐HEV IgM antibody or viral RNA positivity are very limited. Thus, our estimates may have biases, and we were not able to further sub‐analyse regional prevalence, GT‐specific burden or clinical outcome, which require future studies in these aspects.

Accumulating knowledge on HEV biology and transmission routes has facilitated the identification of risk factors for the infection. A wide range of domestic or wild animals have been recognized as reservoirs for the zoonotic strains. Consumption of uncooked meat or meat product from swine, wild boar or deer has been widely reported to cause GT 3 HEV infection in European countries.^35^, ^36^ As expected, consumption of raw meat is an important risk factor revealed by our meta‐analysis. This is in line with previous reports that humans with occupational exposure to pigs are at a high risk of HEV infection.^37^, ^38^ In this study, we observed twofold higher anti‐HEV seropositivity in occupational population who had frequent contact with pig or pig products compared to the general population.

The host range for HEV is ever expanding and cross‐species infections commonly occur.^7^ Intriguingly, recent evidence has indicated that companion animals including dogs, cats, rabbits and horses might be accidental hosts for HEV and might constitute a source for HEV transmission to human.^10^, ^11^, ^39^ Transmission of rat HEV to human has been recently reported in Hong Kong.^40^ Dogs and cats are the most common household pets. Previous studies have reported that seroprevalence of HEV antibodies in dogs ranges from 0.8% in the UK,^41^ 6.79% in Brazil,^42^ 17.8% to 36.55% in different regions of China,^10^, ^11^ 22.7% in India^43^ and 56.6% in Germany.^44^ Interestingly, when comparing with the general population, veterinarians and dog farm staff who are frequently exposed to dogs have significantly higher anti‐HEV antibody positivity.^10^ The anti‐HEV seroprevalence rates in cats have been reported to be 6.28% in China,^11^ 8.1% in Korea^45^ and 33% in Japan.^46^ In this study, we found that people who frequently contact with dogs have higher anti‐HEV IgG seropositivity. This was not found in people who contact with cats, but the number of studies is very limited. These results call more attention to address the potential role of pets in HEV zoonotic transmission, although currently it remains unconfirmed whether pets are reservoirs, requiring further investigation.

Previous studies have indicated the differences of HEV seroprevalence between rural and urban areas.^47^, ^48^, ^49^ We found that rural compared to urban residents have higher risk of HEV infection. This largely agrees with our findings that high exposure to soil is also a risk factor. In addition, we observed the high risk of HEV infection in individuals with lower education levels, consistent with previous studies.^50^, ^51^ Conceivably, this population are more likely living in rural areas with compromised sanitation conditions and more frequent exposure to animals or soil. Although we did not find differences of HEV prevalence with respect to different water source, this does not contradict to the fact that contaminated water is the main source of GT 1 infection, especially during outbreak. In our study, we have excluded studies related to outbreak, and the number of included studies reporting water source is also very limited, which may cause bias.

Of note, there are some limitations of our study. Firstly, the number of available studies, in particular on anti‐HEV IgM antibody and viral RNA positivity, is limited. We were also not able to further analyse detailed regional prevalence, GT‐specific burden or clinical outcome. Secondly, we have focused on HEV prevalence, but did not estimate the incidence, which is also very relevant for assessing the disease burden. Thirdly, the assays used for HEV detection are heterogeneous in sensitivity and specificity, which may affect the estimates. Fourthly, publication bias existed in our study which was reflected in Funnel and Egger test (Figures S41‐S42).

In summary, we found that 1/8 of the global population, corresponding to over 900 million individuals, have ever encountered HEV infection. Importantly, 15‐110 million individuals are experiencing recent or ongoing infection. Consuming raw meat, exposing to soil, blood transfusion, travelling to endemic areas, contacting with dogs, living in rural areas and receiving lower level of education were identified as risk factors for HEV infection. Thus, our results bear important implications for assessing the global burden and devising preventive measures for controlling HEV infection.

## CONFLICT OF INTEREST

The authors do not have any disclosures to report.

## AUTHOR CONTRIBUTIONS

P. Li and J. Liu performed the study, acquisition and analysis of data. W. M. Bramer performed literature database searching. Y. Li, W. Cao, M. P. Peppelenbosch and Q. Pan discussed the data. J. Su and Z. Ma provided technical assistance. P. Li drafted the manuscript. R. A. de Man, M. P. Peppelenbosch and Q. Pan contributed to the writing of the manuscript. P. Li and J. Liu contributed equally and share co‐first authorship. Q. Pan and J. Liu share co‐correspondence authorship.

## Supporting information

Supplementary Material
