# Supplementary material for: The global epidemiology of hepatitis E virus infection: A systematic review and meta‐analysis
Source: Liver Int. 2020 Apr 24;40(7):1516–28. doi: 10.1111/liv.14468 (PMC7384095; doi:10.1111/liv.14468)
Supplement: Supplementary file 1 — Supplementary Material [file LIV-40-1516-s001.pdf]

## Supplementary information to:

# The global epidemiology of hepatitis E virus infection: a systematic review and meta-analysis

***Supplementary Materials***

**Table of Contents**

Supplementary method 1. Searching strategy for prevalence and burden of hepatitis E virus (HEV) infection in the global population. (page 4-5)

Figure S1. Forest plot of estimated pooled anti-HEV IgG seroprevalence among general population based on different countries. (page 6-11)

Figure S2. Forest plot of estimated pooled anti-HEV IgM seroprevalence among general population based on different countries. (page 12-14)

Figure S3. Forest plot of estimated pooled HEV RNA positive rate among general population based on different countries. (page 15-16)

Figure S4. Forest plot of estimated pooled anti-HEV IgG seroprevalence among general population based on different continents. (page 17-20)

Figure S5. Forest plot of estimated pooled anti-HEV IgM seroprevalence among general population based on different continents. (page 21-22)

Figure S6. Forest plot of estimated pooled HEV RNA positive rate among general population based on different continents. (page 23)

Figure S7. Forest plot of estimated pooled anti-HEV IgG seroprevalence among general population based on different ages. (page 24-28)

Figure S8. Anti-HEV IgG seroprevalence among different age range for general population from different continents. (page 29)

Figure S9. Forest plot of estimated pooled anti-HEV IgG seroprevalence among general population based on different genders. (page 30-33)

Figure S10. Forest plot of estimated pooled anti-HEV IgG seroprevalence among general population based on high income countries, upper-middle income countries, lower middle income countries, and low income countries. (page 34-37)

Figure S11. Forest plot of estimated pooled anti-HEV IgG seroprevalence among general population based on developed countries and developing countries. (page 38-41)

Figure S12. Forest plot of estimated pooled anti-HEV IgG seroprevalence among general population based on time period of 1993-2006 and 2007-2019. (page 42-45)

Figure S13. Forest plot of estimated pooled anti-HEV IgG seroprevalence among swine-related occupational population based on different countries. (page 46-47)

Figure S14. Forest plot of estimated pooled anti-HEV IgG seroprevalence among special population. (page 48-49)

Figure S15. Forest plot of estimated pooled anti-HEV IgM seroprevalence among special population. (page 50)

Figure S16. Forest plot of estimated pooled HEV RNA positive rate among special population. (page 51)

Figure S17. Forest plot of estimated pooled seroprevalence of anti-HEV IgG among hepatitis population. (page 52)

Figure S18. Forest plot of estimated pooled seroprevalence of anti-HEV IgM among hepatitis population. (page 53)

Figure S19. Forest plot of estimated pooled HEV RNA positive rate among hepatitis population. (page 54)

Figure S20. Forest plot of estimated pooled seroprevalence of anti-HEV IgG among HIV population based on different countries. (page 55-57)

Figure S21. Forest plot of estimated pooled seroprevalence of anti-HEV IgM among HIV population based on different countries. (page 58-59)

Figure S22. Forest plot of estimated pooled HEV RNA positive rate among HIV population based on different countries. (page 60)

Figure S23. Forest plot of estimated pooled seroprevalence of anti-HEV IgG among transplant population based on different countries. (page 61)

Figure S24. Forest plot of estimated pooled seroprevalence of anti-HEV IgM among transplant population based on different countries. (page 62)

Figure S25. Forest plot of estimated pooled HEV RNA positive rate among transplant population based on different countries. (page 63)

Figure S26. Forest plot of estimated pooled seroprevalence of anti-HEV IgG among hemodialysis population based on different countries. (page 64)

Figure S27. Forest plot of estimated pooled seroprevalence of anti-HEV IgM among hemodialysis population based on different countries. (page 65)

Figure S28. Pooled Odd Ratios of anti-HEV IgG seroprevalence to investigate the risk factors for HEV infection among general population. (page 66)

Figure S29. Odds ratio analysis of anti-HEV IgG seroprevalence for people contacting dogs or not in general population. (page 67)

Figure S30. Odds ratio analysis of anti-HEV IgG seroprevalence for people contacting cats or not in general population. (page 67)

Figure S31. Odds ratio analysis of anti-HEV IgG seroprevalence for people with or without blood transfusion in general population. (page 67)

Figure S32. Odds ratio analysis of anti-HEV IgG seroprevalence for people with or without consumption of raw meat in general population. (page 68)

Figure S33. Odds ratio analysis of anti-HEV IgG seroprevalence for people frequently exposed to soil or unexposed to soil in general population. (page 68)

Figure S34. Odds ratio analysis of anti-HEV IgG seroprevalence for people with or without travelling to endemic areas in general population. (page 68)

Figure S35. Odds ratio analysis of anti-HEV IgG seroprevalence for urban residents or rural residents in general population. (page 69)

Figure S36. Odds ratio analysis of anti-HEV IgG seroprevalence for people receive water source of tap/well/river in general population. (page 70)

Figure S37. Odds ratio analysis of anti-HEV IgG seroprevalence for people accepting education of elementary or above elementary in general population. (page 71)

Figure S38. Odds ratio analysis of anti-HEV IgG seroprevalence for people with or without MSM experience in general population. (page 72)

Figure S39. Odds ratio analysis of anti-HEV IgG seroprevalence for people with or without IDU experience in general population. (page 72)

Figure S40. Forest plot of estimated pooled anti-HEV IgG positive rate among general population based on different ELISA manufactures. (page 73-76)

Figure S41. Funnel plot for anti-HEV IgG seroprevalence among general population. (page 77)

Figure S42. Egger plot for anti-HEV IgG seroprevalence among general population. (page 77)

Table S1. Score of studies evaluated by JBI Critical Appraisal Tools. (page 78-88)

Table S2. Distribution of HEV genotypes 1-4 in different countries. (page 89-90)

Table S3. Sensitivity analysis in epidemiology of anti-HEV IgG in general population. (page 91-94)

Table S4. Manufactures of ELISA assays used in general population. (page 95-98)

Table S5. HEV prevalence among occupational population and special population. (page 99)

**Supplementary file 1.**

We systematically searched Embase.com, Medline Ovid, Web of science, Cochrane CENTRAL, Google scholar to identify studies providing prevalence rate of HEV.

| | | |
|---|---|---|
| Embase.com | 3196 | 3123 |
| Medline Ovid | 2786 | 592 |
| Web of science | 1885 | 453 |
| Cochrane CENTRAL | 28 | 9 |
| Google scholar | 200 | 36 |
| Total | 8095 | 4213 |

The search terms used in different database were as follows:

Embase.com

('Hepatitis E virus'/de OR 'hepatitis E'/de OR 'hepatitis E antibody'/de OR 'hepatitis E antigen'/de OR ((Hepatitis NEAR/3 E) OR hev):ab,ti) AND ('epidemiological data'/de OR 'epidemiology'/de OR 'geographic distribution'/de OR incidence/exp OR 'patient volume'/de OR prevalence/exp OR 'infection rate'/de OR 'age distribution'/de OR seroepidemiology/de OR 'occupation'/de OR vocation/de OR specialization/de OR 'virus virulence'/de OR virulence/de OR geography/de OR 'occupational disease'/de OR 'occupational exposure'/de OR 'occupational health'/de OR 'occupational hazard'/de OR 'geographic names'/exp OR (epidemiolog* OR seroepidemiolog* OR ((geograph* OR global* OR age) NEAR/3 (distribut*)) OR incidenc* OR (patient* NEAR/3 volume*) OR prevalen* OR seroprevalen* OR (infection* NEAR/3 rate*) OR occupation* OR vocation* OR virulen* OR specialization* OR specialisation*):ab,ti) NOT (hepatitis-b-e NOT hepatitis-e) NOT ([animals]/lim NOT [humans]/lim) NOT ('case report'/de OR 'case report*':ti) NOT ([Conference Abstract]/lim) AND [english]/lim

Medline Ovid

(Hepatitis E virus/ OR Hepatitis E/ OR ((Hepatitis ADJ3 E) OR hev).ab,ti.) AND (Epidemiological Monitoring/ OR Epidemiology/ OR Epidemiology.fs. OR exp Incidence/ OR exp Prevalence/ OR Age Distribution/ OR Occupations/ OR Specialization/ OR Virulence/ OR Geography/ OR Occupational Diseases/ OR Occupational Exposure/ OR Occupational Health/ OR exp Geographic Locations/ OR (epidemiolog* OR seroepidemiolog* OR ((geograph* OR global* OR age) ADJ3 (distribut*)) OR incidenc* OR (patient* ADJ3 volume*) OR prevalen* OR seroprevalen* OR (infection* ADJ3 rate*) OR occupation* OR vocation* OR virulen* OR specialization* OR specialisation*).ab,ti.) NOT (hepatitis-b-e NOT hepatitis-e) NOT (exp animals/ NOT humans/) NOT (case report/ OR case report*.ti.) AND english.la.

Web of science

TS=((((Hepatitis NEAR/2 E) OR hev)) AND ((epidemiolog* OR seroepidemiolog* OR ((geograph* OR global* OR age) NEAR/2 (distribut*)) OR incidenc* OR (patient* NEAR/2 volume*) OR prevalen* OR seroprevalen* OR (infection* NEAR/2 rate*) OR occupation* OR vocation* OR virulen* ORspecialization* OR specialisation*)) NOT (hepatitis-b-e NOT hepatitis-e) NOT ((animal* OR rat OR rats OR mouse OR mice OR murine OR dog OR dogs OR canine OR cat OR cats OR feline OR rabbit OR cow OR cows OR bovine OR rodent* OR sheep OR ovine OR pig OR swine OR porcine OR veterinar* OR chick* OR zebrafish* OR baboon* OR nonhuman* OR primate* OR cattle* OR goose OR geese OR duck OR macaque* OR avian* OR bird* OR fish*) NOT (human* OR patient* OR women OR woman OR men OR man))) AND DT=(article) AND LA=(english) NOT TI=("case report*")

Cochrane CENTRAL

(((Hepatitis NEAR/3 E) OR hev):ab,ti) AND ((epidemiolog* OR seroepidemiolog* OR ((geograph* OR global* OR age) NEAR/3 (distribut*)) OR incidenc* OR (patient* NEAR/3 volume*) OR prevalen* OR seroprevalen* OR (infection* NEAR/3 rate*) OR occupation* OR vocation* OR virulen* OR specialization* OR specialisation*):ab,ti) NOT (hepatitis next b next e NOT hepatitis next e)

Google scholar

"HepatitisE" epidemiology|seroepidemiology|"geographic|global|age distribution"|incidence|"patient volume"|prevalence|seroprevalence|"infection rate"|occupational|virulence

**Study selection criteria**

Study population:

General population: blood donors, pregnant women, healthy people, hospital attendants

Occupational population: veterinarians, swine workers, slaughterhouse workers, pork sellers

Special population: people suffering hemodialysis, people with acute hepatitis (hepatitis caused by hepatitis B virus or hepatitis C virus or unknown pathogen), HIV-infected population, transplant recipients (renal transplant, liver transplant, lung transplant, hear transplant, bone marrow transplant).

Study inclusion criteria: provide data related to prevalence of hepatitis E virus infection.

Study exclusion criteria: case reports, reviews, none-HEV studies, antiviral research of HEV, study of clinical drug effect, no primary data, outbreak, animal HEV studies, replicate data in same areas, study population below 50 for general population, study population below 50 for occupational and special population.

**Figure S1. Forest plot of estimated pooled anti-HEV IgG seroprevalence among general population based on different countries.**

Note: country named multiple means people in the study are from more than one country. For example, Halliday et al, 2014 contained people from Papua New Guinea, Fiji, Kiribati; Baylis yet al, 2012 contained people from Sweden, Germany and USA.

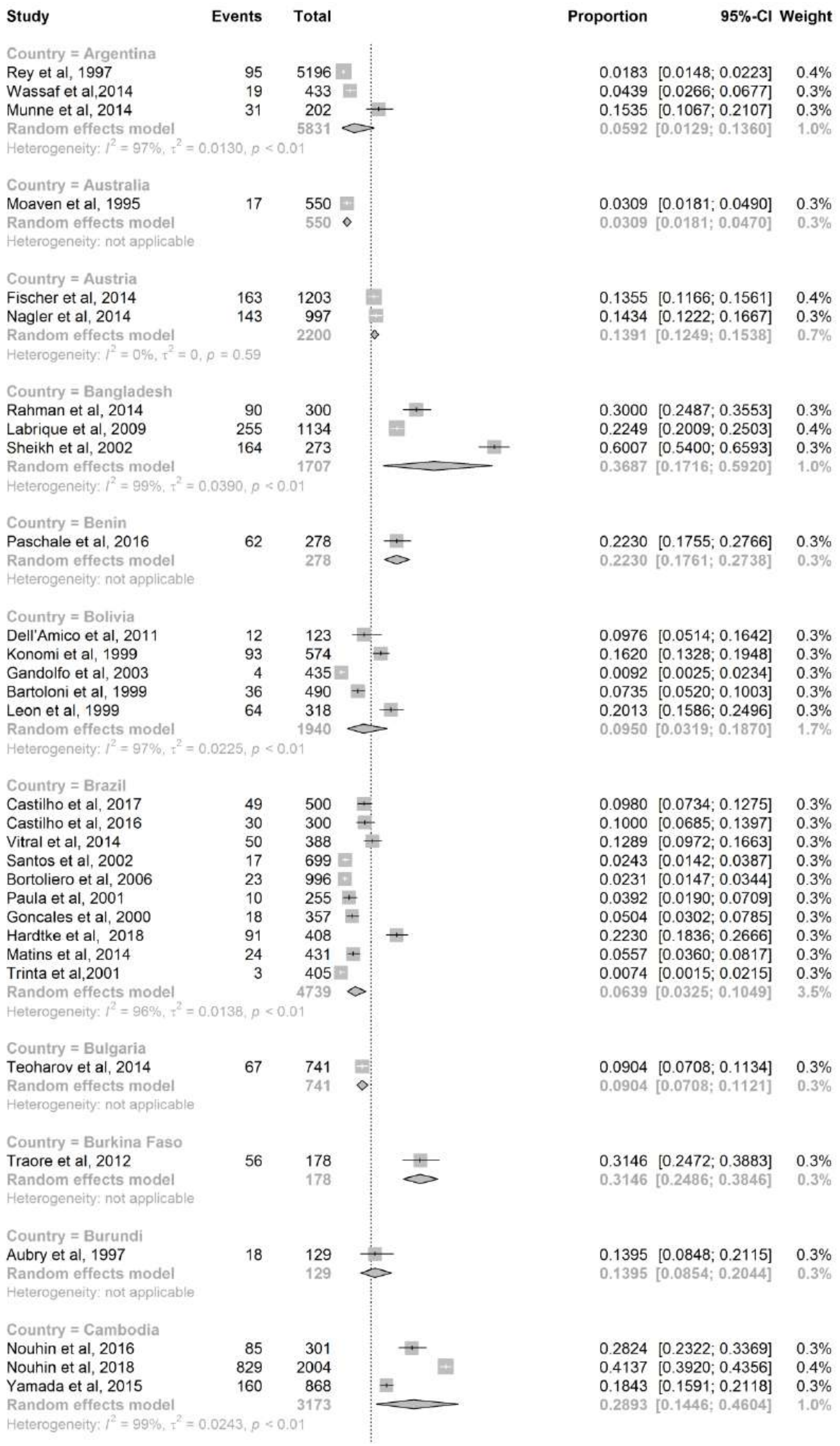

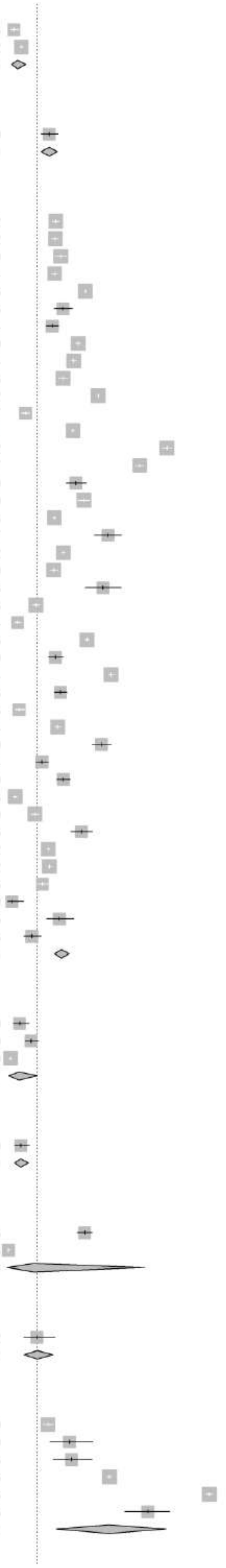

| | | | | | |
|---|---|---|---|---|---|
| Country = Canada | | | | | |
| Minuk et al, 2007 | 11 | 393 | 0.0280 | [0.0141; 0.0495] | 0.3% |
| Fearon et al, 2017 | 241 | 4102 | 0.0588 | [0.0517; 0.0664] | 0.4% |
| Random effects model | | 4495 | 0.0435 | [0.0182; 0.0791] | 0.7% |
| Heterogeneity: $I^2$ = 88%, $\tau^2$ = 0.0026, $p$ < 0.01 | | | | | |
| Country = Chile | | | | | |
| Ibarra et al, 1994 | 82 | 469 | 0.1748 | [0.1415; 0.2123] | 0.3% |
| Random effects model | | 469 | 0.1748 | [0.1418; 0.2105] | 0.3% |
| Heterogeneity: not applicable | | | | | |
| Country = China | | | | | |
| Chang et al, 2009 | 522 | 2572 | 0.2030 | [0.1876; 0.2190] | 0.4% |
| Geng et al, 2011 | 538 | 2682 | 0.2006 | [0.1856; 0.2163] | 0.4% |
| Yuan et al, 2017 | 268 | 1200 | 0.2233 | [0.2001; 0.2480] | 0.4% |
| Chen et al, 2019 | 799 | 4044 | 0.1976 | [0.1854; 0.2102] | 0.4% |
| Guo et al, 2009 | 14610 | 44816 | 0.3260 | [0.3217; 0.3304] | 0.4% |
| Zhuang et al, 2014 | 113 | 486 | 0.2325 | [0.1956; 0.2727] | 0.3% |
| Wong et al, 2004 | 176 | 934 | 0.1884 | [0.1638; 0.2150] | 0.3% |
| Wang et al, 2017 | 2682 | 9069 | 0.2957 | [0.2864; 0.3052] | 0.4% |
| Zhu et al, 2008 | 1289 | 4654 | 0.2770 | [0.2641; 0.2901] | 0.4% |
| Yang et al, 2014 | 473 | 2028 | 0.2332 | [0.2150; 0.2523] | 0.4% |
| Zhu et al, 2014 | 152061 | 400162 | 0.3800 | [0.3785; 0.3815] | 0.4% |
| Peng et al, 1995 | 76 | 997 | 0.0762 | [0.0605; 0.0945] | 0.3% |
| Ren et al, 2013 | 2945 | 10741 | 0.2742 | [0.2658; 0.2827] | 0.4% |
| Feng et al, 2018 | 1273 | 1912 | 0.6658 | [0.6441; 0.6869] | 0.4% |
| Cai et al, 2016 | 1842 | 3337 | 0.5520 | [0.5349; 0.5690] | 0.4% |
| Chiu et al, 2013 | 129 | 450 | 0.2867 | [0.2453; 0.3309] | 0.3% |
| Chan et al, 2017 | 492 | 1539 | 0.3197 | [0.2964; 0.3436] | 0.4% |
| Chen et al, 2012 | 2793 | 14208 | 0.1966 | [0.1901; 0.2032] | 0.4% |
| Liang et al, 2014 | 129 | 307 | 0.4202 | [0.3644; 0.4776] | 0.3% |
| Jia et al, 2014 | 3721 | 15862 | 0.2346 | [0.2280; 0.2413] | 0.4% |
| Ma et al, 2010 | 407 | 2090 | 0.1947 | [0.1780; 0.2124] | 0.4% |
| Li et al, 2011 | 69 | 173 | 0.3988 | [0.3253; 0.4759] | 0.3% |
| Li et al, 2014 | 197 | 1638 | 0.1203 | [0.1049; 0.1370] | 0.4% |
| Lee et al, 1993 | 43 | 984 | 0.0437 | [0.0318; 0.0584] | 0.3% |
| Liu et al, 2019 | 3419 | 10256 | 0.3334 | [0.3242; 0.3426] | 0.4% |
| Kang et al, 2017 | 121 | 600 | 0.2017 | [0.1703; 0.2360] | 0.3% |
| Li et al, 2006 | 3154 | 7284 | 0.4330 | [0.4216; 0.4445] | 0.4% |
| Cui et al, 2016 | 228 | 1028 | 0.2218 | [0.1967; 0.2485] | 0.3% |
| Zeng et al, 2014 | 26 | 513 | 0.0507 | [0.0334; 0.0734] | 0.3% |
| Yu et al, 2009 | 841 | 3994 | 0.2106 | [0.1980; 0.2235] | 0.4% |
| Zhang et al, 2017 | 236 | 600 | 0.3933 | [0.3540; 0.4337] | 0.3% |
| Cheng et al, 2007 | 109 | 754 | 0.1446 | [0.1202; 0.1717] | 0.3% |
| Cong et al, 2014 | 208 | 890 | 0.2337 | [0.2063; 0.2629] | 0.3% |
| Lin et al, 2004 | 86 | 2588 | 0.0332 | [0.0267; 0.0409] | 0.4% |
| Meng et al, 2015 | 174 | 1500 | 0.1160 | [0.1002; 0.1333] | 0.4% |
| Toole et al, 2006 | 132 | 426 | 0.3099 | [0.2662; 0.3562] | 0.3% |
| Dong et al, 2007 | 2073 | 12052 | 0.1720 | [0.1653; 0.1789] | 0.4% |
| Lu et al, 2009 | 1547 | 8762 | 0.1766 | [0.1686; 0.1847] | 0.4% |
| Li et al, 2019 | 139 | 946 | 0.1469 | [0.1250; 0.1711] | 0.3% |
| Lee et al, 2013 | 2 | 100 | 0.0200 | [0.0024; 0.0704] | 0.3% |
| Rui et al, 2018 | 49 | 225 | 0.2178 | [0.1657; 0.2775] | 0.3% |
| Huang et al, 2013 | 30 | 293 | 0.1024 | [0.0702; 0.1429] | 0.3% |
| Random effects model | | 579696 | 0.2268 | [0.1967; 0.2583] | 14.7% |
| Heterogeneity: $I^2$ = 100%, $\tau^2$ = 0.0146, $p$ = 0 | | | | | |
| Country = Cuba | | | | | |
| Quintana et al, 2005 | 11 | 209 | 0.0526 | [0.0266; 0.0922] | 0.3% |
| Villalba et al, 2014 | 47 | 469 | 0.1002 | [0.0746; 0.1310] | 0.3% |
| Lemos et al, 2000 | 16 | 1149 | 0.0139 | [0.0080; 0.0225] | 0.4% |
| Random effects model | | 1827 | 0.0489 | [0.0070; 0.1255] | 1.0% |
| Heterogeneity: $I^2$ = 97%, $\tau^2$ = 0.0145, $p$ < 0.01 | | | | | |
| Country = Czech | | | | | |
| Straková et al, 2016 | 13 | 230 | 0.0565 | [0.0304; 0.0947] | 0.3% |
| Random effects model | | 230 | 0.0565 | [0.0305; 0.0900] | 0.3% |
| Heterogeneity: not applicable | | | | | |
| Country = Denmark | | | | | |
| Christensen et al,2018 | 293 | 906 | 0.3234 | [0.2930; 0.3549] | 0.3% |
| Langer et al, 1997 | 3 | 553 | 0.0054 | [0.0011; 0.0158] | 0.3% |
| Random effects model | | 1459 | 0.1109 | [0.0000; 0.5744] | 0.7% |
| Heterogeneity: $I^2$ = 100%, $\tau^2$ = 0.1407, $p$ < 0.01 | | | | | |
| Country = Djibouti | | | | | |
| Gambel et al, 1998 | 14 | 112 | 0.1250 | [0.0701; 0.2008] | 0.3% |
| Random effects model | | 112 | 0.1250 | [0.0705; 0.1923] | 0.3% |
| Heterogeneity: not applicable | | | | | |
| Country = Egypt | | | | | |
| Kamel et al, 1995 | 214 | 1259 | 0.1700 | [0.1496; 0.1919] | 0.4% |
| Aboulata et al, 2005 | 26 | 100 | 0.2600 | [0.1774; 0.3573] | 0.3% |
| Hasan et al, 2016 | 33 | 123 | 0.2683 | [0.1924; 0.3557] | 0.3% |
| Fix et al, 2000 | 4283 | 10026 | 0.4272 | [0.4175; 0.4369] | 0.4% |
| Sonia et al, 2006 | 2046 | 2428 | 0.8427 | [0.8276; 0.8569] | 0.4% |
| Gad et al, 2011 | 68 | 116 | 0.5862 | [0.4910; 0.6769] | 0.3% |
| Random effects model | | 14052 | 0.4243 | [0.2040; 0.6619] | 2.1% |
| Heterogeneity: $I^2$ = 100%, $\tau^2$ = 0.0894, $p$ = 0 | | | | | |

| Study | Events | Total | 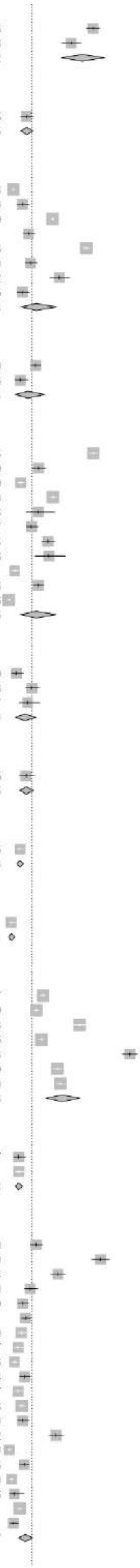 | Proportion | 95% CI | Weight |
|---|---|---|---|---|---|---|
| Country = Ethiopia | | | | | | |
| Niguse et al, 2018 | 359 | 846 | | 0.4243 | [0.3908; 0.4585] | 0.3% |
| Abebe et al, 2017 | 122 | 386 | | 0.3161 | [0.2700; 0.3650] | 0.3% |
| Random effects model | | 1232 | | 0.3709 | [0.2685; 0.4795] | 0.7% |
| Heterogeneity: $I^2$ = 93%, $\tau^2$ = 0.0058, $p$ < 0.01 | | | | | | |
| Country = Finland | | | | | | |
| Kantala et al, 2015 | 37 | 385 | | 0.0961 | [0.0686; 0.1300] | 0.3% |
| Random effects model | | 385 | | 0.0961 | [0.0687; 0.1275] | 0.3% |
| Heterogeneity: not applicable | | | | | | |
| Country = France | | | | | | |
| Boutrouille et al, 2007 | 64 | 1998 | | 0.0320 | [0.0248; 0.0407] | 0.4% |
| Dimeglio et al, 2018 | 23 | 300 | | 0.0767 | [0.0492; 0.1128] | 0.3% |
| Mansuy et al, 2016 | 2371 | 10569 | | 0.2243 | [0.2164; 0.2324] | 0.4% |
| Mansuy et al, 2004 | 46 | 431 | | 0.1067 | [0.0792; 0.1398] | 0.3% |
| Mansuy et al, 2015 | 1310 | 3353 | | 0.3907 | [0.3741; 0.4074] | 0.4% |
| Kaba et al, 2010 | 57 | 490 | | 0.1163 | [0.0893; 0.1481] | 0.3% |
| Chaussade et al, 2013 | 83 | 322 | | 0.2578 | [0.2109; 0.3092] | 0.3% |
| Christophe et al, 2004 | 24 | 315 | | 0.0762 | [0.0494; 0.1112] | 0.3% |
| Random effects model | | 17778 | | 0.1451 | [0.0680; 0.2449] | 2.8% |
| Heterogeneity: $I^2$ = 100%, $\tau^2$ = 0.0331, $p$ < 0.01 | | | | | | |
| Country = Gabon | | | | | | |
| Caron et al, 2008 | 119 | 840 | | 0.1417 | [0.1188; 0.1671] | 0.3% |
| Caron et al, 2012 | 16 | 243 | | 0.0658 | [0.0381; 0.1047] | 0.3% |
| Random effects model | | 1083 | | 0.1023 | [0.0402; 0.1887] | 0.7% |
| Heterogeneity: $I^2$ = 92%, $\tau^2$ = 0.0073, $p$ < 0.01 | | | | | | |
| Country = Germany | | | | | | |
| Wenzel et al, 2014 | 929 | 2184 | | 0.4254 | [0.4045; 0.4464] | 0.4% |
| Juhl et al, 2017 | 70 | 449 | | 0.1559 | [0.1236; 0.1928] | 0.3% |
| Juhl et al, 2014 | 69 | 1019 | | 0.0677 | [0.0531; 0.0849] | 0.3% |
| Mahrt et al, 2018 | 679 | 3000 | | 0.2263 | [0.2115; 0.2417] | 0.4% |
| Krumbholz et al, 2011 | 18 | 116 | | 0.1552 | [0.0946; 0.2341] | 0.3% |
| Krumbholz et al, 2014 | 65 | 537 | | 0.1210 | [0.0947; 0.1517] | 0.3% |
| Jablonka et al, 2017 | 122 | 604 | | 0.2020 | [0.1707; 0.2363] | 0.3% |
| Schielke et al, 2015 | 26 | 126 | | 0.2063 | [0.1394; 0.2875] | 0.3% |
| Nübling et al, 2002 | 20 | 511 | | 0.0391 | [0.0241; 0.0598] | 0.3% |
| Dremsek et al, 2012 | 132 | 853 | | 0.1547 | [0.1311; 0.1808] | 0.3% |
| Krumbholz et al, 2014 | 16 | 1646 | | 0.0097 | [0.0056; 0.0157] | 0.4% |
| Random effects model | | 11045 | | 0.1435 | [0.0669; 0.2430] | 3.8% |
| Heterogeneity: $I^2$ = 99%, $\tau^2$ = 0.0455, $p$ = 0 | | | | | | |
| Country = Ghana | | | | | | |
| Meldal et al, 2013 | 11 | 239 | | 0.0460 | [0.0232; 0.0809] | 0.3% |
| Dorcas et al, 2018 | 48 | 393 | | 0.1221 | [0.0914; 0.1587] | 0.3% |
| Adjei et al, 2009 | 16 | 157 | | 0.1019 | [0.0594; 0.1602] | 0.3% |
| Random effects model | | 789 | | 0.0874 | [0.0439; 0.1438] | 1.0% |
| Heterogeneity: $I^2$ = 83%, $\tau^2$ = 0.0051, $p$ < 0.01 | | | | | | |
| Country = Greece | | | | | | |
| Pittaras et al, 2014 | 25 | 265 | | 0.0943 | [0.0620; 0.1361] | 0.3% |
| Random effects model | | 265 | | 0.0943 | [0.0622; 0.1324] | 0.3% |
| Heterogeneity: not applicable | | | | | | |
| Country = Guyana | | | | | | |
| Talarmin et al, 1997 | 64 | 996 | | 0.0643 | [0.0498; 0.0813] | 0.3% |
| Random effects model | | 996 | | 0.0643 | [0.0499; 0.0803] | 0.3% |
| Heterogeneity: not applicable | | | | | | |
| Country = Iceland | | | | | | |
| Love et al, 2018 | 6 | 291 | | 0.0206 | [0.0076; 0.0443] | 0.3% |
| Random effects model | | 291 | | 0.0206 | [0.0075; 0.0401] | 0.3% |
| Heterogeneity: not applicable | | | | | | |
| Country = India | | | | | | |
| Tripathy et al, 2018 | 433 | 2447 | | 0.1770 | [0.1620; 0.1927] | 0.4% |
| Vivek et al, 2010 | 330 | 2279 | | 0.1448 | [0.1306; 0.1599] | 0.4% |
| Amarapurkar et al, 2008 | 416 | 1163 | | 0.3577 | [0.3301; 0.3860] | 0.4% |
| Kotwal et al, 2014 | 712 | 4175 | | 0.1705 | [0.1592; 0.1823] | 0.4% |
| Katiyar et al, 2018 | 383 | 633 | | 0.6051 | [0.5658; 0.6434] | 0.3% |
| Pathak et al, 2018 | 341 | 1369 | | 0.2491 | [0.2264; 0.2729] | 0.4% |
| Mathur et al, 2001 | 545 | 2070 | | 0.2633 | [0.2444; 0.2828] | 0.4% |
| Random effects model | | 14136 | | 0.2715 | [0.1931; 0.3578] | 2.5% |
| Heterogeneity: $I^2$ = 99%, $\tau^2$ = 0.0156, $p$ < 0.01 | | | | | | |
| Country = Indonesia | | | | | | |
| Widasari et al, 2013 | 16 | 277 | | 0.0578 | [0.0334; 0.0921] | 0.3% |
| Achwan et al, 2007 | 34 | 581 | | 0.0585 | [0.0409; 0.0808] | 0.3% |
| Random effects model | | 858 | | 0.0583 | [0.0436; 0.0749] | 0.7% |
| Heterogeneity: $I^2$ = 0%, $\tau^2$ = 0, $p$ = 0.96 | | | | | | |
| Country = Iran | | | | | | |
| Hassan et al, 2013 | 76 | 530 | | 0.1434 | [0.1147; 0.1762] | 0.3% |
| Fatemeh et al, 2014 | 235 | 510 | | 0.4608 | [0.4169; 0.5051] | 0.3% |
| Azarkar et al, 2017 | 139 | 554 | | 0.2509 | [0.2153; 0.2892] | 0.3% |
| Assarehzadegan et al, 2007 | 46 | 400 | | 0.1150 | [0.0854; 0.1504] | 0.3% |
| Taremi et al, 2007 | 31 | 399 | | 0.0777 | [0.0534; 0.1085] | 0.3% |
| Mohebbi et al, 2012 | 51 | 551 | | 0.0926 | [0.0697; 0.1199] | 0.3% |
| Parsa et al, 2016 | 50 | 700 | | 0.0714 | [0.0535; 0.0931] | 0.3% |
| Jahromi et al, 2013 | 26 | 477 | | 0.0545 | [0.0359; 0.0788] | 0.3% |
| Ataei et al, 2007 | 31 | 816 | | 0.0380 | [0.0260; 0.0535] | 0.3% |
| Hesamizadeh et al, 2015 | 45 | 514 | | 0.0875 | [0.0646; 0.1154] | 0.3% |
| Jahromi et al, 2013 | 26 | 477 | | 0.0545 | [0.0359; 0.0788] | 0.3% |
| Sepanlou et al, 2010 | 105 | 1423 | | 0.0738 | [0.0607; 0.0886] | 0.4% |
| Raoofi et al, 2012 | 31 | 400 | | 0.0775 | [0.0533; 0.1082] | 0.3% |
| Jahanbakhsh et al, 2017 | 136 | 562 | | 0.2420 | [0.2071; 0.2796] | 0.3% |
| Ghorbani et al, 2007 | 9 | 800 | | 0.0112 | [0.0052; 0.0212] | 0.3% |
| Shamsizadeh et al, 2009 | 48 | 566 | | 0.0848 | [0.0632; 0.1109] | 0.3% |
| Saffar et al, 2009 | 25 | 1080 | | 0.0231 | [0.0150; 0.0340] | 0.3% |
| Zakieh et al, 2013 | 5 | 136 | | 0.0368 | [0.0120; 0.0837] | 0.3% |
| Farshadpour et al, 2018 | 83 | 1331 | | 0.0624 | [0.0500; 0.0767] | 0.4% |
| Alizadeh et al, 2016 | 10 | 321 | | 0.0312 | [0.0150; 0.0565] | 0.3% |
| Random effects model | | 12547 | | 0.0898 | [0.0574; 0.1286] | 7.0% |
| Heterogeneity: $I^2$ = 98%, $\tau^2$ = 0.0197, $p$ < 0.01 | | | | | | |

| Study | Events | Total | Proportion | 95% CI | Weight |
|---|---|---|---|---|---|
| **Country = Ireland** | | | | | |
| O'Riordan et al, 2016 | 57 | 1076 | 0.0530 | [0.0404; 0.0681] | 0.3% |
| Hickey et al, 2016 | 16 | 198 | 0.0808 | [0.0469; 0.1279] | 0.3% |
| Random effects model | | 1274 | 0.0617 | [0.0392; 0.0890] | 0.7% |
| Heterogeneity: $I^2 = 52\%$, $\tau^2 = 0.0008$, $p = 0.15$ | | | | | |
| **Country = Israel** | | | | | |
| Mor et al, 2015 | 77 | 729 | 0.1056 | [0.0843; 0.1302] | 0.3% |
| Karetny et al, 1995 | 37 | 1416 | 0.0261 | [0.0185; 0.0358] | 0.4% |
| Lachish et al, 2013 | 19 | 4970 | 0.0038 | [0.0023; 0.0060] | 0.4% |
| Random effects model | | 7115 | 0.0336 | [0.0019; 0.1022] | 1.1% |
| Heterogeneity: $I^2 = 99\%$, $\tau^2 = 0.0154$, $p < 0.01$ | | | | | |
| **Country = Italy** | | | | | |
| Spada et al, 2018 | 869 | 10011 | 0.0868 | [0.0814; 0.0925] | 0.4% |
| Gessoni et al, 1996 | 49 | 1889 | 0.0259 | [0.0193; 0.0341] | 0.4% |
| Ricco et al, 2016 | 9 | 199 | 0.0452 | [0.0209; 0.0841] | 0.3% |
| Puttini et al, 2015 | 12 | 132 | 0.0909 | [0.0479; 0.1534] | 0.3% |
| Pavia et al, 1998 | 3 | 360 | 0.0083 | [0.0017; 0.0242] | 0.3% |
| Zuin et al, 2016 | 380 | 3546 | 0.1072 | [0.0972; 0.1178] | 0.4% |
| Scotto et al, 2012 | 2 | 151 | 0.0132 | [0.0016; 0.0470] | 0.3% |
| Sabato et al, 2017 | 4 | 253 | 0.0158 | [0.0043; 0.0400] | 0.3% |
| Lucarelli et al, 2016 | 153 | 313 | 0.4888 | [0.4322; 0.5457] | 0.3% |
| Marcantonio et al, 2018 | 10 | 198 | 0.0505 | [0.0245; 0.0909] | 0.3% |
| Lanini et al, 2015 | 60 | 1116 | 0.0538 | [0.0413; 0.0687] | 0.4% |
| Rapicetta et al,1999 | 113 | 973 | 0.1161 | [0.0967; 0.1380] | 0.3% |
| Tabili et al, 2013 | 23 | 103 | 0.2233 | [0.1471; 0.3160] | 0.3% |
| Alecci et al, 1997 | 3 | 244 | 0.0123 | [0.0025; 0.0355] | 0.3% |
| Random effects model | | 19488 | 0.0728 | [0.0454; 0.1060] | 4.9% |
| Heterogeneity: $I^2 = 98\%$, $\tau^2 = 0.0116$, $p < 0.01$ | | | | | |
| **Country = Japan** | | | | | |
| Takahashi et al, 2010 | 1167 | 22027 | 0.0530 | [0.0501; 0.0560] | 0.4% |
| Takeda et al, 2010 | 431 | 12600 | 0.0342 | [0.0311; 0.0375] | 0.4% |
| Sakata et al, 2008 | 45 | 1389 | 0.0324 | [0.0237; 0.0431] | 0.4% |
| Toyoda et al, 2008 | 60 | 592 | 0.1014 | [0.0782; 0.1285] | 0.3% |
| Fukuda et al, 2007 | 168 | 3185 | 0.0527 | [0.0452; 0.0611] | 0.4% |
| Ding et al, 2003 | 15 | 533 | 0.0281 | [0.0158; 0.0460] | 0.3% |
| Mitsui et al, 2005 | 6 | 266 | 0.0226 | [0.0083; 0.0484] | 0.3% |
| Goto et al, 2006 | 9 | 344 | 0.0262 | [0.0120; 0.0491] | 0.3% |
| Random effects model | | 40936 | 0.0426 | [0.0327; 0.0537] | 2.8% |
| Heterogeneity: $I^2 = 94\%$, $\tau^2 = 0.0011$, $p < 0.01$ | | | | | |
| **Country = Jordan** | | | | | |
| Mohammad et al, 2017 | 139 | 450 | 0.3089 | [0.2665; 0.3538] | 0.3% |
| Random effects model | | 450 | 0.3089 | [0.2671; 0.3523] | 0.3% |
| Heterogeneity: not applicable | | | | | |
| **Country = Kazakhstan** | | | | | |
| Cainelli et al, 2018 | 11 | 199 | 0.0553 | [0.0279; 0.0968] | 0.3% |
| Random effects model | | 199 | 0.0553 | [0.0279; 0.0912] | 0.3% |
| Heterogeneity: not applicable | | | | | |
| **Country = Korea** | | | | | |
| Ahn et al, 2005 | 138 | 1030 | 0.1340 | [0.1138; 0.1563] | 0.3% |
| Yoon et al, 2014 | 144 | 2450 | 0.0588 | [0.0498; 0.0688] | 0.4% |
| Yun et al, 2011 | 46 | 489 | 0.0941 | [0.0697; 0.1235] | 0.3% |
| Random effects model | | 3969 | 0.0930 | [0.0487; 0.1496] | 1.0% |
| Heterogeneity: $I^2 = 96\%$, $\tau^2 = 0.0057$, $p < 0.01$ | | | | | |
| **Country = Laos** | | | | | |
| Khounvisith et al, 2018 | 38 | 210 | 0.1810 | [0.1313; 0.2398] | 0.3% |
| Random effects model | | 210 | 0.1810 | [0.1320; 0.2358] | 0.3% |
| Heterogeneity: not applicable | | | | | |
| **Country = Malawi** | | | | | |
| Taha et al, 2015 | 80 | 397 | 0.2015 | [0.1631; 0.2444] | 0.3% |
| Random effects model | | 397 | 0.2015 | [0.1636; 0.2423] | 0.3% |
| Heterogeneity: not applicable | | | | | |
| **Country = Mexico** | | | | | |
| Esquivel et al, 2014 | 100 | 273 | 0.3663 | [0.3090; 0.4265] | 0.3% |
| Munoz et al, 1999 | 374 | 3549 | 0.1054 | [0.0955; 0.1160] | 0.4% |
| Héctor et al, 2017 | 1 | 127 | 0.0079 | [0.0002; 0.0431] | 0.3% |
| Cosme et al, 2014 | 25 | 439 | 0.0569 | [0.0372; 0.0829] | 0.3% |
| Esquivel et al, 2015 | 10 | 150 | 0.0667 | [0.0324; 0.1192] | 0.3% |
| Esquivel et al, 2015 | 25 | 439 | 0.0569 | [0.0372; 0.0829] | 0.3% |
| Random effects model | | 4977 | 0.0897 | [0.0375; 0.1614] | 2.1% |
| Heterogeneity: $I^2 = 97\%$, $\tau^2 = 0.0177$, $p < 0.01$ | | | | | |
| **Country = Moldova** | | | | | |
| Drobeniuc et al, 2001 | 63 | 255 | 0.2471 | [0.1954; 0.3047] | 0.3% |
| Random effects model | | 255 | 0.2471 | [0.1962; 0.3018] | 0.3% |
| Heterogeneity: not applicable | | | | | |
| **Country = Mongolia** | | | | | |
| Takahashi et al, 2004 | 28 | 249 | 0.1124 | [0.0760; 0.1584] | 0.3% |
| Davaalkham et al, 2009 | 3 | 520 | 0.0058 | [0.0012; 0.0168] | 0.3% |
| Bira et al, 2007 | 5 | 717 | 0.0070 | [0.0023; 0.0162] | 0.3% |
| Random effects model | | 1486 | 0.0270 | [0.0006; 0.0908] | 1.0% |
| Heterogeneity: $I^2 = 96\%$, $\tau^2 = 0.0149$, $p < 0.01$ | | | | | |

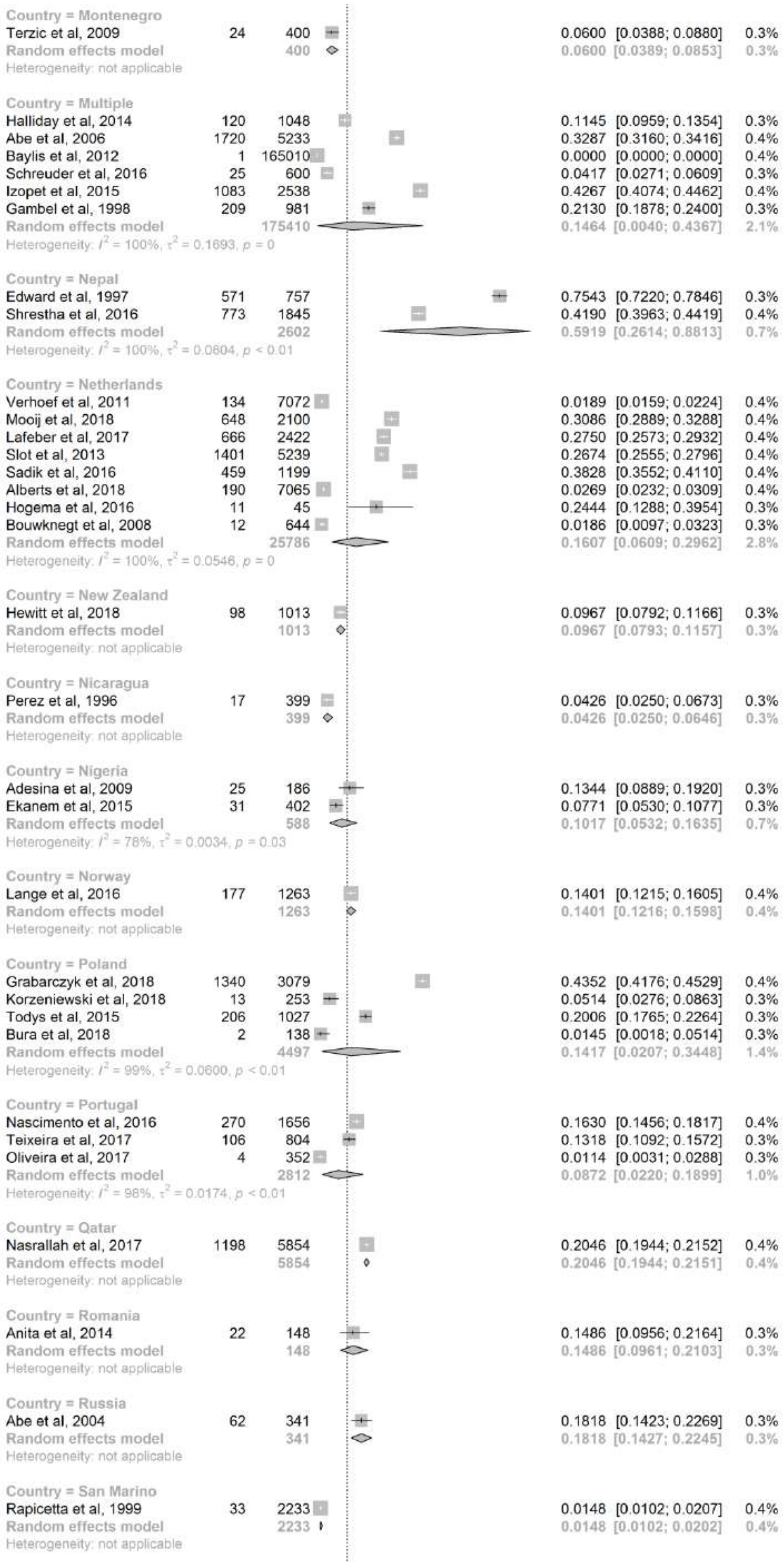

Country = Montenegro
Terzic et al, 2009 24 400 0.0600 [0.0388; 0.0880] 0.3%
Random effects model 400 0.0600 [0.0389; 0.0853] 0.3%
Heterogeneity: not applicable
Country = Multiple
Halliday et al, 2014 120 1048 0.1145 [0.0959; 0.1354] 0.3%
Abe et al, 2006 1720 5233 0.3287 [0.3160; 0.3416] 0.4%
Baylis et al, 2012 1 165010 0.0000 [0.0000; 0.0000] 0.4%
Schreuder et al, 2016 25 600 0.0417 [0.0271; 0.0609] 0.3%
Izopet et al, 2015 1083 2538 0.4267 [0.4074; 0.4462] 0.4%
Gambel et al, 1998 209 981 0.2130 [0.1878; 0.2400] 0.3%
Random effects model 175410 0.1464 [0.0040; 0.4367] 2.1%
Heterogeneity: $I^2$ = 100%, $\tau^2$ = 0.1693, $p$ = 0
Country = Nepal
Edward et al, 1997 571 757 0.7543 [0.7220; 0.7846] 0.3%
Shrestha et al, 2016 773 1845 0.4190 [0.3963; 0.4419] 0.4%
Random effects model 2602 0.5919 [0.2614; 0.8813] 0.7%
Heterogeneity: $I^2$ = 100%, $\tau^2$ = 0.0604, $p$ < 0.01
Country = Netherlands
Verhoef et al, 2011 134 7072 0.0189 [0.0159; 0.0224] 0.4%
Mooij et al, 2018 648 2100 0.3086 [0.2889; 0.3288] 0.4%
Lafeber et al, 2017 666 2422 0.2750 [0.2573; 0.2932] 0.4%
Slot et al, 2013 1401 5239 0.2674 [0.2555; 0.2796] 0.4%
Sadik et al, 2016 459 1199 0.3828 [0.3552; 0.4110] 0.4%
Alberts et al, 2018 190 7065 0.0269 [0.0232; 0.0309] 0.4%
Hogema et al, 2016 11 45 0.2444 [0.1288; 0.3954] 0.3%
Bouwknegt et al, 2008 12 644 0.0186 [0.0097; 0.0323] 0.3%
Random effects model 25786 0.1607 [0.0609; 0.2962] 2.8%
Heterogeneity: $I^2$ = 100%, $\tau^2$ = 0.0546, $p$ = 0
Country = New Zealand
Hewitt et al, 2018 98 1013 0.0967 [0.0792; 0.1166] 0.3%
Random effects model 1013 0.0967 [0.0793; 0.1157] 0.3%
Heterogeneity: not applicable
Country = Nicaragua
Perez et al, 1996 17 399 0.0426 [0.0250; 0.0673] 0.3%
Random effects model 399 0.0426 [0.0250; 0.0646] 0.3%
Heterogeneity: not applicable
Country = Nigeria
Adesina et al, 2009 25 186 0.1344 [0.0889; 0.1920] 0.3%
Ekanem et al, 2015 31 402 0.0771 [0.0530; 0.1077] 0.3%
Random effects model 588 0.1017 [0.0532; 0.1635] 0.7%
Heterogeneity: $I^2$ = 78%, $\tau^2$ = 0.0034, $p$ = 0.03
Country = Norway
Lange et al, 2016 177 1263 0.1401 [0.1215; 0.1605] 0.4%
Random effects model 1263 0.1401 [0.1216; 0.1598] 0.4%
Heterogeneity: not applicable
Country = Poland
Grabarczyk et al, 2018 1340 3079 0.4352 [0.4176; 0.4529] 0.4%
Korzeniewski et al, 2018 13 253 0.0514 [0.0276; 0.0863] 0.3%
Todys et al, 2015 206 1027 0.2006 [0.1765; 0.2264] 0.3%
Bura et al, 2018 2 138 0.0145 [0.0018; 0.0514] 0.3%
Random effects model 4497 0.1417 [0.0207; 0.3448] 1.4%
Heterogeneity: $I^2$ = 99%, $\tau^2$ = 0.0600, $p$ < 0.01
Country = Portugal
Nascimento et al, 2016 270 1656 0.1630 [0.1456; 0.1817] 0.4%
Teixeira et al, 2017 106 804 0.1318 [0.1092; 0.1572] 0.3%
Oliveira et al, 2017 4 352 0.0114 [0.0031; 0.0288] 0.3%
Random effects model 2812 0.0872 [0.0220; 0.1899] 1.0%
Heterogeneity: $I^2$ = 98%, $\tau^2$ = 0.0174, $p$ < 0.01
Country = Qatar
Nasrallah et al, 2017 1198 5854 0.2046 [0.1944; 0.2152] 0.4%
Random effects model 5854 0.2046 [0.1944; 0.2151] 0.4%
Heterogeneity: not applicable
Country = Romania
Anita et al, 2014 22 148 0.1486 [0.0956; 0.2164] 0.3%
Random effects model 148 0.1486 [0.0961; 0.2103] 0.3%
Heterogeneity: not applicable
Country = Russia
Abe et al, 2004 62 341 0.1818 [0.1423; 0.2269] 0.3%
Random effects model 341 0.1818 [0.1427; 0.2245] 0.3%
Heterogeneity: not applicable
Country = San Marino
Rapicetta et al, 1999 33 2233 0.0148 [0.0102; 0.0207] 0.4%
Random effects model 2233 0.0148 [0.0102; 0.0202] 0.4%
Heterogeneity: not applicable

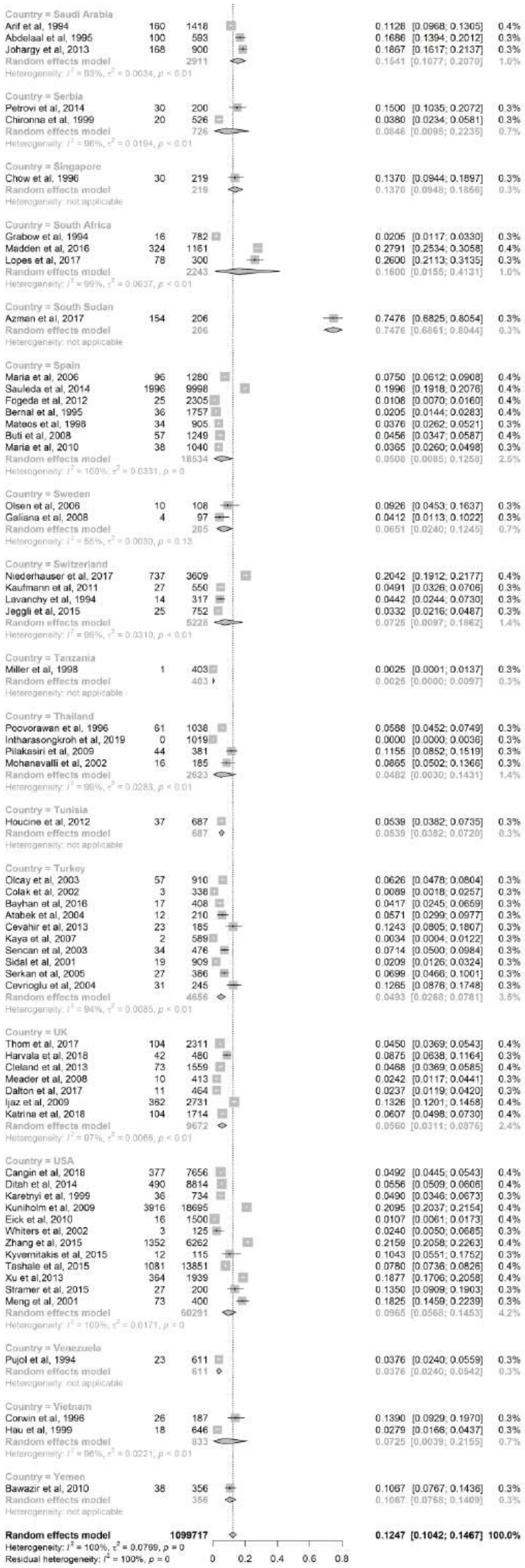
Country = Saudi Arabia
Arif et al, 1994 160 1418 0.1128 [0.0968; 0.1305] 0.4%
Abdelaal et al, 1995 100 593 0.1686 [0.1394; 0.2012] 0.3%
Johargy et al, 2013 168 900 0.1867 [0.1617; 0.2137] 0.3%
Random effects model 2911 0.1541 [0.1077; 0.2070] 1.0%
Heterogeneity: $I^2$ = 83%, $\tau^2$ = 0.0034, $p$ < 0.01
Country = Serbia
Petrovi et al, 2014 30 200 0.1500 [0.1035; 0.2072] 0.3%
Chironna et al, 1999 20 526 0.0380 [0.0234; 0.0581] 0.3%
Random effects model 726 0.0846 [0.0095; 0.2235] 0.7%
Heterogeneity: $I^2$ = 96%, $\tau^2$ = 0.0194, $p$ < 0.01
Country = Singapore
Chow et al, 1996 30 219 0.1370 [0.0944; 0.1897] 0.3%
Random effects model 219 0.1370 [0.0948; 0.1866] 0.3%
Heterogeneity: not applicable
Country = South Africa
Grabow et al, 1994 16 782 0.0205 [0.0117; 0.0330] 0.3%
Madden et al, 2016 324 1161 0.2791 [0.2534; 0.3058] 0.4%
Lopes et al, 2017 78 300 0.2600 [0.2113; 0.3135] 0.3%
Random effects model 2243 0.1600 [0.0155; 0.4131] 1.0%
Heterogeneity: $I^2$ = 99%, $\tau^2$ = 0.0637, $p$ < 0.01
Country = South Sudan
Azman et al, 2017 154 206 0.7476 [0.6825; 0.8054] 0.3%
Random effects model 206 0.7476 [0.6861; 0.8044] 0.3%
Heterogeneity: not applicable
Country = Spain
Maria et al, 2006 96 1280 0.0750 [0.0612; 0.0908] 0.4%
Sauleda et al, 2014 1996 9998 0.1996 [0.1918; 0.2076] 0.4%
Fogeda et al, 2012 25 2305 0.0108 [0.0070; 0.0160] 0.4%
Bernal et al, 1995 36 1757 0.0205 [0.0144; 0.0283] 0.4%
Mateos et al, 1998 34 905 0.0376 [0.0262; 0.0521] 0.3%
Buti et al, 2008 57 1249 0.0456 [0.0347; 0.0587] 0.4%
Maria et al, 2010 38 1040 0.0365 [0.0260; 0.0498] 0.3%
Random effects model 18534 0.0506 [0.0085; 0.1258] 2.5%
Heterogeneity: $I^2$ = 100%, $\tau^2$ = 0.0331, $p$ = 0
Country = Sweden
Olsen et al, 2006 10 108 0.0926 [0.0453; 0.1637] 0.3%
Galiana et al, 2008 4 97 0.0412 [0.0113; 0.1022] 0.3%
Random effects model 205 0.0651 [0.0240; 0.1245] 0.7%
Heterogeneity: $I^2$ = 55%, $\tau^2$ = 0.0030, $p$ = 0.13
Country = Switzerland
Niederhauser et al, 2017 737 3609 0.2042 [0.1912; 0.2177] 0.4%
Kaufmann et al, 2011 27 550 0.0491 [0.0326; 0.0706] 0.3%
Lavanchy et al, 1994 14 317 0.0442 [0.0244; 0.0730] 0.3%
Jeggli et al, 2015 25 752 0.0332 [0.0216; 0.0487] 0.3%
Random effects model 5228 0.0725 [0.0097; 0.1862] 1.4%
Heterogeneity: $I^2$ = 99%, $\tau^2$ = 0.0310, $p$ < 0.01
Country = Tanzania
Miller et al, 1998 1 403 0.0025 [0.0001; 0.0137] 0.3%
Random effects model 403 0.0025 [0.0000; 0.0097] 0.3%
Heterogeneity: not applicable
Country = Thailand
Poovorawan et al, 1996 61 1038 0.0588 [0.0452; 0.0749] 0.3%
Intharasongkroh et al, 2019 0 1019 0.0000 [0.0000; 0.0036] 0.3%
Pilakasiri et al, 2009 44 381 0.1155 [0.0852; 0.1519] 0.3%
Mohanavalli et al, 2002 16 185 0.0865 [0.0502; 0.1366] 0.3%
Random effects model 2623 0.0482 [0.0030; 0.1431] 1.4%
Heterogeneity: $I^2$ = 98%, $\tau^2$ = 0.0283, $p$ < 0.01
Country = Tunisia
Houcine et al, 2012 37 687 0.0539 [0.0382; 0.0735] 0.3%
Random effects model 687 0.0539 [0.0382; 0.0720] 0.3%
Heterogeneity: not applicable
Country = Turkey
Olcay et al, 2003 57 910 0.0626 [0.0478; 0.0804] 0.3%
Colak et al, 2002 3 338 0.0089 [0.0018; 0.0257] 0.3%
Bayhan et al, 2016 17 408 0.0417 [0.0245; 0.0659] 0.3%
Atabek et al, 2004 12 210 0.0571 [0.0299; 0.0977] 0.3%
Cevahir et al, 2013 23 185 0.1243 [0.0805; 0.1807] 0.3%
Kaya et al, 2007 2 589 0.0034 [0.0004; 0.0122] 0.3%
Sencan et al, 2003 34 476 0.0714 [0.0500; 0.0984] 0.3%
Sidal et al, 2001 19 909 0.0209 [0.0126; 0.0324] 0.3%
Serkan et al, 2005 27 386 0.0699 [0.0466; 0.1001] 0.3%
Cevrioglu et al, 2004 31 245 0.1265 [0.0876; 0.1748] 0.3%
Random effects model 4656 0.0493 [0.0268; 0.0781] 3.5%
Heterogeneity: $I^2$ = 94%, $\tau^2$ = 0.0085, $p$ < 0.01
Country = UK
Thom et al, 2017 104 2311 0.0450 [0.0369; 0.0543] 0.4%
Harvala et al, 2018 42 480 0.0875 [0.0638; 0.1164] 0.3%
Cleland et al, 2013 73 1559 0.0468 [0.0369; 0.0585] 0.4%
Meader et al, 2008 10 413 0.0242 [0.0117; 0.0441] 0.3%
Dalton et al, 2017 11 464 0.0237 [0.0119; 0.0420] 0.3%
Ijaz et al, 2009 362 2731 0.1326 [0.1201; 0.1458] 0.4%
Katrina et al, 2018 104 1714 0.0607 [0.0498; 0.0730] 0.4%
Random effects model 9672 0.0560 [0.0311; 0.0876] 2.4%
Heterogeneity: $I^2$ = 97%, $\tau^2$ = 0.0066, $p$ < 0.01
Country = USA
Cangin et al, 2018 377 7656 0.0492 [0.0445; 0.0543] 0.4%
Ditah et al, 2014 490 8814 0.0556 [0.0509; 0.0606] 0.4%
Karetnyi et al, 1999 36 734 0.0490 [0.0346; 0.0673] 0.3%
Kuniholm et al, 2009 3916 18695 0.2095 [0.2037; 0.2154] 0.4%
Eick et al, 2010 16 1500 0.0107 [0.0061; 0.0173] 0.4%
Whiters et al, 2002 3 125 0.0240 [0.0050; 0.0685] 0.3%
Zhang et al, 2015 1352 6262 0.2159 [0.2058; 0.2263] 0.4%
Kyvernitakis et al, 2015 12 115 0.1043 [0.0551; 0.1752] 0.3%
Tashale et al, 2015 1081 13851 0.0780 [0.0736; 0.0826] 0.4%
Xu et al,2013 364 1939 0.1877 [0.1706; 0.2058] 0.4%
Stramer et al, 2015 27 200 0.1350 [0.0909; 0.1903] 0.3%
Meng et al, 2001 73 400 0.1825 [0.1459; 0.2239] 0.3%
Random effects model 60291 0.0965 [0.0568; 0.1453] 4.2%
Heterogeneity: $I^2$ = 100%, $\tau^2$ = 0.0171, $p$ = 0
Country = Venezuela
Pujol et al, 1994 23 611 0.0376 [0.0240; 0.0559] 0.3%
Random effects model 611 0.0376 [0.0240; 0.0542] 0.3%
Heterogeneity: not applicable
Country = Vietnam
Corwin et al, 1996 26 187 0.1390 [0.0929; 0.1970] 0.3%
Hau et al, 1999 18 646 0.0279 [0.0166; 0.0437] 0.3%
Random effects model 833 0.0725 [0.0039; 0.2155] 0.7%
Heterogeneity: $I^2$ = 96%, $\tau^2$ = 0.0221, $p$ < 0.01
Country = Yemen
Bawazir et al, 2010 38 356 0.1067 [0.0767; 0.1436] 0.3%
Random effects model 356 0.1067 [0.0768; 0.1409] 0.3%
Heterogeneity: not applicable
Random effects model 1099717 0.1247 [0.1042; 0.1467] 100.0%
Heterogeneity: $I^2$ = 100%, $\tau^2$ = 0.0769, $p$ = 0
Residual heterogeneity: $I^2$ = 100%, $p$ = 0
0 0.2 0.4 0.6 0.8

**Figure S2. Forest plot of estimated pooled anti-HEV IgM seroprevalence among general population based on different countries.**

Note: country named multiple means people in the study are from more than one country.

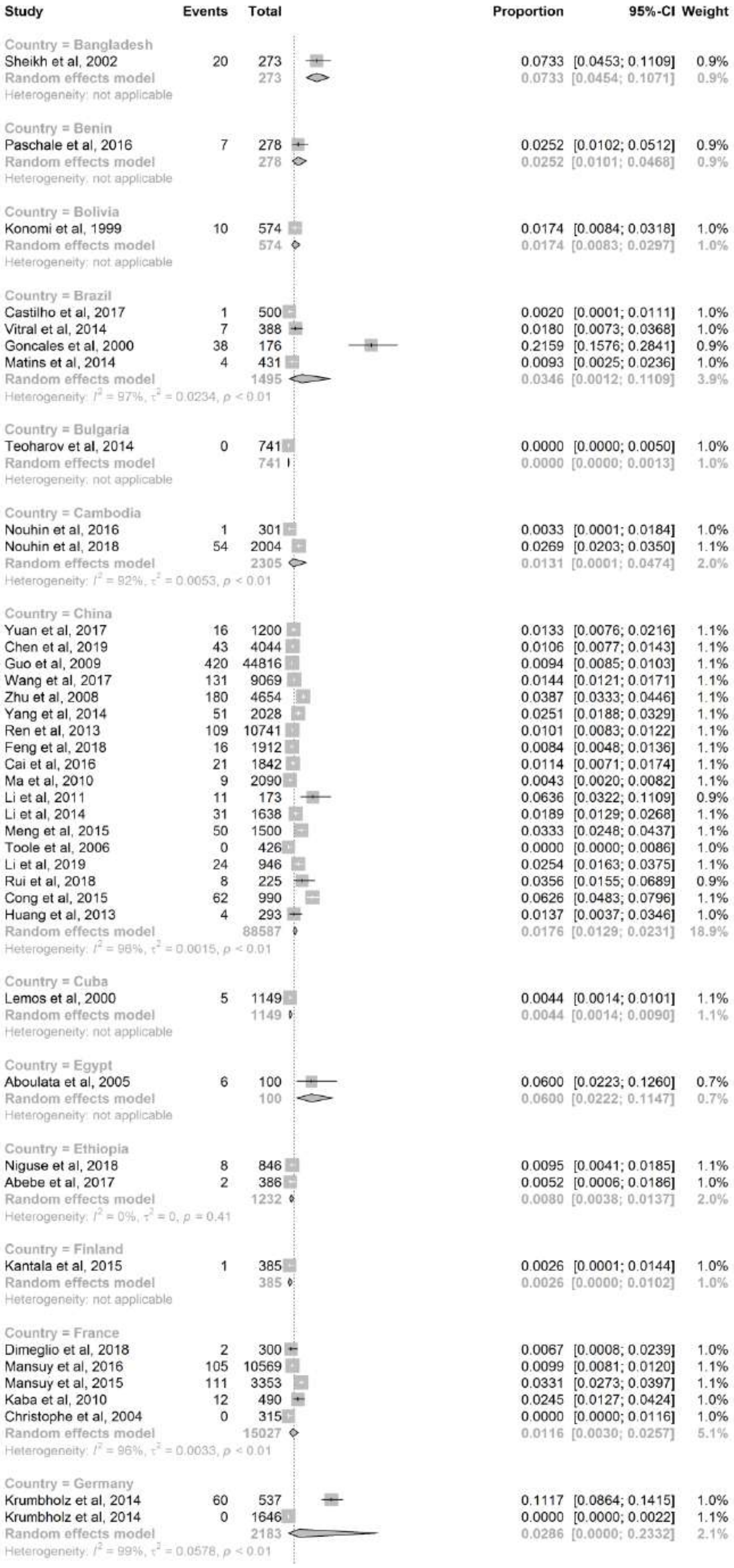

| Study | Events | Total | Proportion | 95%-CI | Weight |
|---|---|---|---|---|---|
| **Country = Ghana** | | | | | |
| Meldal et al, 2013 | 14 | 239 | 0.0586 | [0.0324; 0.0963] | 0.9% |
| Dorcas et al, 2018 | 1 | 393 | 0.0025 | [0.0001; 0.0141] | 1.0% |
| Adjei et al, 2009 | 29 | 157 | 0.1847 | [0.1273; 0.2544] | 0.8% |
| Random effects model | | 789 | 0.0587 | [0.0006; 0.2010] | 2.8% |
| Heterogeneity: $I^2 = 97\%$, $\tau^2 = 0.0368$, $p < 0.01$ | | | | | |
| **Country = India** | | | | | |
| Tripathy et al, 2018 | 5 | 2447 | 0.0020 | [0.0007; 0.0048] | 1.1% |
| Gajjar et al, 2014 | 22 | 460 | 0.0478 | [0.0302; 0.0715] | 1.0% |
| Gupta et al, 2015 | 24 | 252 | 0.0952 | [0.0620; 0.1384] | 0.9% |
| Mathur et al, 2001 | 244 | 2070 | 0.1179 | [0.1043; 0.1326] | 1.1% |
| Premashis et al, 2008 | 53 | 125 | 0.4240 | [0.3361; 0.5156] | 0.8% |
| Random effects model | | 5354 | 0.1018 | [0.0208; 0.2338] | 4.9% |
| Heterogeneity: $I^2 = 99\%$, $\tau^2 = 0.0414$, $p < 0.01$ | | | | | |
| **Country = Iran** | | | | | |
| Fatemeh et al, 2014 | 7 | 510 | 0.0137 | [0.0055; 0.0281] | 1.0% |
| Asaei et al, 2015 | 9 | 1030 | 0.0087 | [0.0040; 0.0165] | 1.1% |
| Jahromi et al, 2013 | 11 | 477 | 0.0231 | [0.0116; 0.0409] | 1.0% |
| Ghorbani et al, 2007 | 0 | 800 | 0.0000 | [0.0000; 0.0046] | 1.0% |
| Farshadpour et al, 2018 | 11 | 1331 | 0.0083 | [0.0041; 0.0147] | 1.1% |
| Random effects model | | 4148 | 0.0080 | [0.0019; 0.0182] | 5.2% |
| Heterogeneity: $I^2 = 88\%$, $\tau^2 = 0.0024$, $p < 0.01$ | | | | | |
| **Country = Ireland** | | | | | |
| O'Riordan et al, 2016 | 2 | 1076 | 0.0019 | [0.0002; 0.0067] | 1.1% |
| Random effects model | | 1076 | 0.0019 | [0.0002; 0.0053] | 1.1% |
| Heterogeneity: not applicable | | | | | |
| **Country = Italy** | | | | | |
| Spada et al, 2018 | 46 | 10011 | 0.0046 | [0.0034; 0.0061] | 1.1% |
| Ricco et al, 2016 | 1 | 199 | 0.0050 | [0.0001; 0.0277] | 0.9% |
| Scotto et al, 2012 | 0 | 151 | 0.0000 | [0.0000; 0.0241] | 0.8% |
| Marcantonio et al, 2018 | 2 | 198 | 0.0101 | [0.0012; 0.0360] | 0.9% |
| Random effects model | | 10559 | 0.0044 | [0.0019; 0.0078] | 3.7% |
| Heterogeneity: $I^2 = 18\%$, $\tau^2 = 0.0002$, $p = 0.30$ | | | | | |
| **Country = Japan** | | | | | |
| Takahashi et al, 2010 | 9 | 22027 | 0.0004 | [0.0002; 0.0008] | 1.1% |
| Sakata et al, 2008 | 14 | 1389 | 0.0101 | [0.0055; 0.0169] | 1.1% |
| Fukuda et al, 2007 | 6 | 3185 | 0.0019 | [0.0007; 0.0041] | 1.1% |
| Ding et al, 2003 | 1 | 533 | 0.0019 | [0.0000; 0.0104] | 1.0% |
| Goto et al, 2006 | 2 | 344 | 0.0058 | [0.0007; 0.0208] | 1.0% |
| Random effects model | | 27478 | 0.0030 | [0.0006; 0.0073] | 5.3% |
| Heterogeneity: $I^2 = 90\%$, $\tau^2 = 0.0010$, $p < 0.01$ | | | | | |
| **Country = Korea** | | | | | |
| Ahn et al, 2005 | 9 | 1030 | 0.0087 | [0.0040; 0.0165] | 1.1% |
| Random effects model | | 1030 | 0.0087 | [0.0040; 0.0153] | 1.1% |
| Heterogeneity: not applicable | | | | | |
| **Country = Mongolia** | | | | | |
| Davaalkham et al, 2009 | 0 | 520 | 0.0000 | [0.0000; 0.0071] | 1.0% |
| Bira et al, 2007 | 0 | 717 | 0.0000 | [0.0000; 0.0051] | 1.0% |
| Random effects model | | 1237 | 0.0000 | [0.0000; 0.0008] | 2.1% |
| Heterogeneity: $I^2 = 0\%$, $\tau^2 = 0$, $p = 1.00$ | | | | | |
| **Country = Multiple** | | | | | |
| Abe et al, 2006 | 14 | 5233 | 0.0027 | [0.0015; 0.0045] | 1.1% |
| Baylis et al, 2012 | 1 | 165010 | 0.0000 | [0.0000; 0.0000] | 1.1% |
| Minuk et al, 2007 | 2 | 393 | 0.0051 | [0.0006; 0.0183] | 1.0% |
| Schreuder et al, 2016 | 1 | 600 | 0.0017 | [0.0000; 0.0093] | 1.0% |
| Random effects model | | 171236 | 0.0015 | [0.0000; 0.0056] | 4.2% |
| Heterogeneity: $I^2 = 95\%$, $\tau^2 = 0.0012$, $p < 0.01$ | | | | | |
| **Country = Nepal** | | | | | |
| Shrestha et al, 2016 | 54 | 1686 | 0.0320 | [0.0242; 0.0416] | 1.1% |
| Random effects model | | 1686 | 0.0320 | [0.0242; 0.0410] | 1.1% |
| Heterogeneity: not applicable | | | | | |
| **Country = Netherlands** | | | | | |
| Slot et al, 2013 | 49 | 5239 | 0.0094 | [0.0069; 0.0123] | 1.1% |
| Sadik et al, 2016 | 7 | 1199 | 0.0058 | [0.0024; 0.0120] | 1.1% |
| Alberts et al, 2018 | 435 | 7065 | 0.0616 | [0.0561; 0.0674] | 1.1% |
| Random effects model | | 13503 | 0.0200 | [0.0005; 0.0663] | 3.3% |
| Heterogeneity: $I^2 = 99\%$, $\tau^2 = 0.0109$, $p < 0.01$ | | | | | |
| **Country = Nigeria** | | | | | |
| Ifeorah et al, 2017 | 1 | 750 | 0.0013 | [0.0000; 0.0074] | 1.0% |
| Fowotade et al, 2018 | 16 | 177 | 0.0904 | [0.0526; 0.1426] | 0.9% |
| Random effects model | | 927 | 0.0283 | [0.0000; 0.1756] | 1.9% |
| Heterogeneity: $I^2 = 98\%$, $\tau^2 = 0.0353$, $p < 0.01$ | | | | | |

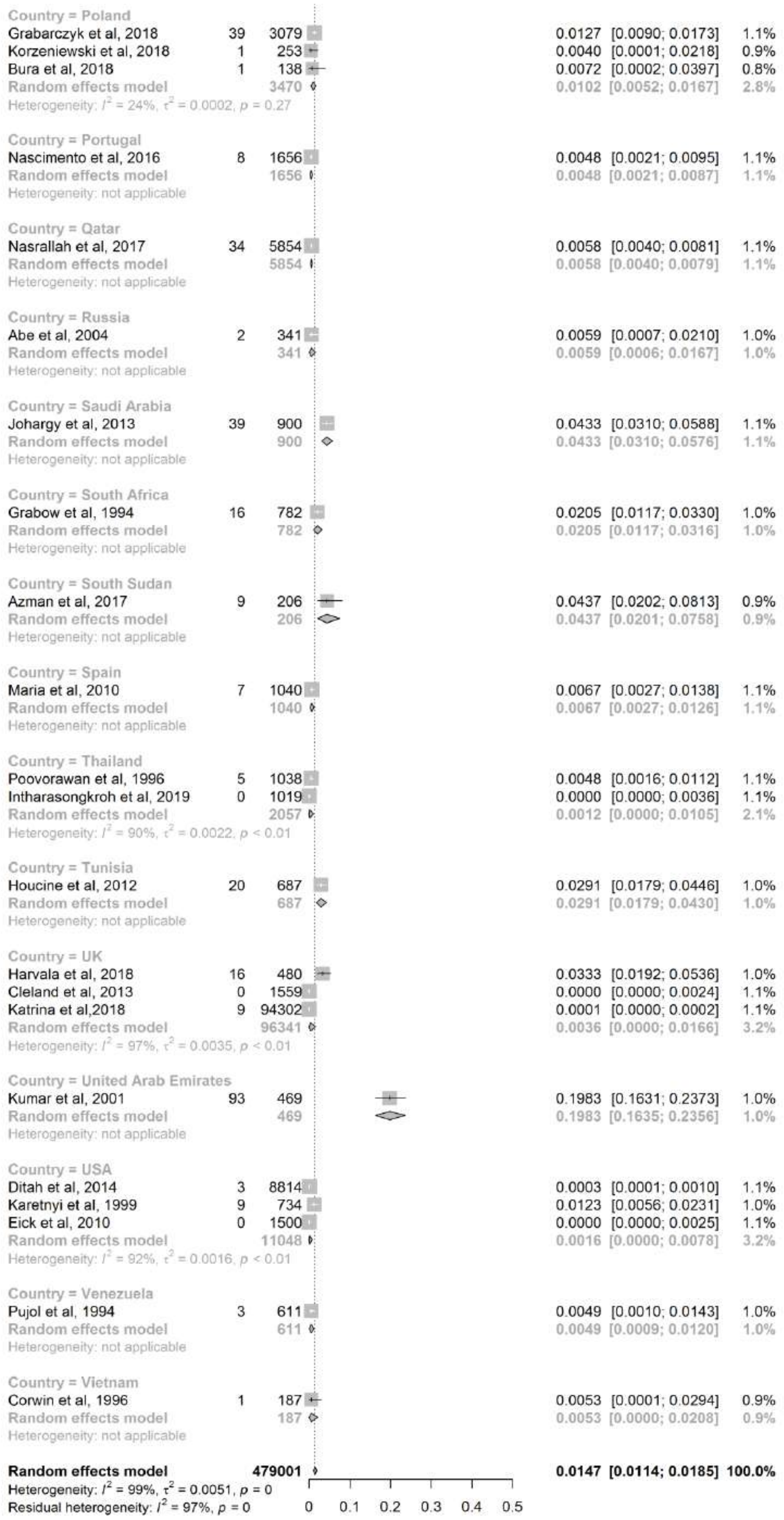

Country = Poland
Grabarczyk et al, 2018 39 3079 0.0127 [0.0090; 0.0173] 1.1%
Korzeniewski et al, 2018 1 253 0.0040 [0.0001; 0.0218] 0.9%
Bura et al, 2018 1 138 0.0072 [0.0002; 0.0397] 0.8%
Random effects model 3470 0.0102 [0.0052; 0.0167] 2.8%
Heterogeneity: $I^2$ = 24%, $\tau^2$ = 0.0002, $p$ = 0.27
Country = Portugal
Nascimento et al, 2016 8 1656 0.0048 [0.0021; 0.0095] 1.1%
Random effects model 1656 0.0048 [0.0021; 0.0087] 1.1%
Heterogeneity: not applicable
Country = Qatar
Nasrallah et al, 2017 34 5854 0.0058 [0.0040; 0.0081] 1.1%
Random effects model 5854 0.0058 [0.0040; 0.0079] 1.1%
Heterogeneity: not applicable
Country = Russia
Abe et al, 2004 2 341 0.0059 [0.0007; 0.0210] 1.0%
Random effects model 341 0.0059 [0.0006; 0.0167] 1.0%
Heterogeneity: not applicable
Country = Saudi Arabia
Johargy et al, 2013 39 900 0.0433 [0.0310; 0.0588] 1.1%
Random effects model 900 0.0433 [0.0310; 0.0576] 1.1%
Heterogeneity: not applicable
Country = South Africa
Grabow et al, 1994 16 782 0.0205 [0.0117; 0.0330] 1.0%
Random effects model 782 0.0205 [0.0117; 0.0316] 1.0%
Heterogeneity: not applicable
Country = South Sudan
Azman et al, 2017 9 206 0.0437 [0.0202; 0.0813] 0.9%
Random effects model 206 0.0437 [0.0201; 0.0758] 0.9%
Heterogeneity: not applicable
Country = Spain
Maria et al, 2010 7 1040 0.0067 [0.0027; 0.0138] 1.1%
Random effects model 1040 0.0067 [0.0027; 0.0126] 1.1%
Heterogeneity: not applicable
Country = Thailand
Poovorawan et al, 1996 5 1038 0.0048 [0.0016; 0.0112] 1.1%
Intharasongkroh et al, 2019 0 1019 0.0000 [0.0000; 0.0036] 1.1%
Random effects model 2057 0.0012 [0.0000; 0.0105] 2.1%
Heterogeneity: $I^2$ = 90%, $\tau^2$ = 0.0022, $p$ < 0.01
Country = Tunisia
Houcine et al, 2012 20 687 0.0291 [0.0179; 0.0446] 1.0%
Random effects model 687 0.0291 [0.0179; 0.0430] 1.0%
Heterogeneity: not applicable
Country = UK
Harvala et al, 2018 16 480 0.0333 [0.0192; 0.0536] 1.0%
Cleland et al, 2013 0 1559 0.0000 [0.0000; 0.0024] 1.1%
Katrina et al,2018 9 94302 0.0001 [0.0000; 0.0002] 1.1%
Random effects model 96341 0.0036 [0.0000; 0.0166] 3.2%
Heterogeneity: $I^2$ = 97%, $\tau^2$ = 0.0035, $p$ < 0.01
Country = United Arab Emirates
Kumar et al, 2001 93 469 0.1983 [0.1631; 0.2373] 1.0%
Random effects model 469 0.1983 [0.1635; 0.2356] 1.0%
Heterogeneity: not applicable
Country = USA
Ditah et al, 2014 3 8814 0.0003 [0.0001; 0.0010] 1.1%
Karetnyi et al, 1999 9 734 0.0123 [0.0056; 0.0231] 1.0%
Eick et al, 2010 0 1500 0.0000 [0.0000; 0.0025] 1.1%
Random effects model 11048 0.0016 [0.0000; 0.0078] 3.2%
Heterogeneity: $I^2$ = 92%, $\tau^2$ = 0.0016, $p$ < 0.01
Country = Venezuela
Pujol et al, 1994 3 611 0.0049 [0.0010; 0.0143] 1.0%
Random effects model 611 0.0049 [0.0009; 0.0120] 1.0%
Heterogeneity: not applicable
Country = Vietnam
Corwin et al, 1996 1 187 0.0053 [0.0001; 0.0294] 0.9%
Random effects model 187 0.0053 [0.0000; 0.0208] 0.9%
Heterogeneity: not applicable
Random effects model 479001 0.0147 [0.0114; 0.0185] 100.0%
Heterogeneity: $I^2$ = 99%, $\tau^2$ = 0.0051, $p$ = 0
Residual heterogeneity: $I^2$ = 97%, $p$ = 0
0 0.1 0.2 0.3 0.4 0.5

**Figure S3. Forest plot of estimated pooled HEV RNA positive rate among general population based on different countries.**

Note: country named multiple means people in the study are from more than one country.

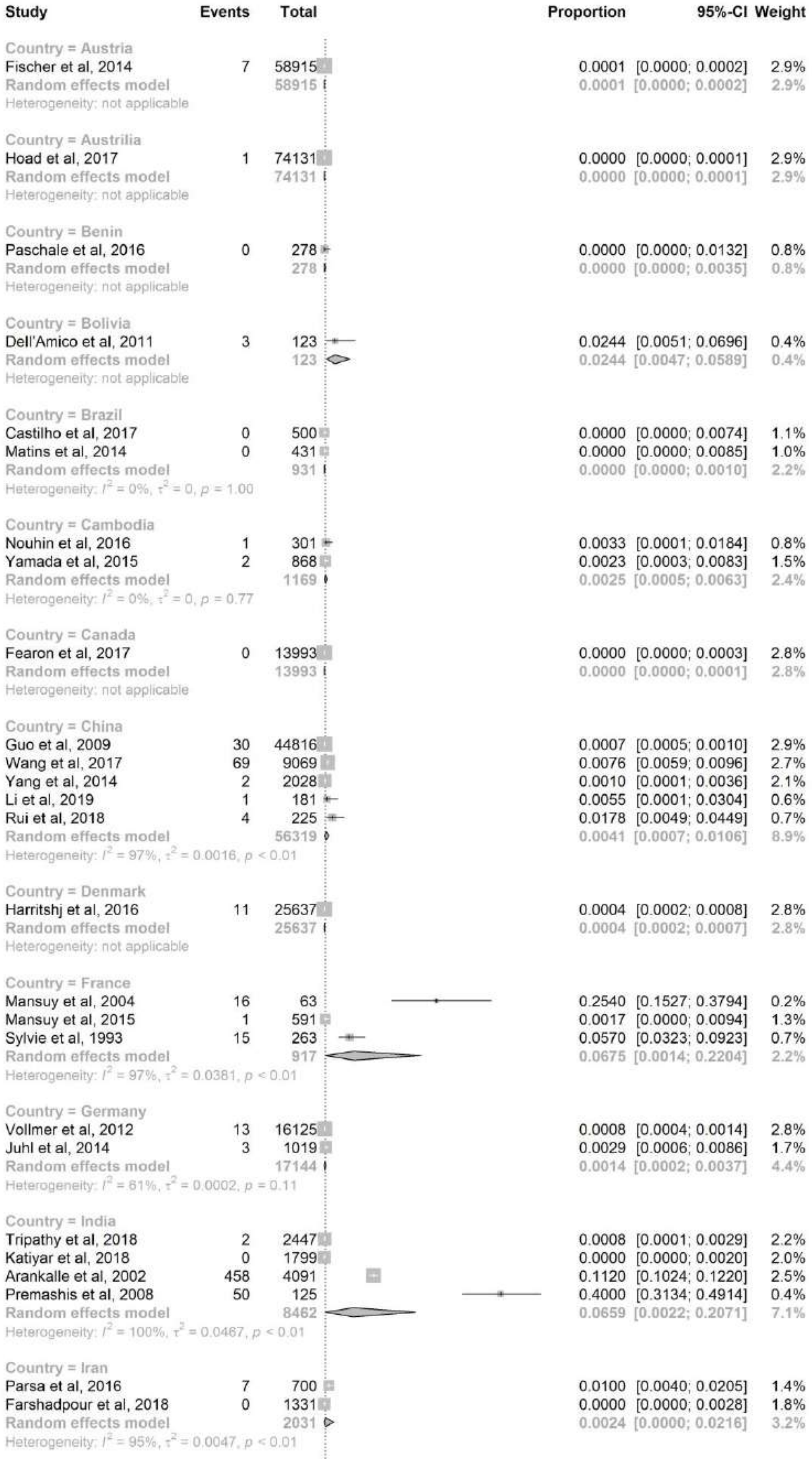

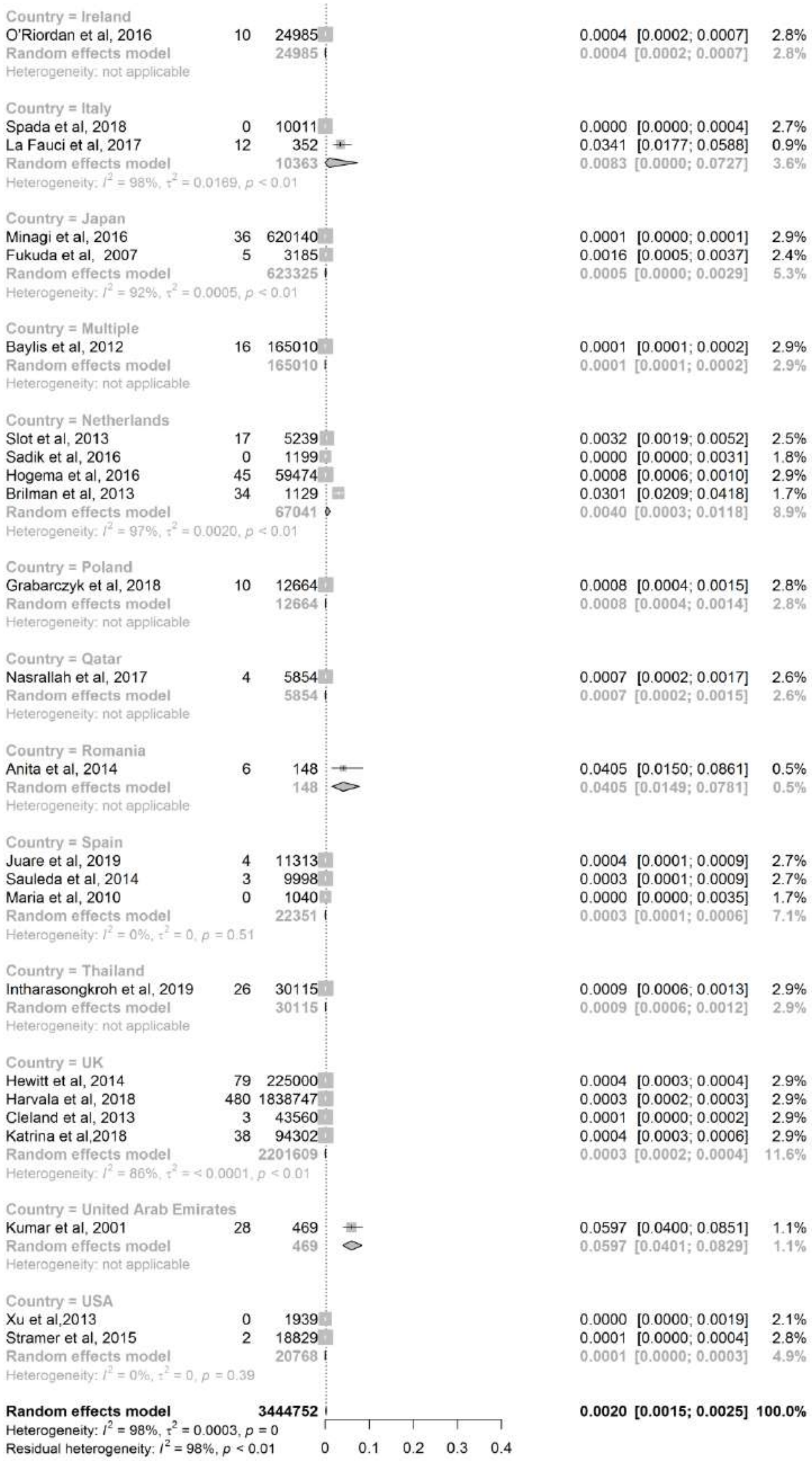
Country = Ireland
O'Riordan et al, 2016 10 24985 0.0004 [0.0002; 0.0007] 2.8%
Random effects model 24985 0.0004 [0.0002; 0.0007] 2.8%
Heterogeneity: not applicable
Country = Italy
Spada et al, 2018 0 10011 0.0000 [0.0000; 0.0004] 2.7%
La Fauci et al, 2017 12 352 0.0341 [0.0177; 0.0588] 0.9%
Random effects model 10363 0.0083 [0.0000; 0.0727] 3.6%
Heterogeneity: $I^2$ = 98%, $\tau^2$ = 0.0169, $p$ < 0.01
Country = Japan
Minagi et al, 2016 36 620140 0.0001 [0.0000; 0.0001] 2.9%
Fukuda et al, 2007 5 3185 0.0016 [0.0005; 0.0037] 2.4%
Random effects model 623325 0.0005 [0.0000; 0.0029] 5.3%
Heterogeneity: $I^2$ = 92%, $\tau^2$ = 0.0005, $p$ < 0.01
Country = Multiple
Baylis et al, 2012 16 165010 0.0001 [0.0001; 0.0002] 2.9%
Random effects model 165010 0.0001 [0.0001; 0.0002] 2.9%
Heterogeneity: not applicable
Country = Netherlands
Slot et al, 2013 17 5239 0.0032 [0.0019; 0.0052] 2.5%
Sadik et al, 2016 0 1199 0.0000 [0.0000; 0.0031] 1.8%
Hogema et al, 2016 45 59474 0.0008 [0.0006; 0.0010] 2.9%
Brilman et al, 2013 34 1129 0.0301 [0.0209; 0.0418] 1.7%
Random effects model 67041 0.0040 [0.0003; 0.0118] 8.9%
Heterogeneity: $I^2$ = 97%, $\tau^2$ = 0.0020, $p$ < 0.01
Country = Poland
Grabarczyk et al, 2018 10 12664 0.0008 [0.0004; 0.0015] 2.8%
Random effects model 12664 0.0008 [0.0004; 0.0014] 2.8%
Heterogeneity: not applicable
Country = Qatar
Nasrallah et al, 2017 4 5854 0.0007 [0.0002; 0.0017] 2.6%
Random effects model 5854 0.0007 [0.0002; 0.0015] 2.6%
Heterogeneity: not applicable
Country = Romania
Anita et al, 2014 6 148 0.0405 [0.0150; 0.0861] 0.5%
Random effects model 148 0.0405 [0.0149; 0.0781] 0.5%
Heterogeneity: not applicable
Country = Spain
Juare et al, 2019 4 11313 0.0004 [0.0001; 0.0009] 2.7%
Sauleda et al, 2014 3 9998 0.0003 [0.0001; 0.0009] 2.7%
Maria et al, 2010 0 1040 0.0000 [0.0000; 0.0035] 1.7%
Random effects model 22351 0.0003 [0.0001; 0.0006] 7.1%
Heterogeneity: $I^2$ = 0%, $\tau^2$ = 0, $p$ = 0.51
Country = Thailand
Intharasongkroh et al, 2019 26 30115 0.0009 [0.0006; 0.0013] 2.9%
Random effects model 30115 0.0009 [0.0006; 0.0012] 2.9%
Heterogeneity: not applicable
Country = UK
Hewitt et al, 2014 79 225000 0.0004 [0.0003; 0.0004] 2.9%
Harvala et al, 2018 480 1838747 0.0003 [0.0002; 0.0003] 2.9%
Cleland et al, 2013 3 43560 0.0001 [0.0000; 0.0002] 2.9%
Katrina et al,2018 38 94302 0.0004 [0.0003; 0.0006] 2.9%
Random effects model 2201609 0.0003 [0.0002; 0.0004] 11.6%
Heterogeneity: $I^2$ = 86%, $\tau^2$ = < 0.0001, $p$ < 0.01
Country = United Arab Emirates
Kumar et al, 2001 28 469 0.0597 [0.0400; 0.0851] 1.1%
Random effects model 469 0.0597 [0.0401; 0.0829] 1.1%
Heterogeneity: not applicable
Country = USA
Xu et al,2013 0 1939 0.0000 [0.0000; 0.0019] 2.1%
Stramer et al, 2015 2 18829 0.0001 [0.0000; 0.0004] 2.8%
Random effects model 20768 0.0001 [0.0000; 0.0003] 4.9%
Heterogeneity: $I^2$ = 0%, $\tau^2$ = 0, $p$ = 0.39
Random effects model 3444752 0.0020 [0.0015; 0.0025] 100.0%
Heterogeneity: $I^2$ = 98%, $\tau^2$ = 0.0003, $p$ = 0
Residual heterogeneity: $I^2$ = 98%, $p$ < 0.01
0 0.1 0.2 0.3 0.4

**Figure S4. Forest plot of estimated anti-HEV IgG seroprevalence among general population based on different continents.**

Note: continent named multiple means people in the study are from more than one country and cannot be included into one continent.

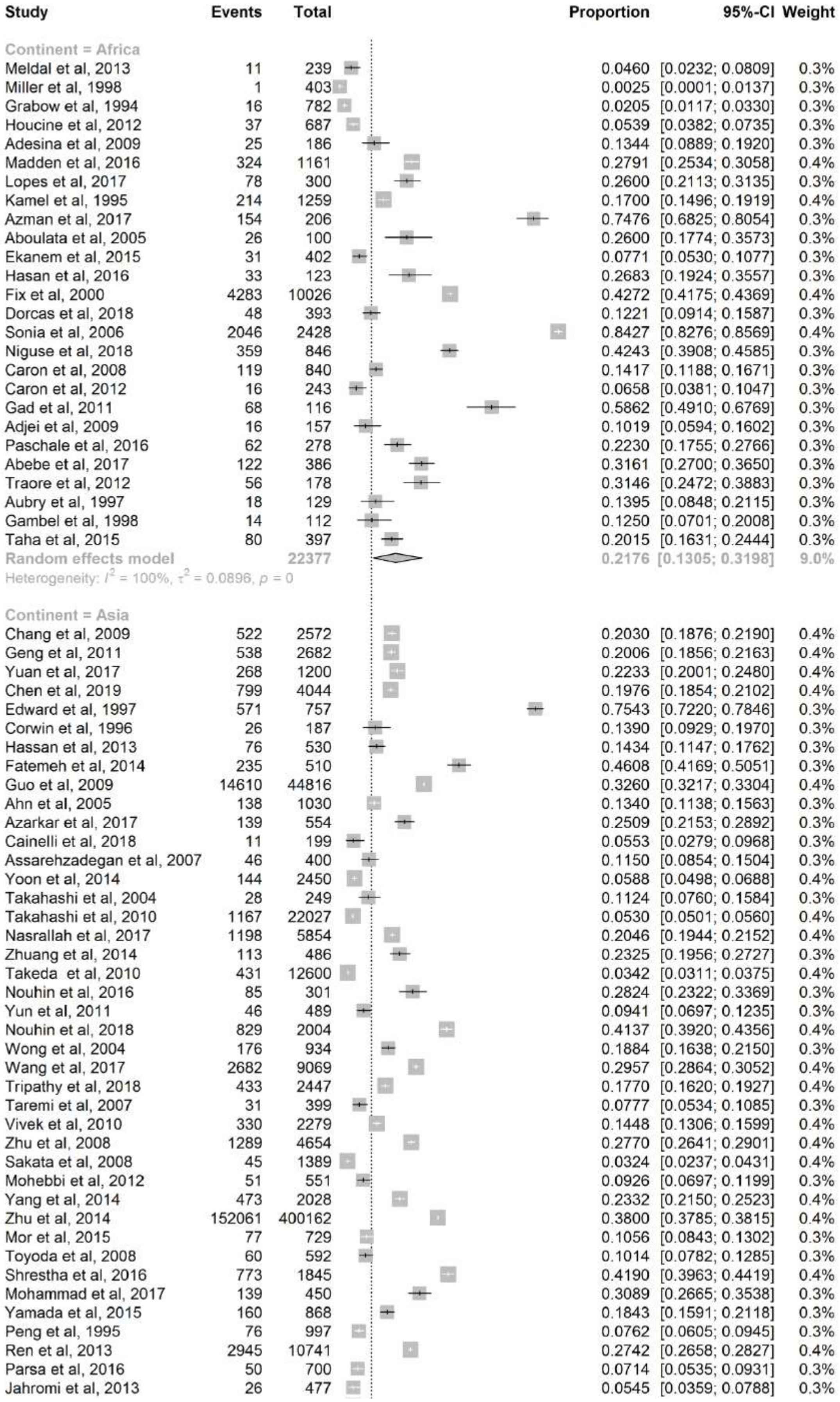

| | | | 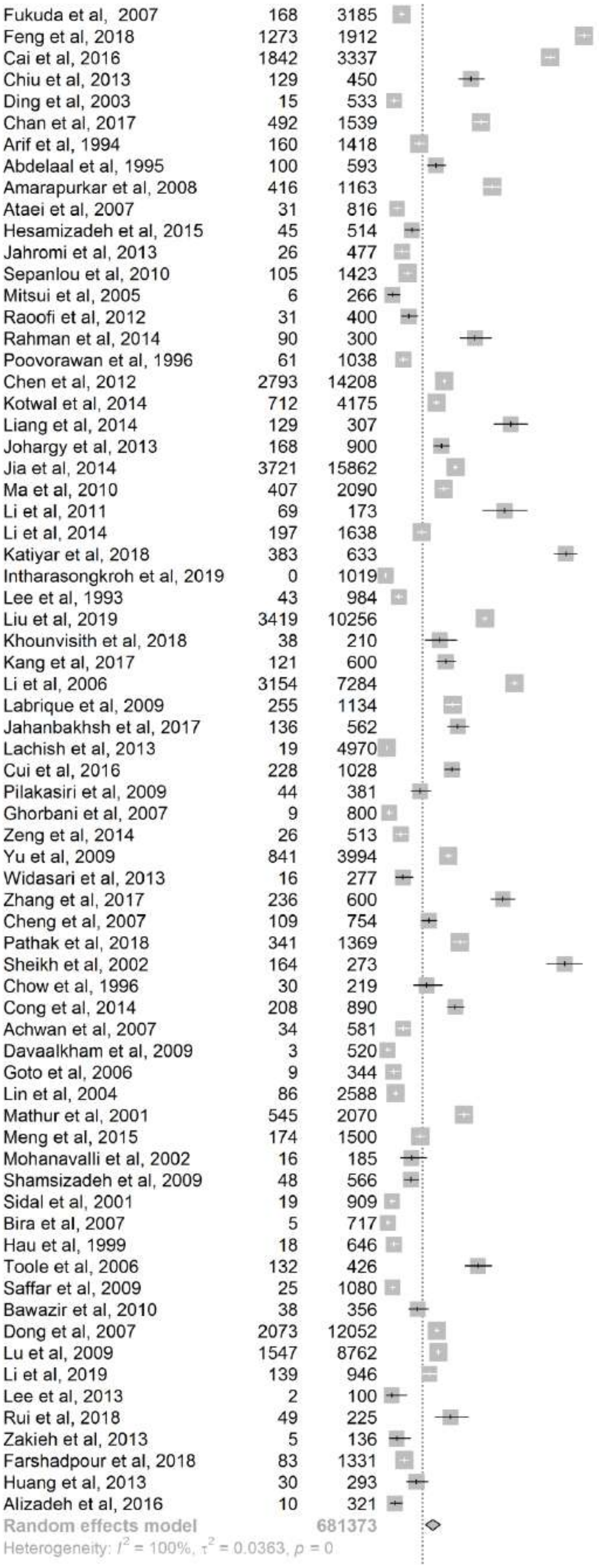 | | | |
|---|---|---|---|---|---|---|
| Fukuda et al, 2007 | 168 | 3185 | | 0.0527 | [0.0452; 0.0611] | 0.4% |
| Feng et al, 2018 | 1273 | 1912 | | 0.6658 | [0.6441; 0.6869] | 0.4% |
| Cai et al, 2016 | 1842 | 3337 | | 0.5520 | [0.5349; 0.5690] | 0.4% |
| Chiu et al, 2013 | 129 | 450 | | 0.2867 | [0.2453; 0.3309] | 0.3% |
| Ding et al, 2003 | 15 | 533 | | 0.0281 | [0.0158; 0.0460] | 0.3% |
| Chan et al, 2017 | 492 | 1539 | | 0.3197 | [0.2964; 0.3436] | 0.4% |
| Arif et al, 1994 | 160 | 1418 | | 0.1128 | [0.0968; 0.1305] | 0.4% |
| Abdelaal et al, 1995 | 100 | 593 | | 0.1686 | [0.1394; 0.2012] | 0.3% |
| Amarapurkar et al, 2008 | 416 | 1163 | | 0.3577 | [0.3301; 0.3860] | 0.4% |
| Ataei et al, 2007 | 31 | 816 | | 0.0380 | [0.0260; 0.0535] | 0.3% |
| Hesamizadeh et al, 2015 | 45 | 514 | | 0.0875 | [0.0646; 0.1154] | 0.3% |
| Jahromi et al, 2013 | 26 | 477 | | 0.0545 | [0.0359; 0.0788] | 0.3% |
| Sepanlou et al, 2010 | 105 | 1423 | | 0.0738 | [0.0607; 0.0886] | 0.4% |
| Mitsui et al, 2005 | 6 | 266 | | 0.0226 | [0.0083; 0.0484] | 0.3% |
| Raoofi et al, 2012 | 31 | 400 | | 0.0775 | [0.0533; 0.1082] | 0.3% |
| Rahman et al, 2014 | 90 | 300 | | 0.3000 | [0.2487; 0.3553] | 0.3% |
| Poovorawan et al, 1996 | 61 | 1038 | | 0.0588 | [0.0452; 0.0749] | 0.3% |
| Chen et al, 2012 | 2793 | 14208 | | 0.1966 | [0.1901; 0.2032] | 0.4% |
| Kotwal et al, 2014 | 712 | 4175 | | 0.1705 | [0.1592; 0.1823] | 0.4% |
| Liang et al, 2014 | 129 | 307 | | 0.4202 | [0.3644; 0.4776] | 0.3% |
| Johargy et al, 2013 | 168 | 900 | | 0.1867 | [0.1617; 0.2137] | 0.3% |
| Jia et al, 2014 | 3721 | 15862 | | 0.2346 | [0.2280; 0.2413] | 0.4% |
| Ma et al, 2010 | 407 | 2090 | | 0.1947 | [0.1780; 0.2124] | 0.4% |
| Li et al, 2011 | 69 | 173 | | 0.3988 | [0.3253; 0.4759] | 0.3% |
| Li et al, 2014 | 197 | 1638 | | 0.1203 | [0.1049; 0.1370] | 0.4% |
| Katiyar et al, 2018 | 383 | 633 | | 0.6051 | [0.5658; 0.6434] | 0.3% |
| Intharasongkroh et al, 2019 | 0 | 1019 | | 0.0000 | [0.0000; 0.0036] | 0.3% |
| Lee et al, 1993 | 43 | 984 | | 0.0437 | [0.0318; 0.0584] | 0.3% |
| Liu et al, 2019 | 3419 | 10256 | | 0.3334 | [0.3242; 0.3426] | 0.4% |
| Khounvisith et al, 2018 | 38 | 210 | | 0.1810 | [0.1313; 0.2398] | 0.3% |
| Kang et al, 2017 | 121 | 600 | | 0.2017 | [0.1703; 0.2360] | 0.3% |
| Li et al, 2006 | 3154 | 7284 | | 0.4330 | [0.4216; 0.4445] | 0.4% |
| Labrique et al, 2009 | 255 | 1134 | | 0.2249 | [0.2009; 0.2503] | 0.4% |
| Jahanbakhsh et al, 2017 | 136 | 562 | | 0.2420 | [0.2071; 0.2796] | 0.3% |
| Lachish et al, 2013 | 19 | 4970 | | 0.0038 | [0.0023; 0.0060] | 0.4% |
| Cui et al, 2016 | 228 | 1028 | | 0.2218 | [0.1967; 0.2485] | 0.3% |
| Pilakasiri et al, 2009 | 44 | 381 | | 0.1155 | [0.0852; 0.1519] | 0.3% |
| Ghorbani et al, 2007 | 9 | 800 | | 0.0112 | [0.0052; 0.0212] | 0.3% |
| Zeng et al, 2014 | 26 | 513 | | 0.0507 | [0.0334; 0.0734] | 0.3% |
| Yu et al, 2009 | 841 | 3994 | | 0.2106 | [0.1980; 0.2235] | 0.4% |
| Widasari et al, 2013 | 16 | 277 | | 0.0578 | [0.0334; 0.0921] | 0.3% |
| Zhang et al, 2017 | 236 | 600 | | 0.3933 | [0.3540; 0.4337] | 0.3% |
| Cheng et al, 2007 | 109 | 754 | | 0.1446 | [0.1202; 0.1717] | 0.3% |
| Pathak et al, 2018 | 341 | 1369 | | 0.2491 | [0.2264; 0.2729] | 0.4% |
| Sheikh et al, 2002 | 164 | 273 | | 0.6007 | [0.5400; 0.6593] | 0.3% |
| Chow et al, 1996 | 30 | 219 | | 0.1370 | [0.0944; 0.1897] | 0.3% |
| Cong et al, 2014 | 208 | 890 | | 0.2337 | [0.2063; 0.2629] | 0.3% |
| Achwan et al, 2007 | 34 | 581 | | 0.0585 | [0.0409; 0.0808] | 0.3% |
| Davaalkham et al, 2009 | 3 | 520 | | 0.0058 | [0.0012; 0.0168] | 0.3% |
| Goto et al, 2006 | 9 | 344 | | 0.0262 | [0.0120; 0.0491] | 0.3% |
| Lin et al, 2004 | 86 | 2588 | | 0.0332 | [0.0267; 0.0409] | 0.4% |
| Mathur et al, 2001 | 545 | 2070 | | 0.2633 | [0.2444; 0.2828] | 0.4% |
| Meng et al, 2015 | 174 | 1500 | | 0.1160 | [0.1002; 0.1333] | 0.4% |
| Mohanavalli et al, 2002 | 16 | 185 | | 0.0865 | [0.0502; 0.1366] | 0.3% |
| Shamsizadeh et al, 2009 | 48 | 566 | | 0.0848 | [0.0632; 0.1109] | 0.3% |
| Sidal et al, 2001 | 19 | 909 | | 0.0209 | [0.0126; 0.0324] | 0.3% |
| Bira et al, 2007 | 5 | 717 | | 0.0070 | [0.0023; 0.0162] | 0.3% |
| Hau et al, 1999 | 18 | 646 | | 0.0279 | [0.0166; 0.0437] | 0.3% |
| Toole et al, 2006 | 132 | 426 | | 0.3099 | [0.2662; 0.3562] | 0.3% |
| Saffar et al, 2009 | 25 | 1080 | | 0.0231 | [0.0150; 0.0340] | 0.3% |
| Bawazir et al, 2010 | 38 | 356 | | 0.1067 | [0.0767; 0.1436] | 0.3% |
| Dong et al, 2007 | 2073 | 12052 | | 0.1720 | [0.1653; 0.1789] | 0.4% |
| Lu et al, 2009 | 1547 | 8762 | | 0.1766 | [0.1686; 0.1847] | 0.4% |
| Li et al, 2019 | 139 | 946 | | 0.1469 | [0.1250; 0.1711] | 0.3% |
| Lee et al, 2013 | 2 | 100 | | 0.0200 | [0.0024; 0.0704] | 0.3% |
| Rui et al, 2018 | 49 | 225 | | 0.2178 | [0.1657; 0.2775] | 0.3% |
| Zakieh et al, 2013 | 5 | 136 | | 0.0368 | [0.0120; 0.0837] | 0.3% |
| Farshadpour et al, 2018 | 83 | 1331 | | 0.0624 | [0.0500; 0.0767] | 0.4% |
| Huang et al, 2013 | 30 | 293 | | 0.1024 | [0.0702; 0.1429] | 0.3% |
| Alizadeh et al, 2016 | 10 | 321 | | 0.0312 | [0.0150; 0.0565] | 0.3% |
| Random effects model | | 681373 | | 0.1580 | [0.1329; 0.1849] | 38.8% |
| Heterogeneity: $I^2 = 100\%$, $\tau^2 = 0.0363$, $p = 0$ | | | | | | |

Continent = Europe

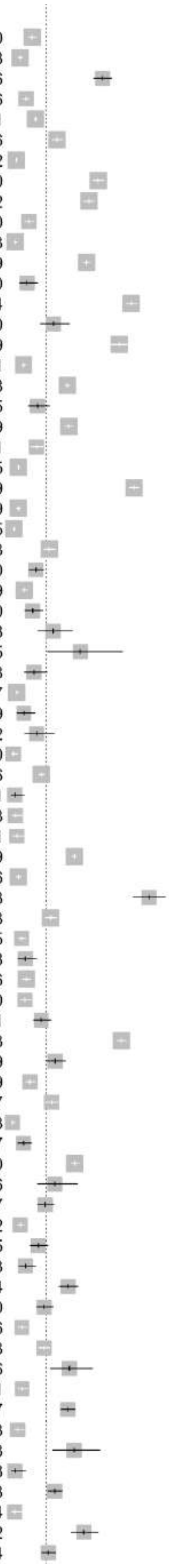

| | | | | | |
|---|---|---|---|---|---|
| Maria et al, 2006 | 96 | 1280 | 0.0750 | [0.0612; 0.0908] | 0.4% |
| Boutrouille et al, 2007 | 64 | 1998 | 0.0320 | [0.0248; 0.0407] | 0.4% |
| Christensen et al,2018 | 293 | 906 | 0.3234 | [0.2930; 0.3549] | 0.3% |
| O'Riordan et al, 2016 | 57 | 1076 | 0.0530 | [0.0404; 0.0681] | 0.3% |
| Spada et al, 2018 | 869 | 10011 | 0.0868 | [0.0814; 0.0925] | 0.4% |
| Nascimento et al, 2016 | 270 | 1656 | 0.1630 | [0.1456; 0.1817] | 0.4% |
| Verhoef et al, 2011 | 134 | 7072 | 0.0189 | [0.0159; 0.0224] | 0.4% |
| Mooij et al, 2018 | 648 | 2100 | 0.3086 | [0.2889; 0.3288] | 0.4% |
| Lafeber et al, 2017 | 666 | 2422 | 0.2750 | [0.2573; 0.2932] | 0.4% |
| Olcay et al, 2003 | 57 | 910 | 0.0626 | [0.0478; 0.0804] | 0.3% |
| Rapicetta et al, 1999 | 33 | 2233 | 0.0148 | [0.0102; 0.0207] | 0.4% |
| Slot et al, 2013 | 1401 | 5239 | 0.2674 | [0.2555; 0.2796] | 0.4% |
| Straková et al, 2016 | 13 | 230 | 0.0565 | [0.0304; 0.0947] | 0.3% |
| Wenzel et al, 2014 | 929 | 2184 | 0.4254 | [0.4045; 0.4464] | 0.4% |
| Petrovi et al, 2014 | 30 | 200 | 0.1500 | [0.1035; 0.2072] | 0.3% |
| Sadik et al, 2016 | 459 | 1199 | 0.3828 | [0.3552; 0.4110] | 0.4% |
| Thom et al, 2017 | 104 | 2311 | 0.0450 | [0.0369; 0.0543] | 0.4% |
| Sauleda et al, 2014 | 1996 | 9998 | 0.1996 | [0.1918; 0.2076] | 0.4% |
| Pittaras et al, 2014 | 25 | 265 | 0.0943 | [0.0620; 0.1361] | 0.3% |
| Niederhauser et al, 2017 | 737 | 3609 | 0.2042 | [0.1912; 0.2177] | 0.4% |
| Teoharov et al, 2014 | 67 | 741 | 0.0904 | [0.0708; 0.1134] | 0.3% |
| Alberts et al, 2018 | 190 | 7065 | 0.0269 | [0.0232; 0.0309] | 0.4% |
| Grabarczyk et al, 2018 | 1340 | 3079 | 0.4352 | [0.4176; 0.4529] | 0.4% |
| Gessoni et al, 1996 | 49 | 1889 | 0.0259 | [0.0193; 0.0341] | 0.4% |
| Fogeda et al, 2012 | 25 | 2305 | 0.0108 | [0.0070; 0.0160] | 0.4% |
| Fischer et al, 2014 | 163 | 1203 | 0.1355 | [0.1166; 0.1561] | 0.4% |
| Harvala et al, 2018 | 42 | 480 | 0.0875 | [0.0638; 0.1164] | 0.3% |
| Cleland et al, 2013 | 73 | 1559 | 0.0468 | [0.0369; 0.0585] | 0.4% |
| Dimeglio et al, 2018 | 23 | 300 | 0.0767 | [0.0492; 0.1128] | 0.3% |
| Anita et al, 2014 | 22 | 148 | 0.1486 | [0.0956; 0.2164] | 0.3% |
| Hogema et al, 2016 | 11 | 45 | 0.2444 | [0.1288; 0.3954] | 0.3% |
| Hickey et al, 2016 | 16 | 198 | 0.0808 | [0.0469; 0.1279] | 0.3% |
| Bernal et al, 1995 | 36 | 1757 | 0.0205 | [0.0144; 0.0283] | 0.4% |
| Ricco et al, 2016 | 9 | 199 | 0.0452 | [0.0209; 0.0841] | 0.3% |
| Puttini et al, 2015 | 12 | 132 | 0.0909 | [0.0479; 0.1534] | 0.3% |
| Pavia et al, 1998 | 3 | 360 | 0.0083 | [0.0017; 0.0242] | 0.3% |
| Zuin et al, 2016 | 380 | 3546 | 0.1072 | [0.0972; 0.1178] | 0.4% |
| Scotto et al, 2012 | 2 | 151 | 0.0132 | [0.0016; 0.0470] | 0.3% |
| Sabato et al, 2017 | 4 | 253 | 0.0158 | [0.0043; 0.0400] | 0.3% |
| Love et al, 2018 | 6 | 291 | 0.0206 | [0.0076; 0.0443] | 0.3% |
| Mansuy et al, 2016 | 2371 | 10569 | 0.2243 | [0.2164; 0.2324] | 0.4% |
| Karetny et al, 1995 | 37 | 1416 | 0.0261 | [0.0185; 0.0358] | 0.4% |
| Lucarelli et al, 2016 | 153 | 313 | 0.4888 | [0.4322; 0.5457] | 0.3% |
| Lange et al, 2016 | 177 | 1263 | 0.1401 | [0.1215; 0.1605] | 0.4% |
| Mateos et al, 1998 | 34 | 905 | 0.0376 | [0.0262; 0.0521] | 0.3% |
| Marcantonio et al, 2018 | 10 | 198 | 0.0505 | [0.0245; 0.0909] | 0.3% |
| Lanini et al, 2015 | 60 | 1116 | 0.0538 | [0.0413; 0.0687] | 0.4% |
| Kaufmann et al, 2011 | 27 | 550 | 0.0491 | [0.0326; 0.0706] | 0.3% |
| Mansuy et al, 2004 | 46 | 431 | 0.1067 | [0.0792; 0.1398] | 0.3% |
| Mansuy et al, 2015 | 1310 | 3353 | 0.3907 | [0.3741; 0.4074] | 0.4% |
| Juhl et al, 2017 | 70 | 449 | 0.1559 | [0.1236; 0.1928] | 0.3% |
| Juhl et al, 2014 | 69 | 1019 | 0.0677 | [0.0531; 0.0849] | 0.3% |
| Nagler et al, 2014 | 143 | 997 | 0.1434 | [0.1222; 0.1667] | 0.3% |
| Langer et al, 1997 | 3 | 553 | 0.0054 | [0.0011; 0.0158] | 0.3% |
| Lavanchy et al, 1994 | 14 | 317 | 0.0442 | [0.0244; 0.0730] | 0.3% |
| Mahrt et al, 2018 | 679 | 3000 | 0.2263 | [0.2115; 0.2417] | 0.4% |
| Krumbholz et al, 2011 | 18 | 116 | 0.1552 | [0.0946; 0.2341] | 0.3% |
| Krumbholz et al, 2014 | 65 | 537 | 0.1210 | [0.0947; 0.1517] | 0.3% |
| Jeggli et al, 2015 | 25 | 752 | 0.0332 | [0.0216; 0.0487] | 0.3% |
| Kantala et al, 2015 | 37 | 385 | 0.0961 | [0.0686; 0.1300] | 0.3% |
| Korzeniewski et al, 2018 | 13 | 253 | 0.0514 | [0.0276; 0.0863] | 0.3% |
| Jablonka et al, 2017 | 122 | 604 | 0.2020 | [0.1707; 0.2363] | 0.3% |
| Kaba et al, 2010 | 57 | 490 | 0.1163 | [0.0893; 0.1481] | 0.3% |
| Chironna et al, 1999 | 20 | 526 | 0.0380 | [0.0234; 0.0581] | 0.3% |
| Rapicetta et al,1999 | 113 | 973 | 0.1161 | [0.0967; 0.1380] | 0.3% |
| Schielke et al, 2015 | 26 | 126 | 0.2063 | [0.1394; 0.2875] | 0.3% |
| Nübling et al, 2002 | 20 | 511 | 0.0391 | [0.0241; 0.0598] | 0.3% |
| Todys et al, 2015 | 206 | 1027 | 0.2006 | [0.1765; 0.2264] | 0.3% |
| Meader et al, 2008 | 10 | 413 | 0.0242 | [0.0117; 0.0441] | 0.3% |
| Tabili et al, 2013 | 23 | 103 | 0.2233 | [0.1471; 0.3160] | 0.3% |
| Bura et al, 2018 | 2 | 138 | 0.0145 | [0.0018; 0.0514] | 0.3% |
| Dremsek et al, 2012 | 132 | 853 | 0.1547 | [0.1311; 0.1808] | 0.3% |
| Alecci et al, 1997 | 3 | 244 | 0.0123 | [0.0025; 0.0355] | 0.3% |
| Chaussade et al, 2013 | 83 | 322 | 0.2578 | [0.2109; 0.3092] | 0.3% |
| Teixeira et al, 2017 | 106 | 804 | 0.1318 | [0.1092; 0.1572] | 0.3% |

| Study | Events | Total | Proportion | 95% CI | Weight |
|---|---|---|---|---|---|
| Olsen et al, 2006 | 10 | 108 | 0.0926 | [0.0453; 0.1637] | 0.3% |
| Bouwknegt et al, 2008 | 12 | 644 | 0.0186 | [0.0097; 0.0323] | 0.3% |
| Drobeniuc et al, 2001 | 63 | 255 | 0.2471 | [0.1954; 0.3047] | 0.3% |
| Galiana et al, 2008 | 4 | 97 | 0.0412 | [0.0113; 0.1022] | 0.3% |
| Dalton et al, 2017 | 11 | 464 | 0.0237 | [0.0119; 0.0420] | 0.3% |
| Colak et al, 2002 | 3 | 338 | 0.0089 | [0.0018; 0.0257] | 0.3% |
| Terzic et al, 2009 | 24 | 400 | 0.0600 | [0.0388; 0.0880] | 0.3% |
| Bayhan et al, 2016 | 17 | 408 | 0.0417 | [0.0245; 0.0659] | 0.3% |
| Abe et al, 2004 | 62 | 341 | 0.1818 | [0.1423; 0.2269] | 0.3% |
| Atabek et al, 2004 | 12 | 210 | 0.0571 | [0.0299; 0.0977] | 0.3% |
| Buti et al, 2008 | 57 | 1249 | 0.0456 | [0.0347; 0.0587] | 0.4% |
| Cevahir et al, 2013 | 23 | 185 | 0.1243 | [0.0805; 0.1807] | 0.3% |
| Kaya et al, 2007 | 2 | 589 | 0.0034 | [0.0004; 0.0122] | 0.3% |
| Krumbholz et al, 2014 | 16 | 1646 | 0.0097 | [0.0056; 0.0157] | 0.4% |
| Oliveira et al, 2017 | 4 | 352 | 0.0114 | [0.0031; 0.0288] | 0.3% |
| Sencan et al, 2003 | 34 | 476 | 0.0714 | [0.0500; 0.0984] | 0.3% |
| Ijaz et al, 2009 | 362 | 2731 | 0.1326 | [0.1201; 0.1458] | 0.4% |
| Serkan et al, 2005 | 27 | 386 | 0.0699 | [0.0466; 0.1001] | 0.3% |
| Christophe et al, 2004 | 24 | 315 | 0.0762 | [0.0494; 0.1112] | 0.3% |
| Maria et al, 2010 | 38 | 1040 | 0.0365 | [0.0260; 0.0498] | 0.3% |
| Cevrioglu et al, 2004 | 31 | 245 | 0.1265 | [0.0876; 0.1748] | 0.3% |
| Katrina et al, 2018 | 104 | 1714 | 0.0607 | [0.0498; 0.0730] | 0.4% |
| Random effects model | | 132419 | 0.0931 | [0.0735; 0.1148] | 33.8% |
| Heterogeneity: $I^2 = 99\%$, $\tau^2 = 0.0312$, $p = 0$ | | | | | |
| **Continent = Multiple** | | | | | |
| Halliday et al, 2014 | 120 | 1048 | 0.1145 | [0.0959; 0.1354] | 0.3% |
| Abe et al, 2006 | 1720 | 5233 | 0.3287 | [0.3160; 0.3416] | 0.4% |
| Baylis et al, 2012 | 1 | 165010 | 0.0000 | [0.0000; 0.0000] | 0.4% |
| Schreuder et al, 2016 | 25 | 600 | 0.0417 | [0.0271; 0.0609] | 0.3% |
| Izopet et al, 2015 | 1083 | 2538 | 0.4267 | [0.4074; 0.4462] | 0.4% |
| Gambel et al, 1998 | 209 | 981 | 0.2130 | [0.1878; 0.2400] | 0.3% |
| Random effects model | | 175410 | 0.1464 | [0.0040; 0.4367] | 2.1% |
| Heterogeneity: $I^2 = 100\%$, $\tau^2 = 0.1693$, $p = 0$ | | | | | |
| **Continent = North America** | | | | | |
| Minuk et al, 2007 | 11 | 393 | 0.0280 | [0.0141; 0.0495] | 0.3% |
| Quintana et al, 2005 | 11 | 209 | 0.0526 | [0.0266; 0.0922] | 0.3% |
| Villalba et al, 2014 | 47 | 469 | 0.1002 | [0.0746; 0.1310] | 0.3% |
| Fearon et al, 2017 | 241 | 4102 | 0.0588 | [0.0517; 0.0664] | 0.4% |
| Cangin et al, 2018 | 377 | 7656 | 0.0492 | [0.0445; 0.0543] | 0.4% |
| Ditah et al, 2014 | 490 | 8814 | 0.0556 | [0.0509; 0.0606] | 0.4% |
| Karetnyi et al, 1999 | 36 | 734 | 0.0490 | [0.0346; 0.0673] | 0.3% |
| Kuniholm et al, 2009 | 3916 | 18695 | 0.2095 | [0.2037; 0.2154] | 0.4% |
| Esquivel et al, 2014 | 100 | 273 | 0.3663 | [0.3090; 0.4265] | 0.3% |
| Eick et al, 2010 | 16 | 1500 | 0.0107 | [0.0061; 0.0173] | 0.4% |
| Whiters et al, 2002 | 3 | 125 | 0.0240 | [0.0050; 0.0685] | 0.3% |
| Munoz et al, 1999 | 374 | 3549 | 0.1054 | [0.0955; 0.1160] | 0.4% |
| Héctor et al, 2017 | 1 | 127 | 0.0079 | [0.0002; 0.0431] | 0.3% |
| Cosme et al, 2014 | 25 | 439 | 0.0569 | [0.0372; 0.0829] | 0.3% |
| Zhang et al, 2015 | 1352 | 6262 | 0.2159 | [0.2058; 0.2263] | 0.4% |
| Kyvernitakis et al, 2015 | 12 | 115 | 0.1043 | [0.0551; 0.1752] | 0.3% |
| Tashale et al, 2015 | 1081 | 13851 | 0.0780 | [0.0736; 0.0826] | 0.4% |
| Xu et al,2013 | 364 | 1939 | 0.1877 | [0.1706; 0.2058] | 0.4% |
| Stramer et al, 2015 | 27 | 200 | 0.1350 | [0.0909; 0.1903] | 0.3% |
| Meng et al, 2001 | 73 | 400 | 0.1825 | [0.1459; 0.2239] | 0.3% |
| Esquivel et al, 2015 | 10 | 150 | 0.0667 | [0.0324; 0.1192] | 0.3% |
| Esquivel et al, 2015 | 25 | 439 | 0.0569 | [0.0372; 0.0829] | 0.3% |
| Perez et al, 1996 | 17 | 399 | 0.0426 | [0.0250; 0.0673] | 0.3% |
| Lemos et al, 2000 | 16 | 1149 | 0.0139 | [0.0080; 0.0225] | 0.4% |
| Random effects model | | 71989 | 0.0805 | [0.0547; 0.1109] | 8.4% |
| Heterogeneity: $I^2 = 99\%$, $\tau^2 = 0.0161$, $p = 0$ | | | | | |
| **Continent = Oceania** | | | | | |
| Moaven et al, 1995 | 17 | 550 | 0.0309 | [0.0181; 0.0490] | 0.3% |
| Hewitt et al, 2018 | 98 | 1013 | 0.0967 | [0.0792; 0.1166] | 0.3% |
| Random effects model | | 1563 | 0.0599 | [0.0122; 0.1403] | 0.7% |
| Heterogeneity: $I^2 = 96\%$, $\tau^2 = 0.0094$, $p < 0.01$ | | | | | |
| **Continent = South America** | | | | | |
| Dell'Amico et al, 2011 | 12 | 123 | 0.0976 | [0.0514; 0.1642] | 0.3% |
| Castilho et al, 2017 | 49 | 500 | 0.0980 | [0.0734; 0.1275] | 0.3% |
| Castilho et al, 2016 | 30 | 300 | 0.1000 | [0.0685; 0.1397] | 0.3% |
| Vitral et al, 2014 | 50 | 388 | 0.1289 | [0.0972; 0.1663] | 0.3% |
| Talarmin et al, 1997 | 64 | 996 | 0.0643 | [0.0498; 0.0813] | 0.3% |
| Santos et al, 2002 | 17 | 699 | 0.0243 | [0.0142; 0.0387] | 0.3% |
| Bortoliero et al, 2006 | 23 | 996 | 0.0231 | [0.0147; 0.0344] | 0.3% |
| Paula et al, 2001 | 10 | 255 | 0.0392 | [0.0190; 0.0709] | 0.3% |
| Goncales et al, 2000 | 18 | 357 | 0.0504 | [0.0302; 0.0785] | 0.3% |
| Hardtke et al, 2018 | 91 | 408 | 0.2230 | [0.1836; 0.2666] | 0.3% |
| Pujol et al, 1994 | 23 | 611 | 0.0376 | [0.0240; 0.0559] | 0.3% |
| Konomi et al, 1999 | 93 | 574 | 0.1620 | [0.1328; 0.1948] | 0.3% |
| Ibarra et al, 1994 | 82 | 469 | 0.1748 | [0.1415; 0.2123] | 0.3% |
| Matins et al, 2014 | 24 | 431 | 0.0557 | [0.0360; 0.0817] | 0.3% |
| Gandolfo et al, 2003 | 4 | 435 | 0.0092 | [0.0025; 0.0234] | 0.3% |
| Rey et al, 1997 | 95 | 5196 | 0.0183 | [0.0148; 0.0223] | 0.4% |
| Wassaf et al,2014 | 19 | 433 | 0.0439 | [0.0266; 0.0677] | 0.3% |
| Munne et al, 2014 | 31 | 202 | 0.1535 | [0.1067; 0.2107] | 0.3% |
| Bartoloni et al, 1999 | 36 | 490 | 0.0735 | [0.0520; 0.1003] | 0.3% |
| Leon et al, 1999 | 64 | 318 | 0.2013 | [0.1586; 0.2496] | 0.3% |
| Trinta et al,2001 | 3 | 405 | 0.0074 | [0.0015; 0.0215] | 0.3% |
| Random effects model | | 14586 | 0.0728 | [0.0483; 0.1019] | 7.3% |
| Heterogeneity: $I^2 = 97\%$, $\tau^2 = 0.0140$, $p < 0.01$ | | | | | |
| **Random effects model** | | **1099717** | **0.1247** | **[0.1042; 0.1467]** | **100.0%** |
| Heterogeneity: $I^2 = 100\%$, $\tau^2 = 0.0769$, $p = 0$ | | | | | |
| Residual heterogeneity: $I^2 = 100\%$, $p = 0$ | | | | | |

Axis: 0 0.2 0.4 0.6 0.8

**Figure S5. Forest plot of estimated pooled anti-HEV IgM seroprevalence among general population based on different continents.**

Note: continent named multiple means people in the study are from more than one country and cannot be included into one continent.

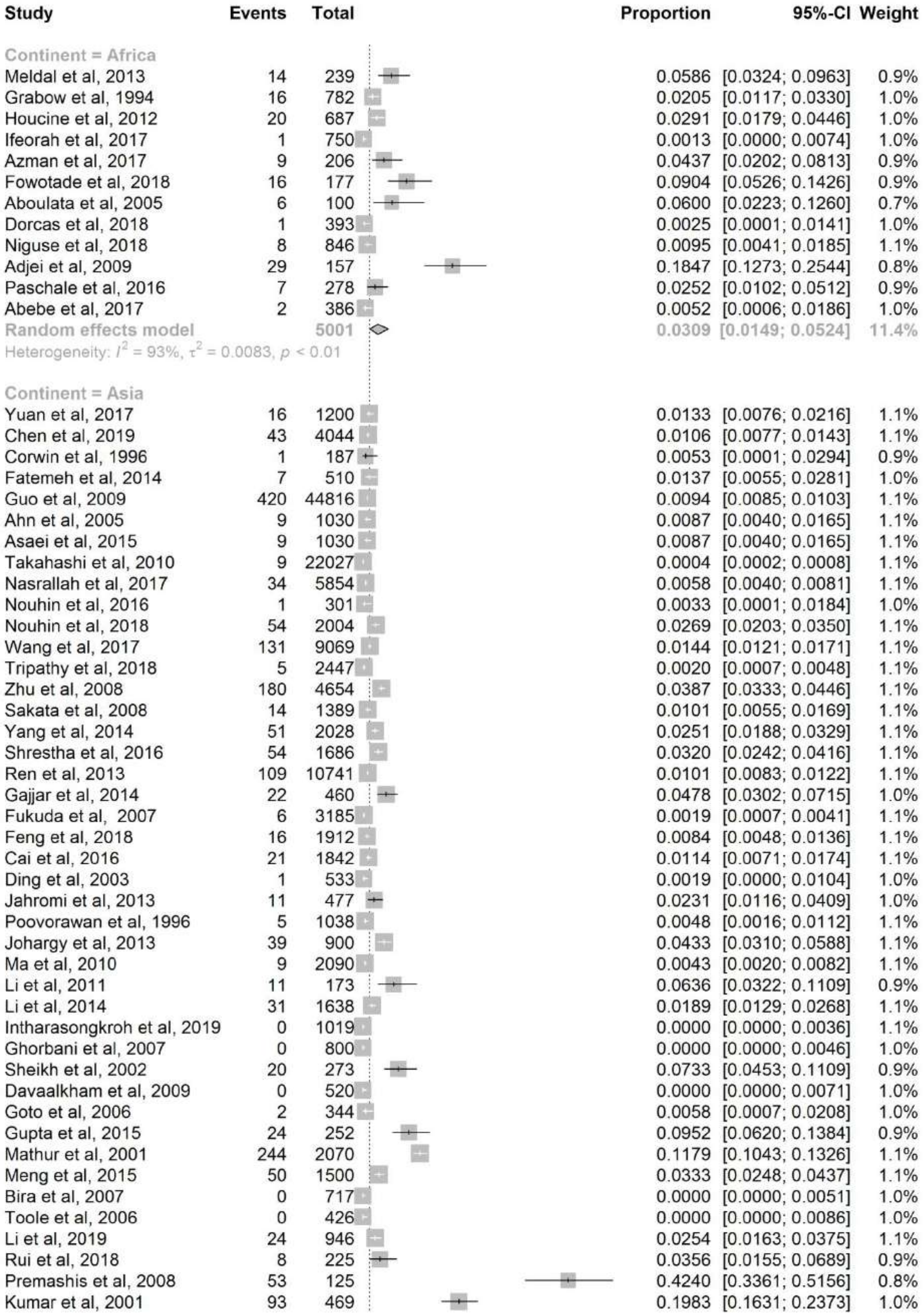

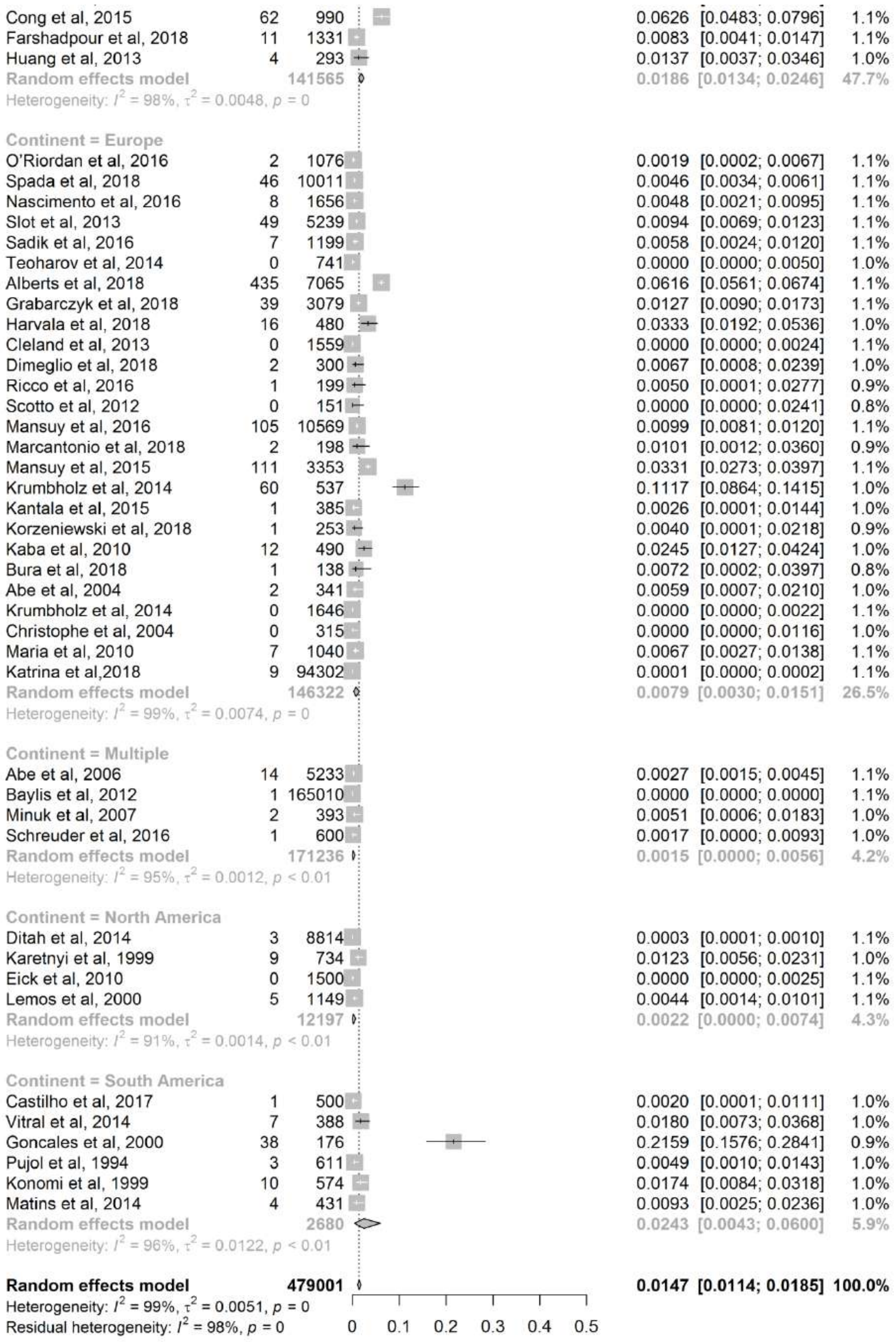
Cong et al, 2015 62 990 0.0626 [0.0483; 0.0796] 1.1%
Farshadpour et al, 2018 11 1331 0.0083 [0.0041; 0.0147] 1.1%
Huang et al, 2013 4 293 0.0137 [0.0037; 0.0346] 1.0%
Random effects model 141565 0.0186 [0.0134; 0.0246] 47.7%
Heterogeneity: $I^2$ = 98%, $\tau^2$ = 0.0048, $p$ = 0
Continent = Europe
O'Riordan et al, 2016 2 1076 0.0019 [0.0002; 0.0067] 1.1%
Spada et al, 2018 46 10011 0.0046 [0.0034; 0.0061] 1.1%
Nascimento et al, 2016 8 1656 0.0048 [0.0021; 0.0095] 1.1%
Slot et al, 2013 49 5239 0.0094 [0.0069; 0.0123] 1.1%
Sadik et al, 2016 7 1199 0.0058 [0.0024; 0.0120] 1.1%
Teoharov et al, 2014 0 741 0.0000 [0.0000; 0.0050] 1.0%
Alberts et al, 2018 435 7065 0.0616 [0.0561; 0.0674] 1.1%
Grabarczyk et al, 2018 39 3079 0.0127 [0.0090; 0.0173] 1.1%
Harvala et al, 2018 16 480 0.0333 [0.0192; 0.0536] 1.0%
Cleland et al, 2013 0 1559 0.0000 [0.0000; 0.0024] 1.1%
Dimeglio et al, 2018 2 300 0.0067 [0.0008; 0.0239] 1.0%
Ricco et al, 2016 1 199 0.0050 [0.0001; 0.0277] 0.9%
Scotto et al, 2012 0 151 0.0000 [0.0000; 0.0241] 0.8%
Mansuy et al, 2016 105 10569 0.0099 [0.0081; 0.0120] 1.1%
Marcantonio et al, 2018 2 198 0.0101 [0.0012; 0.0360] 0.9%
Mansuy et al, 2015 111 3353 0.0331 [0.0273; 0.0397] 1.1%
Krumbholz et al, 2014 60 537 0.1117 [0.0864; 0.1415] 1.0%
Kantala et al, 2015 1 385 0.0026 [0.0001; 0.0144] 1.0%
Korzeniewski et al, 2018 1 253 0.0040 [0.0001; 0.0218] 0.9%
Kaba et al, 2010 12 490 0.0245 [0.0127; 0.0424] 1.0%
Bura et al, 2018 1 138 0.0072 [0.0002; 0.0397] 0.8%
Abe et al, 2004 2 341 0.0059 [0.0007; 0.0210] 1.0%
Krumbholz et al, 2014 0 1646 0.0000 [0.0000; 0.0022] 1.1%
Christophe et al, 2004 0 315 0.0000 [0.0000; 0.0116] 1.0%
Maria et al, 2010 7 1040 0.0067 [0.0027; 0.0138] 1.1%
Katrina et al,2018 9 94302 0.0001 [0.0000; 0.0002] 1.1%
Random effects model 146322 0.0079 [0.0030; 0.0151] 26.5%
Heterogeneity: $I^2$ = 99%, $\tau^2$ = 0.0074, $p$ = 0
Continent = Multiple
Abe et al, 2006 14 5233 0.0027 [0.0015; 0.0045] 1.1%
Baylis et al, 2012 1 165010 0.0000 [0.0000; 0.0000] 1.1%
Minuk et al, 2007 2 393 0.0051 [0.0006; 0.0183] 1.0%
Schreuder et al, 2016 1 600 0.0017 [0.0000; 0.0093] 1.0%
Random effects model 171236 0.0015 [0.0000; 0.0056] 4.2%
Heterogeneity: $I^2$ = 95%, $\tau^2$ = 0.0012, $p$ < 0.01
Continent = North America
Ditah et al, 2014 3 8814 0.0003 [0.0001; 0.0010] 1.1%
Karetnyi et al, 1999 9 734 0.0123 [0.0056; 0.0231] 1.0%
Eick et al, 2010 0 1500 0.0000 [0.0000; 0.0025] 1.1%
Lemos et al, 2000 5 1149 0.0044 [0.0014; 0.0101] 1.1%
Random effects model 12197 0.0022 [0.0000; 0.0074] 4.3%
Heterogeneity: $I^2$ = 91%, $\tau^2$ = 0.0014, $p$ < 0.01
Continent = South America
Castilho et al, 2017 1 500 0.0020 [0.0001; 0.0111] 1.0%
Vitral et al, 2014 7 388 0.0180 [0.0073; 0.0368] 1.0%
Goncales et al, 2000 38 176 0.2159 [0.1576; 0.2841] 0.9%
Pujol et al, 1994 3 611 0.0049 [0.0010; 0.0143] 1.0%
Konomi et al, 1999 10 574 0.0174 [0.0084; 0.0318] 1.0%
Matins et al, 2014 4 431 0.0093 [0.0025; 0.0236] 1.0%
Random effects model 2680 0.0243 [0.0043; 0.0600] 5.9%
Heterogeneity: $I^2$ = 96%, $\tau^2$ = 0.0122, $p$ < 0.01
Random effects model 479001 0.0147 [0.0114; 0.0185] 100.0%
Heterogeneity: $I^2$ = 99%, $\tau^2$ = 0.0051, $p$ = 0
Residual heterogeneity: $I^2$ = 98%, $p$ = 0
0 0.1 0.2 0.3 0.4 0.5

**Figure S6. Forest plot of estimated pooled HEV RNA positive rate among general population based on different continents.**

Note: continent named multiple means people in the study are from more than one country and cannot be included into one continent.

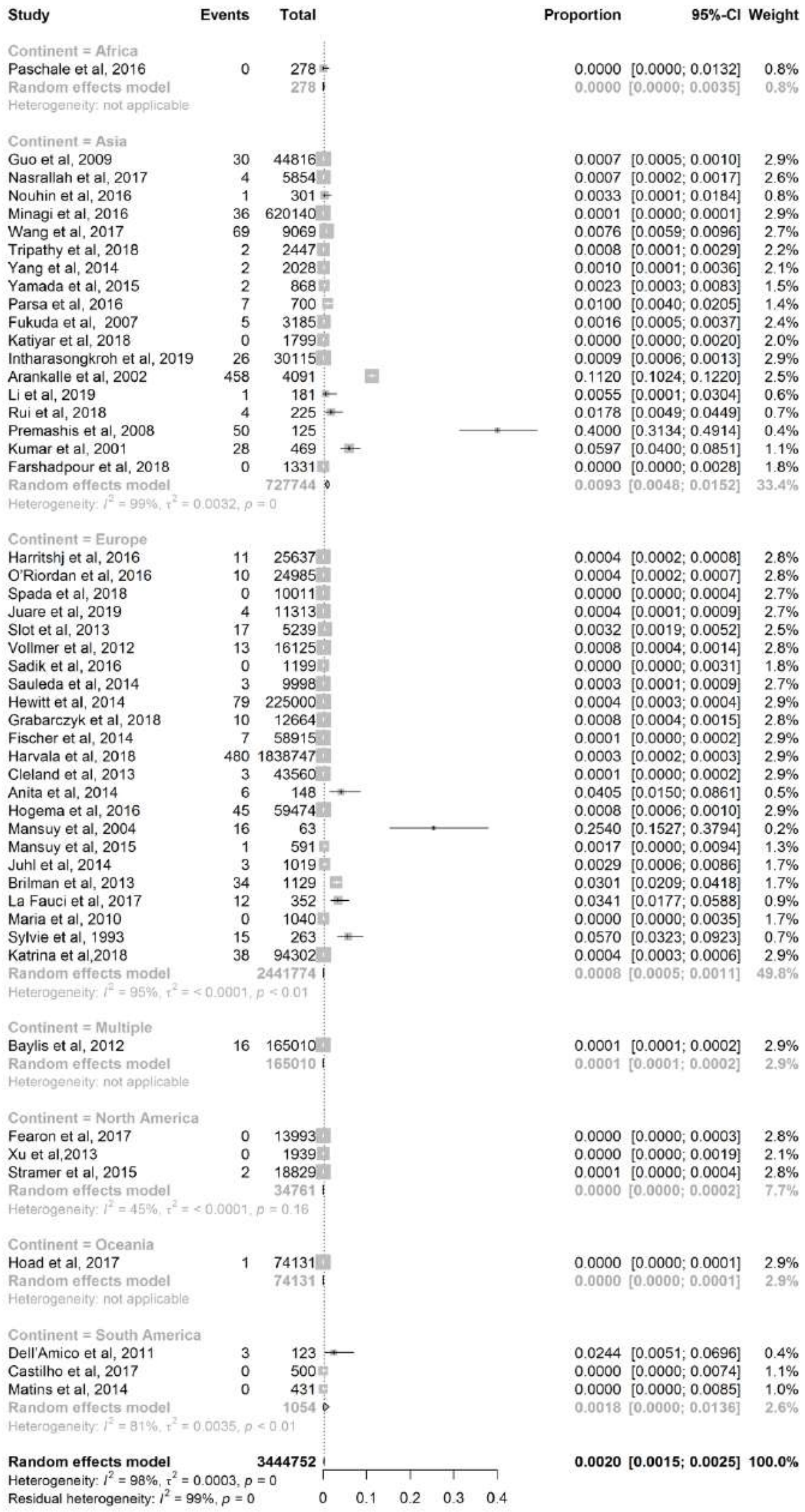

**Figure S7. Forest plot of estimated pooled anti-HEV IgG seroprevalence among general population with different ages.**

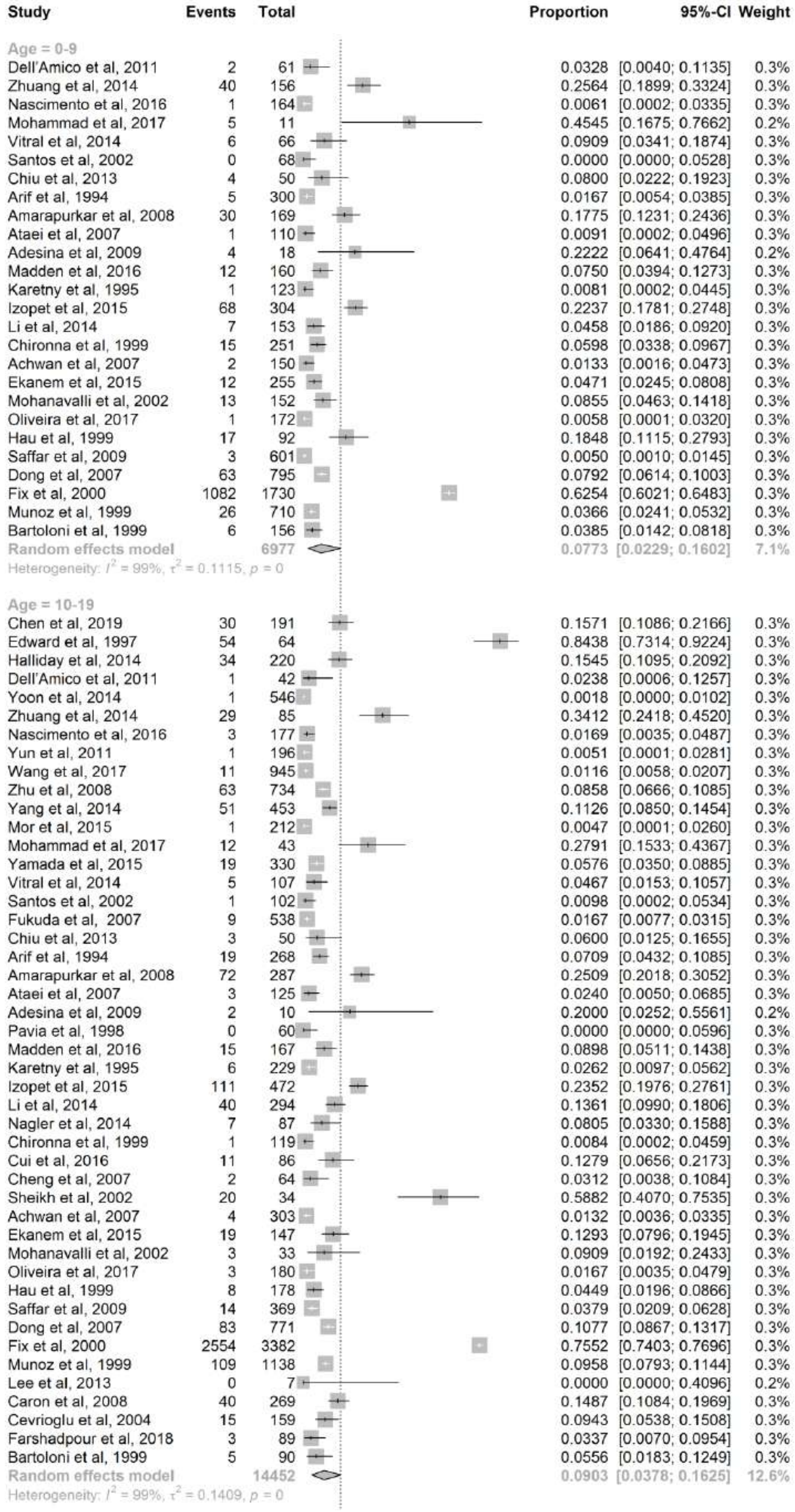

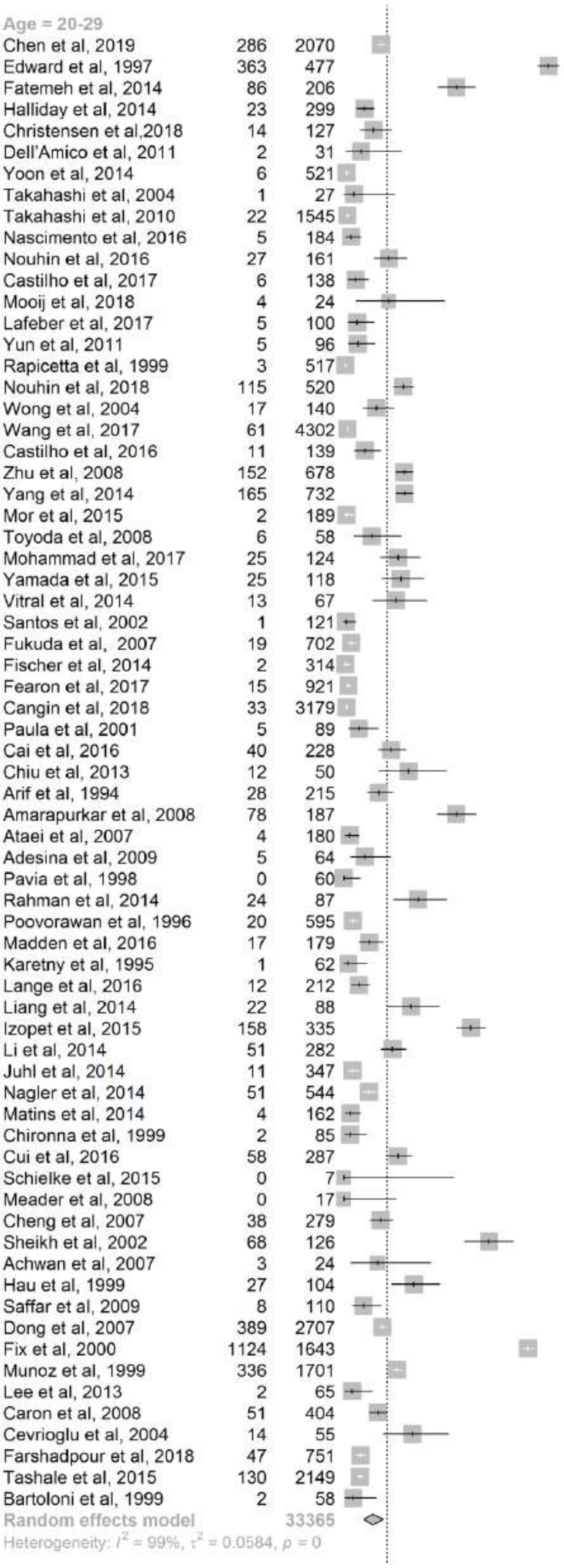

| Age = 20-29 | | | | | |
|---|---|---|---|---|---|
| Chen et al, 2019 | 286 | 2070 | 0.1382 | [0.1236; 0.1538] | 0.3% |
| Edward et al, 1997 | 363 | 477 | 0.7610 | [0.7201; 0.7986] | 0.3% |
| Fatemeh et al, 2014 | 86 | 206 | 0.4175 | [0.3493; 0.4880] | 0.3% |
| Halliday et al, 2014 | 23 | 299 | 0.0769 | [0.0494; 0.1132] | 0.3% |
| Christensen et al,2018 | 14 | 127 | 0.1102 | [0.0616; 0.1780] | 0.3% |
| Dell'Amico et al, 2011 | 2 | 31 | 0.0645 | [0.0079; 0.2142] | 0.3% |
| Yoon et al, 2014 | 6 | 521 | 0.0115 | [0.0042; 0.0249] | 0.3% |
| Takahashi et al, 2004 | 1 | 27 | 0.0370 | [0.0009; 0.1897] | 0.3% |
| Takahashi et al, 2010 | 22 | 1545 | 0.0142 | [0.0089; 0.0215] | 0.3% |
| Nascimento et al, 2016 | 5 | 184 | 0.0272 | [0.0089; 0.0623] | 0.3% |
| Nouhin et al, 2016 | 27 | 161 | 0.1677 | [0.1135; 0.2345] | 0.3% |
| Castilho et al, 2017 | 6 | 138 | 0.0435 | [0.0161; 0.0922] | 0.3% |
| Mooij et al, 2018 | 4 | 24 | 0.1667 | [0.0474; 0.3738] | 0.2% |
| Lafeber et al, 2017 | 5 | 100 | 0.0500 | [0.0164; 0.1128] | 0.3% |
| Yun et al, 2011 | 5 | 96 | 0.0521 | [0.0171; 0.1174] | 0.3% |
| Rapicetta et al, 1999 | 3 | 517 | 0.0058 | [0.0012; 0.0169] | 0.3% |
| Nouhin et al, 2018 | 115 | 520 | 0.2212 | [0.1862; 0.2593] | 0.3% |
| Wong et al, 2004 | 17 | 140 | 0.1214 | [0.0724; 0.1873] | 0.3% |
| Wang et al, 2017 | 61 | 4302 | 0.0142 | [0.0109; 0.0182] | 0.3% |
| Castilho et al, 2016 | 11 | 139 | 0.0791 | [0.0402; 0.1372] | 0.3% |
| Zhu et al, 2008 | 152 | 678 | 0.2242 | [0.1933; 0.2575] | 0.3% |
| Yang et al, 2014 | 165 | 732 | 0.2254 | [0.1956; 0.2574] | 0.3% |
| Mor et al, 2015 | 2 | 189 | 0.0106 | [0.0013; 0.0377] | 0.3% |
| Toyoda et al, 2008 | 6 | 58 | 0.1034 | [0.0389; 0.2117] | 0.3% |
| Mohammad et al, 2017 | 25 | 124 | 0.2016 | [0.1349; 0.2831] | 0.3% |
| Yamada et al, 2015 | 25 | 118 | 0.2119 | [0.1420; 0.2967] | 0.3% |
| Vitral et al, 2014 | 13 | 67 | 0.1940 | [0.1076; 0.3089] | 0.3% |
| Santos et al, 2002 | 1 | 121 | 0.0083 | [0.0002; 0.0452] | 0.3% |
| Fukuda et al, 2007 | 19 | 702 | 0.0271 | [0.0164; 0.0419] | 0.3% |
| Fischer et al, 2014 | 2 | 314 | 0.0064 | [0.0008; 0.0228] | 0.3% |
| Fearon et al, 2017 | 15 | 921 | 0.0163 | [0.0091; 0.0267] | 0.3% |
| Cangin et al, 2018 | 33 | 3179 | 0.0104 | [0.0072; 0.0145] | 0.3% |
| Paula et al, 2001 | 5 | 89 | 0.0562 | [0.0185; 0.1263] | 0.3% |
| Cai et al, 2016 | 40 | 228 | 0.1754 | [0.1284; 0.2311] | 0.3% |
| Chiu et al, 2013 | 12 | 50 | 0.2400 | [0.1306; 0.3817] | 0.3% |
| Arif et al, 1994 | 28 | 215 | 0.1302 | [0.0883; 0.1827] | 0.3% |
| Amarapurkar et al, 2008 | 78 | 187 | 0.4171 | [0.3456; 0.4913] | 0.3% |
| Ataei et al, 2007 | 4 | 180 | 0.0222 | [0.0061; 0.0559] | 0.3% |
| Adesina et al, 2009 | 5 | 64 | 0.0781 | [0.0259; 0.1730] | 0.3% |
| Pavia et al, 1998 | 0 | 60 | 0.0000 | [0.0000; 0.0596] | 0.3% |
| Rahman et al, 2014 | 24 | 87 | 0.2759 | [0.1854; 0.3821] | 0.3% |
| Poovorawan et al, 1996 | 20 | 595 | 0.0336 | [0.0207; 0.0514] | 0.3% |
| Madden et al, 2016 | 17 | 179 | 0.0950 | [0.0563; 0.1477] | 0.3% |
| Karetny et al, 1995 | 1 | 62 | 0.0161 | [0.0004; 0.0866] | 0.3% |
| Lange et al, 2016 | 12 | 212 | 0.0566 | [0.0296; 0.0968] | 0.3% |
| Liang et al, 2014 | 22 | 88 | 0.2500 | [0.1638; 0.3537] | 0.3% |
| Izopet et al, 2015 | 158 | 335 | 0.4716 | [0.4172; 0.5266] | 0.3% |
| Li et al, 2014 | 51 | 282 | 0.1809 | [0.1377; 0.2308] | 0.3% |
| Juhl et al, 2014 | 11 | 347 | 0.0317 | [0.0159; 0.0560] | 0.3% |
| Nagler et al, 2014 | 51 | 544 | 0.0938 | [0.0706; 0.1214] | 0.3% |
| Matins et al, 2014 | 4 | 162 | 0.0247 | [0.0068; 0.0620] | 0.3% |
| Chironna et al, 1999 | 2 | 85 | 0.0235 | [0.0029; 0.0824] | 0.3% |
| Cui et al, 2016 | 58 | 287 | 0.2021 | [0.1572; 0.2533] | 0.3% |
| Schielke et al, 2015 | 0 | 7 | 0.0000 | [0.0000; 0.4096] | 0.2% |
| Meader et al, 2008 | 0 | 17 | 0.0000 | [0.0000; 0.1951] | 0.2% |
| Cheng et al, 2007 | 38 | 279 | 0.1362 | [0.0982; 0.1821] | 0.3% |
| Sheikh et al, 2002 | 68 | 126 | 0.5397 | [0.4486; 0.6288] | 0.3% |
| Achwan et al, 2007 | 3 | 24 | 0.1250 | [0.0266; 0.3236] | 0.2% |
| Hau et al, 1999 | 27 | 104 | 0.2596 | [0.1786; 0.3548] | 0.3% |
| Saffar et al, 2009 | 8 | 110 | 0.0727 | [0.0319; 0.1383] | 0.3% |
| Dong et al, 2007 | 389 | 2707 | 0.1437 | [0.1307; 0.1575] | 0.3% |
| Fix et al, 2000 | 1124 | 1643 | 0.6841 | [0.6610; 0.7066] | 0.3% |
| Munoz et al, 1999 | 336 | 1701 | 0.1975 | [0.1789; 0.2173] | 0.3% |
| Lee et al, 2013 | 2 | 65 | 0.0308 | [0.0037; 0.1068] | 0.3% |
| Caron et al, 2008 | 51 | 404 | 0.1262 | [0.0955; 0.1626] | 0.3% |
| Cevrioglu et al, 2004 | 14 | 55 | 0.2545 | [0.1467; 0.3900] | 0.3% |
| Farshadpour et al, 2018 | 47 | 751 | 0.0626 | [0.0463; 0.0824] | 0.3% |
| Tashale et al, 2015 | 130 | 2149 | 0.0605 | [0.0508; 0.0714] | 0.3% |
| Bartoloni et al, 1999 | 2 | 58 | 0.0345 | [0.0042; 0.1191] | 0.3% |
| Random effects model | | 33365 | 0.1078 | [0.0744; 0.1464] | 19.0% |

Heterogeneity: $I^2 = 99\%$, $\tau^2 = 0.0584$, $p = 0$

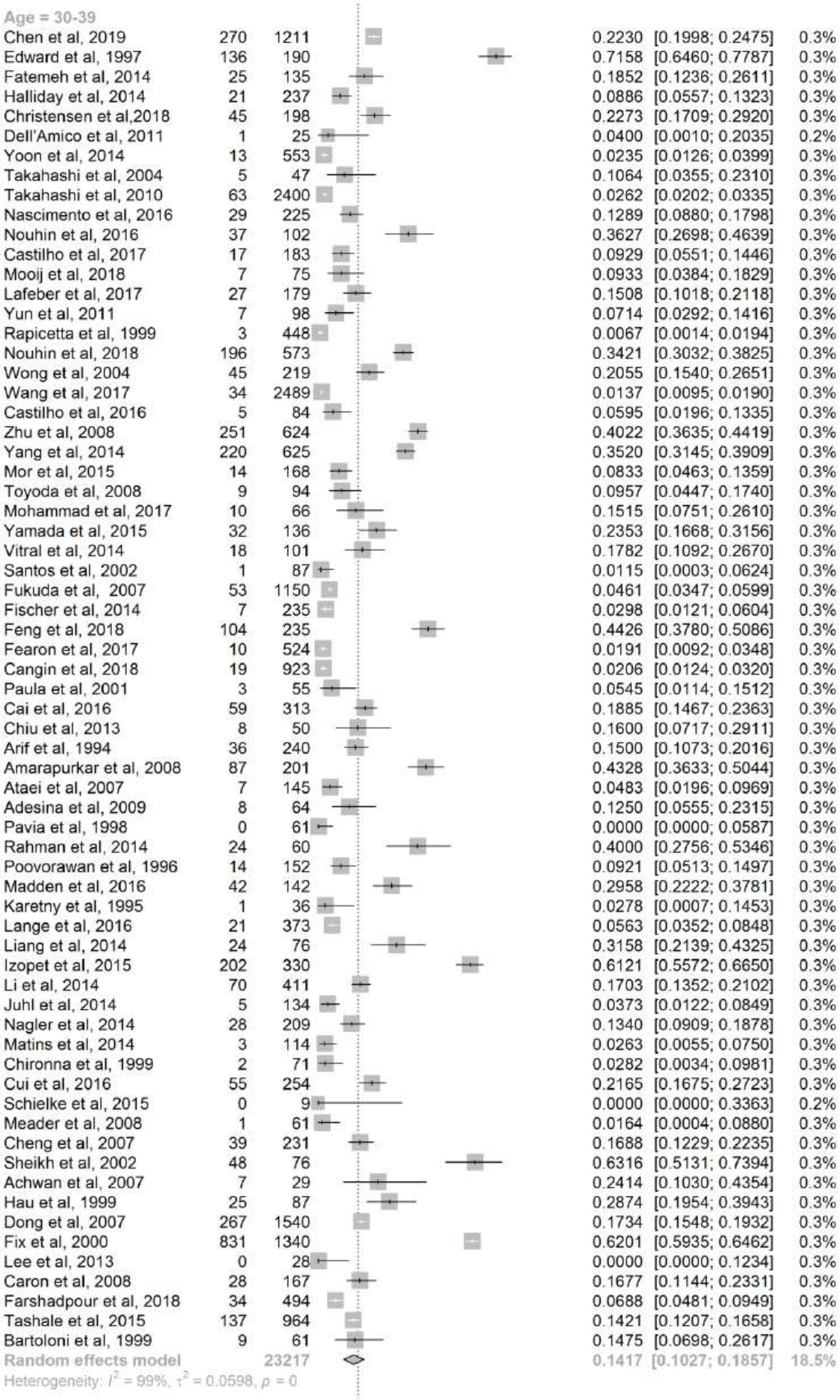
Age = 30-39
Chen et al, 2019 270 1211 0.2230 [0.1998; 0.2475] 0.3%
Edward et al, 1997 136 190 0.7158 [0.6460; 0.7787] 0.3%
Fatemeh et al, 2014 25 135 0.1852 [0.1236; 0.2611] 0.3%
Halliday et al, 2014 21 237 0.0886 [0.0557; 0.1323] 0.3%
Christensen et al,2018 45 198 0.2273 [0.1709; 0.2920] 0.3%
Dell'Amico et al, 2011 1 25 0.0400 [0.0010; 0.2035] 0.2%
Yoon et al, 2014 13 553 0.0235 [0.0126; 0.0399] 0.3%
Takahashi et al, 2004 5 47 0.1064 [0.0355; 0.2310] 0.3%
Takahashi et al, 2010 63 2400 0.0262 [0.0202; 0.0335] 0.3%
Nascimento et al, 2016 29 225 0.1289 [0.0880; 0.1798] 0.3%
Nouhin et al, 2016 37 102 0.3627 [0.2698; 0.4639] 0.3%
Castilho et al, 2017 17 183 0.0929 [0.0551; 0.1446] 0.3%
Mooij et al, 2018 7 75 0.0933 [0.0384; 0.1829] 0.3%
Lafeber et al, 2017 27 179 0.1508 [0.1018; 0.2118] 0.3%
Yun et al, 2011 7 98 0.0714 [0.0292; 0.1416] 0.3%
Rapicetta et al, 1999 3 448 0.0067 [0.0014; 0.0194] 0.3%
Nouhin et al, 2018 196 573 0.3421 [0.3032; 0.3825] 0.3%
Wong et al, 2004 45 219 0.2055 [0.1540; 0.2651] 0.3%
Wang et al, 2017 34 2489 0.0137 [0.0095; 0.0190] 0.3%
Castilho et al, 2016 5 84 0.0595 [0.0196; 0.1335] 0.3%
Zhu et al, 2008 251 624 0.4022 [0.3635; 0.4419] 0.3%
Yang et al, 2014 220 625 0.3520 [0.3145; 0.3909] 0.3%
Mor et al, 2015 14 168 0.0833 [0.0463; 0.1359] 0.3%
Toyoda et al, 2008 9 94 0.0957 [0.0447; 0.1740] 0.3%
Mohammad et al, 2017 10 66 0.1515 [0.0751; 0.2610] 0.3%
Yamada et al, 2015 32 136 0.2353 [0.1668; 0.3156] 0.3%
Vitral et al, 2014 18 101 0.1782 [0.1092; 0.2670] 0.3%
Santos et al, 2002 1 87 0.0115 [0.0003; 0.0624] 0.3%
Fukuda et al, 2007 53 1150 0.0461 [0.0347; 0.0599] 0.3%
Fischer et al, 2014 7 235 0.0298 [0.0121; 0.0604] 0.3%
Feng et al, 2018 104 235 0.4426 [0.3780; 0.5086] 0.3%
Fearon et al, 2017 10 524 0.0191 [0.0092; 0.0348] 0.3%
Cangin et al, 2018 19 923 0.0206 [0.0124; 0.0320] 0.3%
Paula et al, 2001 3 55 0.0545 [0.0114; 0.1512] 0.3%
Cai et al, 2016 59 313 0.1885 [0.1467; 0.2363] 0.3%
Chiu et al, 2013 8 50 0.1600 [0.0717; 0.2911] 0.3%
Arif et al, 1994 36 240 0.1500 [0.1073; 0.2016] 0.3%
Amarapurkar et al, 2008 87 201 0.4328 [0.3633; 0.5044] 0.3%
Ataei et al, 2007 7 145 0.0483 [0.0196; 0.0969] 0.3%
Adesina et al, 2009 8 64 0.1250 [0.0555; 0.2315] 0.3%
Pavia et al, 1998 0 61 0.0000 [0.0000; 0.0587] 0.3%
Rahman et al, 2014 24 60 0.4000 [0.2756; 0.5346] 0.3%
Poovorawan et al, 1996 14 152 0.0921 [0.0513; 0.1497] 0.3%
Madden et al, 2016 42 142 0.2958 [0.2222; 0.3781] 0.3%
Karetny et al, 1995 1 36 0.0278 [0.0007; 0.1453] 0.3%
Lange et al, 2016 21 373 0.0563 [0.0352; 0.0848] 0.3%
Liang et al, 2014 24 76 0.3158 [0.2139; 0.4325] 0.3%
Izopet et al, 2015 202 330 0.6121 [0.5572; 0.6650] 0.3%
Li et al, 2014 70 411 0.1703 [0.1352; 0.2102] 0.3%
Juhl et al, 2014 5 134 0.0373 [0.0122; 0.0849] 0.3%
Nagler et al, 2014 28 209 0.1340 [0.0909; 0.1878] 0.3%
Matins et al, 2014 3 114 0.0263 [0.0055; 0.0750] 0.3%
Chironna et al, 1999 2 71 0.0282 [0.0034; 0.0981] 0.3%
Cui et al, 2016 55 254 0.2165 [0.1675; 0.2723] 0.3%
Schielke et al, 2015 0 9 0.0000 [0.0000; 0.3363] 0.2%
Meader et al, 2008 1 61 0.0164 [0.0004; 0.0880] 0.3%
Cheng et al, 2007 39 231 0.1688 [0.1229; 0.2235] 0.3%
Sheikh et al, 2002 48 76 0.6316 [0.5131; 0.7394] 0.3%
Achwan et al, 2007 7 29 0.2414 [0.1030; 0.4354] 0.3%
Hau et al, 1999 25 87 0.2874 [0.1954; 0.3943] 0.3%
Dong et al, 2007 267 1540 0.1734 [0.1548; 0.1932] 0.3%
Fix et al, 2000 831 1340 0.6201 [0.5935; 0.6462] 0.3%
Lee et al, 2013 0 28 0.0000 [0.0000; 0.1234] 0.3%
Caron et al, 2008 28 167 0.1677 [0.1144; 0.2331] 0.3%
Farshadpour et al, 2018 34 494 0.0688 [0.0481; 0.0949] 0.3%
Tashale et al, 2015 137 964 0.1421 [0.1207; 0.1658] 0.3%
Bartoloni et al, 1999 9 61 0.1475 [0.0698; 0.2617] 0.3%
Random effects model 23217 0.1417 [0.1027; 0.1857] 18.5%
Heterogeneity: $I^2$ = 99%, $\tau^2$ = 0.0598, $p$ = 0

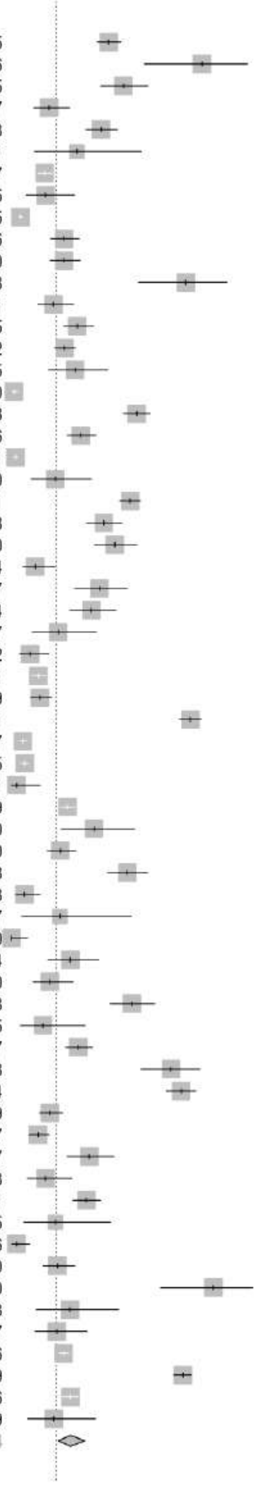

| Study | Events | Total | Proportion | 95% CI | Weight |
|---|---|---|---|---|---|
| **Age = 40-49** | | | | | |
| Chen et al, 2019 | 178 | 505 | 0.3525 | [0.3108; 0.3959] | 0.3% |
| Edward et al, 1997 | 18 | 26 | 0.6923 | [0.4821; 0.8567] | 0.3% |
| Fatemeh et al, 2014 | 55 | 135 | 0.4074 | [0.3237; 0.4953] | 0.3% |
| Halliday et al, 2014 | 16 | 117 | 0.1368 | [0.0802; 0.2126] | 0.3% |
| Christensen et al,2018 | 89 | 273 | 0.3260 | [0.2707; 0.3851] | 0.3% |
| Dell'Amico et al, 2011 | 5 | 21 | 0.2381 | [0.0822; 0.4717] | 0.2% |
| Yoon et al, 2014 | 67 | 557 | 0.1203 | [0.0944; 0.1502] | 0.3% |
| Takahashi et al, 2004 | 8 | 65 | 0.1231 | [0.0547; 0.2282] | 0.3% |
| Takahashi et al, 2010 | 118 | 3455 | 0.0342 | [0.0283; 0.0408] | 0.3% |
| Zhuang et al, 2014 | 47 | 246 | 0.1911 | [0.1439; 0.2458] | 0.3% |
| Nascimento et al, 2016 | 42 | 220 | 0.1909 | [0.1412; 0.2492] | 0.3% |
| Nouhin et al, 2016 | 24 | 38 | 0.6316 | [0.4599; 0.7819] | 0.3% |
| Castilho et al, 2017 | 20 | 131 | 0.1527 | [0.0958; 0.2259] | 0.3% |
| Mooij et al, 2018 | 61 | 255 | 0.2392 | [0.1882; 0.2964] | 0.3% |
| Lafeber et al, 2017 | 83 | 432 | 0.1921 | [0.1560; 0.2325] | 0.3% |
| Yun et al, 2011 | 15 | 65 | 0.2308 | [0.1353; 0.3519] | 0.3% |
| Rapicetta et al, 1999 | 4 | 430 | 0.0093 | [0.0025; 0.0236] | 0.3% |
| Nouhin et al, 2018 | 190 | 418 | 0.4545 | [0.4061; 0.5037] | 0.3% |
| Wong et al, 2004 | 72 | 286 | 0.2517 | [0.2025; 0.3062] | 0.3% |
| Wang et al, 2017 | 17 | 1161 | 0.0146 | [0.0086; 0.0233] | 0.3% |
| Castilho et al, 2016 | 8 | 50 | 0.1600 | [0.0717; 0.2911] | 0.3% |
| Zhu et al, 2008 | 293 | 681 | 0.4302 | [0.3927; 0.4684] | 0.3% |
| Yang et al, 2014 | 73 | 218 | 0.3349 | [0.2726; 0.4017] | 0.3% |
| Mor et al, 2015 | 60 | 160 | 0.3750 | [0.2998; 0.4549] | 0.3% |
| Toyoda et al, 2008 | 9 | 104 | 0.0865 | [0.0403; 0.1579] | 0.3% |
| Mohammad et al, 2017 | 31 | 97 | 0.3196 | [0.2285; 0.4220] | 0.3% |
| Yamada et al, 2015 | 36 | 124 | 0.2903 | [0.2123; 0.3786] | 0.3% |
| Vitral et al, 2014 | 8 | 47 | 0.1702 | [0.0765; 0.3081] | 0.3% |
| Santos et al, 2002 | 7 | 102 | 0.0686 | [0.0280; 0.1363] | 0.3% |
| Fukuda et al, 2007 | 55 | 561 | 0.0980 | [0.0747; 0.1257] | 0.3% |
| Fischer et al, 2014 | 27 | 259 | 0.1042 | [0.0698; 0.1480] | 0.3% |
| Feng et al, 2018 | 364 | 561 | 0.6488 | [0.6077; 0.6884] | 0.3% |
| Fearon et al, 2017 | 30 | 727 | 0.0413 | [0.0280; 0.0584] | 0.3% |
| Cangin et al, 2018 | 47 | 965 | 0.0487 | [0.0360; 0.0642] | 0.3% |
| Paula et al, 2001 | 1 | 51 | 0.0196 | [0.0005; 0.1045] | 0.3% |
| Cai et al, 2016 | 187 | 919 | 0.2035 | [0.1779; 0.2310] | 0.3% |
| Chiu et al, 2013 | 15 | 50 | 0.3000 | [0.1786; 0.4461] | 0.3% |
| Arif et al, 1994 | 39 | 220 | 0.1773 | [0.1292; 0.2343] | 0.3% |
| Amarapurkar et al, 2008 | 79 | 188 | 0.4202 | [0.3488; 0.4942] | 0.3% |
| Ataei et al, 2007 | 5 | 108 | 0.0463 | [0.0152; 0.1047] | 0.3% |
| Adesina et al, 2009 | 3 | 17 | 0.1765 | [0.0380; 0.4343] | 0.2% |
| Pavia et al, 1998 | 0 | 60 | 0.0000 | [0.0000; 0.0596] | 0.3% |
| Rahman et al, 2014 | 18 | 84 | 0.2143 | [0.1322; 0.3174] | 0.3% |
| Poovorawan et al, 1996 | 14 | 100 | 0.1400 | [0.0787; 0.2237] | 0.3% |
| Madden et al, 2016 | 67 | 153 | 0.4379 | [0.3579; 0.5203] | 0.3% |
| Karetny et al, 1995 | 4 | 35 | 0.1143 | [0.0320; 0.2674] | 0.3% |
| Lange et al, 2016 | 77 | 317 | 0.2429 | [0.1967; 0.2940] | 0.3% |
| Liang et al, 2014 | 51 | 88 | 0.5795 | [0.4695; 0.6840] | 0.3% |
| Izopet et al, 2015 | 206 | 334 | 0.6168 | [0.5623; 0.6692] | 0.3% |
| Li et al, 2014 | 39 | 279 | 0.1398 | [0.1013; 0.1861] | 0.3% |
| Juhl et al, 2014 | 27 | 277 | 0.0975 | [0.0652; 0.1386] | 0.3% |
| Nagler et al, 2014 | 33 | 117 | 0.2821 | [0.2028; 0.3727] | 0.3% |
| Matins et al, 2014 | 9 | 73 | 0.1233 | [0.0580; 0.2212] | 0.3% |
| Cui et al, 2016 | 90 | 331 | 0.2719 | [0.2247; 0.3233] | 0.3% |
| Schielke et al, 2015 | 4 | 25 | 0.1600 | [0.0454; 0.3608] | 0.2% |
| Meader et al, 2008 | 2 | 106 | 0.0189 | [0.0023; 0.0665] | 0.3% |
| Cheng et al, 2007 | 30 | 180 | 0.1667 | [0.1154; 0.2293] | 0.3% |
| Sheikh et al, 2002 | 22 | 30 | 0.7333 | [0.5411; 0.8772] | 0.3% |
| Achwan et al, 2007 | 7 | 33 | 0.2121 | [0.0898; 0.3891] | 0.3% |
| Hau et al, 1999 | 11 | 67 | 0.1642 | [0.0849; 0.2748] | 0.3% |
| Dong et al, 2007 | 359 | 1896 | 0.1893 | [0.1719; 0.2077] | 0.3% |
| Fix et al, 2000 | 572 | 919 | 0.6224 | [0.5902; 0.6539] | 0.3% |
| Tashale et al, 2015 | 157 | 736 | 0.2133 | [0.1842; 0.2447] | 0.3% |
| Bartoloni et al, 1999 | 6 | 39 | 0.1538 | [0.0586; 0.3053] | 0.3% |
| **Random effects model** | | 21324 | 0.2153 | [0.1682; 0.2665] | 17.6% |

Heterogeneity: $I^2 = 99\%$, $\tau^2 = 0.0573$, $p = 0$

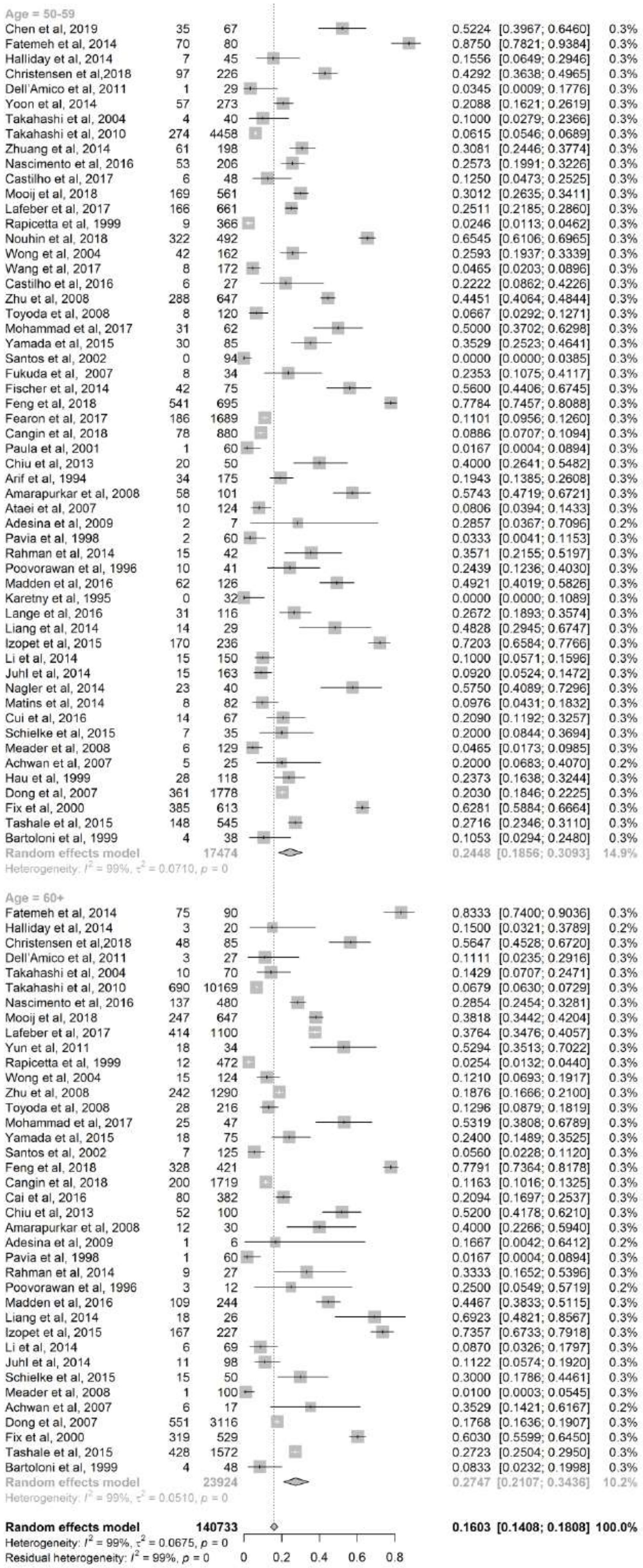
Age = 50-59
Chen et al, 2019 35 67 0.5224 [0.3967; 0.6460] 0.3%
Fatemeh et al, 2014 70 80 0.8750 [0.7821; 0.9384] 0.3%
Halliday et al, 2014 7 45 0.1556 [0.0649; 0.2946] 0.3%
Christensen et al,2018 97 226 0.4292 [0.3638; 0.4965] 0.3%
Dell'Amico et al, 2011 1 29 0.0345 [0.0009; 0.1776] 0.3%
Yoon et al, 2014 57 273 0.2088 [0.1621; 0.2619] 0.3%
Takahashi et al, 2004 4 40 0.1000 [0.0279; 0.2366] 0.3%
Takahashi et al, 2010 274 4458 0.0615 [0.0546; 0.0689] 0.3%
Zhuang et al, 2014 61 198 0.3081 [0.2446; 0.3774] 0.3%
Nascimento et al, 2016 53 206 0.2573 [0.1991; 0.3226] 0.3%
Castilho et al, 2017 6 48 0.1250 [0.0473; 0.2525] 0.3%
Mooij et al, 2018 169 561 0.3012 [0.2635; 0.3411] 0.3%
Lafeber et al, 2017 166 661 0.2511 [0.2185; 0.2860] 0.3%
Rapicetta et al, 1999 9 366 0.0246 [0.0113; 0.0462] 0.3%
Nouhin et al, 2018 322 492 0.6545 [0.6106; 0.6965] 0.3%
Wong et al, 2004 42 162 0.2593 [0.1937; 0.3339] 0.3%
Wang et al, 2017 8 172 0.0465 [0.0203; 0.0896] 0.3%
Castilho et al, 2016 6 27 0.2222 [0.0862; 0.4226] 0.3%
Zhu et al, 2008 288 647 0.4451 [0.4064; 0.4844] 0.3%
Toyoda et al, 2008 8 120 0.0667 [0.0292; 0.1271] 0.3%
Mohammad et al, 2017 31 62 0.5000 [0.3702; 0.6298] 0.3%
Yamada et al, 2015 30 85 0.3529 [0.2523; 0.4641] 0.3%
Santos et al, 2002 0 94 0.0000 [0.0000; 0.0385] 0.3%
Fukuda et al, 2007 8 34 0.2353 [0.1075; 0.4117] 0.3%
Fischer et al, 2014 42 75 0.5600 [0.4406; 0.6745] 0.3%
Feng et al, 2018 541 695 0.7784 [0.7457; 0.8088] 0.3%
Fearon et al, 2017 186 1689 0.1101 [0.0956; 0.1260] 0.3%
Cangin et al, 2018 78 880 0.0886 [0.0707; 0.1094] 0.3%
Paula et al, 2001 1 60 0.0167 [0.0004; 0.0894] 0.3%
Chiu et al, 2013 20 50 0.4000 [0.2641; 0.5482] 0.3%
Arif et al, 1994 34 175 0.1943 [0.1385; 0.2608] 0.3%
Amarapurkar et al, 2008 58 101 0.5743 [0.4719; 0.6721] 0.3%
Ataei et al, 2007 10 124 0.0806 [0.0394; 0.1433] 0.3%
Adesina et al, 2009 2 7 0.2857 [0.0367; 0.7096] 0.2%
Pavia et al, 1998 2 60 0.0333 [0.0041; 0.1153] 0.3%
Rahman et al, 2014 15 42 0.3571 [0.2155; 0.5197] 0.3%
Poovorawan et al, 1996 10 41 0.2439 [0.1236; 0.4030] 0.3%
Madden et al, 2016 62 126 0.4921 [0.4019; 0.5826] 0.3%
Karetny et al, 1995 0 32 0.0000 [0.0000; 0.1089] 0.3%
Lange et al, 2016 31 116 0.2672 [0.1893; 0.3574] 0.3%
Liang et al, 2014 14 29 0.4828 [0.2945; 0.6747] 0.3%
Izopet et al, 2015 170 236 0.7203 [0.6584; 0.7766] 0.3%
Li et al, 2014 15 150 0.1000 [0.0571; 0.1596] 0.3%
Juhl et al, 2014 15 163 0.0920 [0.0524; 0.1472] 0.3%
Nagler et al, 2014 23 40 0.5750 [0.4089; 0.7296] 0.3%
Matins et al, 2014 8 82 0.0976 [0.0431; 0.1832] 0.3%
Cui et al, 2016 14 67 0.2090 [0.1192; 0.3257] 0.3%
Schielke et al, 2015 7 35 0.2000 [0.0844; 0.3694] 0.3%
Meader et al, 2008 6 129 0.0465 [0.0173; 0.0985] 0.3%
Achwan et al, 2007 5 25 0.2000 [0.0683; 0.4070] 0.2%
Hau et al, 1999 28 118 0.2373 [0.1638; 0.3244] 0.3%
Dong et al, 2007 361 1778 0.2030 [0.1846; 0.2225] 0.3%
Fix et al, 2000 385 613 0.6281 [0.5884; 0.6664] 0.3%
Tashale et al, 2015 148 545 0.2716 [0.2346; 0.3110] 0.3%
Bartoloni et al, 1999 4 38 0.1053 [0.0294; 0.2480] 0.3%
Random effects model 17474 0.2448 [0.1856; 0.3093] 14.9%
Heterogeneity: $I^2$ = 99%, $\tau^2$ = 0.0710, $p$ = 0
Age = 60+
Fatemeh et al, 2014 75 90 0.8333 [0.7400; 0.9036] 0.3%
Halliday et al, 2014 3 20 0.1500 [0.0321; 0.3789] 0.2%
Christensen et al,2018 48 85 0.5647 [0.4528; 0.6720] 0.3%
Dell'Amico et al, 2011 3 27 0.1111 [0.0235; 0.2916] 0.3%
Takahashi et al, 2004 10 70 0.1429 [0.0707; 0.2471] 0.3%
Takahashi et al, 2010 690 10169 0.0679 [0.0630; 0.0729] 0.3%
Nascimento et al, 2016 137 480 0.2854 [0.2454; 0.3281] 0.3%
Mooij et al, 2018 247 647 0.3818 [0.3442; 0.4204] 0.3%
Lafeber et al, 2017 414 1100 0.3764 [0.3476; 0.4057] 0.3%
Yun et al, 2011 18 34 0.5294 [0.3513; 0.7022] 0.3%
Rapicetta et al, 1999 12 472 0.0254 [0.0132; 0.0440] 0.3%
Wong et al, 2004 15 124 0.1210 [0.0693; 0.1917] 0.3%
Zhu et al, 2008 242 1290 0.1876 [0.1666; 0.2100] 0.3%
Toyoda et al, 2008 28 216 0.1296 [0.0879; 0.1819] 0.3%
Mohammad et al, 2017 25 47 0.5319 [0.3808; 0.6789] 0.3%
Yamada et al, 2015 18 75 0.2400 [0.1489; 0.3525] 0.3%
Santos et al, 2002 7 125 0.0560 [0.0228; 0.1120] 0.3%
Feng et al, 2018 328 421 0.7791 [0.7364; 0.8178] 0.3%
Cangin et al, 2018 200 1719 0.1163 [0.1016; 0.1325] 0.3%
Cai et al, 2016 80 382 0.2094 [0.1697; 0.2537] 0.3%
Chiu et al, 2013 52 100 0.5200 [0.4178; 0.6210] 0.3%
Amarapurkar et al, 2008 12 30 0.4000 [0.2266; 0.5940] 0.3%
Adesina et al, 2009 1 6 0.1667 [0.0042; 0.6412] 0.2%
Pavia et al, 1998 1 60 0.0167 [0.0004; 0.0894] 0.3%
Rahman et al, 2014 9 27 0.3333 [0.1652; 0.5396] 0.3%
Poovorawan et al, 1996 3 12 0.2500 [0.0549; 0.5719] 0.2%
Madden et al, 2016 109 244 0.4467 [0.3833; 0.5115] 0.3%
Liang et al, 2014 18 26 0.6923 [0.4821; 0.8567] 0.3%
Izopet et al, 2015 167 227 0.7357 [0.6733; 0.7918] 0.3%
Li et al, 2014 6 69 0.0870 [0.0326; 0.1797] 0.3%
Juhl et al, 2014 11 98 0.1122 [0.0574; 0.1920] 0.3%
Schielke et al, 2015 15 50 0.3000 [0.1786; 0.4461] 0.3%
Meader et al, 2008 1 100 0.0100 [0.0003; 0.0545] 0.3%
Achwan et al, 2007 6 17 0.3529 [0.1421; 0.6167] 0.2%
Dong et al, 2007 551 3116 0.1768 [0.1636; 0.1907] 0.3%
Fix et al, 2000 319 529 0.6030 [0.5599; 0.6450] 0.3%
Tashale et al, 2015 428 1572 0.2723 [0.2504; 0.2950] 0.3%
Bartoloni et al, 1999 4 48 0.0833 [0.0232; 0.1998] 0.3%
Random effects model 23924 0.2747 [0.2107; 0.3436] 10.2%
Heterogeneity: $I^2$ = 99%, $\tau^2$ = 0.0510, $p$ = 0
Random effects model 140733 0.1603 [0.1408; 0.1808] 100.0%
Heterogeneity: $I^2$ = 99%, $\tau^2$ = 0.0675, $p$ = 0
Residual heterogeneity: $I^2$ = 99%, $p$ = 0
0 0.2 0.4 0.6 0.8

Figure S8. Anti-HEV IgG seroprevalence among different age range for general population from different continents

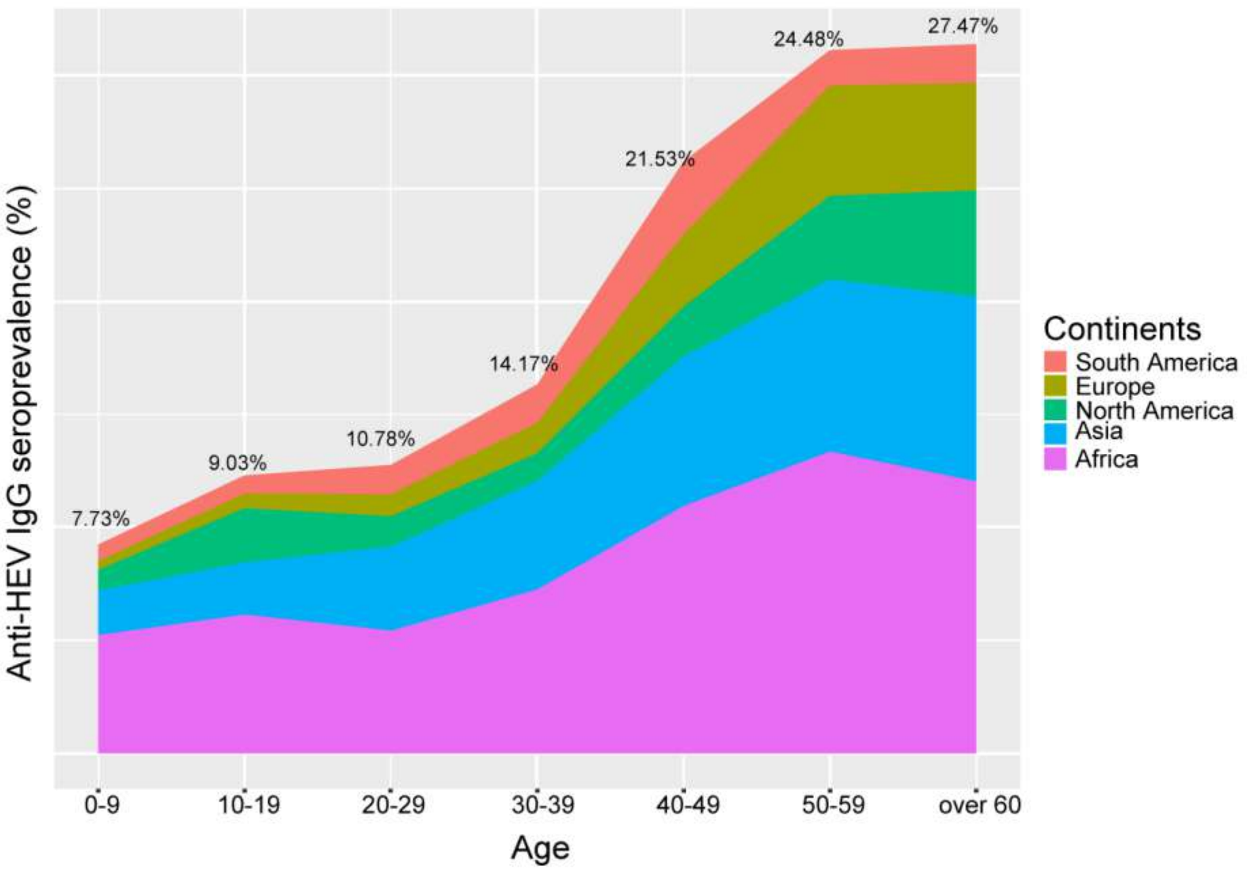

**Figure S9. Forest plot of estimated pooled anti-HEV IgG seroprevalence among general population with different genders.**

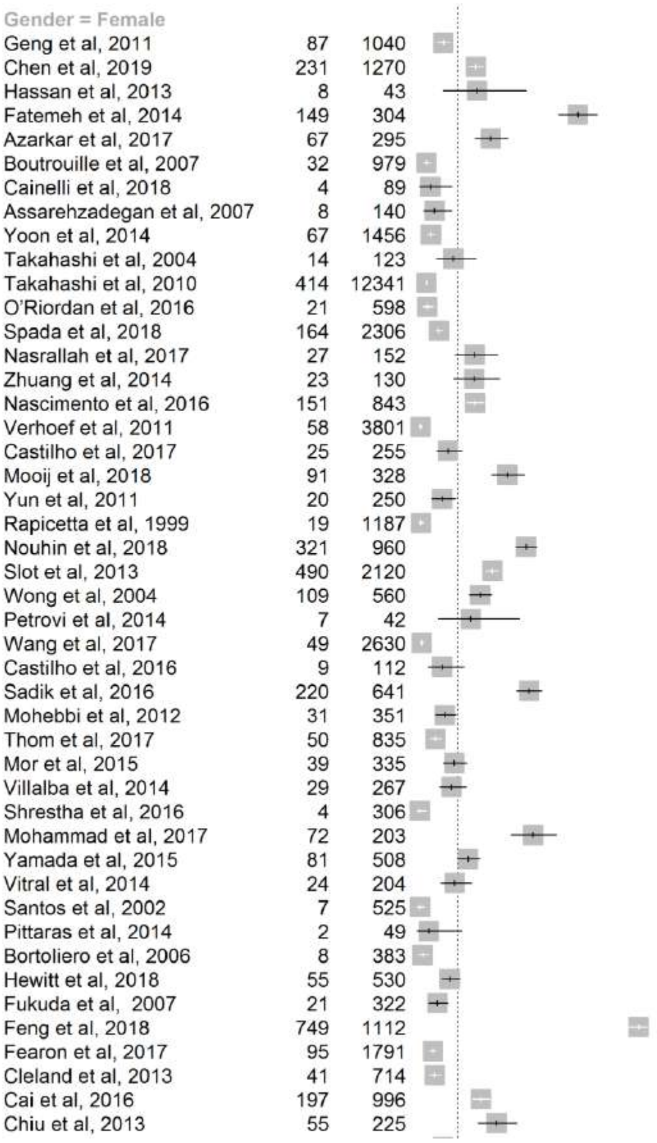

| Study | Events | Total | Proportion | 95%-CI | Weight |
|---|---|---|---|---|---|
| Gender = Female | | | | | |
| Geng et al, 2011 | 87 | 1040 | 0.0837 | [0.0675; 0.1022] | 0.4% |
| Chen et al, 2019 | 231 | 1270 | 0.1819 | [0.1610; 0.2042] | 0.4% |
| Hassan et al, 2013 | 8 | 43 | 0.1860 | [0.0839; 0.3340] | 0.4% |
| Fatemeh et al, 2014 | 149 | 304 | 0.4901 | [0.4326; 0.5478] | 0.4% |
| Azarkar et al, 2017 | 67 | 295 | 0.2271 | [0.1806; 0.2792] | 0.4% |
| Boutrouille et al, 2007 | 32 | 979 | 0.0327 | [0.0225; 0.0458] | 0.4% |
| Cainelli et al, 2018 | 4 | 89 | 0.0449 | [0.0124; 0.1111] | 0.4% |
| Assarehzadegan et al, 2007 | 8 | 140 | 0.0571 | [0.0250; 0.1095] | 0.4% |
| Yoon et al, 2014 | 67 | 1456 | 0.0460 | [0.0358; 0.0581] | 0.4% |
| Takahashi et al, 2004 | 14 | 123 | 0.1138 | [0.0636; 0.1836] | 0.4% |
| Takahashi et al, 2010 | 414 | 12341 | 0.0335 | [0.0304; 0.0369] | 0.4% |
| O'Riordan et al, 2016 | 21 | 598 | 0.0351 | [0.0219; 0.0532] | 0.4% |
| Spada et al, 2018 | 164 | 2306 | 0.0711 | [0.0610; 0.0824] | 0.4% |
| Nasrallah et al, 2017 | 27 | 152 | 0.1776 | [0.1204; 0.2478] | 0.4% |
| Zhuang et al, 2014 | 23 | 130 | 0.1769 | [0.1156; 0.2535] | 0.4% |
| Nascimento et al, 2016 | 151 | 843 | 0.1791 | [0.1538; 0.2067] | 0.4% |
| Verhoef et al, 2011 | 58 | 3801 | 0.0153 | [0.0116; 0.0197] | 0.4% |
| Castilho et al, 2017 | 25 | 255 | 0.0980 | [0.0645; 0.1413] | 0.4% |
| Mooij et al, 2018 | 91 | 328 | 0.2774 | [0.2297; 0.3293] | 0.4% |
| Yun et al, 2011 | 20 | 250 | 0.0800 | [0.0495; 0.1209] | 0.4% |
| Rapicetta et al, 1999 | 19 | 1187 | 0.0160 | [0.0097; 0.0249] | 0.4% |
| Nouhin et al, 2018 | 321 | 960 | 0.3344 | [0.3046; 0.3652] | 0.4% |
| Slot et al, 2013 | 490 | 2120 | 0.2311 | [0.2133; 0.2497] | 0.4% |
| Wong et al, 2004 | 109 | 560 | 0.1946 | [0.1626; 0.2299] | 0.4% |
| Petrovi et al, 2014 | 7 | 42 | 0.1667 | [0.0697; 0.3136] | 0.4% |
| Wang et al, 2017 | 49 | 2630 | 0.0186 | [0.0138; 0.0246] | 0.4% |
| Castilho et al, 2016 | 9 | 112 | 0.0804 | [0.0374; 0.1471] | 0.4% |
| Sadik et al, 2016 | 220 | 641 | 0.3432 | [0.3065; 0.3814] | 0.4% |
| Mohebbi et al, 2012 | 31 | 351 | 0.0883 | [0.0608; 0.1230] | 0.4% |
| Thom et al, 2017 | 50 | 835 | 0.0599 | [0.0448; 0.0782] | 0.4% |
| Mor et al, 2015 | 39 | 335 | 0.1164 | [0.0841; 0.1557] | 0.4% |
| Villalba et al, 2014 | 29 | 267 | 0.1086 | [0.0740; 0.1523] | 0.4% |
| Shrestha et al, 2016 | 4 | 306 | 0.0131 | [0.0036; 0.0331] | 0.4% |
| Mohammad et al, 2017 | 72 | 203 | 0.3547 | [0.2890; 0.4247] | 0.4% |
| Yamada et al, 2015 | 81 | 508 | 0.1594 | [0.1287; 0.1942] | 0.4% |
| Vitral et al, 2014 | 24 | 204 | 0.1176 | [0.0769; 0.1700] | 0.4% |
| Santos et al, 2002 | 7 | 525 | 0.0133 | [0.0054; 0.0273] | 0.4% |
| Pittaras et al, 2014 | 2 | 49 | 0.0408 | [0.0050; 0.1398] | 0.4% |
| Bortoliero et al, 2006 | 8 | 383 | 0.0209 | [0.0091; 0.0407] | 0.4% |
| Hewitt et al, 2018 | 55 | 530 | 0.1038 | [0.0791; 0.1329] | 0.4% |
| Fukuda et al, 2007 | 21 | 322 | 0.0652 | [0.0408; 0.0980] | 0.4% |
| Feng et al, 2018 | 749 | 1112 | 0.6736 | [0.6451; 0.7011] | 0.4% |
| Fearon et al, 2017 | 95 | 1791 | 0.0530 | [0.0431; 0.0645] | 0.4% |
| Cleland et al, 2013 | 41 | 714 | 0.0574 | [0.0415; 0.0771] | 0.4% |
| Cai et al, 2016 | 197 | 996 | 0.1978 | [0.1735; 0.2239] | 0.4% |
| Chiu et al, 2013 | 55 | 225 | 0.2444 | [0.1898; 0.3060] | 0.4% |

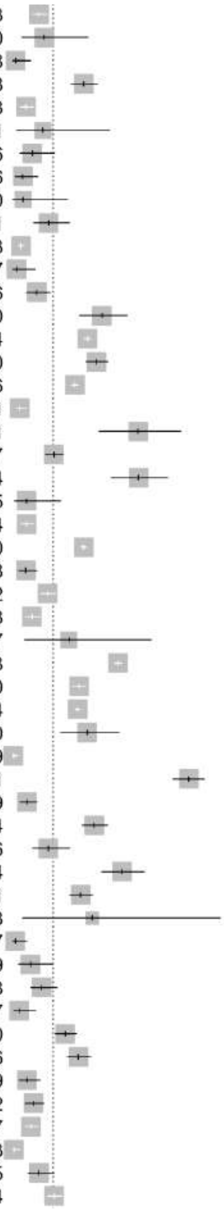

| | | | | | |
|---|---|---|---|---|---|
| Arif et al, 1994 | 55 | 663 | 0.0830 | [0.0631; 0.1066] | 0.4% |
| Anita et al, 2014 | 4 | 40 | 0.1000 | [0.0279; 0.2366] | 0.4% |
| Grabow et al, 1994 | 1 | 98 | 0.0102 | [0.0003; 0.0555] | 0.4% |
| Hardtke et al, 2018 | 91 | 408 | 0.2230 | [0.1836; 0.2666] | 0.4% |
| Ataei et al, 2007 | 18 | 428 | 0.0421 | [0.0251; 0.0657] | 0.4% |
| Hesamizadeh et al, 2015 | 2 | 21 | 0.0952 | [0.0117; 0.3038] | 0.3% |
| Hickey et al, 2016 | 6 | 96 | 0.0625 | [0.0233; 0.1311] | 0.4% |
| Houcine et al, 2012 | 4 | 126 | 0.0317 | [0.0087; 0.0793] | 0.4% |
| Jahromi et al, 2013 | 1 | 30 | 0.0333 | [0.0008; 0.1722] | 0.3% |
| Adesina et al, 2009 | 16 | 141 | 0.1135 | [0.0663; 0.1777] | 0.4% |
| Bernal et al, 1995 | 26 | 993 | 0.0262 | [0.0172; 0.0381] | 0.4% |
| Pavia et al, 1998 | 1 | 77 | 0.0130 | [0.0003; 0.0702] | 0.4% |
| Raoofi et al, 2012 | 17 | 226 | 0.0752 | [0.0444; 0.1177] | 0.4% |
| Rahman et al, 2014 | 42 | 150 | 0.2800 | [0.2098; 0.3591] | 0.4% |
| Chen et al, 2012 | 1179 | 5014 | 0.2351 | [0.2235; 0.2471] | 0.4% |
| Madden et al, 2016 | 163 | 620 | 0.2629 | [0.2286; 0.2994] | 0.4% |
| Mansuy et al, 2016 | 964 | 4946 | 0.1949 | [0.1839; 0.2062] | 0.4% |
| Karetny et al, 1995 | 8 | 361 | 0.0222 | [0.0096; 0.0432] | 0.4% |
| Lucarelli et al, 2016 | 24 | 61 | 0.3934 | [0.2707; 0.5269] | 0.4% |
| Lange et al, 2016 | 75 | 577 | 0.1300 | [0.1036; 0.1602] | 0.4% |
| Liang et al, 2014 | 49 | 124 | 0.3952 | [0.3086; 0.4869] | 0.4% |
| Marcantonio et al, 2018 | 2 | 45 | 0.0444 | [0.0054; 0.1515] | 0.4% |
| Lanini et al, 2015 | 18 | 414 | 0.0435 | [0.0260; 0.0678] | 0.4% |
| Jia et al, 2014 | 1751 | 7870 | 0.2225 | [0.2133; 0.2318] | 0.4% |
| Kaufmann et al, 2011 | 9 | 218 | 0.0413 | [0.0190; 0.0769] | 0.4% |
| Li et al, 2014 | 77 | 702 | 0.1097 | [0.0875; 0.1352] | 0.4% |
| Juhl et al, 2014 | 26 | 428 | 0.0607 | [0.0401; 0.0877] | 0.4% |
| Nagler et al, 2014 | 3 | 17 | 0.1765 | [0.0380; 0.4343] | 0.3% |
| Liu et al, 2019 | 2285 | 6903 | 0.3310 | [0.3199; 0.3423] | 0.4% |
| Mahrt et al, 2018 | 312 | 1500 | 0.2080 | [0.1877; 0.2294] | 0.4% |
| Kuniholm et al, 2009 | 2065 | 10124 | 0.2040 | [0.1962; 0.2120] | 0.4% |
| Krumbholz et al, 2011 | 21 | 90 | 0.2333 | [0.1506; 0.3343] | 0.4% |
| Jeggli et al, 2015 | 1 | 349 | 0.0029 | [0.0001; 0.0159] | 0.4% |
| Kang et al, 2017 | 227 | 411 | 0.5523 | [0.5028; 0.6011] | 0.4% |
| Matins et al, 2014 | 12 | 269 | 0.0446 | [0.0233; 0.0766] | 0.4% |
| Jahanbakhsh et al, 2017 | 124 | 484 | 0.2562 | [0.2179; 0.2975] | 0.4% |
| Rapicetta et al,1999 | 14 | 126 | 0.1111 | [0.0621; 0.1794] | 0.4% |
| Esquivel et al, 2014 | 70 | 204 | 0.3431 | [0.2783; 0.4127] | 0.4% |
| Cui et al, 2016 | 111 | 521 | 0.2131 | [0.1786; 0.2508] | 0.4% |
| Schielke et al, 2015 | 2 | 8 | 0.2500 | [0.0319; 0.6509] | 0.2% |
| Meader et al, 2008 | 1 | 117 | 0.0085 | [0.0002; 0.0467] | 0.4% |
| Whiters et al, 2002 | 5 | 89 | 0.0562 | [0.0185; 0.1263] | 0.4% |
| Widasari et al, 2013 | 19 | 208 | 0.0913 | [0.0559; 0.1390] | 0.4% |
| Galiana et al, 2008 | 2 | 97 | 0.0206 | [0.0025; 0.0725] | 0.4% |
| Cheng et al, 2007 | 84 | 510 | 0.1647 | [0.1336; 0.1998] | 0.4% |
| Cong et al, 2014 | 98 | 476 | 0.2059 | [0.1704; 0.2450] | 0.4% |
| Bayhan et al, 2016 | 8 | 179 | 0.0447 | [0.0195; 0.0862] | 0.4% |
| Achwan et al, 2007 | 21 | 322 | 0.0652 | [0.0408; 0.0980] | 0.4% |
| Buti et al, 2008 | 35 | 607 | 0.0577 | [0.0405; 0.0793] | 0.4% |
| Davaalkham et al, 2009 | 1 | 223 | 0.0045 | [0.0001; 0.0247] | 0.4% |
| Ekanem et al, 2015 | 17 | 205 | 0.0829 | [0.0491; 0.1295] | 0.4% |
| Meng et al, 2015 | 103 | 784 | 0.1314 | [0.1085; 0.1571] | 0.4% |

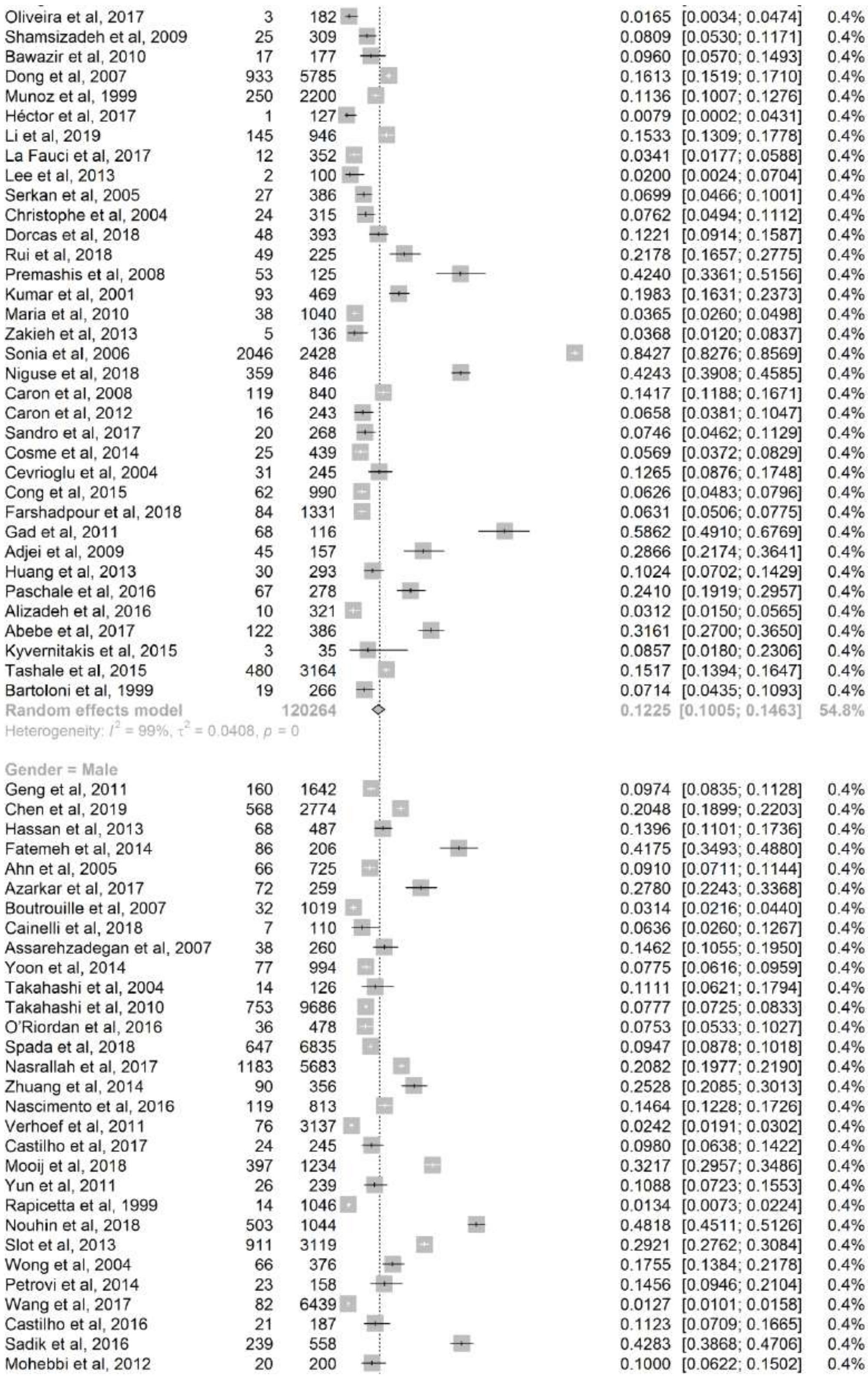

Oliveira et al, 2017 3 182 0.0165 [0.0034; 0.0474] 0.4%
Shamsizadeh et al, 2009 25 309 0.0809 [0.0530; 0.1171] 0.4%
Bawazir et al, 2010 17 177 0.0960 [0.0570; 0.1493] 0.4%
Dong et al, 2007 933 5785 0.1613 [0.1519; 0.1710] 0.4%
Munoz et al, 1999 250 2200 0.1136 [0.1007; 0.1276] 0.4%
Héctor et al, 2017 1 127 0.0079 [0.0002; 0.0431] 0.4%
Li et al, 2019 145 946 0.1533 [0.1309; 0.1778] 0.4%
La Fauci et al, 2017 12 352 0.0341 [0.0177; 0.0588] 0.4%
Lee et al, 2013 2 100 0.0200 [0.0024; 0.0704] 0.4%
Serkan et al, 2005 27 386 0.0699 [0.0466; 0.1001] 0.4%
Christophe et al, 2004 24 315 0.0762 [0.0494; 0.1112] 0.4%
Dorcas et al, 2018 48 393 0.1221 [0.0914; 0.1587] 0.4%
Rui et al, 2018 49 225 0.2178 [0.1657; 0.2775] 0.4%
Premashis et al, 2008 53 125 0.4240 [0.3361; 0.5156] 0.4%
Kumar et al, 2001 93 469 0.1983 [0.1631; 0.2373] 0.4%
Maria et al, 2010 38 1040 0.0365 [0.0260; 0.0498] 0.4%
Zakieh et al, 2013 5 136 0.0368 [0.0120; 0.0837] 0.4%
Sonia et al, 2006 2046 2428 0.8427 [0.8276; 0.8569] 0.4%
Niguse et al, 2018 359 846 0.4243 [0.3908; 0.4585] 0.4%
Caron et al, 2008 119 840 0.1417 [0.1188; 0.1671] 0.4%
Caron et al, 2012 16 243 0.0658 [0.0381; 0.1047] 0.4%
Sandro et al, 2017 20 268 0.0746 [0.0462; 0.1129] 0.4%
Cosme et al, 2014 25 439 0.0569 [0.0372; 0.0829] 0.4%
Cevrioglu et al, 2004 31 245 0.1265 [0.0876; 0.1748] 0.4%
Cong et al, 2015 62 990 0.0626 [0.0483; 0.0796] 0.4%
Farshadpour et al, 2018 84 1331 0.0631 [0.0506; 0.0775] 0.4%
Gad et al, 2011 68 116 0.5862 [0.4910; 0.6769] 0.4%
Adjei et al, 2009 45 157 0.2866 [0.2174; 0.3641] 0.4%
Huang et al, 2013 30 293 0.1024 [0.0702; 0.1429] 0.4%
Paschale et al, 2016 67 278 0.2410 [0.1919; 0.2957] 0.4%
Alizadeh et al, 2016 10 321 0.0312 [0.0150; 0.0565] 0.4%
Abebe et al, 2017 122 386 0.3161 [0.2700; 0.3650] 0.4%
Kyvernitakis et al, 2015 3 35 0.0857 [0.0180; 0.2306] 0.4%
Tashale et al, 2015 480 3164 0.1517 [0.1394; 0.1647] 0.4%
Bartoloni et al, 1999 19 266 0.0714 [0.0435; 0.1093] 0.4%
Random effects model 120264 0.1225 [0.1005; 0.1463] 54.8%
Heterogeneity: $I^2$ = 99%, $\tau^2$ = 0.0408, $p$ = 0
Gender = Male
Geng et al, 2011 160 1642 0.0974 [0.0835; 0.1128] 0.4%
Chen et al, 2019 568 2774 0.2048 [0.1899; 0.2203] 0.4%
Hassan et al, 2013 68 487 0.1396 [0.1101; 0.1736] 0.4%
Fatemeh et al, 2014 86 206 0.4175 [0.3493; 0.4880] 0.4%
Ahn et al, 2005 66 725 0.0910 [0.0711; 0.1144] 0.4%
Azarkar et al, 2017 72 259 0.2780 [0.2243; 0.3368] 0.4%
Boutrouille et al, 2007 32 1019 0.0314 [0.0216; 0.0440] 0.4%
Cainelli et al, 2018 7 110 0.0636 [0.0260; 0.1267] 0.4%
Assarehzadegan et al, 2007 38 260 0.1462 [0.1055; 0.1950] 0.4%
Yoon et al, 2014 77 994 0.0775 [0.0616; 0.0959] 0.4%
Takahashi et al, 2004 14 126 0.1111 [0.0621; 0.1794] 0.4%
Takahashi et al, 2010 753 9686 0.0777 [0.0725; 0.0833] 0.4%
O'Riordan et al, 2016 36 478 0.0753 [0.0533; 0.1027] 0.4%
Spada et al, 2018 647 6835 0.0947 [0.0878; 0.1018] 0.4%
Nasrallah et al, 2017 1183 5683 0.2082 [0.1977; 0.2190] 0.4%
Zhuang et al, 2014 90 356 0.2528 [0.2085; 0.3013] 0.4%
Nascimento et al, 2016 119 813 0.1464 [0.1228; 0.1726] 0.4%
Verhoef et al, 2011 76 3137 0.0242 [0.0191; 0.0302] 0.4%
Castilho et al, 2017 24 245 0.0980 [0.0638; 0.1422] 0.4%
Mooij et al, 2018 397 1234 0.3217 [0.2957; 0.3486] 0.4%
Yun et al, 2011 26 239 0.1088 [0.0723; 0.1553] 0.4%
Rapicetta et al, 1999 14 1046 0.0134 [0.0073; 0.0224] 0.4%
Nouhin et al, 2018 503 1044 0.4818 [0.4511; 0.5126] 0.4%
Slot et al, 2013 911 3119 0.2921 [0.2762; 0.3084] 0.4%
Wong et al, 2004 66 376 0.1755 [0.1384; 0.2178] 0.4%
Petrovi et al, 2014 23 158 0.1456 [0.0946; 0.2104] 0.4%
Wang et al, 2017 82 6439 0.0127 [0.0101; 0.0158] 0.4%
Castilho et al, 2016 21 187 0.1123 [0.0709; 0.1665] 0.4%
Sadik et al, 2016 239 558 0.4283 [0.3868; 0.4706] 0.4%
Mohebbi et al, 2012 20 200 0.1000 [0.0622; 0.1502] 0.4%

| | | | | | |
|---|---|---|---|---|---|
| Thom et al, 2017 | 54 | 879 | 0.0614 | [0.0465; 0.0794] | 0.4% |
| Mor et al, 2015 | 38 | 394 | 0.0964 | [0.0692; 0.1300] | 0.4% |
| Villalba et al, 2014 | 18 | 202 | 0.0891 | [0.0537; 0.1372] | 0.4% |
| Mohammad et al, 2017 | 67 | 247 | 0.2713 | [0.2168; 0.3313] | 0.4% |
| Yamada et al, 2015 | 79 | 360 | 0.2194 | [0.1778; 0.2658] | 0.4% |
| Vitral et al, 2014 | 26 | 184 | 0.1413 | [0.0944; 0.2002] | 0.4% |
| Santos et al, 2002 | 10 | 174 | 0.0575 | [0.0279; 0.1032] | 0.4% |
| Pittaras et al, 2014 | 23 | 216 | 0.1065 | [0.0687; 0.1555] | 0.4% |
| Bortoliero et al, 2006 | 15 | 590 | 0.0254 | [0.0143; 0.0416] | 0.4% |
| Hewitt et al, 2018 | 43 | 483 | 0.0890 | [0.0652; 0.1180] | 0.4% |
| Fukuda et al, 2007 | 147 | 2863 | 0.0513 | [0.0435; 0.0601] | 0.4% |
| Feng et al, 2018 | 588 | 800 | 0.7350 | [0.7030; 0.7653] | 0.4% |
| Fearon et al, 2017 | 146 | 2070 | 0.0705 | [0.0599; 0.0824] | 0.4% |
| Cleland et al, 2013 | 32 | 845 | 0.0379 | [0.0260; 0.0530] | 0.4% |
| Cai et al, 2016 | 169 | 846 | 0.1998 | [0.1733; 0.2283] | 0.4% |
| Chiu et al, 2013 | 74 | 225 | 0.3289 | [0.2679; 0.3945] | 0.4% |
| Arif et al, 1994 | 105 | 755 | 0.1391 | [0.1152; 0.1658] | 0.4% |
| Anita et al, 2014 | 6 | 38 | 0.1579 | [0.0602; 0.3125] | 0.4% |
| Grabow et al, 1994 | 5 | 129 | 0.0388 | [0.0127; 0.0881] | 0.4% |
| Ataei et al, 2007 | 13 | 388 | 0.0335 | [0.0180; 0.0566] | 0.4% |
| Hesamizadeh et al, 2015 | 43 | 493 | 0.0872 | [0.0638; 0.1157] | 0.4% |
| Hickey et al, 2016 | 9 | 102 | 0.0882 | [0.0411; 0.1609] | 0.4% |
| Houcine et al, 2012 | 33 | 561 | 0.0588 | [0.0408; 0.0816] | 0.4% |
| Jahromi et al, 2013 | 25 | 447 | 0.0559 | [0.0365; 0.0815] | 0.4% |
| Adesina et al, 2009 | 9 | 45 | 0.2000 | [0.0958; 0.3460] | 0.4% |
| Bernal et al, 1995 | 10 | 764 | 0.0131 | [0.0063; 0.0239] | 0.4% |
| Pavia et al, 1998 | 2 | 283 | 0.0071 | [0.0009; 0.0253] | 0.4% |
| Raoofi et al, 2012 | 14 | 174 | 0.0805 | [0.0447; 0.1313] | 0.4% |
| Rahman et al, 2014 | 48 | 150 | 0.3200 | [0.2463; 0.4010] | 0.4% |
| Chen et al, 2012 | 1614 | 8015 | 0.2014 | [0.1926; 0.2103] | 0.4% |
| Madden et al, 2016 | 161 | 541 | 0.2976 | [0.2593; 0.3381] | 0.4% |
| Mansuy et al, 2016 | 1407 | 5623 | 0.2502 | [0.2389; 0.2618] | 0.4% |
| Karetny et al, 1995 | 5 | 75 | 0.0667 | [0.0220; 0.1488] | 0.4% |
| Lucarelli et al, 2016 | 129 | 252 | 0.5119 | [0.4484; 0.5751] | 0.4% |
| Lange et al, 2016 | 102 | 686 | 0.1487 | [0.1229; 0.1775] | 0.4% |
| Liang et al, 2014 | 80 | 183 | 0.4372 | [0.3641; 0.5123] | 0.4% |
| Marcantonio et al, 2018 | 8 | 153 | 0.0523 | [0.0228; 0.1004] | 0.4% |
| Lanini et al, 2015 | 42 | 702 | 0.0598 | [0.0435; 0.0800] | 0.4% |
| Jia et al, 2014 | 1970 | 7996 | 0.2464 | [0.2370; 0.2560] | 0.4% |
| Kaufmann et al, 2011 | 18 | 332 | 0.0542 | [0.0324; 0.0843] | 0.4% |
| Li et al, 2014 | 151 | 936 | 0.1613 | [0.1383; 0.1865] | 0.4% |
| Juhl et al, 2014 | 43 | 591 | 0.0728 | [0.0532; 0.0968] | 0.4% |
| Nagler et al, 2014 | 139 | 980 | 0.1418 | [0.1206; 0.1653] | 0.4% |
| Liu et al, 2019 | 1136 | 3353 | 0.3388 | [0.3228; 0.3551] | 0.4% |
| Mahrt et al, 2018 | 367 | 1500 | 0.2447 | [0.2231; 0.2672] | 0.4% |
| Kuniholm et al, 2009 | 1851 | 8571 | 0.2160 | [0.2073; 0.2248] | 0.4% |
| Krumbholz et al, 2011 | 27 | 132 | 0.2045 | [0.1393; 0.2835] | 0.4% |
| Jeggli et al, 2015 | 29 | 429 | 0.0676 | [0.0457; 0.0956] | 0.4% |
| Kang et al, 2017 | 103 | 789 | 0.1305 | [0.1078; 0.1561] | 0.4% |
| Korzeniewski et al, 2018 | 14 | 224 | 0.0625 | [0.0346; 0.1026] | 0.4% |
| Li et al, 2006 | 1576 | 3844 | 0.4100 | [0.3944; 0.4257] | 0.4% |
| Labrique et al, 2009 | 121 | 614 | 0.1971 | [0.1663; 0.2308] | 0.4% |
| Matins et al, 2014 | 12 | 162 | 0.0741 | [0.0389; 0.1258] | 0.4% |
| Jahanbakhsh et al, 2017 | 12 | 78 | 0.1538 | [0.0821; 0.2533] | 0.4% |
| Rapicetta et al,1999 | 99 | 847 | 0.1169 | [0.0960; 0.1405] | 0.4% |
| Esquivel et al, 2014 | 30 | 69 | 0.4348 | [0.3158; 0.5596] | 0.4% |
| Cui et al, 2016 | 117 | 507 | 0.2308 | [0.1948; 0.2699] | 0.4% |
| Schielke et al, 2015 | 24 | 118 | 0.2034 | [0.1349; 0.2873] | 0.4% |
| Meader et al, 2008 | 9 | 296 | 0.0304 | [0.0140; 0.0569] | 0.4% |
| Whiters et al, 2002 | 16 | 201 | 0.0796 | [0.0462; 0.1261] | 0.4% |
| Widasari et al, 2013 | 19 | 282 | 0.0674 | [0.0410; 0.1032] | 0.4% |
| Galiana et al, 2008 | 21 | 101 | 0.2079 | [0.1336; 0.3001] | 0.4% |
| Cheng et al, 2007 | 25 | 244 | 0.1025 | [0.0674; 0.1475] | 0.4% |
| Cong et al, 2014 | 110 | 414 | 0.2657 | [0.2238; 0.3110] | 0.4% |
| Bayhan et al, 2016 | 9 | 229 | 0.0393 | [0.0181; 0.0733] | 0.4% |
| Achwan et al, 2007 | 13 | 259 | 0.0502 | [0.0270; 0.0843] | 0.4% |
| Buti et al, 2008 | 22 | 642 | 0.0343 | [0.0216; 0.0514] | 0.4% |
| Davaalkham et al, 2009 | 2 | 216 | 0.0093 | [0.0011; 0.0330] | 0.4% |
| Ekanem et al, 2015 | 14 | 197 | 0.0711 | [0.0394; 0.1164] | 0.4% |
| Meng et al, 2015 | 121 | 716 | 0.1690 | [0.1423; 0.1985] | 0.4% |
| Oliveira et al, 2017 | 1 | 170 | 0.0059 | [0.0001; 0.0323] | 0.4% |
| Shamsizadeh et al, 2009 | 23 | 257 | 0.0895 | [0.0576; 0.1313] | 0.4% |
| Bawazir et al, 2010 | 21 | 179 | 0.1173 | [0.0741; 0.1737] | 0.4% |
| Dong et al, 2007 | 1140 | 6267 | 0.1819 | [0.1724; 0.1917] | 0.4% |
| Munoz et al, 1999 | 124 | 1349 | 0.0919 | [0.0770; 0.1086] | 0.4% |
| Kyvernitakis et al, 2015 | 9 | 80 | 0.1125 | [0.0528; 0.2028] | 0.4% |
| Tashale et al, 2015 | 520 | 2802 | 0.1856 | [0.1713; 0.2005] | 0.4% |
| Bartoloni et al, 1999 | 17 | 224 | 0.0759 | [0.0448; 0.1187] | 0.4% |
| Random effects model | | 129569 | 0.1339 | [0.1134; 0.1559] | 45.2% |
| Heterogeneity: $I^2 = 99\%$, $\tau^2 = 0.0264$, $p = 0$ | | | | | |
| **Random effects model** | | **249833** | **0.1276** | **[0.1125; 0.1435]** | **100.0%** |
| Heterogeneity: $I^2 = 99\%$, $\tau^2 = 0.0327$, $p = 0$ | | | | | |
| Residual heterogeneity: $I^2 = 99\%$, $p = 0$ | | | | | |

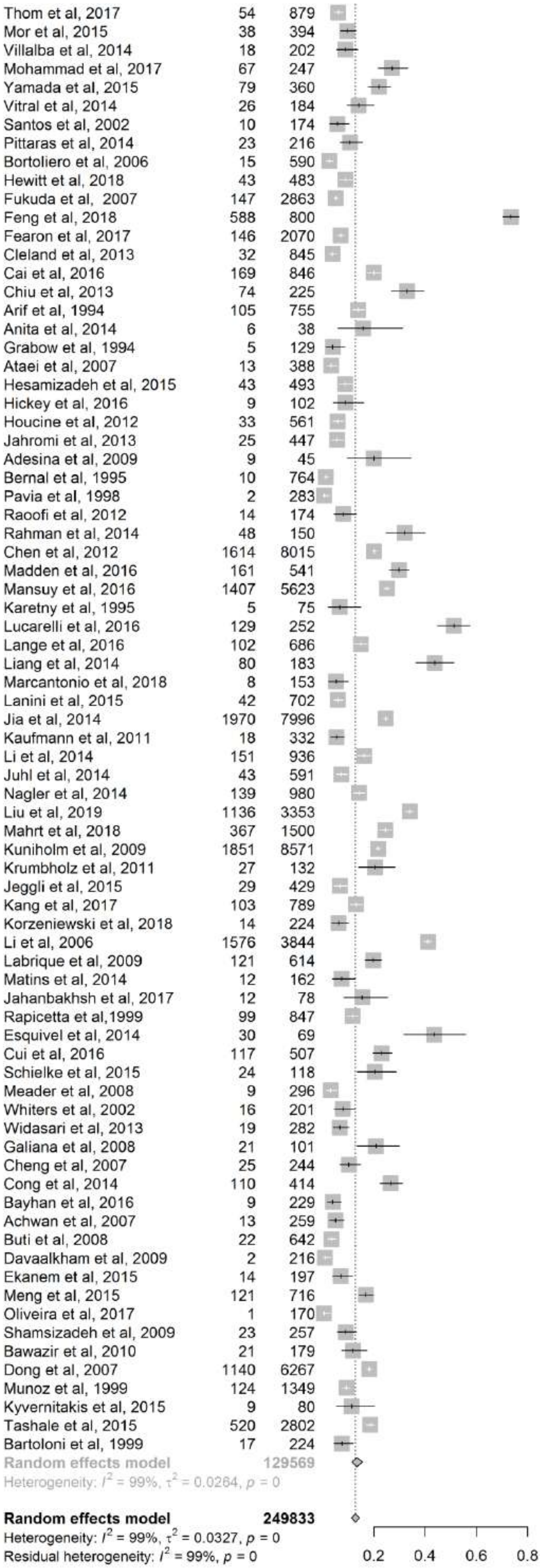

**Figure S10. Forest plot of estimated pooled anti-HEV IgG seroprevalence among general population based on high income countries, upper-middle income countries, lower middle income countries, and low income countries.**

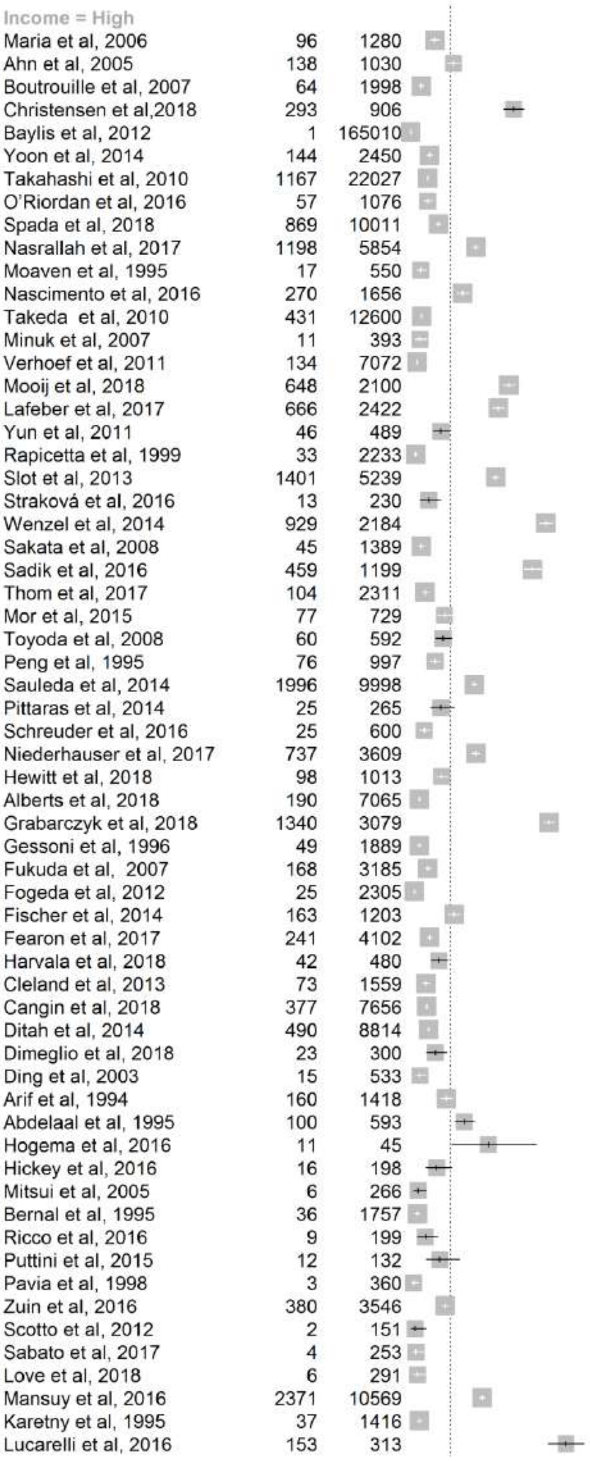

| Study | Events | Total | Proportion | 95%-CI | Weight |
|---|---|---|---|---|---|
| Income = High | | | | | |
| Maria et al, 2006 | 96 | 1280 | 0.0750 | [0.0612; 0.0908] | 0.4% |
| Ahn et al, 2005 | 138 | 1030 | 0.1340 | [0.1138; 0.1563] | 0.3% |
| Boutrouille et al, 2007 | 64 | 1998 | 0.0320 | [0.0248; 0.0407] | 0.4% |
| Christensen et al,2018 | 293 | 906 | 0.3234 | [0.2930; 0.3549] | 0.3% |
| Baylis et al, 2012 | 1 | 165010 | 0.0000 | [0.0000; 0.0000] | 0.4% |
| Yoon et al, 2014 | 144 | 2450 | 0.0588 | [0.0498; 0.0688] | 0.4% |
| Takahashi et al, 2010 | 1167 | 22027 | 0.0530 | [0.0501; 0.0560] | 0.4% |
| O'Riordan et al, 2016 | 57 | 1076 | 0.0530 | [0.0404; 0.0681] | 0.3% |
| Spada et al, 2018 | 869 | 10011 | 0.0868 | [0.0814; 0.0925] | 0.4% |
| Nasrallah et al, 2017 | 1198 | 5854 | 0.2046 | [0.1944; 0.2152] | 0.4% |
| Moaven et al, 1995 | 17 | 550 | 0.0309 | [0.0181; 0.0490] | 0.3% |
| Nascimento et al, 2016 | 270 | 1656 | 0.1630 | [0.1456; 0.1817] | 0.4% |
| Takeda et al, 2010 | 431 | 12600 | 0.0342 | [0.0311; 0.0375] | 0.4% |
| Minuk et al, 2007 | 11 | 393 | 0.0280 | [0.0141; 0.0495] | 0.3% |
| Verhoef et al, 2011 | 134 | 7072 | 0.0189 | [0.0159; 0.0224] | 0.4% |
| Mooij et al, 2018 | 648 | 2100 | 0.3086 | [0.2889; 0.3288] | 0.4% |
| Lafeber et al, 2017 | 666 | 2422 | 0.2750 | [0.2573; 0.2932] | 0.4% |
| Yun et al, 2011 | 46 | 489 | 0.0941 | [0.0697; 0.1235] | 0.3% |
| Rapicetta et al, 1999 | 33 | 2233 | 0.0148 | [0.0102; 0.0207] | 0.4% |
| Slot et al, 2013 | 1401 | 5239 | 0.2674 | [0.2555; 0.2796] | 0.4% |
| Straková et al, 2016 | 13 | 230 | 0.0565 | [0.0304; 0.0947] | 0.3% |
| Wenzel et al, 2014 | 929 | 2184 | 0.4254 | [0.4045; 0.4464] | 0.4% |
| Sakata et al, 2008 | 45 | 1389 | 0.0324 | [0.0237; 0.0431] | 0.4% |
| Sadik et al, 2016 | 459 | 1199 | 0.3828 | [0.3552; 0.4110] | 0.4% |
| Thom et al, 2017 | 104 | 2311 | 0.0450 | [0.0369; 0.0543] | 0.4% |
| Mor et al, 2015 | 77 | 729 | 0.1056 | [0.0843; 0.1302] | 0.3% |
| Toyoda et al, 2008 | 60 | 592 | 0.1014 | [0.0782; 0.1285] | 0.3% |
| Peng et al, 1995 | 76 | 997 | 0.0762 | [0.0605; 0.0945] | 0.3% |
| Sauleda et al, 2014 | 1996 | 9998 | 0.1996 | [0.1918; 0.2076] | 0.4% |
| Pittaras et al, 2014 | 25 | 265 | 0.0943 | [0.0620; 0.1361] | 0.3% |
| Schreuder et al, 2016 | 25 | 600 | 0.0417 | [0.0271; 0.0609] | 0.3% |
| Niederhauser et al, 2017 | 737 | 3609 | 0.2042 | [0.1912; 0.2177] | 0.4% |
| Hewitt et al, 2018 | 98 | 1013 | 0.0967 | [0.0792; 0.1166] | 0.3% |
| Alberts et al, 2018 | 190 | 7065 | 0.0269 | [0.0232; 0.0309] | 0.4% |
| Grabarczyk et al, 2018 | 1340 | 3079 | 0.4352 | [0.4176; 0.4529] | 0.4% |
| Gessoni et al, 1996 | 49 | 1889 | 0.0259 | [0.0193; 0.0341] | 0.4% |
| Fukuda et al, 2007 | 168 | 3185 | 0.0527 | [0.0452; 0.0611] | 0.4% |
| Fogeda et al, 2012 | 25 | 2305 | 0.0108 | [0.0070; 0.0160] | 0.4% |
| Fischer et al, 2014 | 163 | 1203 | 0.1355 | [0.1166; 0.1561] | 0.4% |
| Fearon et al, 2017 | 241 | 4102 | 0.0588 | [0.0517; 0.0664] | 0.4% |
| Harvala et al, 2018 | 42 | 480 | 0.0875 | [0.0638; 0.1164] | 0.3% |
| Cleland et al, 2013 | 73 | 1559 | 0.0468 | [0.0369; 0.0585] | 0.4% |
| Cangin et al, 2018 | 377 | 7656 | 0.0492 | [0.0445; 0.0543] | 0.4% |
| Ditah et al, 2014 | 490 | 8814 | 0.0556 | [0.0509; 0.0606] | 0.4% |
| Dimeglio et al, 2018 | 23 | 300 | 0.0767 | [0.0492; 0.1128] | 0.3% |
| Ding et al, 2003 | 15 | 533 | 0.0281 | [0.0158; 0.0460] | 0.3% |
| Arif et al, 1994 | 160 | 1418 | 0.1128 | [0.0968; 0.1305] | 0.4% |
| Abdelaal et al, 1995 | 100 | 593 | 0.1686 | [0.1394; 0.2012] | 0.3% |
| Hogema et al, 2016 | 11 | 45 | 0.2444 | [0.1288; 0.3954] | 0.3% |
| Hickey et al, 2016 | 16 | 198 | 0.0808 | [0.0469; 0.1279] | 0.3% |
| Mitsui et al, 2005 | 6 | 266 | 0.0226 | [0.0083; 0.0484] | 0.3% |
| Bernal et al, 1995 | 36 | 1757 | 0.0205 | [0.0144; 0.0283] | 0.4% |
| Ricco et al, 2016 | 9 | 199 | 0.0452 | [0.0209; 0.0841] | 0.3% |
| Puttini et al, 2015 | 12 | 132 | 0.0909 | [0.0479; 0.1534] | 0.3% |
| Pavia et al, 1998 | 3 | 360 | 0.0083 | [0.0017; 0.0242] | 0.3% |
| Zuin et al, 2016 | 380 | 3546 | 0.1072 | [0.0972; 0.1178] | 0.4% |
| Scotto et al, 2012 | 2 | 151 | 0.0132 | [0.0016; 0.0470] | 0.3% |
| Sabato et al, 2017 | 4 | 253 | 0.0158 | [0.0043; 0.0400] | 0.3% |
| Love et al, 2018 | 6 | 291 | 0.0206 | [0.0076; 0.0443] | 0.3% |
| Mansuy et al, 2016 | 2371 | 10569 | 0.2243 | [0.2164; 0.2324] | 0.4% |
| Karetny et al, 1995 | 37 | 1416 | 0.0261 | [0.0185; 0.0358] | 0.4% |
| Lucarelli et al, 2016 | 153 | 313 | 0.4888 | [0.4322; 0.5457] | 0.3% |

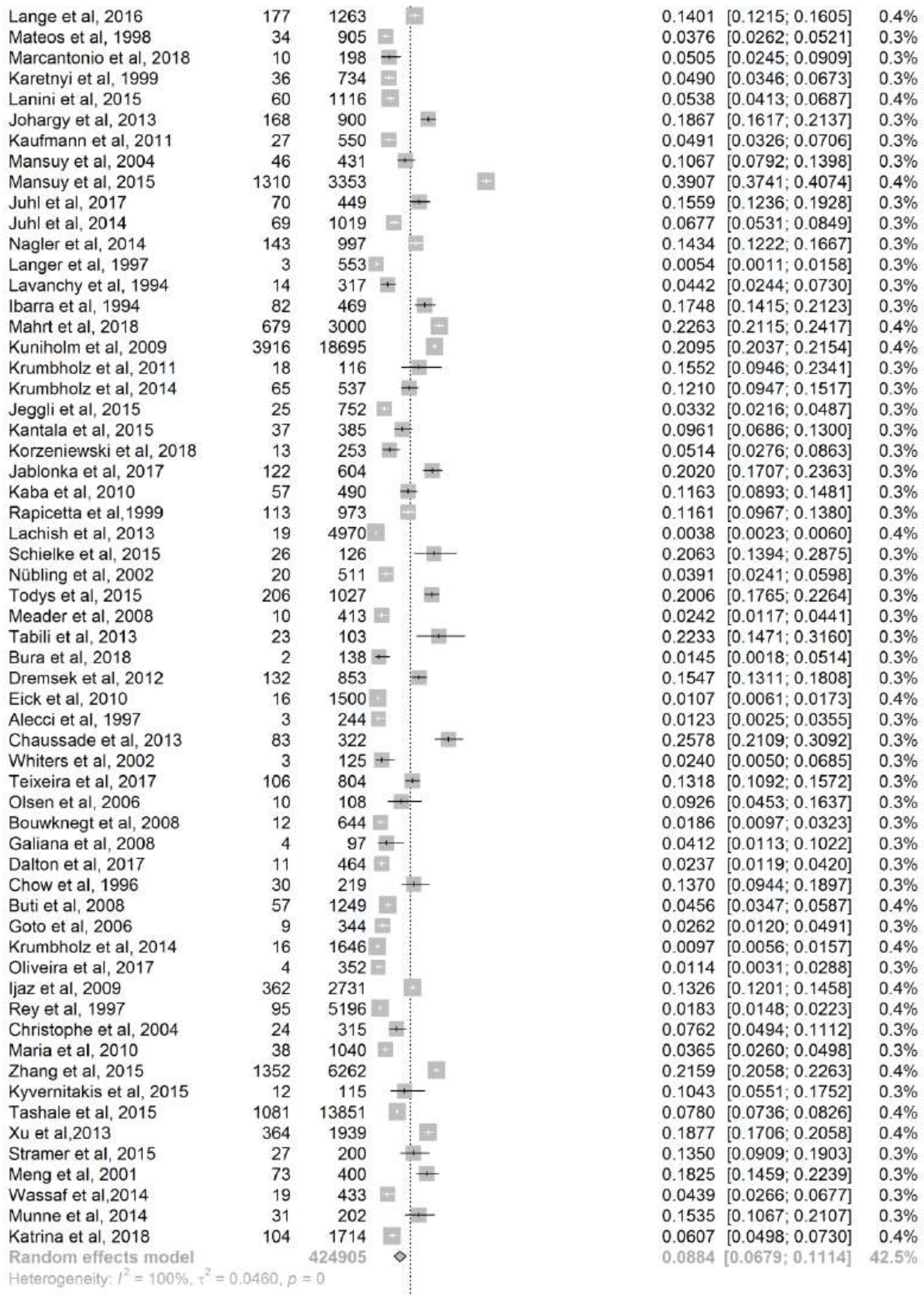
Lange et al, 2016 177 1263 0.1401 [0.1215; 0.1605] 0.4%
Mateos et al, 1998 34 905 0.0376 [0.0262; 0.0521] 0.3%
Marcantonio et al, 2018 10 198 0.0505 [0.0245; 0.0909] 0.3%
Karetnyi et al, 1999 36 734 0.0490 [0.0346; 0.0673] 0.3%
Lanini et al, 2015 60 1116 0.0538 [0.0413; 0.0687] 0.4%
Johargy et al, 2013 168 900 0.1867 [0.1617; 0.2137] 0.3%
Kaufmann et al, 2011 27 550 0.0491 [0.0326; 0.0706] 0.3%
Mansuy et al, 2004 46 431 0.1067 [0.0792; 0.1398] 0.3%
Mansuy et al, 2015 1310 3353 0.3907 [0.3741; 0.4074] 0.4%
Juhl et al, 2017 70 449 0.1559 [0.1236; 0.1928] 0.3%
Juhl et al, 2014 69 1019 0.0677 [0.0531; 0.0849] 0.3%
Nagler et al, 2014 143 997 0.1434 [0.1222; 0.1667] 0.3%
Langer et al, 1997 3 553 0.0054 [0.0011; 0.0158] 0.3%
Lavanchy et al, 1994 14 317 0.0442 [0.0244; 0.0730] 0.3%
Ibarra et al, 1994 82 469 0.1748 [0.1415; 0.2123] 0.3%
Mahrt et al, 2018 679 3000 0.2263 [0.2115; 0.2417] 0.4%
Kuniholm et al, 2009 3916 18695 0.2095 [0.2037; 0.2154] 0.4%
Krumbholz et al, 2011 18 116 0.1552 [0.0946; 0.2341] 0.3%
Krumbholz et al, 2014 65 537 0.1210 [0.0947; 0.1517] 0.3%
Jeggli et al, 2015 25 752 0.0332 [0.0216; 0.0487] 0.3%
Kantala et al, 2015 37 385 0.0961 [0.0686; 0.1300] 0.3%
Korzeniewski et al, 2018 13 253 0.0514 [0.0276; 0.0863] 0.3%
Jablonka et al, 2017 122 604 0.2020 [0.1707; 0.2363] 0.3%
Kaba et al, 2010 57 490 0.1163 [0.0893; 0.1481] 0.3%
Rapicetta et al,1999 113 973 0.1161 [0.0967; 0.1380] 0.3%
Lachish et al, 2013 19 4970 0.0038 [0.0023; 0.0060] 0.4%
Schielke et al, 2015 26 126 0.2063 [0.1394; 0.2875] 0.3%
Nübling et al, 2002 20 511 0.0391 [0.0241; 0.0598] 0.3%
Todys et al, 2015 206 1027 0.2006 [0.1765; 0.2264] 0.3%
Meader et al, 2008 10 413 0.0242 [0.0117; 0.0441] 0.3%
Tabili et al, 2013 23 103 0.2233 [0.1471; 0.3160] 0.3%
Bura et al, 2018 2 138 0.0145 [0.0018; 0.0514] 0.3%
Dremsek et al, 2012 132 853 0.1547 [0.1311; 0.1808] 0.3%
Eick et al, 2010 16 1500 0.0107 [0.0061; 0.0173] 0.4%
Alecci et al, 1997 3 244 0.0123 [0.0025; 0.0355] 0.3%
Chaussade et al, 2013 83 322 0.2578 [0.2109; 0.3092] 0.3%
Whiters et al, 2002 3 125 0.0240 [0.0050; 0.0685] 0.3%
Teixeira et al, 2017 106 804 0.1318 [0.1092; 0.1572] 0.3%
Olsen et al, 2006 10 108 0.0926 [0.0453; 0.1637] 0.3%
Bouwknegt et al, 2008 12 644 0.0186 [0.0097; 0.0323] 0.3%
Galiana et al, 2008 4 97 0.0412 [0.0113; 0.1022] 0.3%
Dalton et al, 2017 11 464 0.0237 [0.0119; 0.0420] 0.3%
Chow et al, 1996 30 219 0.1370 [0.0944; 0.1897] 0.3%
Buti et al, 2008 57 1249 0.0456 [0.0347; 0.0587] 0.4%
Goto et al, 2006 9 344 0.0262 [0.0120; 0.0491] 0.3%
Krumbholz et al, 2014 16 1646 0.0097 [0.0056; 0.0157] 0.4%
Oliveira et al, 2017 4 352 0.0114 [0.0031; 0.0288] 0.3%
Ijaz et al, 2009 362 2731 0.1326 [0.1201; 0.1458] 0.4%
Rey et al, 1997 95 5196 0.0183 [0.0148; 0.0223] 0.4%
Christophe et al, 2004 24 315 0.0762 [0.0494; 0.1112] 0.3%
Maria et al, 2010 38 1040 0.0365 [0.0260; 0.0498] 0.3%
Zhang et al, 2015 1352 6262 0.2159 [0.2058; 0.2263] 0.4%
Kyvernitakis et al, 2015 12 115 0.1043 [0.0551; 0.1752] 0.3%
Tashale et al, 2015 1081 13851 0.0780 [0.0736; 0.0826] 0.4%
Xu et al,2013 364 1939 0.1877 [0.1706; 0.2058] 0.4%
Stramer et al, 2015 27 200 0.1350 [0.0909; 0.1903] 0.3%
Meng et al, 2001 73 400 0.1825 [0.1459; 0.2239] 0.3%
Wassaf et al,2014 19 433 0.0439 [0.0266; 0.0677] 0.3%
Munne et al, 2014 31 202 0.1535 [0.1067; 0.2107] 0.3%
Katrina et al, 2018 104 1714 0.0607 [0.0498; 0.0730] 0.4%
Random effects model 424905 0.0884 [0.0679; 0.1114] 42.5%
Heterogeneity: I^2 = 100%, τ^2 = 0.0460, p = 0

| Study | Events | Total | Proportion | 95% CI | Weight |
|---|---|---|---|---|---|
| **Income = Low** | | | | | |
| Edward et al, 1997 | 571 | 757 | 0.7543 | [0.7220; 0.7846] | 0.3% |
| Shrestha et al, 2016 | 773 | 1845 | 0.4190 | [0.3963; 0.4419] | 0.4% |
| Miller et al, 1998 | 1 | 403 | 0.0025 | [0.0001; 0.0137] | 0.3% |
| Azman et al, 2017 | 154 | 206 | 0.7476 | [0.6825; 0.8054] | 0.3% |
| Bawazir et al, 2010 | 38 | 356 | 0.1067 | [0.0767; 0.1436] | 0.3% |
| Niguse et al, 2018 | 359 | 846 | 0.4243 | [0.3908; 0.4585] | 0.3% |
| Paschale et al, 2016 | 62 | 278 | 0.2230 | [0.1755; 0.2766] | 0.3% |
| Abebe et al, 2017 | 122 | 386 | 0.3161 | [0.2700; 0.3650] | 0.3% |
| Traore et al, 2012 | 56 | 178 | 0.3146 | [0.2472; 0.3883] | 0.3% |
| Aubry et al, 1997 | 18 | 129 | 0.1395 | [0.0848; 0.2115] | 0.3% |
| Taha et al, 2015 | 80 | 397 | 0.2015 | [0.1631; 0.2444] | 0.3% |
| Random effects model | | 5781 | 0.3044 | [0.1660; 0.4639] | 3.8% |
| Heterogeneity: $I^2 = 99\%$, $\tau^2 = 0.0770$, $p < 0.01$ | | | | | |
| **Income = Lower-middle** | | | | | |
| Corwin et al, 1996 | 26 | 187 | 0.1390 | [0.0929; 0.1970] | 0.3% |
| Dell'Amico et al, 2011 | 12 | 123 | 0.0976 | [0.0514; 0.1642] | 0.3% |
| Takahashi et al, 2004 | 28 | 249 | 0.1124 | [0.0760; 0.1584] | 0.3% |
| Nouhin et al, 2016 | 85 | 301 | 0.2824 | [0.2322; 0.3369] | 0.3% |
| Nouhin et al, 2018 | 829 | 2004 | 0.4137 | [0.3920; 0.4356] | 0.4% |
| Tripathy et al, 2018 | 433 | 2447 | 0.1770 | [0.1620; 0.1927] | 0.4% |
| Vivek et al, 2010 | 330 | 2279 | 0.1448 | [0.1306; 0.1599] | 0.4% |
| Yamada et al, 2015 | 160 | 868 | 0.1843 | [0.1591; 0.2118] | 0.3% |
| Amarapurkar et al, 2008 | 416 | 1163 | 0.3577 | [0.3301; 0.3860] | 0.4% |
| Houcine et al, 2012 | 37 | 687 | 0.0539 | [0.0382; 0.0735] | 0.3% |
| Adesina et al, 2009 | 25 | 186 | 0.1344 | [0.0889; 0.1920] | 0.3% |
| Rahman et al, 2014 | 90 | 300 | 0.3000 | [0.2487; 0.3553] | 0.3% |
| Kotwal et al, 2014 | 712 | 4175 | 0.1705 | [0.1592; 0.1823] | 0.4% |
| Konomi et al, 1999 | 93 | 574 | 0.1620 | [0.1328; 0.1948] | 0.3% |
| Katiyar et al, 2018 | 383 | 633 | 0.6051 | [0.5658; 0.6434] | 0.3% |
| Kamel et al, 1995 | 214 | 1259 | 0.1700 | [0.1496; 0.1919] | 0.4% |
| Khounvisith et al, 2018 | 38 | 210 | 0.1810 | [0.1313; 0.2398] | 0.3% |
| Labrique et al, 2009 | 255 | 1134 | 0.2249 | [0.2009; 0.2503] | 0.4% |
| Widasari et al, 2013 | 16 | 277 | 0.0578 | [0.0334; 0.0921] | 0.3% |
| Drobeniuc et al, 2001 | 63 | 255 | 0.2471 | [0.1954; 0.3047] | 0.3% |
| Pathak et al, 2018 | 341 | 1369 | 0.2491 | [0.2264; 0.2729] | 0.4% |
| Sheikh et al, 2002 | 164 | 273 | 0.6007 | [0.5400; 0.6593] | 0.3% |
| Aboulata et al, 2005 | 26 | 100 | 0.2600 | [0.1774; 0.3573] | 0.3% |
| Achwan et al, 2007 | 34 | 581 | 0.0585 | [0.0409; 0.0808] | 0.3% |
| Davaalkham et al, 2009 | 3 | 520 | 0.0058 | [0.0012; 0.0168] | 0.3% |
| Ekanem et al, 2015 | 31 | 402 | 0.0771 | [0.0530; 0.1077] | 0.3% |
| Gandolfo et al, 2003 | 4 | 435 | 0.0092 | [0.0025; 0.0234] | 0.3% |
| Hasan et al, 2016 | 33 | 123 | 0.2683 | [0.1924; 0.3557] | 0.3% |
| Mathur et al, 2001 | 545 | 2070 | 0.2633 | [0.2444; 0.2828] | 0.4% |
| Bira et al, 2007 | 5 | 717 | 0.0070 | [0.0023; 0.0162] | 0.3% |
| Hau et al, 1999 | 18 | 646 | 0.0279 | [0.0166; 0.0437] | 0.3% |
| Fix et al, 2000 | 4283 | 10026 | 0.4272 | [0.4175; 0.4369] | 0.4% |
| Sonia et al, 2006 | 2046 | 2428 | 0.8427 | [0.8276; 0.8569] | 0.4% |
| Gad et al, 2011 | 68 | 116 | 0.5862 | [0.4910; 0.6769] | 0.3% |
| Adjei et al, 2009 | 16 | 157 | 0.1019 | [0.0594; 0.1602] | 0.3% |
| Gambel et al, 1998 | 14 | 112 | 0.1250 | [0.0701; 0.2008] | 0.3% |
| Perez et al, 1996 | 17 | 399 | 0.0426 | [0.0250; 0.0673] | 0.3% |
| Bartoloni et al, 1999 | 36 | 490 | 0.0735 | [0.0520; 0.1003] | 0.3% |
| Leon et al, 1999 | 64 | 318 | 0.2013 | [0.1586; 0.2496] | 0.3% |
| Random effects model | | 40593 | 0.1904 | [0.1325; 0.2560] | 13.6% |
| Heterogeneity: $I^2 = 100\%$, $\tau^2 = 0.0626$, $p = 0$ | | | | | |
| **Income = Mixed** | | | | | |
| Halliday et al, 2014 | 120 | 1048 | 0.1145 | [0.0959; 0.1354] | 0.3% |
| Abe et al, 2006 | 1720 | 5233 | 0.3287 | [0.3160; 0.3416] | 0.4% |
| Izopet et al, 2015 | 1083 | 2538 | 0.4267 | [0.4074; 0.4462] | 0.4% |
| Gambel et al, 1998 | 209 | 981 | 0.2130 | [0.1878; 0.2400] | 0.3% |
| Random effects model | | 9800 | 0.2620 | [0.1542; 0.3868] | 1.4% |
| Heterogeneity: $I^2 = 99\%$, $\tau^2 = 0.0185$, $p < 0.01$ | | | | | |
| **Income = Upper-middle** | | | | | |
| Chang et al, 2009 | 522 | 2572 | 0.2030 | [0.1876; 0.2190] | 0.4% |
| Geng et al, 2011 | 538 | 2682 | 0.2006 | [0.1856; 0.2163] | 0.4% |
| Yuan et al, 2017 | 268 | 1200 | 0.2233 | [0.2001; 0.2480] | 0.4% |
| Chen et al, 2019 | 799 | 4044 | 0.1976 | [0.1854; 0.2102] | 0.4% |
| Hassan et al, 2013 | 76 | 530 | 0.1434 | [0.1147; 0.1762] | 0.3% |
| Fatemeh et al, 2014 | 235 | 510 | 0.4608 | [0.4169; 0.5051] | 0.3% |
| Guo et al, 2009 | 14610 | 44816 | 0.3260 | [0.3217; 0.3304] | 0.4% |
| Azarkar et al, 2017 | 139 | 554 | 0.2509 | [0.2153; 0.2892] | 0.3% |
| Cainelli et al, 2018 | 11 | 199 | 0.0553 | [0.0279; 0.0968] | 0.3% |
| Assarehzadegan et al, 2007 | 46 | 400 | 0.1150 | [0.0854; 0.1504] | 0.3% |
| Zhuang et al, 2014 | 113 | 486 | 0.2325 | [0.1956; 0.2727] | 0.3% |
| Castilho et al, 2017 | 49 | 500 | 0.0980 | [0.0734; 0.1275] | 0.3% |
| Olcay et al, 2003 | 57 | 910 | 0.0626 | [0.0478; 0.0804] | 0.3% |

| Study | Events | Total | Proportion | 95%-CI | Weight |
|---|---|---|---|---|---|
| Wong et al, 2004 | 176 | 934 | 0.1884 | [0.1638; 0.2150] | 0.3% |
| Meldal et al, 2013 | 11 | 239 | 0.0460 | [0.0232; 0.0809] | 0.3% |
| Petrovi et al, 2014 | 30 | 200 | 0.1500 | [0.1035; 0.2072] | 0.3% |
| Wang et al, 2017 | 2682 | 9069 | 0.2957 | [0.2864; 0.3052] | 0.4% |
| Quintana et al, 2005 | 11 | 209 | 0.0526 | [0.0266; 0.0922] | 0.3% |
| Taremi et al, 2007 | 31 | 399 | 0.0777 | [0.0534; 0.1085] | 0.3% |
| Castilho et al, 2016 | 30 | 300 | 0.1000 | [0.0685; 0.1397] | 0.3% |
| Zhu et al, 2008 | 1289 | 4654 | 0.2770 | [0.2641; 0.2901] | 0.4% |
| Mohebbi et al, 2012 | 51 | 551 | 0.0926 | [0.0697; 0.1199] | 0.3% |
| Yang et al, 2014 | 473 | 2028 | 0.2332 | [0.2150; 0.2523] | 0.4% |
| Zhu et al, 2014 | 152061 | 400162 | 0.3800 | [0.3785; 0.3815] | 0.4% |
| Villalba et al, 2014 | 47 | 469 | 0.1002 | [0.0746; 0.1310] | 0.3% |
| Mohammad et al, 2017 | 139 | 450 | 0.3089 | [0.2665; 0.3538] | 0.3% |
| Vitral et al, 2014 | 50 | 388 | 0.1289 | [0.0972; 0.1663] | 0.3% |
| Talarmin et al, 1997 | 64 | 996 | 0.0643 | [0.0498; 0.0813] | 0.3% |
| Ren et al, 2013 | 2945 | 10741 | 0.2742 | [0.2658; 0.2827] | 0.4% |
| Santos et al, 2002 | 17 | 699 | 0.0243 | [0.0142; 0.0387] | 0.3% |
| Parsa et al, 2016 | 50 | 700 | 0.0714 | [0.0535; 0.0931] | 0.3% |
| Jahromi et al, 2013 | 26 | 477 | 0.0545 | [0.0359; 0.0788] | 0.3% |
| Teoharov et al, 2014 | 67 | 741 | 0.0904 | [0.0708; 0.1134] | 0.3% |
| Bortoliero et al, 2006 | 23 | 996 | 0.0231 | [0.0147; 0.0344] | 0.3% |
| Feng et al, 2018 | 1273 | 1912 | 0.6658 | [0.6441; 0.6869] | 0.4% |
| Paula et al, 2001 | 10 | 255 | 0.0392 | [0.0190; 0.0709] | 0.3% |
| Goncales et al, 2000 | 18 | 357 | 0.0504 | [0.0302; 0.0785] | 0.3% |
| Cai et al, 2016 | 1842 | 3337 | 0.5520 | [0.5349; 0.5690] | 0.4% |
| Chiu et al, 2013 | 129 | 450 | 0.2867 | [0.2453; 0.3309] | 0.3% |
| Chan et al, 2017 | 492 | 1539 | 0.3197 | [0.2964; 0.3436] | 0.4% |
| Anita et al, 2014 | 22 | 148 | 0.1486 | [0.0956; 0.2164] | 0.3% |
| Grabow et al, 1994 | 16 | 782 | 0.0205 | [0.0117; 0.0330] | 0.3% |
| Hardtke et al, 2018 | 91 | 408 | 0.2230 | [0.1836; 0.2666] | 0.3% |
| Ataei et al, 2007 | 31 | 816 | 0.0380 | [0.0260; 0.0535] | 0.3% |
| Hesamizadeh et al, 2015 | 45 | 514 | 0.0875 | [0.0646; 0.1154] | 0.3% |
| Jahromi et al, 2013 | 26 | 477 | 0.0545 | [0.0359; 0.0788] | 0.3% |
| Sepanlou et al, 2010 | 105 | 1423 | 0.0738 | [0.0607; 0.0886] | 0.4% |
| Pujol et al, 1994 | 23 | 611 | 0.0376 | [0.0240; 0.0559] | 0.3% |
| Raoofi et al, 2012 | 31 | 400 | 0.0775 | [0.0533; 0.1082] | 0.3% |
| Poovorawan et al, 1996 | 61 | 1038 | 0.0588 | [0.0452; 0.0749] | 0.3% |
| Chen et al, 2012 | 2793 | 14208 | 0.1966 | [0.1901; 0.2032] | 0.4% |
| Madden et al, 2016 | 324 | 1161 | 0.2791 | [0.2534; 0.3058] | 0.4% |
| Lopes et al, 2017 | 78 | 300 | 0.2600 | [0.2113; 0.3135] | 0.3% |
| Liang et al, 2014 | 129 | 307 | 0.4202 | [0.3644; 0.4776] | 0.3% |
| Jia et al, 2014 | 3721 | 15862 | 0.2346 | [0.2280; 0.2413] | 0.4% |
| Ma et al, 2010 | 407 | 2090 | 0.1947 | [0.1780; 0.2124] | 0.4% |
| Li et al, 2011 | 69 | 173 | 0.3988 | [0.3253; 0.4759] | 0.3% |
| Li et al, 2014 | 197 | 1638 | 0.1203 | [0.1049; 0.1370] | 0.4% |
| Intharasongkroh et al, 2019 | 0 | 1019 | 0.0000 | [0.0000; 0.0036] | 0.3% |
| Lee et al, 1993 | 43 | 984 | 0.0437 | [0.0318; 0.0584] | 0.3% |
| Liu et al, 2019 | 3419 | 10256 | 0.3334 | [0.3242; 0.3426] | 0.4% |
| Kang et al, 2017 | 121 | 600 | 0.2017 | [0.1703; 0.2360] | 0.3% |
| Li et al, 2006 | 3154 | 7284 | 0.4330 | [0.4216; 0.4445] | 0.4% |
| Matins et al, 2014 | 24 | 431 | 0.0557 | [0.0360; 0.0817] | 0.3% |
| Jahanbakhsh et al, 2017 | 136 | 562 | 0.2420 | [0.2071; 0.2796] | 0.3% |
| Chironna et al, 1999 | 20 | 526 | 0.0380 | [0.0234; 0.0581] | 0.3% |
| Esquivel et al, 2014 | 100 | 273 | 0.3663 | [0.3090; 0.4265] | 0.3% |
| Cui et al, 2016 | 228 | 1028 | 0.2218 | [0.1967; 0.2485] | 0.3% |
| Pilakasiri et al, 2009 | 44 | 381 | 0.1155 | [0.0852; 0.1519] | 0.3% |
| Ghorbani et al, 2007 | 9 | 800 | 0.0112 | [0.0052; 0.0212] | 0.3% |
| Zeng et al, 2014 | 26 | 513 | 0.0507 | [0.0334; 0.0734] | 0.3% |
| Yu et al, 2009 | 841 | 3994 | 0.2106 | [0.1980; 0.2235] | 0.4% |
| Zhang et al, 2017 | 236 | 600 | 0.3933 | [0.3540; 0.4337] | 0.3% |
| Cheng et al, 2007 | 109 | 754 | 0.1446 | [0.1202; 0.1717] | 0.3% |
| Colak et al, 2002 | 3 | 338 | 0.0089 | [0.0018; 0.0257] | 0.3% |
| Cong et al, 2014 | 208 | 890 | 0.2337 | [0.2063; 0.2629] | 0.3% |
| Terzic et al, 2009 | 24 | 400 | 0.0600 | [0.0388; 0.0880] | 0.3% |
| Bayhan et al, 2016 | 17 | 408 | 0.0417 | [0.0245; 0.0659] | 0.3% |
| Abe et al, 2004 | 62 | 341 | 0.1818 | [0.1423; 0.2269] | 0.3% |
| Atabek et al, 2004 | 12 | 210 | 0.0571 | [0.0299; 0.0977] | 0.3% |
| Cevahir et al, 2013 | 23 | 185 | 0.1243 | [0.0805; 0.1807] | 0.3% |
| Kaya et al, 2007 | 2 | 589 | 0.0034 | [0.0004; 0.0122] | 0.3% |
| Lin et al, 2004 | 86 | 2588 | 0.0332 | [0.0267; 0.0409] | 0.4% |
| Meng et al, 2015 | 174 | 1500 | 0.1160 | [0.1002; 0.1333] | 0.4% |
| Mohanavalli et al, 2002 | 16 | 185 | 0.0865 | [0.0502; 0.1366] | 0.3% |
| Sencan et al, 2003 | 34 | 476 | 0.0714 | [0.0500; 0.0984] | 0.3% |
| Shamsizadeh et al, 2009 | 48 | 566 | 0.0848 | [0.0632; 0.1109] | 0.3% |
| Sidal et al, 2001 | 19 | 909 | 0.0209 | [0.0126; 0.0324] | 0.3% |
| Toole et al, 2006 | 132 | 426 | 0.3099 | [0.2662; 0.3562] | 0.3% |
| Saffar et al, 2009 | 25 | 1080 | 0.0231 | [0.0150; 0.0340] | 0.3% |
| Dong et al, 2007 | 2073 | 12052 | 0.1720 | [0.1653; 0.1789] | 0.4% |
| Lu et al, 2009 | 1547 | 8762 | 0.1766 | [0.1686; 0.1847] | 0.4% |
| Munoz et al, 1999 | 374 | 3549 | 0.1054 | [0.0955; 0.1160] | 0.4% |
| Héctor et al, 2017 | 1 | 127 | 0.0079 | [0.0002; 0.0431] | 0.3% |
| Li et al, 2019 | 139 | 946 | 0.1469 | [0.1250; 0.1711] | 0.3% |
| Lee et al, 2013 | 2 | 100 | 0.0200 | [0.0024; 0.0704] | 0.3% |
| Serkan et al, 2005 | 27 | 386 | 0.0699 | [0.0466; 0.1001] | 0.3% |
| Dorcas et al, 2018 | 48 | 393 | 0.1221 | [0.0914; 0.1587] | 0.3% |
| Rui et al, 2018 | 49 | 225 | 0.2178 | [0.1657; 0.2775] | 0.3% |
| Zakieh et al, 2013 | 5 | 136 | 0.0368 | [0.0120; 0.0837] | 0.3% |
| Caron et al, 2008 | 119 | 840 | 0.1417 | [0.1188; 0.1671] | 0.3% |
| Caron et al, 2012 | 16 | 243 | 0.0658 | [0.0381; 0.1047] | 0.3% |
| Cosme et al, 2014 | 25 | 439 | 0.0569 | [0.0372; 0.0829] | 0.3% |
| Cevrioglu et al, 2004 | 31 | 245 | 0.1265 | [0.0876; 0.1748] | 0.3% |
| Farshadpour et al, 2018 | 83 | 1331 | 0.0624 | [0.0500; 0.0767] | 0.4% |
| Huang et al, 2013 | 30 | 293 | 0.1024 | [0.0702; 0.1429] | 0.3% |
| Alizadeh et al, 2016 | 10 | 321 | 0.0312 | [0.0150; 0.0565] | 0.3% |
| Esquivel et al, 2015 | 10 | 150 | 0.0667 | [0.0324; 0.1192] | 0.3% |
| Esquivel et al, 2015 | 25 | 439 | 0.0569 | [0.0372; 0.0829] | 0.3% |
| Trinta et al,2001 | 3 | 405 | 0.0074 | [0.0015; 0.0215] | 0.3% |
| Lemos et al, 2000 | 16 | 1149 | 0.0139 | [0.0080; 0.0225] | 0.4% |
| Random effects model | | 618638 | 0.1279 | [0.1081; 0.1492] | 38.7% |
| Heterogeneity: $I^2 = 100\%$, $\tau^2 = 0.0268$, $p = 0$ | | | | | |
| **Random effects model** | | **1099717** | **0.1247** | **[0.1042; 0.1467]** | **100.0%** |
| Heterogeneity: $I^2 = 100\%$, $\tau^2 = 0.0769$, $p = 0$ | | | | | |
| Residual heterogeneity: $I^2 = 100\%$, $p = 0$ | | | | | |

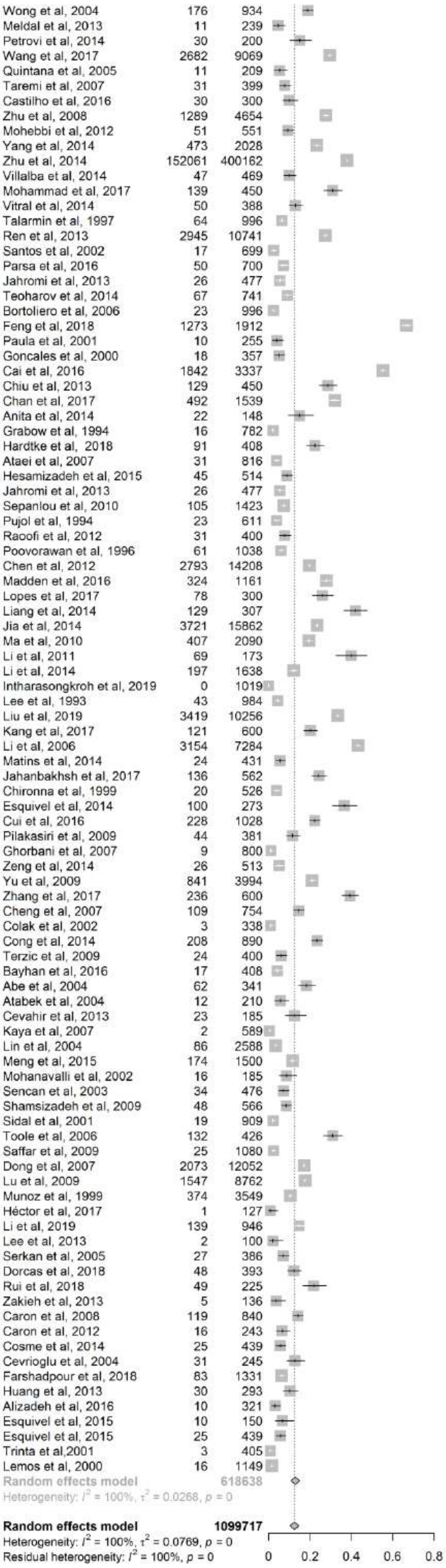

**Figure S11. Forest plot of estimated pooled anti-HEV IgG seroprevalence among general population based on developed countries and developing countries.**

Note: subgroup named mixed means study contained people from several different countries and thus cannot be divided into developed country or developing country.

| Study | Events | Total | Proportion | 95%-CI | Weight |
|---|---|---|---|---|---|
| Subgroup = Developed Country | | | | | |
| Maria et al, 2006 | 96 | 1280 | 0.0750 | [0.0612; 0.0908] | 0.4% |
| Ahn et al, 2005 | 138 | 1030 | 0.1340 | [0.1138; 0.1563] | 0.3% |
| Boutrouille et al, 2007 | 64 | 1998 | 0.0320 | [0.0248; 0.0407] | 0.4% |
| Christensen et al,2018 | 293 | 906 | 0.3234 | [0.2930; 0.3549] | 0.3% |
| Baylis et al, 2012 | 1 | 165010 | 0.0000 | [0.0000; 0.0000] | 0.4% |
| Yoon et al, 2014 | 144 | 2450 | 0.0588 | [0.0498; 0.0688] | 0.4% |
| Takahashi et al, 2010 | 1167 | 22027 | 0.0530 | [0.0501; 0.0560] | 0.4% |
| O'Riordan et al, 2016 | 57 | 1076 | 0.0530 | [0.0404; 0.0681] | 0.3% |
| Spada et al, 2018 | 869 | 10011 | 0.0868 | [0.0814; 0.0925] | 0.4% |
| Moaven et al, 1995 | 17 | 550 | 0.0309 | [0.0181; 0.0490] | 0.3% |
| Nascimento et al, 2016 | 270 | 1656 | 0.1630 | [0.1456; 0.1817] | 0.4% |
| Takeda et al, 2010 | 431 | 12600 | 0.0342 | [0.0311; 0.0375] | 0.4% |
| Minuk et al, 2007 | 11 | 393 | 0.0280 | [0.0141; 0.0495] | 0.3% |
| Verhoef et al, 2011 | 134 | 7072 | 0.0189 | [0.0159; 0.0224] | 0.4% |
| Mooij et al, 2018 | 648 | 2100 | 0.3086 | [0.2889; 0.3288] | 0.4% |
| Lafeber et al, 2017 | 666 | 2422 | 0.2750 | [0.2573; 0.2932] | 0.4% |
| Yun et al, 2011 | 46 | 489 | 0.0941 | [0.0697; 0.1235] | 0.3% |
| Slot et al, 2013 | 1401 | 5239 | 0.2674 | [0.2555; 0.2796] | 0.4% |
| Straková et al, 2016 | 13 | 230 | 0.0565 | [0.0304; 0.0947] | 0.3% |
| Wenzel et al, 2014 | 929 | 2184 | 0.4254 | [0.4045; 0.4464] | 0.4% |
| Sakata et al, 2008 | 45 | 1389 | 0.0324 | [0.0237; 0.0431] | 0.4% |
| Sadik et al, 2016 | 459 | 1199 | 0.3828 | [0.3552; 0.4110] | 0.4% |
| Thom et al, 2017 | 104 | 2311 | 0.0450 | [0.0369; 0.0543] | 0.4% |
| Mor et al, 2015 | 77 | 729 | 0.1056 | [0.0843; 0.1302] | 0.3% |
| Toyoda et al, 2008 | 60 | 592 | 0.1014 | [0.0782; 0.1285] | 0.3% |
| Sauleda et al, 2014 | 1996 | 9998 | 0.1996 | [0.1918; 0.2076] | 0.4% |
| Pittaras et al, 2014 | 25 | 265 | 0.0943 | [0.0620; 0.1361] | 0.3% |
| Niederhauser et al, 2017 | 737 | 3609 | 0.2042 | [0.1912; 0.2177] | 0.4% |
| Hewitt et al, 2018 | 98 | 1013 | 0.0967 | [0.0792; 0.1166] | 0.3% |
| Alberts et al, 2018 | 190 | 7065 | 0.0269 | [0.0232; 0.0309] | 0.4% |
| Gessoni et al, 1996 | 49 | 1889 | 0.0259 | [0.0193; 0.0341] | 0.4% |
| Fukuda et al, 2007 | 168 | 3185 | 0.0527 | [0.0452; 0.0611] | 0.4% |
| Fogeda et al, 2012 | 25 | 2305 | 0.0108 | [0.0070; 0.0160] | 0.4% |
| Fischer et al, 2014 | 163 | 1203 | 0.1355 | [0.1166; 0.1561] | 0.4% |
| Fearon et al, 2017 | 241 | 4102 | 0.0588 | [0.0517; 0.0664] | 0.4% |
| Harvala et al, 2018 | 42 | 480 | 0.0875 | [0.0638; 0.1164] | 0.3% |
| Cleland et al, 2013 | 73 | 1559 | 0.0468 | [0.0369; 0.0585] | 0.4% |
| Cangin et al, 2018 | 377 | 7656 | 0.0492 | [0.0445; 0.0543] | 0.4% |
| Ditah et al, 2014 | 490 | 8814 | 0.0556 | [0.0509; 0.0606] | 0.4% |
| Dimeglio et al, 2018 | 23 | 300 | 0.0767 | [0.0492; 0.1128] | 0.3% |
| Ding et al, 2003 | 15 | 533 | 0.0281 | [0.0158; 0.0460] | 0.3% |
| Hogema et al, 2016 | 11 | 45 | 0.2444 | [0.1288; 0.3954] | 0.3% |
| Hickey et al, 2016 | 16 | 198 | 0.0808 | [0.0469; 0.1279] | 0.3% |
| Mitsui et al, 2005 | 6 | 266 | 0.0226 | [0.0083; 0.0484] | 0.3% |
| Bernal et al, 1995 | 36 | 1757 | 0.0205 | [0.0144; 0.0283] | 0.4% |
| Ricco et al, 2016 | 9 | 199 | 0.0452 | [0.0209; 0.0841] | 0.3% |
| Puttini et al, 2015 | 12 | 132 | 0.0909 | [0.0479; 0.1534] | 0.3% |
| Pavia et al, 1998 | 3 | 360 | 0.0083 | [0.0017; 0.0242] | 0.3% |
| Zuin et al, 2016 | 380 | 3546 | 0.1072 | [0.0972; 0.1178] | 0.4% |
| Scotto et al, 2012 | 2 | 151 | 0.0132 | [0.0016; 0.0470] | 0.3% |
| Sabato et al, 2017 | 4 | 253 | 0.0158 | [0.0043; 0.0400] | 0.3% |
| Love et al, 2018 | 6 | 291 | 0.0206 | [0.0076; 0.0443] | 0.3% |
| Mansuy et al, 2016 | 2371 | 10569 | 0.2243 | [0.2164; 0.2324] | 0.4% |
| Karetny et al, 1995 | 37 | 1416 | 0.0261 | [0.0185; 0.0358] | 0.4% |
| Lucarelli et al, 2016 | 153 | 313 | 0.4888 | [0.4322; 0.5457] | 0.3% |
| Lange et al, 2016 | 177 | 1263 | 0.1401 | [0.1215; 0.1605] | 0.4% |
| Mateos et al, 1998 | 34 | 905 | 0.0376 | [0.0262; 0.0521] | 0.3% |
| Marcantonio et al, 2018 | 10 | 198 | 0.0505 | [0.0245; 0.0909] | 0.3% |
| Karetnyi et al, 1999 | 36 | 734 | 0.0490 | [0.0346; 0.0673] | 0.3% |
| Lanini et al, 2015 | 60 | 1116 | 0.0538 | [0.0413; 0.0687] | 0.4% |
| Kaufmann et al, 2011 | 27 | 550 | 0.0491 | [0.0326; 0.0706] | 0.3% |
| Mansuy et al, 2004 | 46 | 431 | 0.1067 | [0.0792; 0.1398] | 0.3% |
| Mansuy et al, 2015 | 1310 | 3353 | 0.3907 | [0.3741; 0.4074] | 0.4% |
| Juhl et al, 2017 | 70 | 449 | 0.1559 | [0.1236; 0.1928] | 0.3% |
| Juhl et al, 2014 | 69 | 1019 | 0.0677 | [0.0531; 0.0849] | 0.3% |
| Nagler et al, 2014 | 143 | 997 | 0.1434 | [0.1222; 0.1667] | 0.3% |

| | | | | | |
|---|---|---|---|---|---|
| Langer et al, 1997 | 3 | 553 | 0.0054 | [0.0011; 0.0158] | 0.3% |
| Lavanchy et al, 1994 | 14 | 317 | 0.0442 | [0.0244; 0.0730] | 0.3% |
| Mahrt et al, 2018 | 679 | 3000 | 0.2263 | [0.2115; 0.2417] | 0.4% |
| Kuniholm et al, 2009 | 3916 | 18695 | 0.2095 | [0.2037; 0.2154] | 0.4% |
| Krumbholz et al, 2011 | 18 | 116 | 0.1552 | [0.0946; 0.2341] | 0.3% |
| Krumbholz et al, 2014 | 65 | 537 | 0.1210 | [0.0947; 0.1517] | 0.3% |
| Jeggli et al, 2015 | 25 | 752 | 0.0332 | [0.0216; 0.0487] | 0.3% |
| Kantala et al, 2015 | 37 | 385 | 0.0961 | [0.0686; 0.1300] | 0.3% |
| Jablonka et al, 2017 | 122 | 604 | 0.2020 | [0.1707; 0.2363] | 0.3% |
| Kaba et al, 2010 | 57 | 490 | 0.1163 | [0.0893; 0.1481] | 0.3% |
| Rapicetta et al,1999 | 113 | 973 | 0.1161 | [0.0967; 0.1380] | 0.3% |
| Lachish et al, 2013 | 19 | 4970 | 0.0038 | [0.0023; 0.0060] | 0.4% |
| Schielke et al, 2015 | 26 | 126 | 0.2063 | [0.1394; 0.2875] | 0.3% |
| Nübling et al, 2002 | 20 | 511 | 0.0391 | [0.0241; 0.0598] | 0.3% |
| Meader et al, 2008 | 10 | 413 | 0.0242 | [0.0117; 0.0441] | 0.3% |
| Tabili et al, 2013 | 23 | 103 | 0.2233 | [0.1471; 0.3160] | 0.3% |
| Dremsek et al, 2012 | 132 | 853 | 0.1547 | [0.1311; 0.1808] | 0.3% |
| Eick et al, 2010 | 16 | 1500 | 0.0107 | [0.0061; 0.0173] | 0.4% |
| Alecci et al, 1997 | 3 | 244 | 0.0123 | [0.0025; 0.0355] | 0.3% |
| Chaussade et al, 2013 | 83 | 322 | 0.2578 | [0.2109; 0.3092] | 0.3% |
| Whiters et al, 2002 | 3 | 125 | 0.0240 | [0.0050; 0.0685] | 0.3% |
| Teixeira et al, 2017 | 106 | 804 | 0.1318 | [0.1092; 0.1572] | 0.3% |
| Olsen et al, 2006 | 10 | 108 | 0.0926 | [0.0453; 0.1637] | 0.3% |
| Bouwknegt et al, 2008 | 12 | 644 | 0.0186 | [0.0097; 0.0323] | 0.3% |
| Galiana et al, 2008 | 4 | 97 | 0.0412 | [0.0113; 0.1022] | 0.3% |
| Dalton et al, 2017 | 11 | 464 | 0.0237 | [0.0119; 0.0420] | 0.3% |
| Chow et al, 1996 | 30 | 219 | 0.1370 | [0.0944; 0.1897] | 0.3% |
| Buti et al, 2008 | 57 | 1249 | 0.0456 | [0.0347; 0.0587] | 0.4% |
| Goto et al, 2006 | 9 | 344 | 0.0262 | [0.0120; 0.0491] | 0.3% |
| Krumbholz et al, 2014 | 16 | 1646 | 0.0097 | [0.0056; 0.0157] | 0.4% |
| Oliveira et al, 2017 | 4 | 352 | 0.0114 | [0.0031; 0.0288] | 0.3% |
| Ijaz et al, 2009 | 362 | 2731 | 0.1326 | [0.1201; 0.1458] | 0.4% |
| Christophe et al, 2004 | 24 | 315 | 0.0762 | [0.0494; 0.1112] | 0.3% |
| Maria et al, 2010 | 38 | 1040 | 0.0365 | [0.0260; 0.0498] | 0.3% |
| Zhang et al, 2015 | 1352 | 6262 | 0.2159 | [0.2058; 0.2263] | 0.4% |
| Kyvernitakis et al, 2015 | 12 | 115 | 0.1043 | [0.0551; 0.1752] | 0.3% |
| Tashale et al, 2015 | 1081 | 13851 | 0.0780 | [0.0736; 0.0826] | 0.4% |
| Xu et al,2013 | 364 | 1939 | 0.1877 | [0.1706; 0.2058] | 0.4% |
| Stramer et al, 2015 | 27 | 200 | 0.1350 | [0.0909; 0.1903] | 0.3% |
| Meng et al, 2001 | 73 | 400 | 0.1825 | [0.1459; 0.2239] | 0.3% |
| Katrina et al, 2018 | 104 | 1714 | 0.0607 | [0.0498; 0.0730] | 0.4% |
| Random effects model | | 401513 | 0.0859 | [0.0646; 0.1099] | 37.3% |
| Heterogeneity: $I^2 = 100\%$, $\tau^2 = 0.0450$, $p = 0$ | | | | | |
| | | | | | |
| Subgroup = Developing Country | | | | | |
| Chang et al, 2009 | 522 | 2572 | 0.2030 | [0.1876; 0.2190] | 0.4% |
| Geng et al, 2011 | 538 | 2682 | 0.2006 | [0.1856; 0.2163] | 0.4% |
| Yuan et al, 2017 | 268 | 1200 | 0.2233 | [0.2001; 0.2480] | 0.4% |
| Chen et al, 2019 | 799 | 4044 | 0.1976 | [0.1854; 0.2102] | 0.4% |
| Edward et al, 1997 | 571 | 757 | 0.7543 | [0.7220; 0.7846] | 0.3% |
| Corwin et al, 1996 | 26 | 187 | 0.1390 | [0.0929; 0.1970] | 0.3% |
| Hassan et al, 2013 | 76 | 530 | 0.1434 | [0.1147; 0.1762] | 0.3% |
| Fatemeh et al, 2014 | 235 | 510 | 0.4608 | [0.4169; 0.5051] | 0.3% |
| Guo et al, 2009 | 14610 | 44816 | 0.3260 | [0.3217; 0.3304] | 0.4% |
| Halliday et al, 2014 | 120 | 1048 | 0.1145 | [0.0959; 0.1354] | 0.3% |
| Azarkar et al, 2017 | 139 | 554 | 0.2509 | [0.2153; 0.2892] | 0.3% |
| Cainelli et al, 2018 | 11 | 199 | 0.0553 | [0.0279; 0.0968] | 0.3% |
| Assarehzadegan et al, 2007 | 46 | 400 | 0.1150 | [0.0854; 0.1504] | 0.3% |
| Dell'Amico et al, 2011 | 12 | 123 | 0.0976 | [0.0514; 0.1642] | 0.3% |
| Takahashi et al, 2004 | 28 | 249 | 0.1124 | [0.0760; 0.1584] | 0.3% |
| Nasrallah et al, 2017 | 1198 | 5854 | 0.2046 | [0.1944; 0.2152] | 0.4% |
| Zhuang et al, 2014 | 113 | 486 | 0.2325 | [0.1956; 0.2727] | 0.3% |

| | | | | | |
|---|---|---|---|---|---|
| Nouhin et al, 2016 | 85 | 301 | 0.2824 | [0.2322; 0.3369] | 0.3% |
| Castilho et al, 2017 | 49 | 500 | 0.0980 | [0.0734; 0.1275] | 0.3% |
| Olcay et al, 2003 | 57 | 910 | 0.0626 | [0.0478; 0.0804] | 0.3% |
| Rapicetta et al, 1999 | 33 | 2233 | 0.0148 | [0.0102; 0.0207] | 0.4% |
| Nouhin et al, 2018 | 829 | 2004 | 0.4137 | [0.3920; 0.4356] | 0.4% |
| Wong et al, 2004 | 176 | 934 | 0.1884 | [0.1638; 0.2150] | 0.3% |
| Meldal et al, 2013 | 11 | 239 | 0.0460 | [0.0232; 0.0809] | 0.3% |
| Petrovi et al, 2014 | 30 | 200 | 0.1500 | [0.1035; 0.2072] | 0.3% |
| Wang et al, 2017 | 2682 | 9069 | 0.2957 | [0.2864; 0.3052] | 0.4% |
| Tripathy et al, 2018 | 433 | 2447 | 0.1770 | [0.1620; 0.1927] | 0.4% |
| Quintana et al, 2005 | 11 | 209 | 0.0526 | [0.0266; 0.0922] | 0.3% |
| Taremi et al, 2007 | 31 | 399 | 0.0777 | [0.0534; 0.1085] | 0.3% |
| Castilho et al, 2016 | 30 | 300 | 0.1000 | [0.0685; 0.1397] | 0.3% |
| Vivek et al, 2010 | 330 | 2279 | 0.1448 | [0.1306; 0.1599] | 0.4% |
| Zhu et al, 2008 | 1289 | 4654 | 0.2770 | [0.2641; 0.2901] | 0.4% |
| Mohebbi et al, 2012 | 51 | 551 | 0.0926 | [0.0697; 0.1199] | 0.3% |
| Yang et al, 2014 | 473 | 2028 | 0.2332 | [0.2150; 0.2523] | 0.4% |
| Zhu et al, 2014 | 152061 | 400162 | 0.3800 | [0.3785; 0.3815] | 0.4% |
| Villalba et al, 2014 | 47 | 469 | 0.1002 | [0.0746; 0.1310] | 0.3% |
| Shrestha et al, 2016 | 773 | 1845 | 0.4190 | [0.3963; 0.4419] | 0.4% |
| Mohammad et al, 2017 | 139 | 450 | 0.3089 | [0.2665; 0.3538] | 0.3% |
| Yamada et al, 2015 | 160 | 868 | 0.1843 | [0.1591; 0.2118] | 0.3% |
| Vitral et al, 2014 | 50 | 388 | 0.1289 | [0.0972; 0.1663] | 0.3% |
| Peng et al, 1995 | 76 | 997 | 0.0762 | [0.0605; 0.0945] | 0.3% |
| Talarmin et al, 1997 | 64 | 996 | 0.0643 | [0.0498; 0.0813] | 0.3% |
| Ren et al, 2013 | 2945 | 10741 | 0.2742 | [0.2658; 0.2827] | 0.4% |
| Santos et al, 2002 | 17 | 699 | 0.0243 | [0.0142; 0.0387] | 0.3% |
| Miller et al, 1998 | 1 | 403 | 0.0025 | [0.0001; 0.0137] | 0.3% |
| Parsa et al, 2016 | 50 | 700 | 0.0714 | [0.0535; 0.0931] | 0.3% |
| Schreuder et al, 2016 | 25 | 600 | 0.0417 | [0.0271; 0.0609] | 0.3% |
| Jahromi et al, 2013 | 26 | 477 | 0.0545 | [0.0359; 0.0788] | 0.3% |
| Teoharov et al, 2014 | 67 | 741 | 0.0904 | [0.0708; 0.1134] | 0.3% |
| Bortoliero et al, 2006 | 23 | 996 | 0.0231 | [0.0147; 0.0344] | 0.3% |
| Grabarczyk et al, 2018 | 1340 | 3079 | 0.4352 | [0.4176; 0.4529] | 0.4% |
| Feng et al, 2018 | 1273 | 1912 | 0.6658 | [0.6441; 0.6869] | 0.4% |
| Paula et al, 2001 | 10 | 255 | 0.0392 | [0.0190; 0.0709] | 0.3% |
| Goncales et al, 2000 | 18 | 357 | 0.0504 | [0.0302; 0.0785] | 0.3% |
| Cai et al, 2016 | 1842 | 3337 | 0.5520 | [0.5349; 0.5690] | 0.4% |
| Chiu et al, 2013 | 129 | 450 | 0.2867 | [0.2453; 0.3309] | 0.3% |
| Chan et al, 2017 | 492 | 1539 | 0.3197 | [0.2964; 0.3436] | 0.4% |
| Arif et al, 1994 | 160 | 1418 | 0.1128 | [0.0968; 0.1305] | 0.4% |
| Abdelaal et al, 1995 | 100 | 593 | 0.1686 | [0.1394; 0.2012] | 0.3% |
| Amarapurkar et al, 2008 | 416 | 1163 | 0.3577 | [0.3301; 0.3860] | 0.4% |
| Anita et al, 2014 | 22 | 148 | 0.1486 | [0.0956; 0.2164] | 0.3% |
| Grabow et al, 1994 | 16 | 782 | 0.0205 | [0.0117; 0.0330] | 0.3% |
| Hardtke et al, 2018 | 91 | 408 | 0.2230 | [0.1836; 0.2666] | 0.3% |
| Ataei et al, 2007 | 31 | 816 | 0.0380 | [0.0260; 0.0535] | 0.3% |
| Hesamizadeh et al, 2015 | 45 | 514 | 0.0875 | [0.0646; 0.1154] | 0.3% |
| Houcine et al, 2012 | 37 | 687 | 0.0539 | [0.0382; 0.0735] | 0.3% |
| Jahromi et al, 2013 | 26 | 477 | 0.0545 | [0.0359; 0.0788] | 0.3% |
| Sepanlou et al, 2010 | 105 | 1423 | 0.0738 | [0.0607; 0.0886] | 0.4% |
| Adesina et al, 2009 | 25 | 186 | 0.1344 | [0.0889; 0.1920] | 0.3% |
| Pujol et al, 1994 | 23 | 611 | 0.0376 | [0.0240; 0.0559] | 0.3% |
| Raoofi et al, 2012 | 31 | 400 | 0.0775 | [0.0533; 0.1082] | 0.3% |
| Rahman et al, 2014 | 90 | 300 | 0.3000 | [0.2487; 0.3553] | 0.3% |
| Poovorawan et al, 1996 | 61 | 1038 | 0.0588 | [0.0452; 0.0749] | 0.3% |
| Chen et al, 2012 | 2793 | 14208 | 0.1966 | [0.1901; 0.2032] | 0.4% |
| Madden et al, 2016 | 324 | 1161 | 0.2791 | [0.2534; 0.3058] | 0.4% |
| Lopes et al, 2017 | 78 | 300 | 0.2600 | [0.2113; 0.3135] | 0.3% |
| Kotwal et al, 2014 | 712 | 4175 | 0.1705 | [0.1592; 0.1823] | 0.4% |
| Liang et al, 2014 | 129 | 307 | 0.4202 | [0.3644; 0.4776] | 0.3% |
| Johargy et al, 2013 | 168 | 900 | 0.1867 | [0.1617; 0.2137] | 0.3% |
| Jia et al, 2014 | 3721 | 15862 | 0.2346 | [0.2280; 0.2413] | 0.4% |
| Ma et al, 2010 | 407 | 2090 | 0.1947 | [0.1780; 0.2124] | 0.4% |
| Li et al, 2011 | 69 | 173 | 0.3988 | [0.3253; 0.4759] | 0.3% |
| Konomi et al, 1999 | 93 | 574 | 0.1620 | [0.1328; 0.1948] | 0.3% |
| Li et al, 2014 | 197 | 1638 | 0.1203 | [0.1049; 0.1370] | 0.4% |
| Katiyar et al, 2018 | 383 | 633 | 0.6051 | [0.5658; 0.6434] | 0.3% |
| Intharasongkroh et al, 2019 | 0 | 1019 | 0.0000 | [0.0000; 0.0036] | 0.3% |
| Kamel et al, 1995 | 214 | 1259 | 0.1700 | [0.1496; 0.1919] | 0.4% |
| Lee et al, 1993 | 43 | 984 | 0.0437 | [0.0318; 0.0584] | 0.3% |
| Ibarra et al, 1994 | 82 | 469 | 0.1748 | [0.1415; 0.2123] | 0.3% |
| Liu et al, 2019 | 3419 | 10256 | 0.3334 | [0.3242; 0.3426] | 0.4% |
| Khounvisith et al, 2018 | 38 | 210 | 0.1810 | [0.1313; 0.2398] | 0.3% |

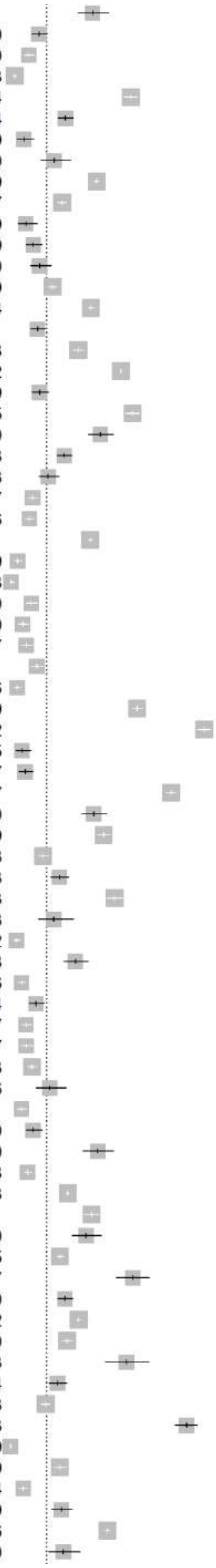

| | | | | | |
|---|---|---|---|---|---|
| Kang et al, 2017 | 121 | 600 | 0.2017 | [0.1703; 0.2360] | 0.3% |
| Korzeniewski et al, 2018 | 13 | 253 | 0.0514 | [0.0276; 0.0863] | 0.3% |
| Li et al, 2006 | 3154 | 7284 | 0.4330 | [0.4216; 0.4445] | 0.4% |
| Labrique et al, 2009 | 255 | 1134 | 0.2249 | [0.2009; 0.2503] | 0.4% |
| Matins et al, 2014 | 24 | 431 | 0.0557 | [0.0360; 0.0817] | 0.3% |
| Jahanbakhsh et al, 2017 | 136 | 562 | 0.2420 | [0.2071; 0.2796] | 0.3% |
| Chironna et al, 1999 | 20 | 526 | 0.0380 | [0.0234; 0.0581] | 0.3% |
| Azman et al, 2017 | 154 | 206 | 0.7476 | [0.6825; 0.8054] | 0.3% |
| Esquivel et al, 2014 | 100 | 273 | 0.3663 | [0.3090; 0.4265] | 0.3% |
| Cui et al, 2016 | 228 | 1028 | 0.2218 | [0.1967; 0.2485] | 0.3% |
| Todys et al, 2015 | 206 | 1027 | 0.2006 | [0.1765; 0.2264] | 0.3% |
| Pilakasiri et al, 2009 | 44 | 381 | 0.1155 | [0.0852; 0.1519] | 0.3% |
| Ghorbani et al, 2007 | 9 | 800 | 0.0112 | [0.0052; 0.0212] | 0.3% |
| Bura et al, 2018 | 2 | 138 | 0.0145 | [0.0018; 0.0514] | 0.3% |
| Zeng et al, 2014 | 26 | 513 | 0.0507 | [0.0334; 0.0734] | 0.3% |
| Yu et al, 2009 | 841 | 3994 | 0.2106 | [0.1980; 0.2235] | 0.4% |
| Widasari et al, 2013 | 16 | 277 | 0.0578 | [0.0334; 0.0921] | 0.3% |
| Zhang et al, 2017 | 236 | 600 | 0.3933 | [0.3540; 0.4337] | 0.3% |
| Drobeniuc et al, 2001 | 63 | 255 | 0.2471 | [0.1954; 0.3047] | 0.3% |
| Cheng et al, 2007 | 109 | 754 | 0.1446 | [0.1202; 0.1717] | 0.3% |
| Pathak et al, 2018 | 341 | 1369 | 0.2491 | [0.2264; 0.2729] | 0.4% |
| Sheikh et al, 2002 | 164 | 273 | 0.6007 | [0.5400; 0.6593] | 0.3% |
| Colak et al, 2002 | 3 | 338 | 0.0089 | [0.0018; 0.0257] | 0.3% |
| Cong et al, 2014 | 208 | 890 | 0.2337 | [0.2063; 0.2629] | 0.3% |
| Terzic et al, 2009 | 24 | 400 | 0.0600 | [0.0388; 0.0880] | 0.3% |
| Bayhan et al, 2016 | 17 | 408 | 0.0417 | [0.0245; 0.0659] | 0.3% |
| Abe et al, 2004 | 62 | 341 | 0.1818 | [0.1423; 0.2269] | 0.3% |
| Aboulata et al, 2005 | 26 | 100 | 0.2600 | [0.1774; 0.3573] | 0.3% |
| Achwan et al, 2007 | 34 | 581 | 0.0585 | [0.0409; 0.0808] | 0.3% |
| Atabek et al, 2004 | 12 | 210 | 0.0571 | [0.0299; 0.0977] | 0.3% |
| Cevahir et al, 2013 | 23 | 185 | 0.1243 | [0.0805; 0.1807] | 0.3% |
| Davaalkham et al, 2009 | 3 | 520 | 0.0058 | [0.0012; 0.0168] | 0.3% |
| Ekanem et al, 2015 | 31 | 402 | 0.0771 | [0.0530; 0.1077] | 0.3% |
| Gandolfo et al, 2003 | 4 | 435 | 0.0092 | [0.0025; 0.0234] | 0.3% |
| Hasan et al, 2016 | 33 | 123 | 0.2683 | [0.1924; 0.3557] | 0.3% |
| Kaya et al, 2007 | 2 | 589 | 0.0034 | [0.0004; 0.0122] | 0.3% |
| Lin et al, 2004 | 86 | 2588 | 0.0332 | [0.0267; 0.0409] | 0.4% |
| Mathur et al, 2001 | 545 | 2070 | 0.2633 | [0.2444; 0.2828] | 0.4% |
| Meng et al, 2015 | 174 | 1500 | 0.1160 | [0.1002; 0.1333] | 0.4% |
| Mohanavalli et al, 2002 | 16 | 185 | 0.0865 | [0.0502; 0.1366] | 0.3% |
| Sencan et al, 2003 | 34 | 476 | 0.0714 | [0.0500; 0.0984] | 0.3% |
| Shamsizadeh et al, 2009 | 48 | 566 | 0.0848 | [0.0632; 0.1109] | 0.3% |
| Sidal et al, 2001 | 19 | 909 | 0.0209 | [0.0126; 0.0324] | 0.3% |
| Bira et al, 2007 | 5 | 717 | 0.0070 | [0.0023; 0.0162] | 0.3% |
| Hau et al, 1999 | 18 | 646 | 0.0279 | [0.0166; 0.0437] | 0.3% |
| Toole et al, 2006 | 132 | 426 | 0.3099 | [0.2662; 0.3562] | 0.3% |
| Saffar et al, 2009 | 25 | 1080 | 0.0231 | [0.0150; 0.0340] | 0.3% |
| Bawazir et al, 2010 | 38 | 356 | 0.1067 | [0.0767; 0.1436] | 0.3% |
| Dong et al, 2007 | 2073 | 12052 | 0.1720 | [0.1653; 0.1789] | 0.4% |
| Lu et al, 2009 | 1547 | 8762 | 0.1766 | [0.1686; 0.1847] | 0.4% |
| Fix et al, 2000 | 4283 | 10026 | 0.4272 | [0.4175; 0.4369] | 0.4% |
| Rey et al, 1997 | 95 | 5196 | 0.0183 | [0.0148; 0.0223] | 0.4% |
| Munoz et al, 1999 | 374 | 3549 | 0.1054 | [0.0955; 0.1160] | 0.4% |
| Héctor et al, 2017 | 1 | 127 | 0.0079 | [0.0002; 0.0431] | 0.3% |
| Li et al, 2019 | 139 | 946 | 0.1469 | [0.1250; 0.1711] | 0.3% |
| Lee et al, 2013 | 2 | 100 | 0.0200 | [0.0024; 0.0704] | 0.3% |
| Serkan et al, 2005 | 27 | 386 | 0.0699 | [0.0466; 0.1001] | 0.3% |
| Dorcas et al, 2018 | 48 | 393 | 0.1221 | [0.0914; 0.1587] | 0.3% |
| Rui et al, 2018 | 49 | 225 | 0.2178 | [0.1657; 0.2775] | 0.3% |
| Zakieh et al, 2013 | 5 | 136 | 0.0368 | [0.0120; 0.0837] | 0.3% |
| Sonia et al, 2006 | 2046 | 2428 | 0.8427 | [0.8276; 0.8569] | 0.4% |
| Niguse et al, 2018 | 359 | 846 | 0.4243 | [0.3908; 0.4585] | 0.3% |
| Caron et al, 2008 | 119 | 840 | 0.1417 | [0.1188; 0.1671] | 0.3% |
| Caron et al, 2012 | 16 | 243 | 0.0658 | [0.0381; 0.1047] | 0.3% |
| Cosme et al, 2014 | 25 | 439 | 0.0569 | [0.0372; 0.0829] | 0.3% |
| Cevrioglu et al, 2004 | 31 | 245 | 0.1265 | [0.0876; 0.1748] | 0.3% |
| Farshadpour et al, 2018 | 83 | 1331 | 0.0624 | [0.0500; 0.0767] | 0.4% |
| Gad et al, 2011 | 68 | 116 | 0.5862 | [0.4910; 0.6769] | 0.3% |
| Adjei et al, 2009 | 16 | 157 | 0.1019 | [0.0594; 0.1602] | 0.3% |
| Huang et al, 2013 | 30 | 293 | 0.1024 | [0.0702; 0.1429] | 0.3% |
| Paschale et al, 2016 | 62 | 278 | 0.2230 | [0.1755; 0.2766] | 0.3% |
| Alizadeh et al, 2016 | 10 | 321 | 0.0312 | [0.0150; 0.0565] | 0.3% |
| Abebe et al, 2017 | 122 | 386 | 0.3161 | [0.2700; 0.3650] | 0.3% |
| Traore et al, 2012 | 56 | 178 | 0.3146 | [0.2472; 0.3883] | 0.3% |
| Aubry et al, 1997 | 18 | 129 | 0.1395 | [0.0848; 0.2115] | 0.3% |
| Gambel et al, 1998 | 14 | 112 | 0.1250 | [0.0701; 0.2008] | 0.3% |
| Esquivel et al, 2015 | 10 | 150 | 0.0667 | [0.0324; 0.1192] | 0.3% |
| Esquivel et al, 2015 | 25 | 439 | 0.0569 | [0.0372; 0.0829] | 0.3% |
| Perez et al, 1996 | 17 | 399 | 0.0426 | [0.0250; 0.0673] | 0.3% |
| Wassaf et al,2014 | 19 | 433 | 0.0439 | [0.0266; 0.0677] | 0.3% |
| Munne et al, 2014 | 31 | 202 | 0.1535 | [0.1067; 0.2107] | 0.3% |
| Bartoloni et al, 1999 | 36 | 490 | 0.0735 | [0.0520; 0.1003] | 0.3% |
| Leon et al, 1999 | 64 | 318 | 0.2013 | [0.1586; 0.2496] | 0.3% |
| Trinta et al,2001 | 3 | 405 | 0.0074 | [0.0015; 0.0215] | 0.3% |
| Lemos et al, 2000 | 16 | 1149 | 0.0139 | [0.0080; 0.0225] | 0.4% |
| Taha et al, 2015 | 80 | 397 | 0.2015 | [0.1631; 0.2444] | 0.3% |
| Random effects model | | 689452 | 0.1483 | [0.1298; 0.1677] | 61.7% |
| Heterogeneity: $I^2 = 100\%$, $\tau^2 = 0.0322$, $p = 0$ | | | | | |
| Subgroup = Mixed | | | | | |
| Abe et al, 2006 | 1720 | 5233 | 0.3287 | [0.3160; 0.3416] | 0.4% |
| Izopet et al, 2015 | 1083 | 2538 | 0.4267 | [0.4074; 0.4462] | 0.4% |
| Gambel et al, 1998 | 209 | 981 | 0.2130 | [0.1878; 0.2400] | 0.3% |
| Random effects model | | 8752 | 0.3202 | [0.2270; 0.4213] | 1.1% |
| Heterogeneity: $I^2 = 99\%$, $\tau^2 = 0.0085$, $p < 0.01$ | | | | | |
| **Random effects model** | | **1099717** | **0.1247** | **[0.1042; 0.1467]** | **100.0%** |
| Heterogeneity: $I^2 = 100\%$, $\tau^2 = 0.0769$, $p = 0$ | | | | | |
| Residual heterogeneity: $I^2 = 100\%$, $p = 0$ | | | | | |

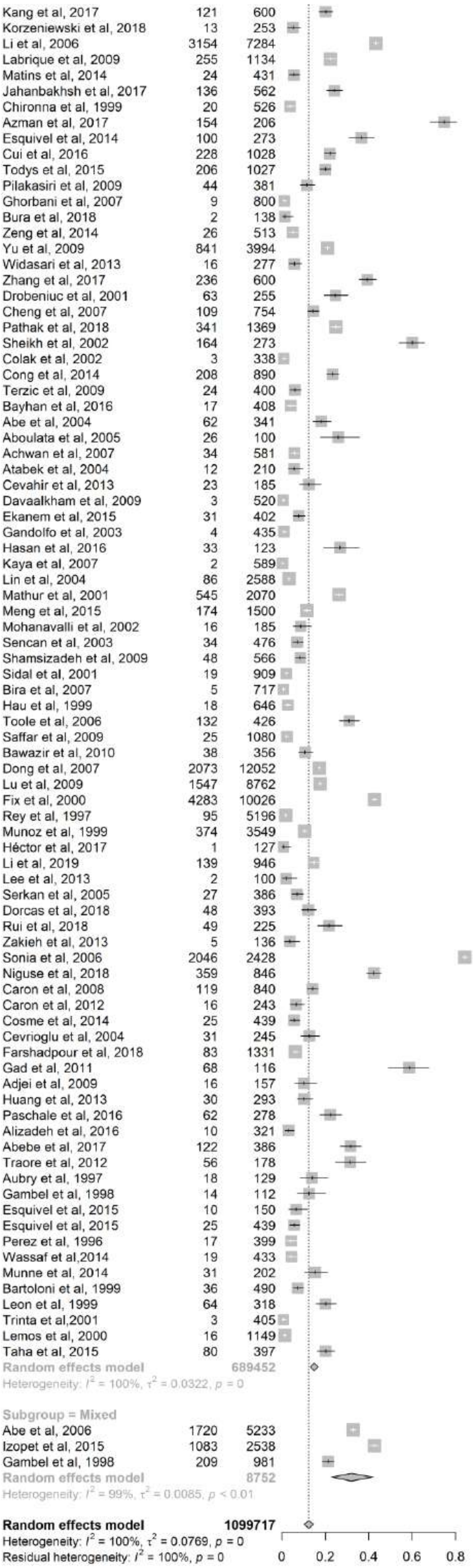

**Figure S12. Forest plot of estimated pooled anti-HEV IgG seroprevalence among general population based on time period of 1993-2006 and 2007-2019.**

| Study | Events | Total | Proportion | 95%-CI | Weight |
|---|---|---|---|---|---|
| Time = 1993-2006 | | | | | |
| Edward et al, 1997 | 571 | 757 | 0.7543 | [0.7220; 0.7846] | 0.3% |
| Corwin et al, 1996 | 26 | 187 | 0.1390 | [0.0929; 0.1970] | 0.3% |
| Ahn et al, 2005 | 138 | 1030 | 0.1340 | [0.1138; 0.1563] | 0.3% |
| Abe et al, 2006 | 1720 | 5233 | 0.3287 | [0.3160; 0.3416] | 0.4% |
| Takahashi et al, 2004 | 28 | 249 | 0.1124 | [0.0760; 0.1584] | 0.3% |
| Moaven et al, 1995 | 17 | 550 | 0.0309 | [0.0181; 0.0490] | 0.3% |
| Olcay et al, 2003 | 57 | 910 | 0.0626 | [0.0478; 0.0804] | 0.3% |
| Rapicetta et al, 1999 | 33 | 2233 | 0.0148 | [0.0102; 0.0207] | 0.4% |
| Wong et al, 2004 | 176 | 934 | 0.1884 | [0.1638; 0.2150] | 0.3% |
| Quintana et al, 2005 | 11 | 209 | 0.0526 | [0.0266; 0.0922] | 0.3% |
| Peng et al, 1995 | 76 | 997 | 0.0762 | [0.0605; 0.0945] | 0.3% |
| Talarmin et al, 1997 | 64 | 996 | 0.0643 | [0.0498; 0.0813] | 0.3% |
| Santos et al, 2002 | 17 | 699 | 0.0243 | [0.0142; 0.0387] | 0.3% |
| Miller et al, 1998 | 1 | 403 | 0.0025 | [0.0001; 0.0137] | 0.3% |
| Bortoliero et al, 2006 | 23 | 996 | 0.0231 | [0.0147; 0.0344] | 0.3% |
| Gessoni et al, 1996 | 49 | 1889 | 0.0259 | [0.0193; 0.0341] | 0.4% |
| Paula et al, 2001 | 10 | 255 | 0.0392 | [0.0190; 0.0709] | 0.3% |
| Goncales et al, 2000 | 18 | 357 | 0.0504 | [0.0302; 0.0785] | 0.3% |
| Ding et al, 2003 | 15 | 533 | 0.0281 | [0.0158; 0.0460] | 0.3% |
| Arif et al, 1994 | 160 | 1418 | 0.1128 | [0.0968; 0.1305] | 0.4% |
| Abdelaal et al, 1995 | 100 | 593 | 0.1686 | [0.1394; 0.2012] | 0.3% |
| Grabow et al, 1994 | 16 | 782 | 0.0205 | [0.0117; 0.0330] | 0.3% |
| Mitsui et al, 2005 | 6 | 266 | 0.0226 | [0.0083; 0.0484] | 0.3% |
| Bernal et al, 1995 | 36 | 1757 | 0.0205 | [0.0144; 0.0283] | 0.4% |
| Pujol et al, 1994 | 23 | 611 | 0.0376 | [0.0240; 0.0559] | 0.3% |
| Pavia et al, 1998 | 3 | 360 | 0.0083 | [0.0017; 0.0242] | 0.3% |
| Poovorawan et al, 1996 | 61 | 1038 | 0.0588 | [0.0452; 0.0749] | 0.3% |
| Karetny et al, 1995 | 37 | 1416 | 0.0261 | [0.0185; 0.0358] | 0.4% |
| Mateos et al, 1998 | 34 | 905 | 0.0376 | [0.0262; 0.0521] | 0.3% |
| Karetnyi et al, 1999 | 36 | 734 | 0.0490 | [0.0346; 0.0673] | 0.3% |
| Mansuy et al, 2004 | 46 | 431 | 0.1067 | [0.0792; 0.1398] | 0.3% |
| Konomi et al, 1999 | 93 | 574 | 0.1620 | [0.1328; 0.1948] | 0.3% |
| Kamel et al, 1995 | 214 | 1259 | 0.1700 | [0.1496; 0.1919] | 0.4% |
| Langer et al, 1997 | 3 | 553 | 0.0054 | [0.0011; 0.0158] | 0.3% |
| Lee et al, 1993 | 43 | 984 | 0.0437 | [0.0318; 0.0584] | 0.3% |
| Lavanchy et al, 1994 | 14 | 317 | 0.0442 | [0.0244; 0.0730] | 0.3% |
| Ibarra et al, 1994 | 82 | 469 | 0.1748 | [0.1415; 0.2123] | 0.3% |
| Li et al, 2006 | 3154 | 7284 | 0.4330 | [0.4216; 0.4445] | 0.4% |
| Chironna et al, 1999 | 20 | 526 | 0.0380 | [0.0234; 0.0581] | 0.3% |
| Rapicetta et al,1999 | 113 | 973 | 0.1161 | [0.0967; 0.1380] | 0.3% |
| Nübling et al, 2002 | 20 | 511 | 0.0391 | [0.0241; 0.0598] | 0.3% |
| Gambel et al, 1998 | 209 | 981 | 0.2130 | [0.1878; 0.2400] | 0.3% |
| Alecci et al, 1997 | 3 | 244 | 0.0123 | [0.0025; 0.0355] | 0.3% |
| Whiters et al, 2002 | 3 | 125 | 0.0240 | [0.0050; 0.0685] | 0.3% |
| Olsen et al, 2006 | 10 | 108 | 0.0926 | [0.0453; 0.1637] | 0.3% |
| Drobeniuc et al, 2001 | 63 | 255 | 0.2471 | [0.1954; 0.3047] | 0.3% |
| Sheikh et al, 2002 | 164 | 273 | 0.6007 | [0.5400; 0.6593] | 0.3% |
| Chow et al, 1996 | 30 | 219 | 0.1370 | [0.0944; 0.1897] | 0.3% |
| Colak et al, 2002 | 3 | 338 | 0.0089 | [0.0018; 0.0257] | 0.3% |
| Abe et al, 2004 | 62 | 341 | 0.1818 | [0.1423; 0.2269] | 0.3% |
| Aboulata et al, 2005 | 26 | 100 | 0.2600 | [0.1774; 0.3573] | 0.3% |
| Atabek et al, 2004 | 12 | 210 | 0.0571 | [0.0299; 0.0977] | 0.3% |
| Gandolfo et al, 2003 | 4 | 435 | 0.0092 | [0.0025; 0.0234] | 0.3% |
| Goto et al, 2006 | 9 | 344 | 0.0262 | [0.0120; 0.0491] | 0.3% |
| Lin et al, 2004 | 86 | 2588 | 0.0332 | [0.0267; 0.0409] | 0.4% |
| Mathur et al, 2001 | 545 | 2070 | 0.2633 | [0.2444; 0.2828] | 0.4% |
| Mohanavalli et al, 2002 | 16 | 185 | 0.0865 | [0.0502; 0.1366] | 0.3% |
| Sencan et al, 2003 | 34 | 476 | 0.0714 | [0.0500; 0.0984] | 0.3% |
| Sidal et al, 2001 | 19 | 909 | 0.0209 | [0.0126; 0.0324] | 0.3% |
| Hau et al, 1999 | 18 | 646 | 0.0279 | [0.0166; 0.0437] | 0.3% |
| Toole et al, 2006 | 132 | 426 | 0.3099 | [0.2662; 0.3562] | 0.3% |
| Fix et al, 2000 | 4283 | 10026 | 0.4272 | [0.4175; 0.4369] | 0.4% |
| Rey et al, 1997 | 95 | 5196 | 0.0183 | [0.0148; 0.0223] | 0.4% |

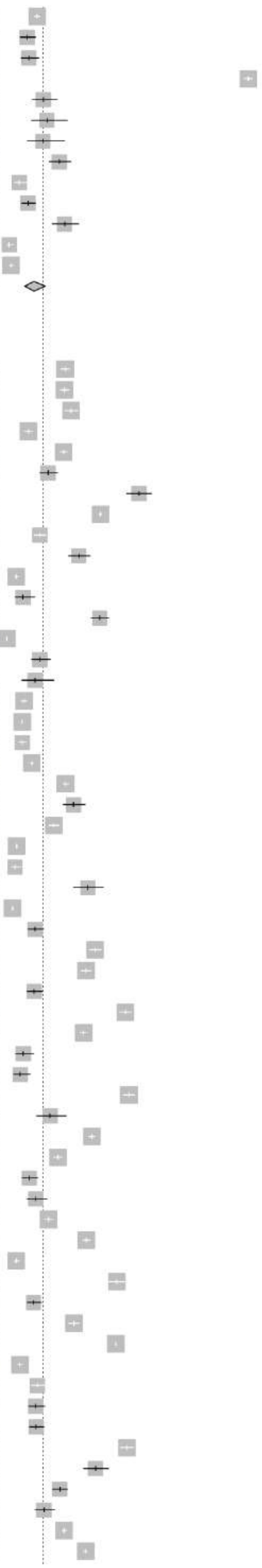

| | | | | | |
|---|---|---|---|---|---|
| Munoz et al, 1999 | 374 | 3549 | 0.1054 | [0.0955; 0.1160] | 0.4% |
| Serkan et al, 2005 | 27 | 386 | 0.0699 | [0.0466; 0.1001] | 0.3% |
| Christophe et al, 2004 | 24 | 315 | 0.0762 | [0.0494; 0.1112] | 0.3% |
| Sonia et al, 2006 | 2046 | 2428 | 0.8427 | [0.8276; 0.8569] | 0.4% |
| Cevrioglu et al, 2004 | 31 | 245 | 0.1265 | [0.0876; 0.1748] | 0.3% |
| Aubry et al, 1997 | 18 | 129 | 0.1395 | [0.0848; 0.2115] | 0.3% |
| Gambel et al, 1998 | 14 | 112 | 0.1250 | [0.0701; 0.2008] | 0.3% |
| Meng et al, 2001 | 73 | 400 | 0.1825 | [0.1459; 0.2239] | 0.3% |
| Perez et al, 1996 | 17 | 399 | 0.0426 | [0.0250; 0.0673] | 0.3% |
| Bartoloni et al, 1999 | 36 | 490 | 0.0735 | [0.0520; 0.1003] | 0.3% |
| Leon et al, 1999 | 64 | 318 | 0.2013 | [0.1586; 0.2496] | 0.3% |
| Trinta et al,2001 | 3 | 405 | 0.0074 | [0.0015; 0.0215] | 0.3% |
| Lemos et al, 2000 | 16 | 1149 | 0.0139 | [0.0080; 0.0225] | 0.4% |
| **Random effects model** | | 79998 | **0.0943** | **[0.0611; 0.1337]** | **26.5%** |
| Heterogeneity: $I^2 = 100\%$, $\tau^2 = 0.0761$, $p = 0$ | | | | | |
| | | | | | |
| **Time = 2007-2019** | | | | | |
| Chang et al, 2009 | 522 | 2572 | 0.2030 | [0.1876; 0.2190] | 0.4% |
| Geng et al, 2011 | 538 | 2682 | 0.2006 | [0.1856; 0.2163] | 0.4% |
| Yuan et al, 2017 | 268 | 1200 | 0.2233 | [0.2001; 0.2480] | 0.4% |
| Maria et al, 2006 | 96 | 1280 | 0.0750 | [0.0612; 0.0908] | 0.4% |
| Chen et al, 2019 | 799 | 4044 | 0.1976 | [0.1854; 0.2102] | 0.4% |
| Hassan et al, 2013 | 76 | 530 | 0.1434 | [0.1147; 0.1762] | 0.3% |
| Fatemeh et al, 2014 | 235 | 510 | 0.4608 | [0.4169; 0.5051] | 0.3% |
| Guo et al, 2009 | 14610 | 44816 | 0.3260 | [0.3217; 0.3304] | 0.4% |
| Halliday et al, 2014 | 120 | 1048 | 0.1145 | [0.0959; 0.1354] | 0.3% |
| Azarkar et al, 2017 | 139 | 554 | 0.2509 | [0.2153; 0.2892] | 0.3% |
| Boutrouille et al, 2007 | 64 | 1998 | 0.0320 | [0.0248; 0.0407] | 0.4% |
| Cainelli et al, 2018 | 11 | 199 | 0.0553 | [0.0279; 0.0968] | 0.3% |
| Christensen et al,2018 | 293 | 906 | 0.3234 | [0.2930; 0.3549] | 0.3% |
| Baylis et al, 2012 | 1 | 165010 | 0.0000 | [0.0000; 0.0000] | 0.4% |
| Assarehzadegan et al, 2007 | 46 | 400 | 0.1150 | [0.0854; 0.1504] | 0.3% |
| Dell'Amico et al, 2011 | 12 | 123 | 0.0976 | [0.0514; 0.1642] | 0.3% |
| Yoon et al, 2014 | 144 | 2450 | 0.0588 | [0.0498; 0.0688] | 0.4% |
| Takahashi et al, 2010 | 1167 | 22027 | 0.0530 | [0.0501; 0.0560] | 0.4% |
| O'Riordan et al, 2016 | 57 | 1076 | 0.0530 | [0.0404; 0.0681] | 0.3% |
| Spada et al, 2018 | 869 | 10011 | 0.0868 | [0.0814; 0.0925] | 0.4% |
| Nasrallah et al, 2017 | 1198 | 5854 | 0.2046 | [0.1944; 0.2152] | 0.4% |
| Zhuang et al, 2014 | 113 | 486 | 0.2325 | [0.1956; 0.2727] | 0.3% |
| Nascimento et al, 2016 | 270 | 1656 | 0.1630 | [0.1456; 0.1817] | 0.4% |
| Takeda et al, 2010 | 431 | 12600 | 0.0342 | [0.0311; 0.0375] | 0.4% |
| Minuk et al, 2007 | 11 | 393 | 0.0280 | [0.0141; 0.0495] | 0.3% |
| Nouhin et al, 2016 | 85 | 301 | 0.2824 | [0.2322; 0.3369] | 0.3% |
| Verhoef et al, 2011 | 134 | 7072 | 0.0189 | [0.0159; 0.0224] | 0.4% |
| Castilho et al, 2017 | 49 | 500 | 0.0980 | [0.0734; 0.1275] | 0.3% |
| Mooij et al, 2018 | 648 | 2100 | 0.3086 | [0.2889; 0.3288] | 0.4% |
| Lafeber et al, 2017 | 666 | 2422 | 0.2750 | [0.2573; 0.2932] | 0.4% |
| Yun et al, 2011 | 46 | 489 | 0.0941 | [0.0697; 0.1235] | 0.3% |
| Nouhin et al, 2018 | 829 | 2004 | 0.4137 | [0.3920; 0.4356] | 0.4% |
| Slot et al, 2013 | 1401 | 5239 | 0.2674 | [0.2555; 0.2796] | 0.4% |
| Straková et al, 2016 | 13 | 230 | 0.0565 | [0.0304; 0.0947] | 0.3% |
| Meldal et al, 2013 | 11 | 239 | 0.0460 | [0.0232; 0.0809] | 0.3% |
| Wenzel et al, 2014 | 929 | 2184 | 0.4254 | [0.4045; 0.4464] | 0.4% |
| Petrovi et al, 2014 | 30 | 200 | 0.1500 | [0.1035; 0.2072] | 0.3% |
| Wang et al, 2017 | 2682 | 9069 | 0.2957 | [0.2864; 0.3052] | 0.4% |
| Tripathy et al, 2018 | 433 | 2447 | 0.1770 | [0.1620; 0.1927] | 0.4% |
| Taremi et al, 2007 | 31 | 399 | 0.0777 | [0.0534; 0.1085] | 0.3% |
| Castilho et al, 2016 | 30 | 300 | 0.1000 | [0.0685; 0.1397] | 0.3% |
| Vivek et al, 2010 | 330 | 2279 | 0.1448 | [0.1306; 0.1599] | 0.4% |
| Zhu et al, 2008 | 1289 | 4654 | 0.2770 | [0.2641; 0.2901] | 0.4% |
| Sakata et al, 2008 | 45 | 1389 | 0.0324 | [0.0237; 0.0431] | 0.4% |
| Sadik et al, 2016 | 459 | 1199 | 0.3828 | [0.3552; 0.4110] | 0.4% |
| Mohebbi et al, 2012 | 51 | 551 | 0.0926 | [0.0697; 0.1199] | 0.3% |
| Yang et al, 2014 | 473 | 2028 | 0.2332 | [0.2150; 0.2523] | 0.4% |
| Zhu et al, 2014 | 152061 | 400162 | 0.3800 | [0.3785; 0.3815] | 0.4% |
| Thom et al, 2017 | 104 | 2311 | 0.0450 | [0.0369; 0.0543] | 0.4% |
| Mor et al, 2015 | 77 | 729 | 0.1056 | [0.0843; 0.1302] | 0.3% |
| Villalba et al, 2014 | 47 | 469 | 0.1002 | [0.0746; 0.1310] | 0.3% |
| Toyoda et al, 2008 | 60 | 592 | 0.1014 | [0.0782; 0.1285] | 0.3% |
| Shrestha et al, 2016 | 773 | 1845 | 0.4190 | [0.3963; 0.4419] | 0.4% |
| Mohammad et al, 2017 | 139 | 450 | 0.3089 | [0.2665; 0.3538] | 0.3% |
| Yamada et al, 2015 | 160 | 868 | 0.1843 | [0.1591; 0.2118] | 0.3% |
| Vitral et al, 2014 | 50 | 388 | 0.1289 | [0.0972; 0.1663] | 0.3% |
| Sauleda et al, 2014 | 1996 | 9998 | 0.1996 | [0.1918; 0.2076] | 0.4% |
| Ren et al, 2013 | 2945 | 10741 | 0.2742 | [0.2658; 0.2827] | 0.4% |

| | | | | | |
|---|---|---|---|---|---|
| Parsa et al, 2016 | 50 | 700 | 0.0714 | [0.0535; 0.0931] | 0.3% |
| Pittaras et al, 2014 | 25 | 265 | 0.0943 | [0.0620; 0.1361] | 0.3% |
| Schreuder et al, 2016 | 25 | 600 | 0.0417 | [0.0271; 0.0609] | 0.3% |
| Jahromi et al, 2013 | 26 | 477 | 0.0545 | [0.0359; 0.0788] | 0.3% |
| Niederhauser et al, 2017 | 737 | 3609 | 0.2042 | [0.1912; 0.2177] | 0.4% |
| Teoharov et al, 2014 | 67 | 741 | 0.0904 | [0.0708; 0.1134] | 0.3% |
| Hewitt et al, 2018 | 98 | 1013 | 0.0967 | [0.0792; 0.1166] | 0.3% |
| Alberts et al, 2018 | 190 | 7065 | 0.0269 | [0.0232; 0.0309] | 0.4% |
| Grabarczyk et al, 2018 | 1340 | 3079 | 0.4352 | [0.4176; 0.4529] | 0.4% |
| Fukuda et al, 2007 | 168 | 3185 | 0.0527 | [0.0452; 0.0611] | 0.4% |
| Fogeda et al, 2012 | 25 | 2305 | 0.0108 | [0.0070; 0.0160] | 0.4% |
| Fischer et al, 2014 | 163 | 1203 | 0.1355 | [0.1166; 0.1561] | 0.4% |
| Feng et al, 2018 | 1273 | 1912 | 0.6658 | [0.6441; 0.6869] | 0.4% |
| Fearon et al, 2017 | 241 | 4102 | 0.0588 | [0.0517; 0.0664] | 0.4% |
| Harvala et al, 2018 | 42 | 480 | 0.0875 | [0.0638; 0.1164] | 0.3% |
| Cleland et al, 2013 | 73 | 1559 | 0.0468 | [0.0369; 0.0585] | 0.4% |
| Cangin et al, 2018 | 377 | 7656 | 0.0492 | [0.0445; 0.0543] | 0.4% |
| Ditah et al, 2014 | 490 | 8814 | 0.0556 | [0.0509; 0.0606] | 0.4% |
| Cai et al, 2016 | 1842 | 3337 | 0.5520 | [0.5349; 0.5690] | 0.4% |
| Chiu et al, 2013 | 129 | 450 | 0.2867 | [0.2453; 0.3309] | 0.3% |
| Dimeglio et al, 2018 | 23 | 300 | 0.0767 | [0.0492; 0.1128] | 0.3% |
| Chan et al, 2017 | 492 | 1539 | 0.3197 | [0.2964; 0.3436] | 0.4% |
| Amarapurkar et al, 2008 | 416 | 1163 | 0.3577 | [0.3301; 0.3860] | 0.4% |
| Anita et al, 2014 | 22 | 148 | 0.1486 | [0.0956; 0.2164] | 0.3% |
| Hardtke et al, 2018 | 91 | 408 | 0.2230 | [0.1836; 0.2666] | 0.3% |
| Ataei et al, 2007 | 31 | 816 | 0.0380 | [0.0260; 0.0535] | 0.3% |
| Hesamizadeh et al, 2015 | 45 | 514 | 0.0875 | [0.0646; 0.1154] | 0.3% |
| Hogema et al, 2016 | 11 | 45 | 0.2444 | [0.1288; 0.3954] | 0.3% |
| Hickey et al, 2016 | 16 | 198 | 0.0808 | [0.0469; 0.1279] | 0.3% |
| Houcine et al, 2012 | 37 | 687 | 0.0539 | [0.0382; 0.0735] | 0.3% |
| Jahromi et al, 2013 | 26 | 477 | 0.0545 | [0.0359; 0.0788] | 0.3% |
| Sepanlou et al, 2010 | 105 | 1423 | 0.0738 | [0.0607; 0.0886] | 0.4% |
| Adesina et al, 2009 | 25 | 186 | 0.1344 | [0.0889; 0.1920] | 0.3% |
| Ricco et al, 2016 | 9 | 199 | 0.0452 | [0.0209; 0.0841] | 0.3% |
| Puttini et al, 2015 | 12 | 132 | 0.0909 | [0.0479; 0.1534] | 0.3% |
| Zuin et al, 2016 | 380 | 3546 | 0.1072 | [0.0972; 0.1178] | 0.4% |
| Scotto et al, 2012 | 2 | 151 | 0.0132 | [0.0016; 0.0470] | 0.3% |
| Raoofi et al, 2012 | 31 | 400 | 0.0775 | [0.0533; 0.1082] | 0.3% |
| Rahman et al, 2014 | 90 | 300 | 0.3000 | [0.2487; 0.3553] | 0.3% |
| Sabato et al, 2017 | 4 | 253 | 0.0158 | [0.0043; 0.0400] | 0.3% |
| Chen et al, 2012 | 2793 | 14208 | 0.1966 | [0.1901; 0.2032] | 0.4% |
| Madden et al, 2016 | 324 | 1161 | 0.2791 | [0.2534; 0.3058] | 0.4% |
| Love et al, 2018 | 6 | 291 | 0.0206 | [0.0076; 0.0443] | 0.3% |
| Lopes et al, 2017 | 78 | 300 | 0.2600 | [0.2113; 0.3135] | 0.3% |
| Mansuy et al, 2016 | 2371 | 10569 | 0.2243 | [0.2164; 0.2324] | 0.4% |
| Lucarelli et al, 2016 | 153 | 313 | 0.4888 | [0.4322; 0.5457] | 0.3% |
| Lange et al, 2016 | 177 | 1263 | 0.1401 | [0.1215; 0.1605] | 0.4% |
| Kotwal et al, 2014 | 712 | 4175 | 0.1705 | [0.1592; 0.1823] | 0.4% |
| Liang et al, 2014 | 129 | 307 | 0.4202 | [0.3644; 0.4776] | 0.3% |
| Marcantonio et al, 2018 | 10 | 198 | 0.0505 | [0.0245; 0.0909] | 0.3% |
| Lanini et al, 2015 | 60 | 1116 | 0.0538 | [0.0413; 0.0687] | 0.4% |
| Johargy et al, 2013 | 168 | 900 | 0.1867 | [0.1617; 0.2137] | 0.3% |
| Jia et al, 2014 | 3721 | 15862 | 0.2346 | [0.2280; 0.2413] | 0.4% |
| Ma et al, 2010 | 407 | 2090 | 0.1947 | [0.1780; 0.2124] | 0.4% |
| Kaufmann et al, 2011 | 27 | 550 | 0.0491 | [0.0326; 0.0706] | 0.3% |
| Mansuy et al, 2015 | 1310 | 3353 | 0.3907 | [0.3741; 0.4074] | 0.4% |
| Izopet et al, 2015 | 1083 | 2538 | 0.4267 | [0.4074; 0.4462] | 0.4% |
| Li et al, 2011 | 69 | 173 | 0.3988 | [0.3253; 0.4759] | 0.3% |
| Li et al, 2014 | 197 | 1638 | 0.1203 | [0.1049; 0.1370] | 0.4% |
| Juhl et al, 2017 | 70 | 449 | 0.1559 | [0.1236; 0.1928] | 0.3% |
| Katiyar et al, 2018 | 383 | 633 | 0.6051 | [0.5658; 0.6434] | 0.3% |
| Intharasongkroh et al, 2019 | 0 | 1019 | 0.0000 | [0.0000; 0.0036] | 0.3% |
| Juhl et al, 2014 | 69 | 1019 | 0.0677 | [0.0531; 0.0849] | 0.3% |
| Nagler et al, 2014 | 143 | 997 | 0.1434 | [0.1222; 0.1667] | 0.3% |
| Liu et al, 2019 | 3419 | 10256 | 0.3334 | [0.3242; 0.3426] | 0.4% |
| Mahrt et al, 2018 | 679 | 3000 | 0.2263 | [0.2115; 0.2417] | 0.4% |
| Kuniholm et al, 2009 | 3916 | 18695 | 0.2095 | [0.2037; 0.2154] | 0.4% |
| Krumbholz et al, 2011 | 18 | 116 | 0.1552 | [0.0946; 0.2341] | 0.3% |
| Khounvisith et al, 2018 | 38 | 210 | 0.1810 | [0.1313; 0.2398] | 0.3% |

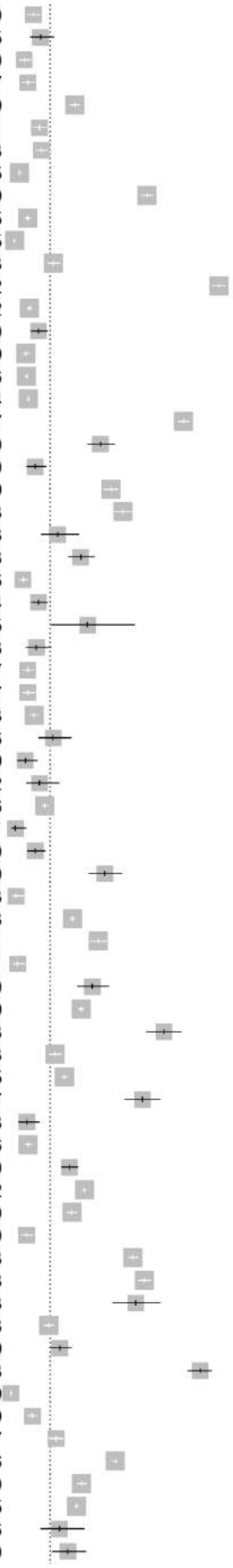

| Study | Events | Total | Proportion | 95%-CI | Weight |
|---|---|---|---|---|---|
| Krumbholz et al, 2014 | 65 | 537 | 0.1210 | [0.0947; 0.1517] | 0.3% |
| Jeggli et al, 2015 | 25 | 752 | 0.0332 | [0.0216; 0.0487] | 0.3% |
| Kantala et al, 2015 | 37 | 385 | 0.0961 | [0.0686; 0.1300] | 0.3% |
| Kang et al, 2017 | 121 | 600 | 0.2017 | [0.1703; 0.2360] | 0.3% |
| Korzeniewski et al, 2018 | 13 | 253 | 0.0514 | [0.0276; 0.0863] | 0.3% |
| Labrique et al, 2009 | 255 | 1134 | 0.2249 | [0.2009; 0.2503] | 0.4% |
| Jablonka et al, 2017 | 122 | 604 | 0.2020 | [0.1707; 0.2363] | 0.3% |
| Matins et al, 2014 | 24 | 431 | 0.0557 | [0.0360; 0.0817] | 0.3% |
| Jahanbakhsh et al, 2017 | 136 | 562 | 0.2420 | [0.2071; 0.2796] | 0.3% |
| Kaba et al, 2010 | 57 | 490 | 0.1163 | [0.0893; 0.1481] | 0.3% |
| Azman et al, 2017 | 154 | 206 | 0.7476 | [0.6825; 0.8054] | 0.3% |
| Lachish et al, 2013 | 19 | 4970 | 0.0038 | [0.0023; 0.0060] | 0.4% |
| Esquivel et al, 2014 | 100 | 273 | 0.3663 | [0.3090; 0.4265] | 0.3% |
| Cui et al, 2016 | 228 | 1028 | 0.2218 | [0.1967; 0.2485] | 0.3% |
| Schielke et al, 2015 | 26 | 126 | 0.2063 | [0.1394; 0.2875] | 0.3% |
| Todys et al, 2015 | 206 | 1027 | 0.2006 | [0.1765; 0.2264] | 0.3% |
| Meader et al, 2008 | 10 | 413 | 0.0242 | [0.0117; 0.0441] | 0.3% |
| Tabili et al, 2013 | 23 | 103 | 0.2233 | [0.1471; 0.3160] | 0.3% |
| Pilakasiri et al, 2009 | 44 | 381 | 0.1155 | [0.0852; 0.1519] | 0.3% |
| Ghorbani et al, 2007 | 9 | 800 | 0.0112 | [0.0052; 0.0212] | 0.3% |
| Bura et al, 2018 | 2 | 138 | 0.0145 | [0.0018; 0.0514] | 0.3% |
| Dremsek et al, 2012 | 132 | 853 | 0.1547 | [0.1311; 0.1808] | 0.3% |
| Eick et al, 2010 | 16 | 1500 | 0.0107 | [0.0061; 0.0173] | 0.4% |
| Chaussade et al, 2013 | 83 | 322 | 0.2578 | [0.2109; 0.3092] | 0.3% |
| Zeng et al, 2014 | 26 | 513 | 0.0507 | [0.0334; 0.0734] | 0.3% |
| Yu et al, 2009 | 841 | 3994 | 0.2106 | [0.1980; 0.2235] | 0.4% |
| Teixeira et al, 2017 | 106 | 804 | 0.1318 | [0.1092; 0.1572] | 0.3% |
| Widasari et al, 2013 | 16 | 277 | 0.0578 | [0.0334; 0.0921] | 0.3% |
| Zhang et al, 2017 | 236 | 600 | 0.3933 | [0.3540; 0.4337] | 0.3% |
| Bouwknegt et al, 2008 | 12 | 644 | 0.0186 | [0.0097; 0.0323] | 0.3% |
| Galiana et al, 2008 | 4 | 97 | 0.0412 | [0.0113; 0.1022] | 0.3% |
| Dalton et al, 2017 | 11 | 464 | 0.0237 | [0.0119; 0.0420] | 0.3% |
| Cheng et al, 2007 | 109 | 754 | 0.1446 | [0.1202; 0.1717] | 0.3% |
| Pathak et al, 2018 | 341 | 1369 | 0.2491 | [0.2264; 0.2729] | 0.4% |
| Cong et al, 2014 | 208 | 890 | 0.2337 | [0.2063; 0.2629] | 0.3% |
| Terzic et al, 2009 | 24 | 400 | 0.0600 | [0.0388; 0.0880] | 0.3% |
| Bayhan et al, 2016 | 17 | 408 | 0.0417 | [0.0245; 0.0659] | 0.3% |
| Achwan et al, 2007 | 34 | 581 | 0.0585 | [0.0409; 0.0808] | 0.3% |
| Buti et al, 2008 | 57 | 1249 | 0.0456 | [0.0347; 0.0587] | 0.4% |
| Cevahir et al, 2013 | 23 | 185 | 0.1243 | [0.0805; 0.1807] | 0.3% |
| Davaalkham et al, 2009 | 3 | 520 | 0.0058 | [0.0012; 0.0168] | 0.3% |
| Ekanem et al, 2015 | 31 | 402 | 0.0771 | [0.0530; 0.1077] | 0.3% |
| Hasan et al, 2016 | 33 | 123 | 0.2683 | [0.1924; 0.3557] | 0.3% |
| Kaya et al, 2007 | 2 | 589 | 0.0034 | [0.0004; 0.0122] | 0.3% |
| Krumbholz et al, 2014 | 16 | 1646 | 0.0097 | [0.0056; 0.0157] | 0.4% |
| Meng et al, 2015 | 174 | 1500 | 0.1160 | [0.1002; 0.1333] | 0.4% |
| Oliveira et al, 2017 | 4 | 352 | 0.0114 | [0.0031; 0.0288] | 0.3% |
| Shamsizadeh et al, 2009 | 48 | 566 | 0.0848 | [0.0632; 0.1109] | 0.3% |
| Bira et al, 2007 | 5 | 717 | 0.0070 | [0.0023; 0.0162] | 0.3% |
| Saffar et al, 2009 | 25 | 1080 | 0.0231 | [0.0150; 0.0340] | 0.3% |
| Bawazir et al, 2010 | 38 | 356 | 0.1067 | [0.0767; 0.1436] | 0.3% |
| Dong et al, 2007 | 2073 | 12052 | 0.1720 | [0.1653; 0.1789] | 0.4% |
| Lu et al, 2009 | 1547 | 8762 | 0.1766 | [0.1686; 0.1847] | 0.4% |
| Ijaz et al, 2009 | 362 | 2731 | 0.1326 | [0.1201; 0.1458] | 0.4% |
| Héctor et al, 2017 | 1 | 127 | 0.0079 | [0.0002; 0.0431] | 0.3% |
| Li et al, 2019 | 139 | 946 | 0.1469 | [0.1250; 0.1711] | 0.3% |
| Lee et al, 2013 | 2 | 100 | 0.0200 | [0.0024; 0.0704] | 0.3% |
| Dorcas et al, 2018 | 48 | 393 | 0.1221 | [0.0914; 0.1587] | 0.3% |
| Rui et al, 2018 | 49 | 225 | 0.2178 | [0.1657; 0.2775] | 0.3% |
| Maria et al, 2010 | 38 | 1040 | 0.0365 | [0.0260; 0.0498] | 0.3% |
| Zakieh et al, 2013 | 5 | 136 | 0.0368 | [0.0120; 0.0837] | 0.3% |
| Niguse et al, 2018 | 359 | 846 | 0.4243 | [0.3908; 0.4585] | 0.3% |
| Caron et al, 2008 | 119 | 840 | 0.1417 | [0.1188; 0.1671] | 0.3% |
| Caron et al, 2012 | 16 | 243 | 0.0658 | [0.0381; 0.1047] | 0.3% |
| Cosme et al, 2014 | 25 | 439 | 0.0569 | [0.0372; 0.0829] | 0.3% |
| Farshadpour et al, 2018 | 83 | 1331 | 0.0624 | [0.0500; 0.0767] | 0.4% |
| Gad et al, 2011 | 68 | 116 | 0.5862 | [0.4910; 0.6769] | 0.3% |
| Adjei et al, 2009 | 16 | 157 | 0.1019 | [0.0594; 0.1602] | 0.3% |
| Huang et al, 2013 | 30 | 293 | 0.1024 | [0.0702; 0.1429] | 0.3% |
| Paschale et al, 2016 | 62 | 278 | 0.2230 | [0.1755; 0.2766] | 0.3% |
| Alizadeh et al, 2016 | 10 | 321 | 0.0312 | [0.0150; 0.0565] | 0.3% |
| Abebe et al, 2017 | 122 | 386 | 0.3161 | [0.2700; 0.3650] | 0.3% |
| Traore et al, 2012 | 56 | 178 | 0.3146 | [0.2472; 0.3883] | 0.3% |
| Zhang et al, 2015 | 1352 | 6262 | 0.2159 | [0.2058; 0.2263] | 0.4% |
| Kyvernitakis et al, 2015 | 12 | 115 | 0.1043 | [0.0551; 0.1752] | 0.3% |
| Tashale et al, 2015 | 1081 | 13851 | 0.0780 | [0.0736; 0.0826] | 0.4% |
| Xu et al,2013 | 364 | 1939 | 0.1877 | [0.1706; 0.2058] | 0.4% |
| Stramer et al, 2015 | 27 | 200 | 0.1350 | [0.0909; 0.1903] | 0.3% |
| Esquivel et al, 2015 | 10 | 150 | 0.0667 | [0.0324; 0.1192] | 0.3% |
| Esquivel et al, 2015 | 25 | 439 | 0.0569 | [0.0372; 0.0829] | 0.3% |
| Wassaf et al,2014 | 19 | 433 | 0.0439 | [0.0266; 0.0677] | 0.3% |
| Munne et al, 2014 | 31 | 202 | 0.1535 | [0.1067; 0.2107] | 0.3% |
| Katrina et al, 2018 | 104 | 1714 | 0.0607 | [0.0498; 0.0730] | 0.4% |
| Taha et al, 2015 | 80 | 397 | 0.2015 | [0.1631; 0.2444] | 0.3% |
| **Random effects model** | | 1019719 | 0.1365 | [0.1115; 0.1635] | 73.5% |
| Heterogeneity: $I^2 = 100\%$, $\tau^2 = 0.0785$, $p = 0$ | | | | | |
| **Random effects model** | | **1099717** | **0.1247** | **[0.1042; 0.1467]** | **100.0%** |
| Heterogeneity: $I^2 = 100\%$, $\tau^2 = 0.0769$, $p = 0$ | | | | | |
| Residual heterogeneity: $I^2 = 100\%$, $p = 0$ | | | | | |

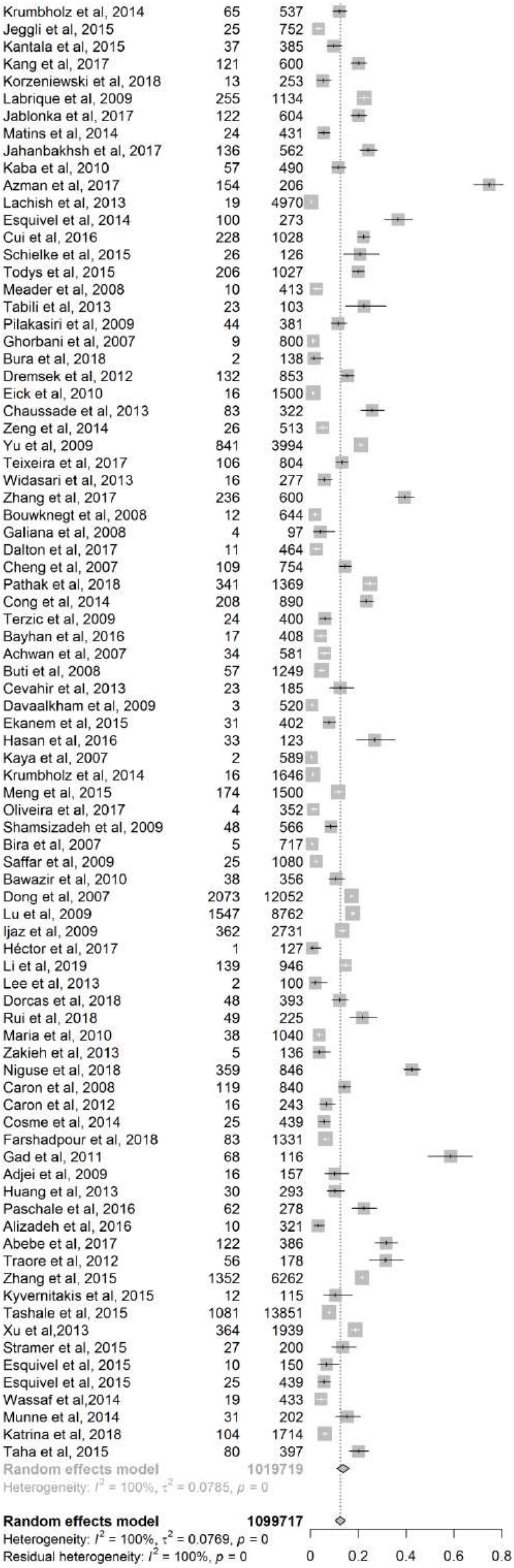

**Figure S13. Forest plot of estimated pooled anti-HEV IgG seroprevalence among swine-related occupational population based on different countries.**

Note: country named multiple means people in the study are from more than one country.

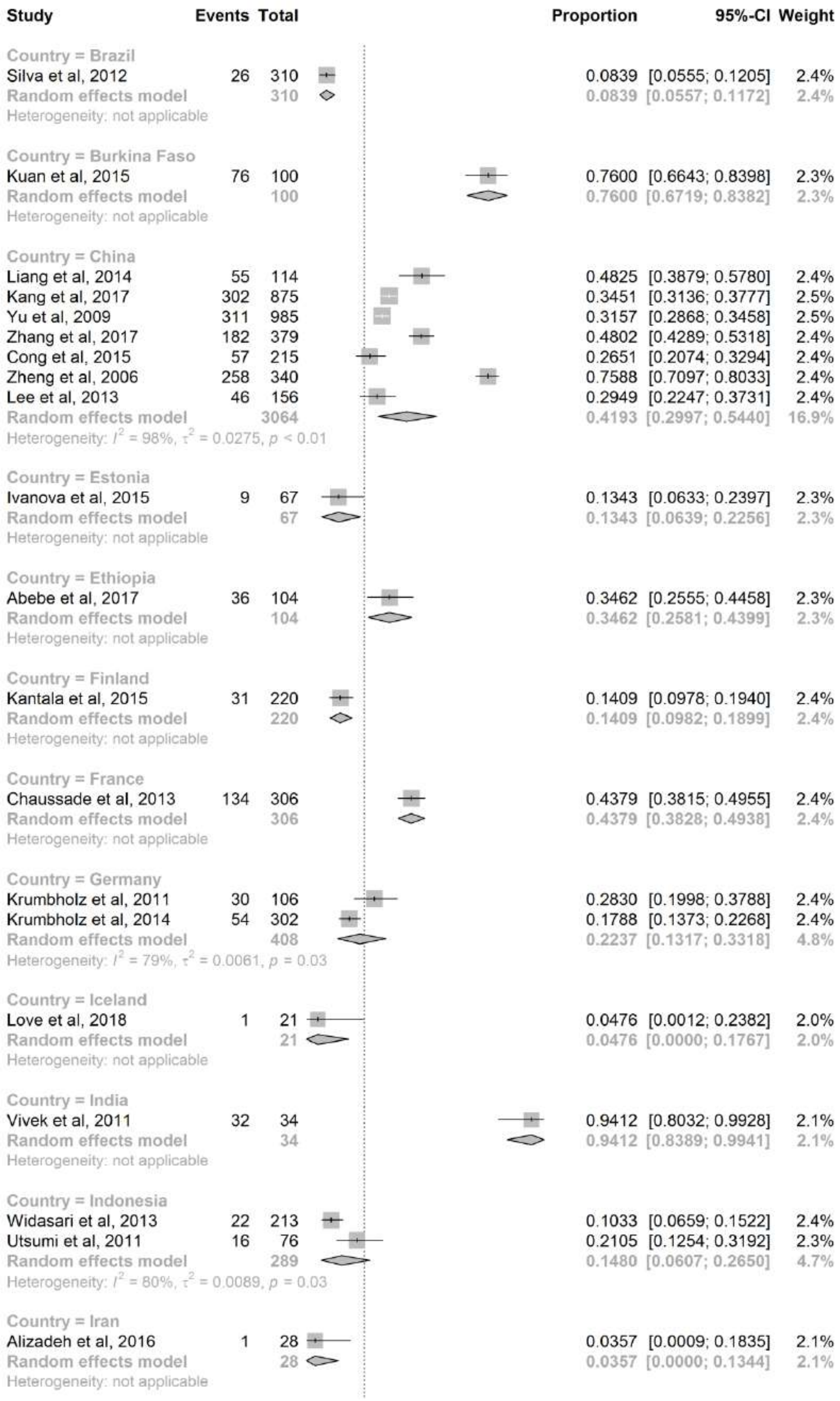

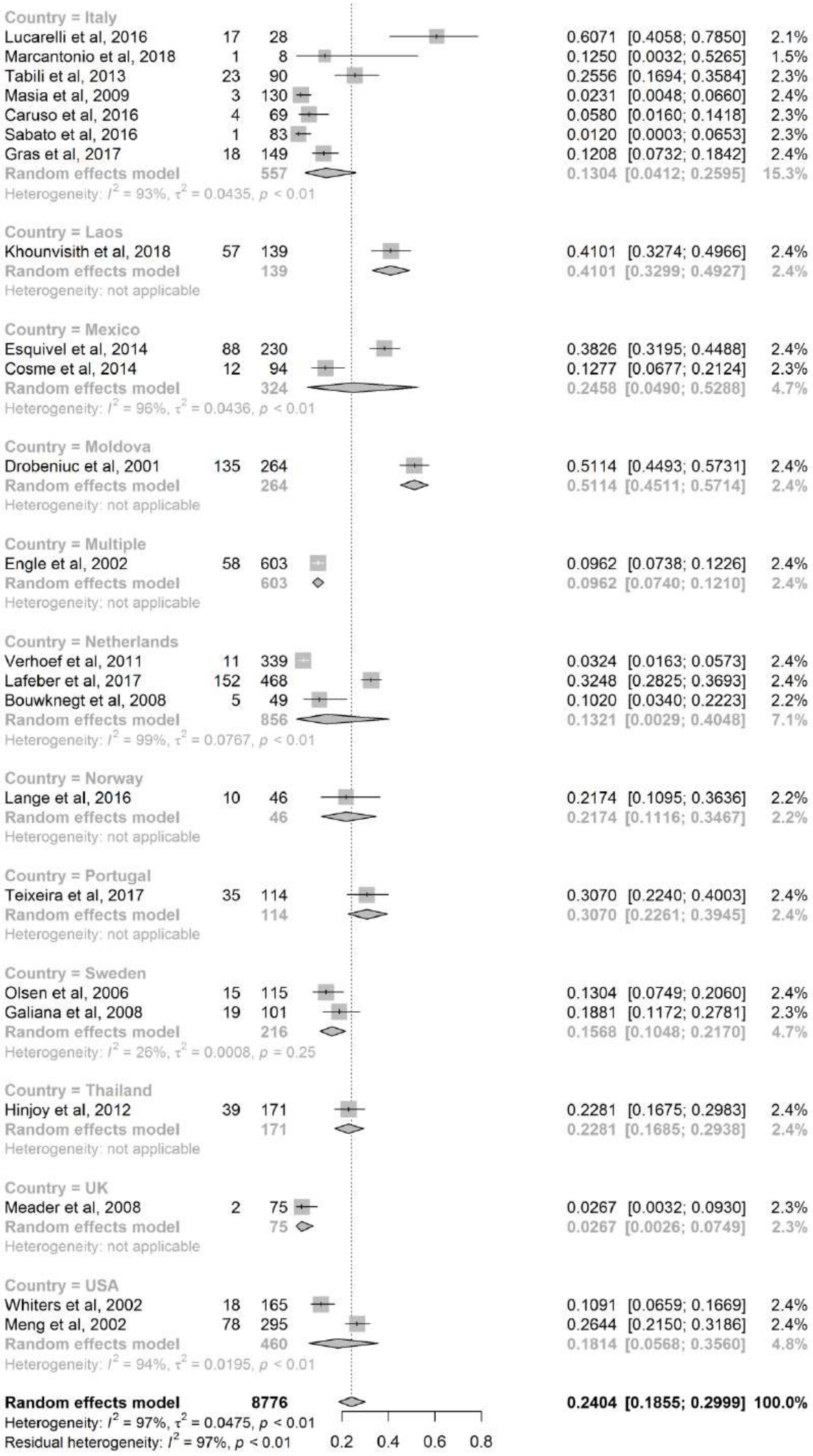
Country = Italy
Lucarelli et al, 2016 17 28 0.6071 [0.4058; 0.7850] 2.1%
Marcantonio et al, 2018 1 8 0.1250 [0.0032; 0.5265] 1.5%
Tabili et al, 2013 23 90 0.2556 [0.1694; 0.3584] 2.3%
Masia et al, 2009 3 130 0.0231 [0.0048; 0.0660] 2.4%
Caruso et al, 2016 4 69 0.0580 [0.0160; 0.1418] 2.3%
Sabato et al, 2016 1 83 0.0120 [0.0003; 0.0653] 2.3%
Gras et al, 2017 18 149 0.1208 [0.0732; 0.1842] 2.4%
Random effects model 557 0.1304 [0.0412; 0.2595] 15.3%
Heterogeneity: $I^2$ = 93%, $\tau^2$ = 0.0435, $p$ < 0.01
Country = Laos
Khounvisith et al, 2018 57 139 0.4101 [0.3274; 0.4966] 2.4%
Random effects model 139 0.4101 [0.3299; 0.4927] 2.4%
Heterogeneity: not applicable
Country = Mexico
Esquivel et al, 2014 88 230 0.3826 [0.3195; 0.4488] 2.4%
Cosme et al, 2014 12 94 0.1277 [0.0677; 0.2124] 2.3%
Random effects model 324 0.2458 [0.0490; 0.5288] 4.7%
Heterogeneity: $I^2$ = 96%, $\tau^2$ = 0.0436, $p$ < 0.01
Country = Moldova
Drobeniuc et al, 2001 135 264 0.5114 [0.4493; 0.5731] 2.4%
Random effects model 264 0.5114 [0.4511; 0.5714] 2.4%
Heterogeneity: not applicable
Country = Multiple
Engle et al, 2002 58 603 0.0962 [0.0738; 0.1226] 2.4%
Random effects model 603 0.0962 [0.0740; 0.1210] 2.4%
Heterogeneity: not applicable
Country = Netherlands
Verhoef et al, 2011 11 339 0.0324 [0.0163; 0.0573] 2.4%
Lafeber et al, 2017 152 468 0.3248 [0.2825; 0.3693] 2.4%
Bouwknegt et al, 2008 5 49 0.1020 [0.0340; 0.2223] 2.2%
Random effects model 856 0.1321 [0.0029; 0.4048] 7.1%
Heterogeneity: $I^2$ = 99%, $\tau^2$ = 0.0767, $p$ < 0.01
Country = Norway
Lange et al, 2016 10 46 0.2174 [0.1095; 0.3636] 2.2%
Random effects model 46 0.2174 [0.1116; 0.3467] 2.2%
Heterogeneity: not applicable
Country = Portugal
Teixeira et al, 2017 35 114 0.3070 [0.2240; 0.4003] 2.4%
Random effects model 114 0.3070 [0.2261; 0.3945] 2.4%
Heterogeneity: not applicable
Country = Sweden
Olsen et al, 2006 15 115 0.1304 [0.0749; 0.2060] 2.4%
Galiana et al, 2008 19 101 0.1881 [0.1172; 0.2781] 2.3%
Random effects model 216 0.1568 [0.1048; 0.2170] 4.7%
Heterogeneity: $I^2$ = 26%, $\tau^2$ = 0.0008, $p$ = 0.25
Country = Thailand
Hinjoy et al, 2012 39 171 0.2281 [0.1675; 0.2983] 2.4%
Random effects model 171 0.2281 [0.1685; 0.2938] 2.4%
Heterogeneity: not applicable
Country = UK
Meader et al, 2008 2 75 0.0267 [0.0032; 0.0930] 2.3%
Random effects model 75 0.0267 [0.0026; 0.0749] 2.3%
Heterogeneity: not applicable
Country = USA
Whiters et al, 2002 18 165 0.1091 [0.0659; 0.1669] 2.4%
Meng et al, 2002 78 295 0.2644 [0.2150; 0.3186] 2.4%
Random effects model 460 0.1814 [0.0568; 0.3560] 4.8%
Heterogeneity: $I^2$ = 94%, $\tau^2$ = 0.0195, $p$ < 0.01
Random effects model 8776 0.2404 [0.1855; 0.2999] 100.0%
Heterogeneity: $I^2$ = 97%, $\tau^2$ = 0.0475, $p$ < 0.01
Residual heterogeneity: $I^2$ = 97%, $p$ < 0.01
0.2 0.4 0.6 0.8

**Figure S14. Forest plot of estimated pooled anti-HEV IgG seroprevalence among special population.**

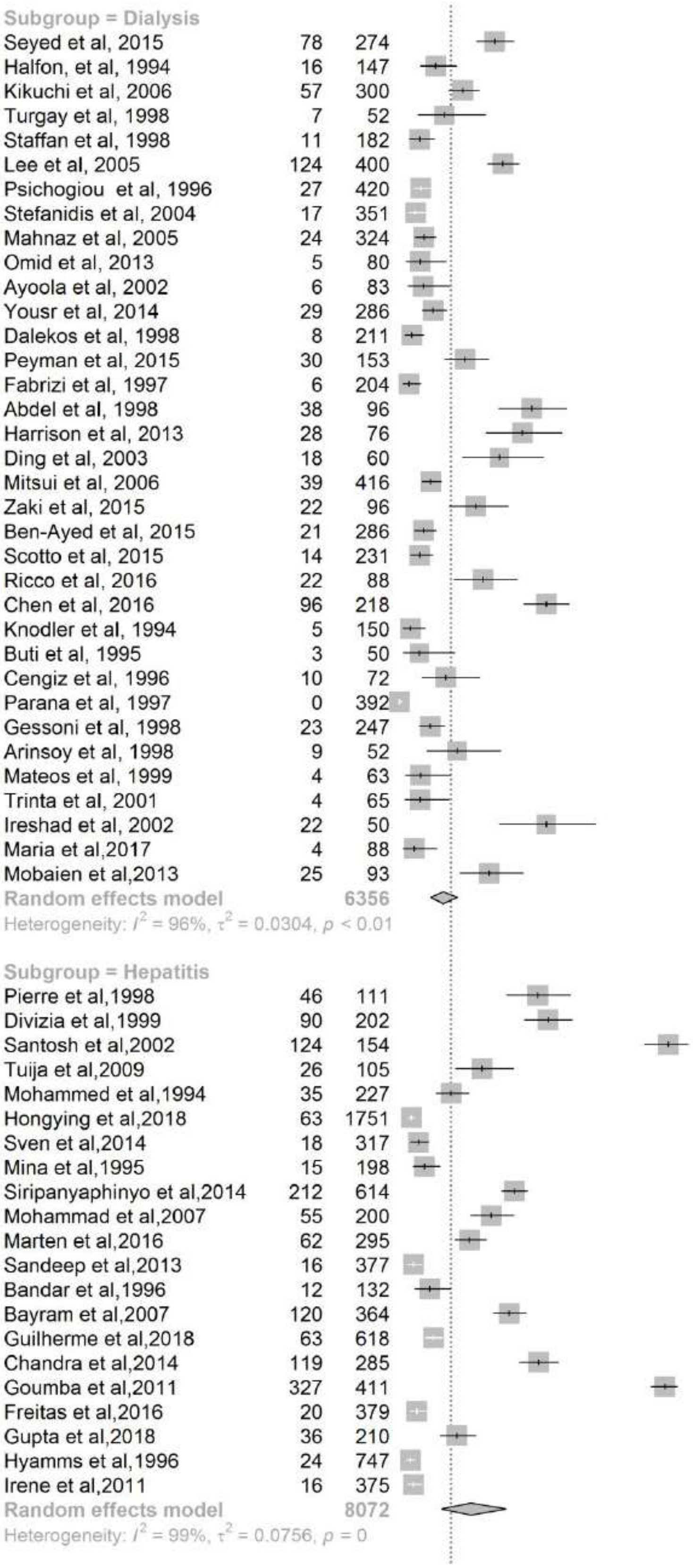

| Study | Events | Total | Proportion | 95%-CI | Weight |
|---|---|---|---|---|---|
| Subgroup = Dialysis | | | | | |
| Seyed et al, 2015 | 78 | 274 | 0.2847 | [0.2320; 0.3421] | 0.8% |
| Halfon, et al, 1994 | 16 | 147 | 0.1088 | [0.0635; 0.1707] | 0.8% |
| Kikuchi et al, 2006 | 57 | 300 | 0.1900 | [0.1472; 0.2390] | 0.8% |
| Turgay et al, 1998 | 7 | 52 | 0.1346 | [0.0559; 0.2579] | 0.7% |
| Staffan et al, 1998 | 11 | 182 | 0.0604 | [0.0306; 0.1056] | 0.8% |
| Lee et al, 2005 | 124 | 400 | 0.3100 | [0.2650; 0.3579] | 0.8% |
| Psichogiou et al, 1996 | 27 | 420 | 0.0643 | [0.0428; 0.0922] | 0.8% |
| Stefanidis et al, 2004 | 17 | 351 | 0.0484 | [0.0285; 0.0764] | 0.8% |
| Mahnaz et al, 2005 | 24 | 324 | 0.0741 | [0.0480; 0.1082] | 0.8% |
| Omid et al, 2013 | 5 | 80 | 0.0625 | [0.0206; 0.1399] | 0.8% |
| Ayoola et al, 2002 | 6 | 83 | 0.0723 | [0.0270; 0.1507] | 0.8% |
| Yousr et al, 2014 | 29 | 286 | 0.1014 | [0.0690; 0.1424] | 0.8% |
| Dalekos et al, 1998 | 8 | 211 | 0.0379 | [0.0165; 0.0733] | 0.8% |
| Peyman et al, 2015 | 30 | 153 | 0.1961 | [0.1364; 0.2679] | 0.8% |
| Fabrizi et al, 1997 | 6 | 204 | 0.0294 | [0.0109; 0.0629] | 0.8% |
| Abdel et al, 1998 | 38 | 96 | 0.3958 | [0.2975; 0.5008] | 0.8% |
| Harrison et al, 2013 | 28 | 76 | 0.3684 | [0.2606; 0.4869] | 0.8% |
| Ding et al, 2003 | 18 | 60 | 0.3000 | [0.1885; 0.4321] | 0.7% |
| Mitsui et al, 2006 | 39 | 416 | 0.0938 | [0.0675; 0.1259] | 0.8% |
| Zaki et al, 2015 | 22 | 96 | 0.2292 | [0.1495; 0.3261] | 0.8% |
| Ben-Ayed et al, 2015 | 21 | 286 | 0.0734 | [0.0460; 0.1101] | 0.8% |
| Scotto et al, 2015 | 14 | 231 | 0.0606 | [0.0335; 0.0996] | 0.8% |
| Ricco et al, 2016 | 22 | 88 | 0.2500 | [0.1638; 0.3537] | 0.8% |
| Chen et al, 2016 | 96 | 218 | 0.4404 | [0.3734; 0.5090] | 0.8% |
| Knodler et al, 1994 | 5 | 150 | 0.0333 | [0.0109; 0.0761] | 0.8% |
| Buti et al, 1995 | 3 | 50 | 0.0600 | [0.0125; 0.1655] | 0.7% |
| Cengiz et al, 1996 | 10 | 72 | 0.1389 | [0.0687; 0.2406] | 0.8% |
| Parana et al, 1997 | 0 | 392 | 0.0000 | [0.0000; 0.0094] | 0.8% |
| Gessoni et al, 1998 | 23 | 247 | 0.0931 | [0.0599; 0.1364] | 0.8% |
| Arinsoy et al, 1998 | 9 | 52 | 0.1731 | [0.0823; 0.3033] | 0.7% |
| Mateos et al, 1999 | 4 | 63 | 0.0635 | [0.0176; 0.1547] | 0.8% |
| Trinta et al, 2001 | 4 | 65 | 0.0615 | [0.0170; 0.1501] | 0.8% |
| Ireshad et al, 2002 | 22 | 50 | 0.4400 | [0.2999; 0.5875] | 0.7% |
| Maria et al,2017 | 4 | 88 | 0.0455 | [0.0125; 0.1123] | 0.8% |
| Mobaien et al,2013 | 25 | 93 | 0.2688 | [0.1821; 0.3708] | 0.8% |
| Random effects model | | 6356 | 0.1310 | [0.0934; 0.1739] | 27.4% |
| Heterogeneity: $I^2 = 96\%$, $\tau^2 = 0.0304$, $p < 0.01$ | | | | | |
| Subgroup = Hepatitis | | | | | |
| Pierre et al,1998 | 46 | 111 | 0.4144 | [0.3217; 0.5118] | 0.8% |
| Divizia et al,1999 | 90 | 202 | 0.4455 | [0.3758; 0.5169] | 0.8% |
| Santosh et al,2002 | 124 | 154 | 0.8052 | [0.7337; 0.8645] | 0.8% |
| Tuija et al,2009 | 26 | 105 | 0.2476 | [0.1686; 0.3414] | 0.8% |
| Mohammed et al,1994 | 35 | 227 | 0.1542 | [0.1098; 0.2079] | 0.8% |
| Hongying et al,2018 | 63 | 1751 | 0.0360 | [0.0278; 0.0458] | 0.8% |
| Sven et al,2014 | 18 | 317 | 0.0568 | [0.0340; 0.0883] | 0.8% |
| Mina et al,1995 | 15 | 198 | 0.0758 | [0.0430; 0.1219] | 0.8% |
| Siripanyaphinyo et al,2014 | 212 | 614 | 0.3453 | [0.3077; 0.3844] | 0.8% |
| Mohammad et al,2007 | 55 | 200 | 0.2750 | [0.2144; 0.3424] | 0.8% |
| Marten et al,2016 | 62 | 295 | 0.2102 | [0.1651; 0.2612] | 0.8% |
| Sandeep et al,2013 | 16 | 377 | 0.0424 | [0.0244; 0.0680] | 0.8% |
| Bandar et al,1996 | 12 | 132 | 0.0909 | [0.0479; 0.1534] | 0.8% |
| Bayram et al,2007 | 120 | 364 | 0.3297 | [0.2816; 0.3806] | 0.8% |
| Guilherme et al,2018 | 63 | 618 | 0.1019 | [0.0792; 0.1285] | 0.8% |
| Chandra et al,2014 | 119 | 285 | 0.4175 | [0.3597; 0.4772] | 0.8% |
| Goumba et al,2011 | 327 | 411 | 0.7956 | [0.7534; 0.8336] | 0.8% |
| Freitas et al,2016 | 20 | 379 | 0.0528 | [0.0325; 0.0803] | 0.8% |
| Gupta et al,2018 | 36 | 210 | 0.1714 | [0.1230; 0.2293] | 0.8% |
| Hyamms et al,1996 | 24 | 747 | 0.0321 | [0.0207; 0.0474] | 0.8% |
| Irene et al,2011 | 16 | 375 | 0.0427 | [0.0246; 0.0684] | 0.8% |
| Random effects model | | 8072 | 0.2149 | [0.1265; 0.3192] | 16.8% |
| Heterogeneity: $I^2 = 99\%$, $\tau^2 = 0.0756$, $p = 0$ | | | | | |

| Study | Events | Total | Proportion | 95% CI | Weight |
|---|---|---|---|---|---|
| Subgroup = HIV | | | | | |
| Maciej et al,2017 | 124 | 244 | 0.5082 | [0.4437; 0.5725] | 0.8% |
| Lene et al,2018 | 16 | 200 | 0.0800 | [0.0464; 0.1267] | 0.8% |
| Michelle et al,2014 | 12 | 191 | 0.0628 | [0.0329; 0.1072] | 0.8% |
| Sarah et al,2012 | 4 | 261 | 0.0153 | [0.0042; 0.0388] | 0.8% |
| Huang et al,2017 | 252 | 639 | 0.3944 | [0.3563; 0.4335] | 0.8% |
| Ananta et al,2017 | 181 | 459 | 0.3943 | [0.3493; 0.4407] | 0.8% |
| Mamadou et al,2011 | 8 | 184 | 0.0435 | [0.0190; 0.0839] | 0.8% |
| Antonio et al,2014 | 88 | 894 | 0.0984 | [0.0797; 0.1199] | 0.8% |
| Gaetano et al,2015 | 34 | 509 | 0.0668 | [0.0467; 0.0921] | 0.8% |
| Hassan et al,2015 | 26 | 158 | 0.1646 | [0.1104; 0.2317] | 0.8% |
| Marianna et al,2015 | 18 | 243 | 0.0741 | [0.0445; 0.1145] | 0.8% |
| Juan et al,2014 | 161 | 613 | 0.2626 | [0.2282; 0.2994] | 0.8% |
| Alain et al,2011 | 19 | 735 | 0.0259 | [0.0156; 0.0401] | 0.8% |
| Kenneth et al,2014 | 32 | 166 | 0.1928 | [0.1357; 0.2611] | 0.8% |
| Sonia et al,2019 | 42 | 234 | 0.1795 | [0.1325; 0.2348] | 0.8% |
| Jardi et al,2012 | 22 | 238 | 0.0924 | [0.0588; 0.1366] | 0.8% |
| Kenta et al,2016 | 99 | 208 | 0.4760 | [0.4065; 0.5462] | 0.8% |
| Shiyi et al,2018 | 342 | 770 | 0.4442 | [0.4087; 0.4801] | 0.8% |
| Keane et al,2011 | 13 | 138 | 0.0942 | [0.0511; 0.1557] | 0.8% |
| Rivero-Juarez et al,2016 | 189 | 894 | 0.2114 | [0.1851; 0.2397] | 0.8% |
| Ng et al,2000 | 15 | 145 | 0.1034 | [0.0591; 0.1649] | 0.8% |
| Amitis et al,2013 | 10 | 100 | 0.1000 | [0.0490; 0.1762] | 0.8% |
| Pischke et al,2015 | 64 | 246 | 0.2602 | [0.2065; 0.3197] | 0.8% |
| Balayan et al,1997 | 13 | 117 | 0.1111 | [0.0605; 0.1825] | 0.8% |
| Denali et al,2018 | 229 | 494 | 0.4636 | [0.4189; 0.5087] | 0.8% |
| Torsten et al,2013 | 182 | 402 | 0.4527 | [0.4033; 0.5028] | 0.8% |
| Torsten et al,2013 | 41 | 289 | 0.1419 | [0.1038; 0.1875] | 0.8% |
| Ferreira et al,2017 | 38 | 354 | 0.1073 | [0.0771; 0.1444] | 0.8% |
| Florence et al,2016 | 116 | 300 | 0.3867 | [0.3313; 0.4443] | 0.8% |
| Rode et al,2014 | 51 | 504 | 0.1012 | [0.0763; 0.1309] | 0.8% |
| Luan et al,2018 | 15 | 366 | 0.0410 | [0.0231; 0.0667] | 0.8% |
| Christophe et al, 2010 | 22 | 245 | 0.0898 | [0.0571; 0.1328] | 0.8% |
| Lindemann et al, 2013 | 45 | 448 | 0.1004 | [0.0742; 0.1321] | 0.8% |
| Caron et al, 2012 | 13 | 183 | 0.0710 | [0.0384; 0.1184] | 0.8% |
| Barciela et al, 2014 | 22 | 238 | 0.0924 | [0.0588; 0.1366] | 0.8% |
| Sellier et al, 2011 | 4 | 108 | 0.0370 | [0.0102; 0.0921] | 0.8% |
| Hassing et al, 2014 | 30 | 256 | 0.1172 | [0.0805; 0.1631] | 0.8% |
| Nouhin et al, 2015 | 247 | 825 | 0.2994 | [0.2683; 0.3319] | 0.8% |
| Cianflone et al, 2012 | 7 | 194 | 0.0361 | [0.0146; 0.0729] | 0.8% |
| Fainboim et al, 2002 | 32 | 484 | 0.0661 | [0.0457; 0.0921] | 0.8% |
| Taha et al, 2015 | 52 | 403 | 0.1290 | [0.0979; 0.1657] | 0.8% |
| Alberts et al, 2018 | 20 | 98 | 0.2041 | [0.1293; 0.2974] | 0.8% |
| Sibiro et al, 2018 | 136 | 200 | 0.6800 | [0.6105; 0.7440] | 0.8% |
| Shadmand et al, 2018 | 26 | 73 | 0.3562 | [0.2475; 0.4769] | 0.8% |
| Random effects model | | 15050 | 0.1691 | [0.1264; 0.2167] | 35.2% |
| Heterogeneity: $I^2 = 98\%$, $\tau^2 = 0.0407$, $p = 0$ | | | | | |
| Subgroup = Transplant | | | | | |
| Gaetano et al, 2015 | 4 | 120 | 0.0333 | [0.0092; 0.0831] | 0.8% |
| Maria et al, 2017 | 7 | 120 | 0.0583 | [0.0238; 0.1165] | 0.8% |
| Koji et al, 2018 | 8 | 184 | 0.0435 | [0.0190; 0.0839] | 0.8% |
| Alberto et al, 2016 | 32 | 333 | 0.0961 | [0.0667; 0.1329] | 0.8% |
| Naik et al, 2012 | 52 | 205 | 0.2537 | [0.1956; 0.3190] | 0.8% |
| Florence et al, 2011 | 89 | 700 | 0.1271 | [0.1034; 0.1541] | 0.8% |
| Valérie et al, 2013 | 13 | 160 | 0.0812 | [0.0440; 0.1349] | 0.8% |
| Ludi et al, 2015 | 61 | 145 | 0.4207 | [0.3393; 0.5054] | 0.8% |
| Yuki et al, 2015 | 54 | 1893 | 0.0285 | [0.0215; 0.0371] | 0.8% |
| Jerusa et al, 2018 | 8 | 316 | 0.0253 | [0.0110; 0.0493] | 0.8% |
| Vincent et al, 2018 | 68 | 263 | 0.2586 | [0.2067; 0.3159] | 0.8% |
| Florence et al, 2013 | 101 | 263 | 0.3840 | [0.3250; 0.4458] | 0.8% |
| Buffaz et al, 2014 | 60 | 206 | 0.2913 | [0.2302; 0.3584] | 0.8% |
| Elizabeth et al, 2009 | 9 | 285 | 0.0316 | [0.0145; 0.0591] | 0.8% |
| Hoerning et al, 2012 | 4 | 124 | 0.0323 | [0.0089; 0.0805] | 0.8% |
| Mar et al, 2014 | 43 | 634 | 0.0678 | [0.0495; 0.0903] | 0.8% |
| Pischke et al, 2009 | 10 | 226 | 0.0442 | [0.0214; 0.0799] | 0.8% |
| Pischke et al, 2012 | 31 | 274 | 0.1131 | [0.0782; 0.1567] | 0.8% |
| Halac et al, 2011 | 22 | 80 | 0.2750 | [0.1810; 0.3862] | 0.8% |
| Khameneh et al, 2011 | 28 | 91 | 0.3077 | [0.2151; 0.4132] | 0.8% |
| Harrison et al, 2013 | 16 | 88 | 0.1818 | [0.1076; 0.2784] | 0.8% |
| Lim et al, 2017 | 44 | 244 | 0.1803 | [0.1342; 0.2344] | 0.8% |
| Buti et al, 2009 | 3 | 120 | 0.0250 | [0.0052; 0.0713] | 0.8% |
| Laverdure et al, 2015 | 8 | 96 | 0.0833 | [0.0367; 0.1576] | 0.8% |
| Magnusson et al, 2015 | 8 | 62 | 0.1290 | [0.0574; 0.2385] | 0.8% |
| Sue et al, 2016 | 6 | 311 | 0.0193 | [0.0071; 0.0415] | 0.8% |
| Random effects model | | 7543 | 0.1168 | [0.0791; 0.1606] | 20.6% |
| Heterogeneity: $I^2 = 97\%$, $\tau^2 = 0.0259$, $p < 0.01$ | | | | | |
| **Random effects model** | | **37021** | **0.1543** | **[0.1282; 0.1824]** | **100.0%** |
| Heterogeneity: $I^2 = 98\%$, $\tau^2 = 0.0448$, $p = 0$ | | | | | |
| Residual heterogeneity: $I^2 = 98\%$, $p = 0$ | | | | | |

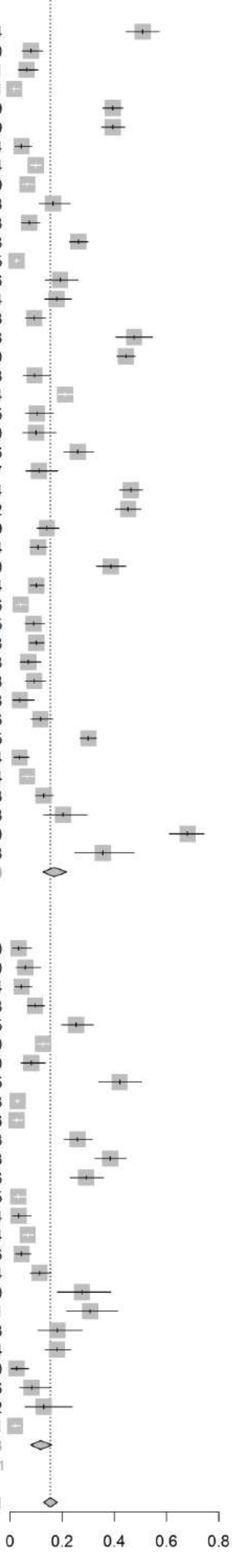

**Figure S15. Forest plot of estimated pooled anti-HEV IgM seroprevalence among special population.**

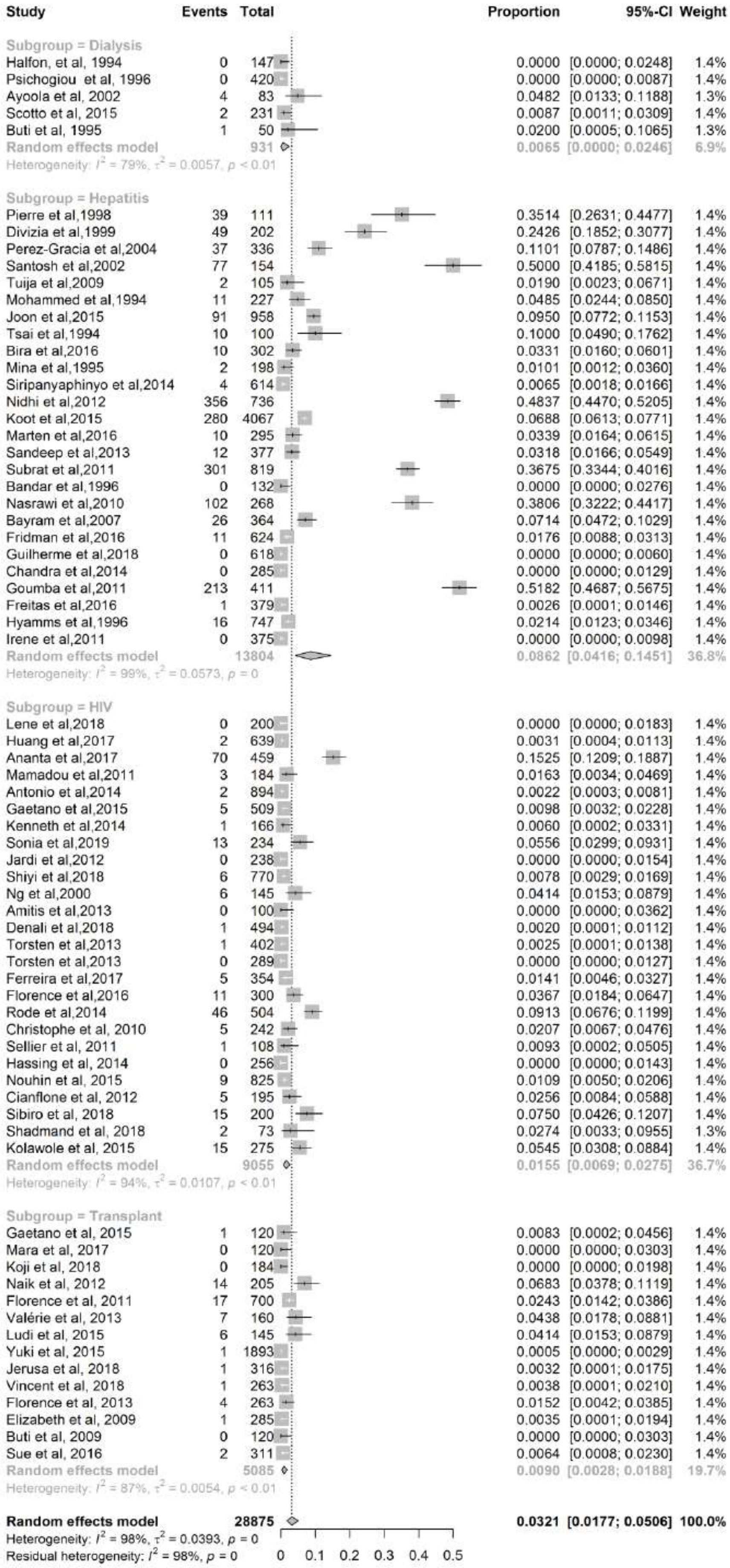

**Figure S16. Forest plot of estimated pooled HEV RNA positive rate among special population.**

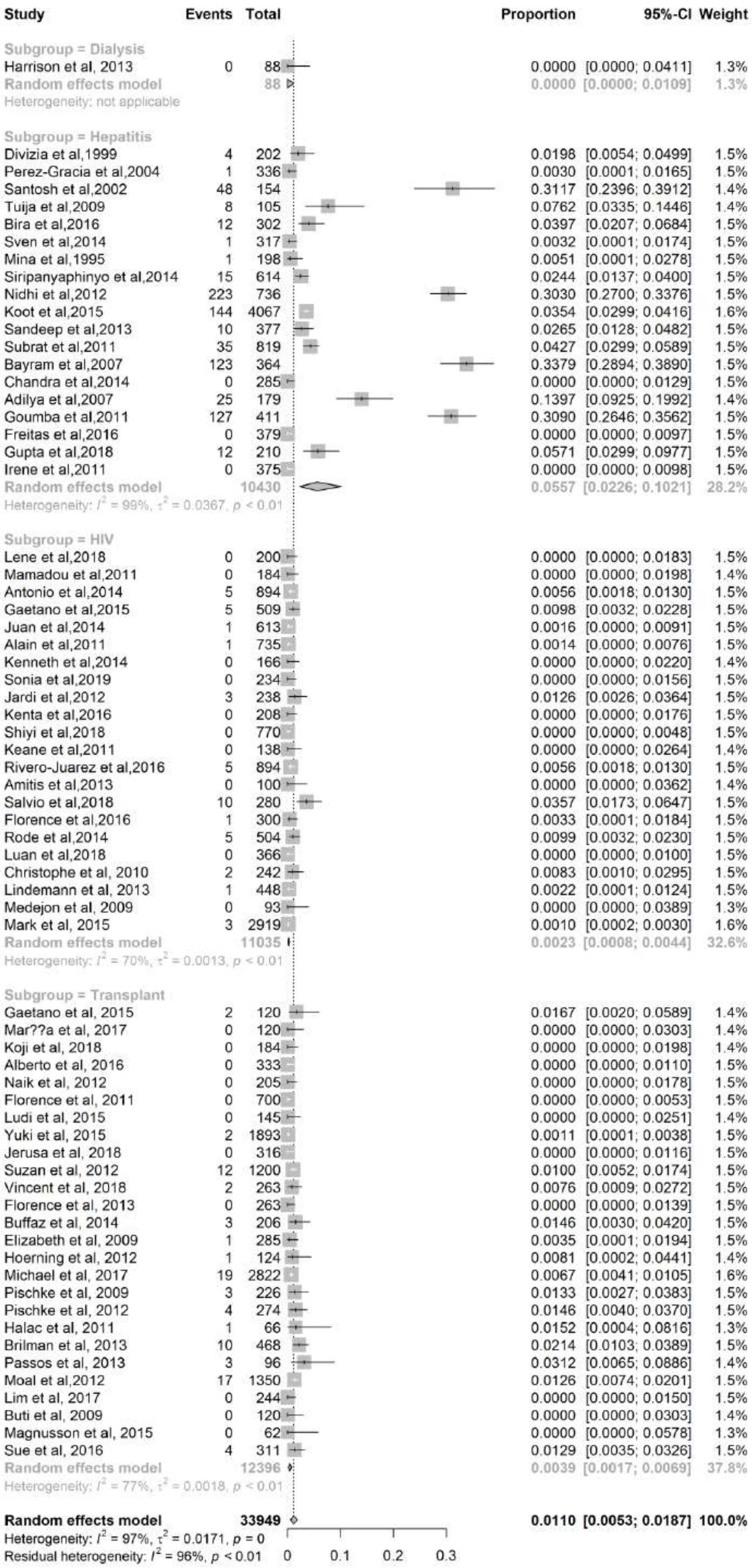

**Figure S17. Forest plot of estimated pooled seroprevalence of anti-HEV IgG among hepatitis population.**

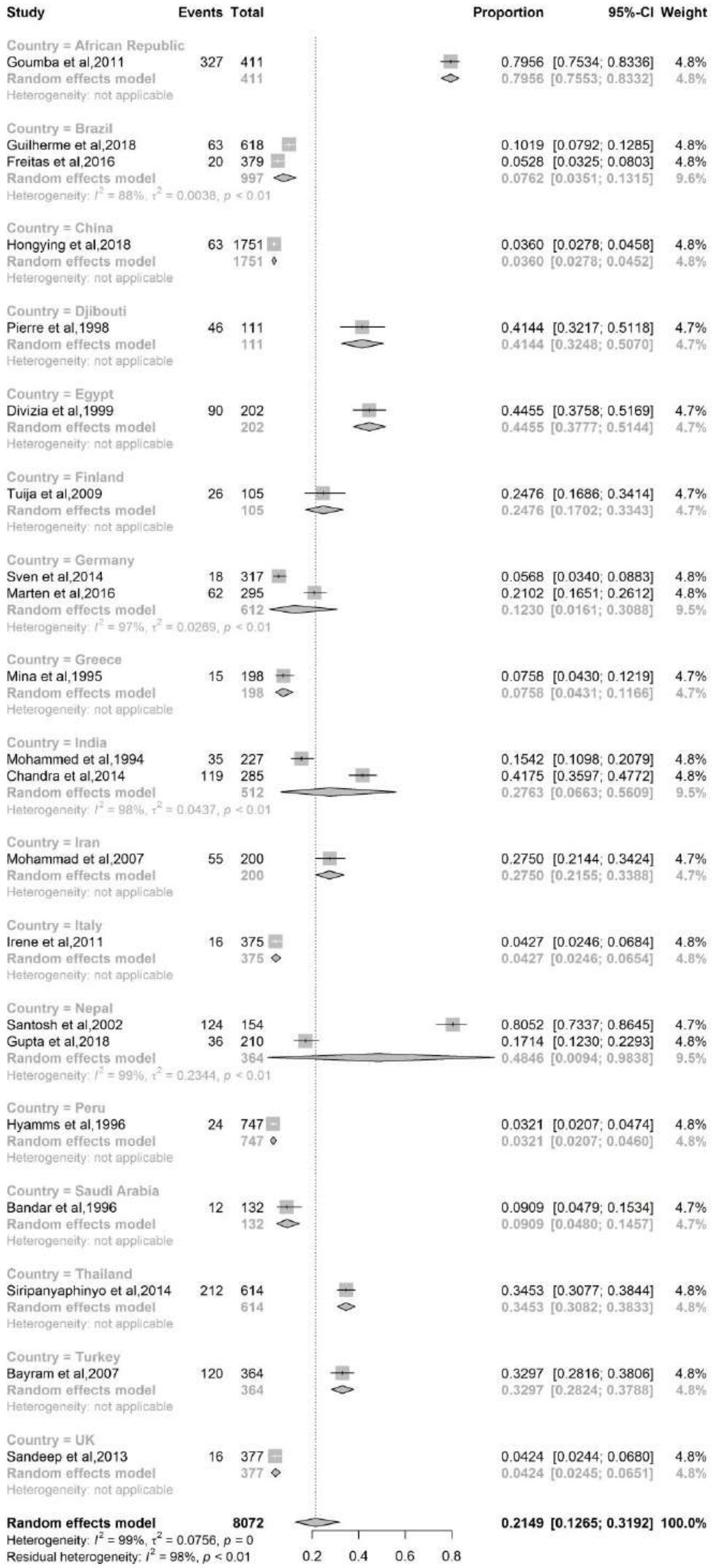

**Figure S18. Forest plot of estimated pooled seroprevalence of anti-HEV IgM among hepatitis population.**

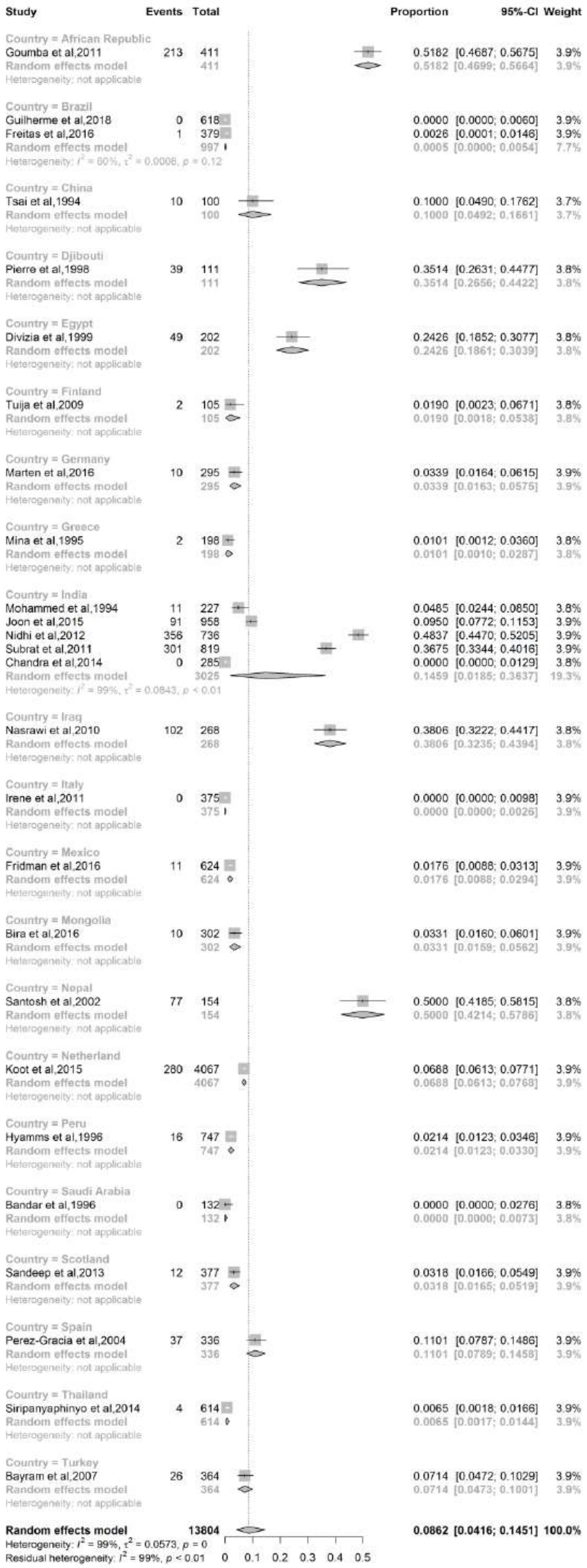

**Figure S19. Forest plot of estimated pooled HEV RNA positive rate among hepatitis population.**

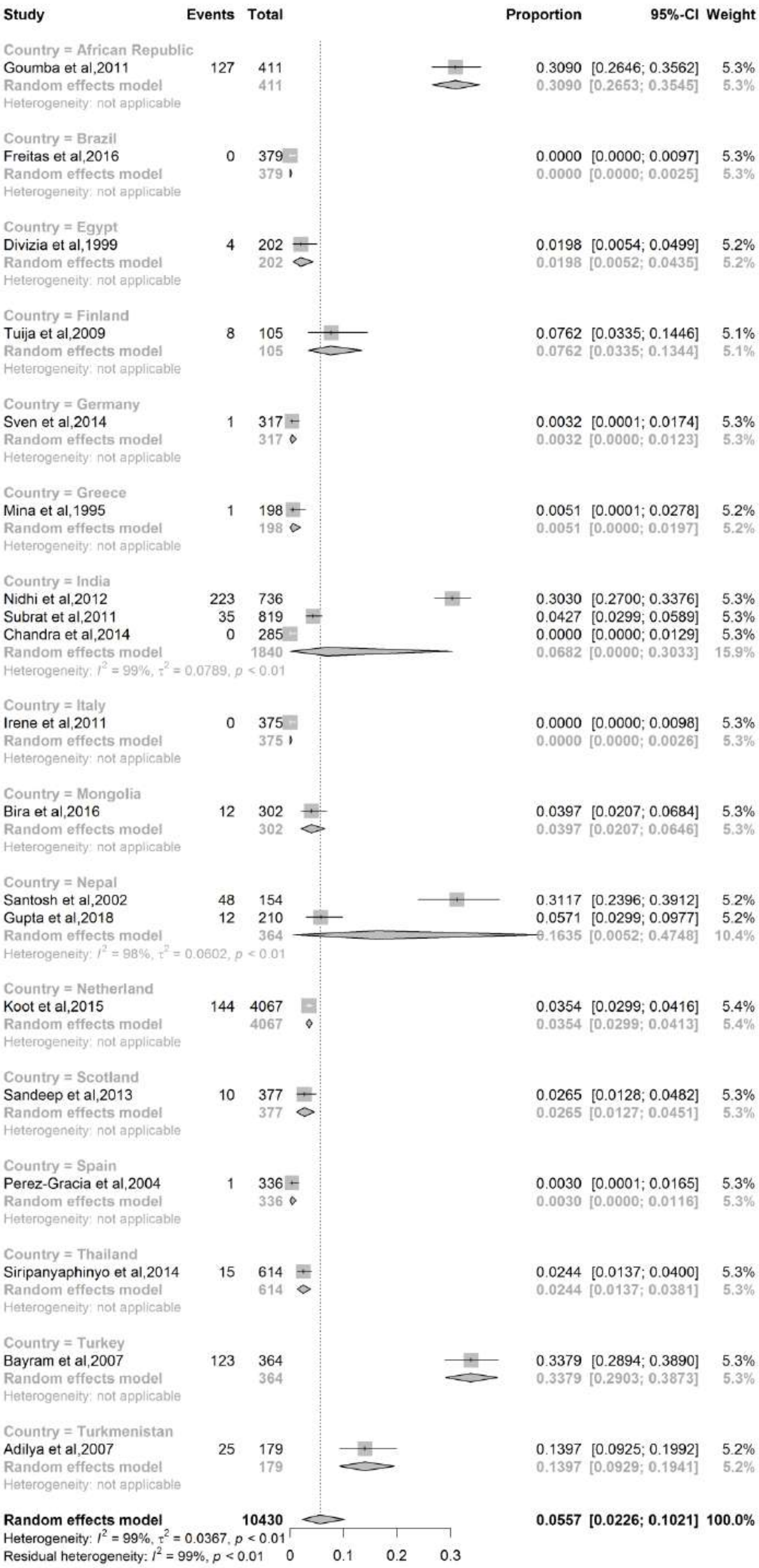

**Figure S20. Forest plot of estimated pooled seroprevalence of anti-HEV IgG among HIV population based on different countries.**

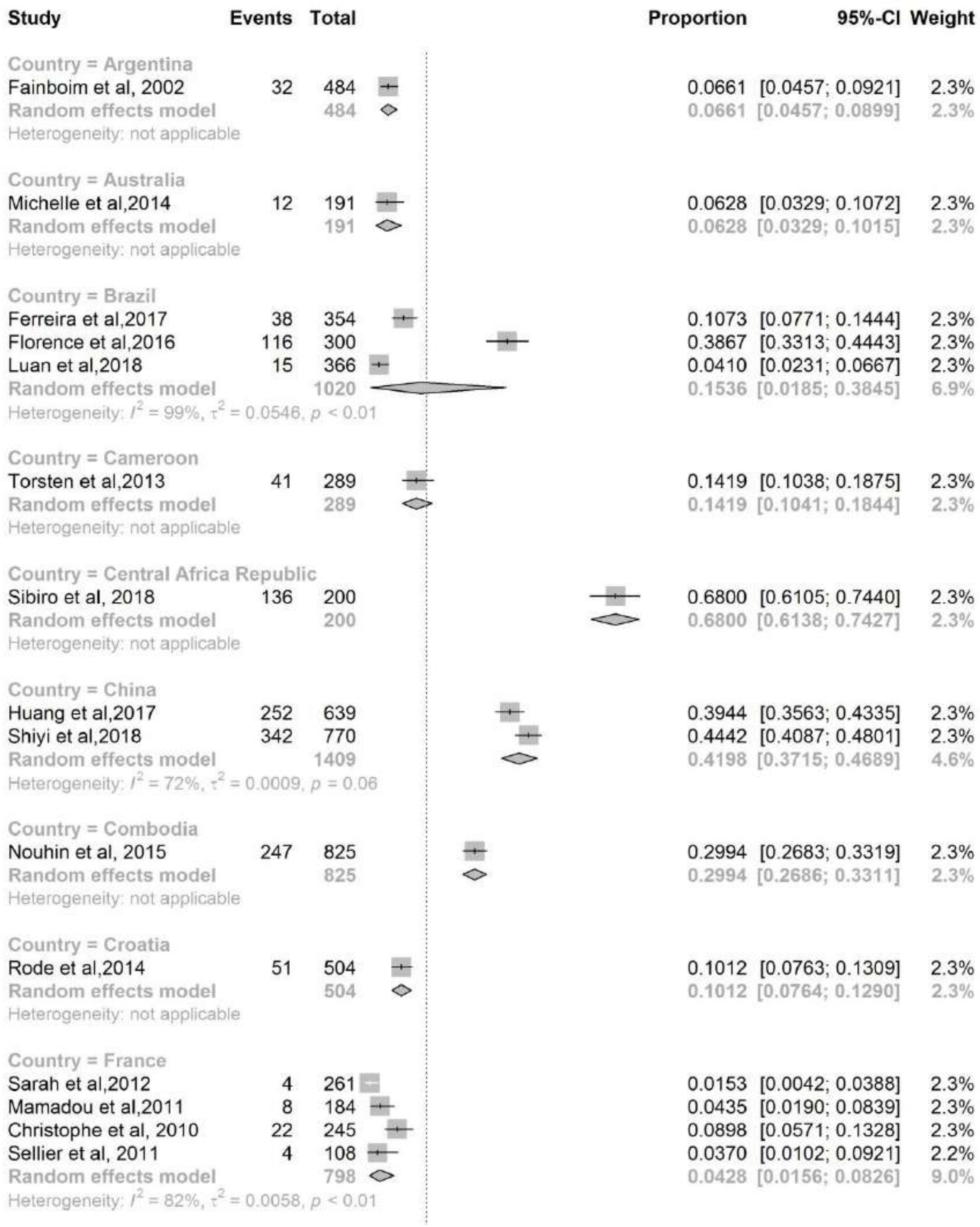

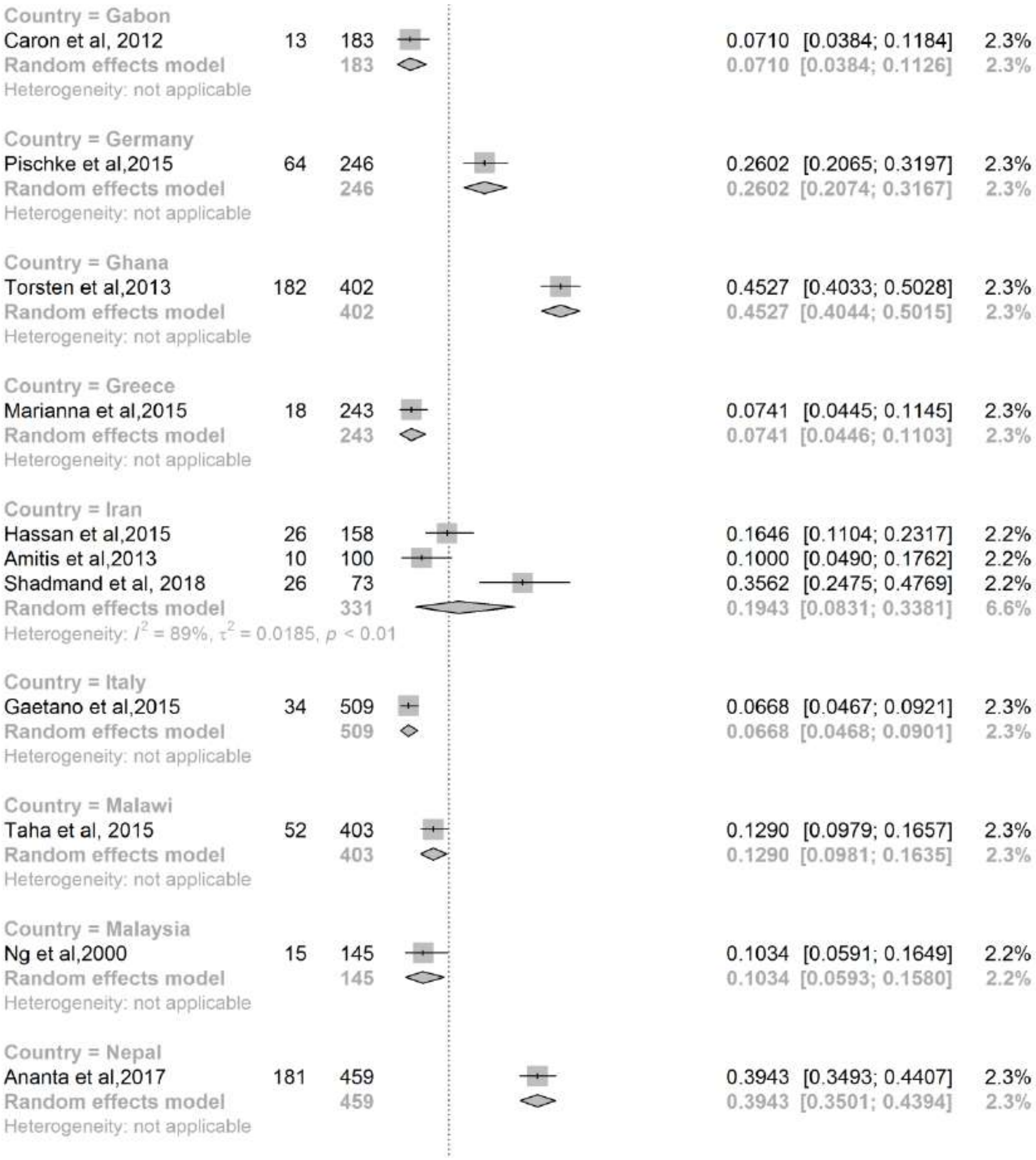

Country = Gabon
Caron et al, 2012 13 183 0.0710 [0.0384; 0.1184] 2.3%
Random effects model 183 0.0710 [0.0384; 0.1126] 2.3%
Heterogeneity: not applicable
Country = Germany
Pischke et al,2015 64 246 0.2602 [0.2065; 0.3197] 2.3%
Random effects model 246 0.2602 [0.2074; 0.3167] 2.3%
Heterogeneity: not applicable
Country = Ghana
Torsten et al,2013 182 402 0.4527 [0.4033; 0.5028] 2.3%
Random effects model 402 0.4527 [0.4044; 0.5015] 2.3%
Heterogeneity: not applicable
Country = Greece
Marianna et al,2015 18 243 0.0741 [0.0445; 0.1145] 2.3%
Random effects model 243 0.0741 [0.0446; 0.1103] 2.3%
Heterogeneity: not applicable
Country = Iran
Hassan et al,2015 26 158 0.1646 [0.1104; 0.2317] 2.2%
Amitis et al,2013 10 100 0.1000 [0.0490; 0.1762] 2.2%
Shadmand et al, 2018 26 73 0.3562 [0.2475; 0.4769] 2.2%
Random effects model 331 0.1943 [0.0831; 0.3381] 6.6%
Heterogeneity: $I^2$ = 89%, $\tau^2$ = 0.0185, $p$ < 0.01
Country = Italy
Gaetano et al,2015 34 509 0.0668 [0.0467; 0.0921] 2.3%
Random effects model 509 0.0668 [0.0468; 0.0901] 2.3%
Heterogeneity: not applicable
Country = Malawi
Taha et al, 2015 52 403 0.1290 [0.0979; 0.1657] 2.3%
Random effects model 403 0.1290 [0.0981; 0.1635] 2.3%
Heterogeneity: not applicable
Country = Malaysia
Ng et al,2000 15 145 0.1034 [0.0591; 0.1649] 2.2%
Random effects model 145 0.1034 [0.0593; 0.1580] 2.2%
Heterogeneity: not applicable
Country = Nepal
Ananta et al,2017 181 459 0.3943 [0.3493; 0.4407] 2.3%
Random effects model 459 0.3943 [0.3501; 0.4394] 2.3%
Heterogeneity: not applicable

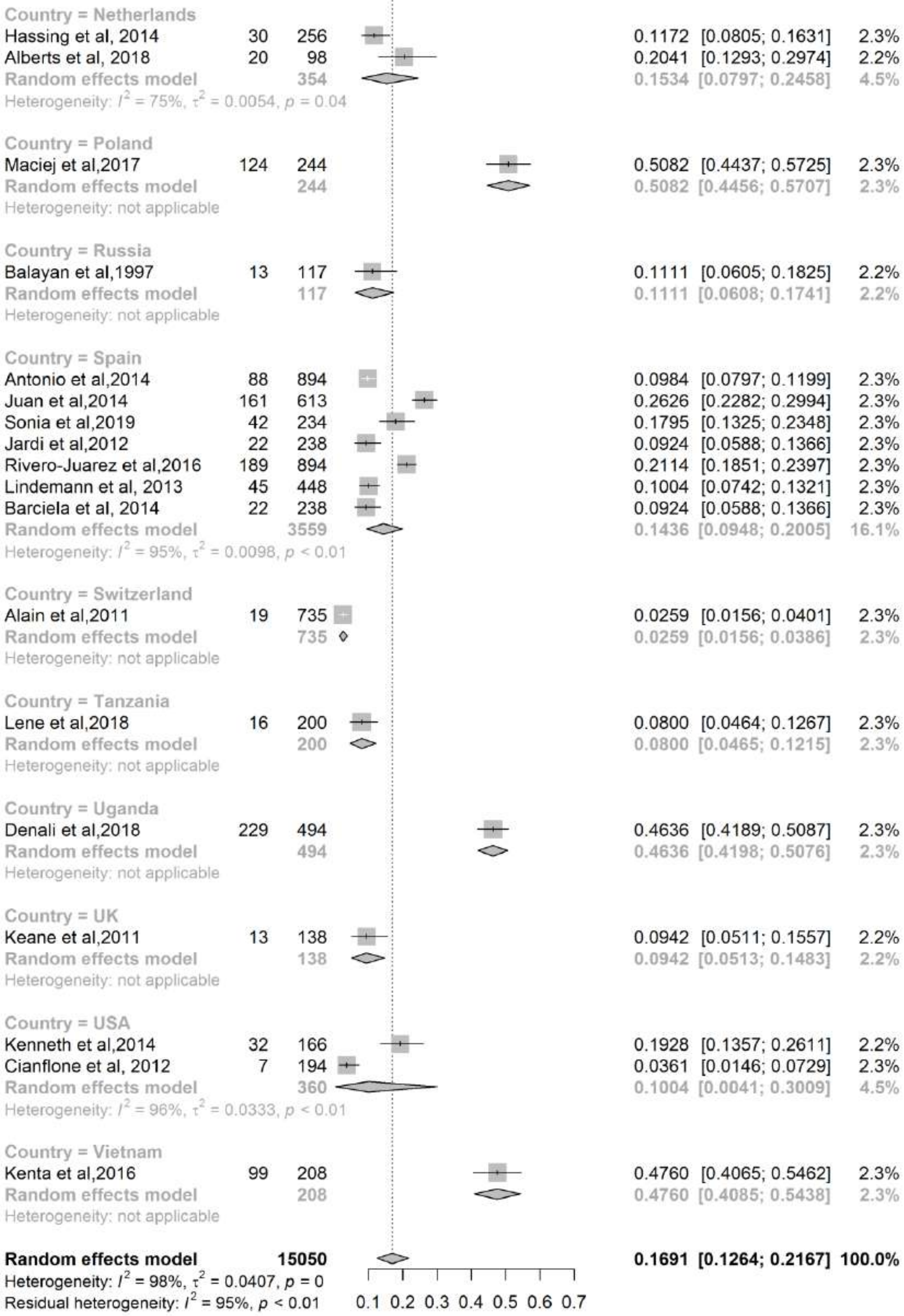

Country = Netherlands
Hassing et al, 2014 30 256 0.1172 [0.0805; 0.1631] 2.3%
Alberts et al, 2018 20 98 0.2041 [0.1293; 0.2974] 2.2%
Random effects model 354 0.1534 [0.0797; 0.2458] 4.5%
Heterogeneity: $I^2$ = 75%, $\tau^2$ = 0.0054, $p$ = 0.04
Country = Poland
Maciej et al,2017 124 244 0.5082 [0.4437; 0.5725] 2.3%
Random effects model 244 0.5082 [0.4456; 0.5707] 2.3%
Heterogeneity: not applicable
Country = Russia
Balayan et al,1997 13 117 0.1111 [0.0605; 0.1825] 2.2%
Random effects model 117 0.1111 [0.0608; 0.1741] 2.2%
Heterogeneity: not applicable
Country = Spain
Antonio et al,2014 88 894 0.0984 [0.0797; 0.1199] 2.3%
Juan et al,2014 161 613 0.2626 [0.2282; 0.2994] 2.3%
Sonia et al,2019 42 234 0.1795 [0.1325; 0.2348] 2.3%
Jardi et al,2012 22 238 0.0924 [0.0588; 0.1366] 2.3%
Rivero-Juarez et al,2016 189 894 0.2114 [0.1851; 0.2397] 2.3%
Lindemann et al, 2013 45 448 0.1004 [0.0742; 0.1321] 2.3%
Barciela et al, 2014 22 238 0.0924 [0.0588; 0.1366] 2.3%
Random effects model 3559 0.1436 [0.0948; 0.2005] 16.1%
Heterogeneity: $I^2$ = 95%, $\tau^2$ = 0.0098, $p$ < 0.01
Country = Switzerland
Alain et al,2011 19 735 0.0259 [0.0156; 0.0401] 2.3%
Random effects model 735 0.0259 [0.0156; 0.0386] 2.3%
Heterogeneity: not applicable
Country = Tanzania
Lene et al,2018 16 200 0.0800 [0.0464; 0.1267] 2.3%
Random effects model 200 0.0800 [0.0465; 0.1215] 2.3%
Heterogeneity: not applicable
Country = Uganda
Denali et al,2018 229 494 0.4636 [0.4189; 0.5087] 2.3%
Random effects model 494 0.4636 [0.4198; 0.5076] 2.3%
Heterogeneity: not applicable
Country = UK
Keane et al,2011 13 138 0.0942 [0.0511; 0.1557] 2.2%
Random effects model 138 0.0942 [0.0513; 0.1483] 2.2%
Heterogeneity: not applicable
Country = USA
Kenneth et al,2014 32 166 0.1928 [0.1357; 0.2611] 2.2%
Cianflone et al, 2012 7 194 0.0361 [0.0146; 0.0729] 2.3%
Random effects model 360 0.1004 [0.0041; 0.3009] 4.5%
Heterogeneity: $I^2$ = 96%, $\tau^2$ = 0.0333, $p$ < 0.01
Country = Vietnam
Kenta et al,2016 99 208 0.4760 [0.4065; 0.5462] 2.3%
Random effects model 208 0.4760 [0.4085; 0.5438] 2.3%
Heterogeneity: not applicable
Random effects model 15050 0.1691 [0.1264; 0.2167] 100.0%
Heterogeneity: $I^2$ = 98%, $\tau^2$ = 0.0407, $p$ = 0
Residual heterogeneity: $I^2$ = 95%, $p$ < 0.01
0.1 0.2 0.3 0.4 0.5 0.6 0.7

**Figure S21. Forest plot of estimated pooled seroprevalence of anti-HEV IgM among HIV population based on different countries.**

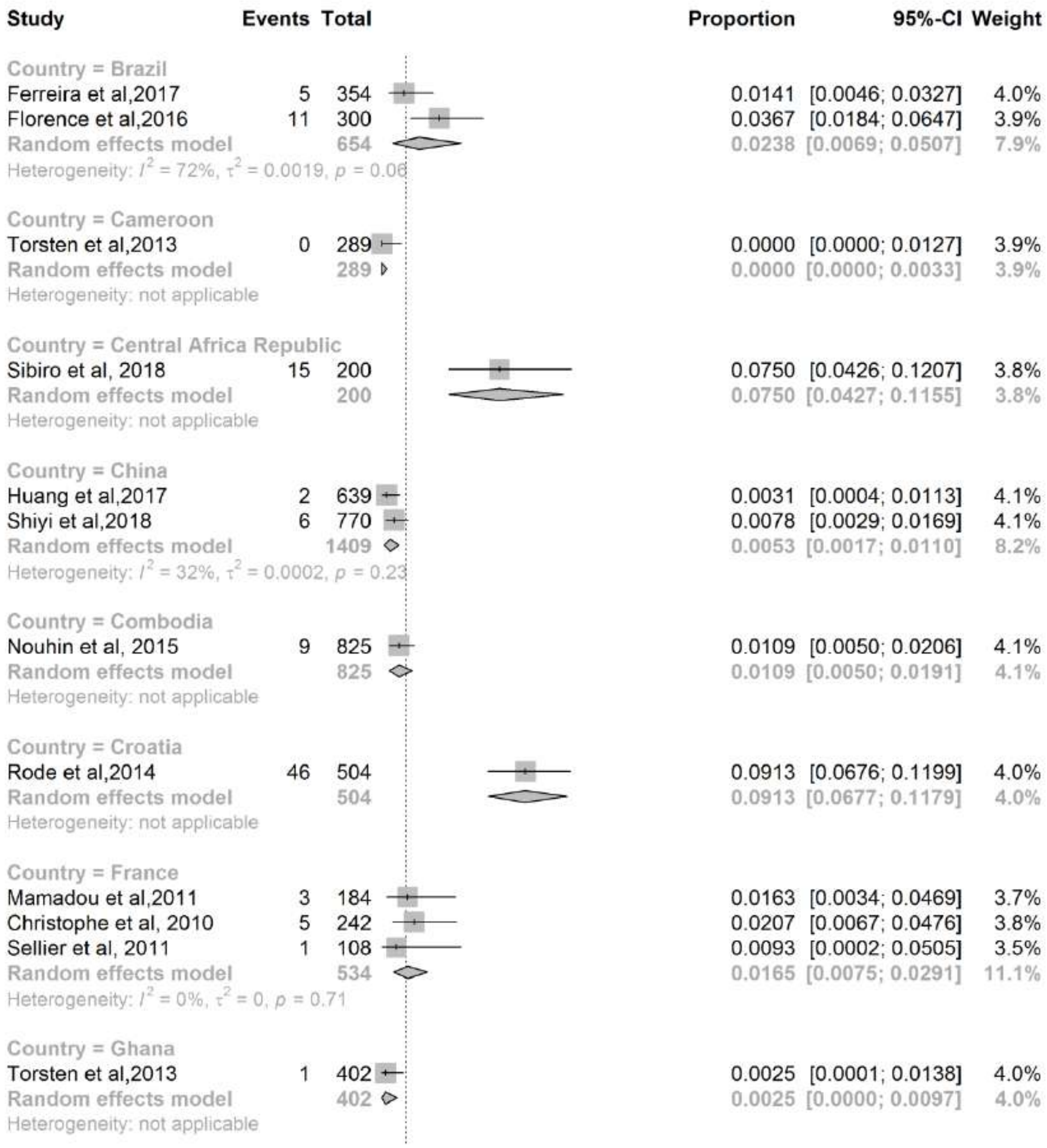

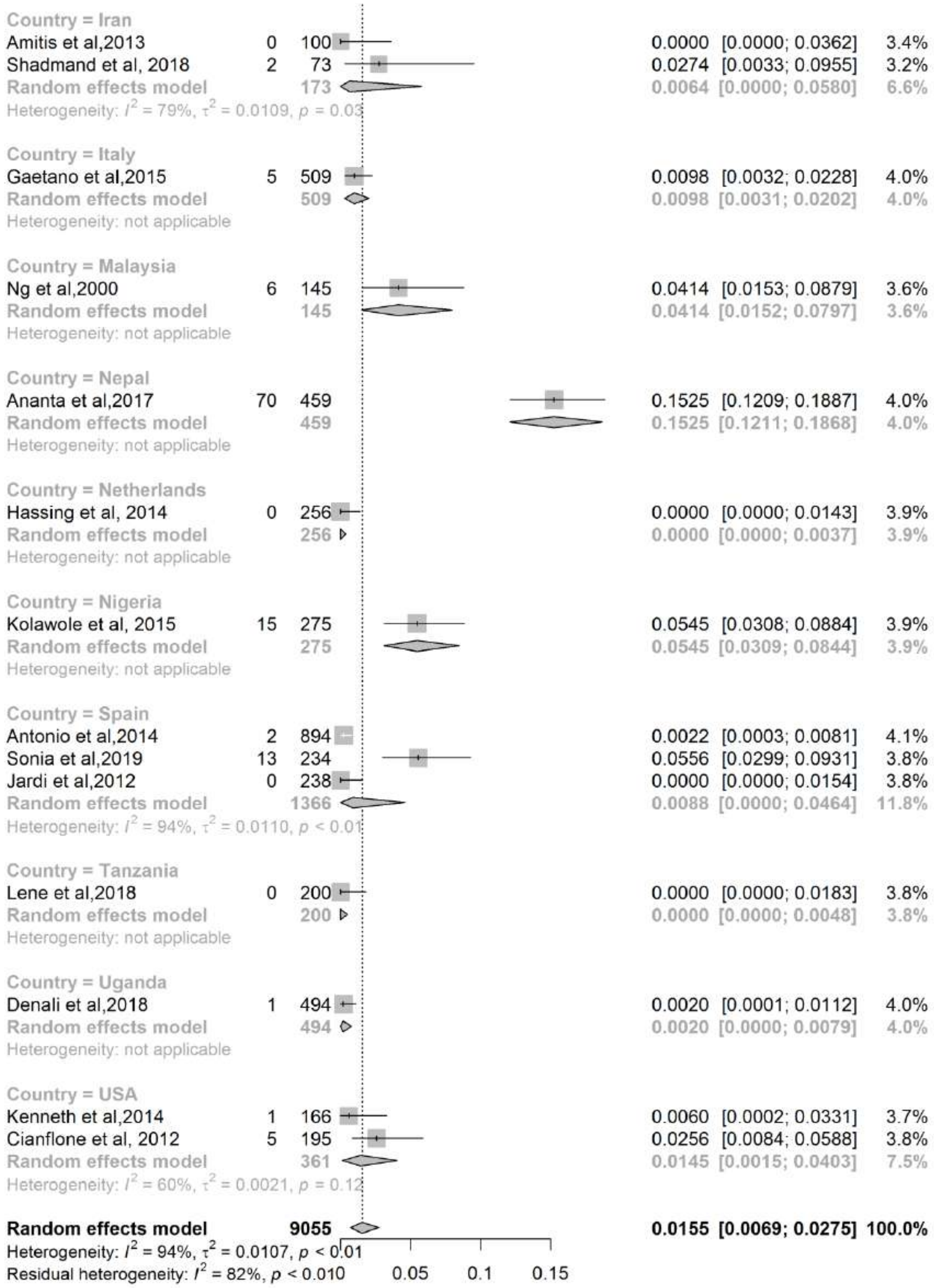
Country = Iran
Amitis et al,2013 0 100 0.0000 [0.0000; 0.0362] 3.4%
Shadmand et al, 2018 2 73 0.0274 [0.0033; 0.0955] 3.2%
Random effects model 173 0.0064 [0.0000; 0.0580] 6.6%
Heterogeneity: $I^2$ = 79%, $\tau^2$ = 0.0109, $p$ = 0.03
Country = Italy
Gaetano et al,2015 5 509 0.0098 [0.0032; 0.0228] 4.0%
Random effects model 509 0.0098 [0.0031; 0.0202] 4.0%
Heterogeneity: not applicable
Country = Malaysia
Ng et al,2000 6 145 0.0414 [0.0153; 0.0879] 3.6%
Random effects model 145 0.0414 [0.0152; 0.0797] 3.6%
Heterogeneity: not applicable
Country = Nepal
Ananta et al,2017 70 459 0.1525 [0.1209; 0.1887] 4.0%
Random effects model 459 0.1525 [0.1211; 0.1868] 4.0%
Heterogeneity: not applicable
Country = Netherlands
Hassing et al, 2014 0 256 0.0000 [0.0000; 0.0143] 3.9%
Random effects model 256 0.0000 [0.0000; 0.0037] 3.9%
Heterogeneity: not applicable
Country = Nigeria
Kolawole et al, 2015 15 275 0.0545 [0.0308; 0.0884] 3.9%
Random effects model 275 0.0545 [0.0309; 0.0844] 3.9%
Heterogeneity: not applicable
Country = Spain
Antonio et al,2014 2 894 0.0022 [0.0003; 0.0081] 4.1%
Sonia et al,2019 13 234 0.0556 [0.0299; 0.0931] 3.8%
Jardi et al,2012 0 238 0.0000 [0.0000; 0.0154] 3.8%
Random effects model 1366 0.0088 [0.0000; 0.0464] 11.8%
Heterogeneity: $I^2$ = 94%, $\tau^2$ = 0.0110, $p$ < 0.01
Country = Tanzania
Lene et al,2018 0 200 0.0000 [0.0000; 0.0183] 3.8%
Random effects model 200 0.0000 [0.0000; 0.0048] 3.8%
Heterogeneity: not applicable
Country = Uganda
Denali et al,2018 1 494 0.0020 [0.0001; 0.0112] 4.0%
Random effects model 494 0.0020 [0.0000; 0.0079] 4.0%
Heterogeneity: not applicable
Country = USA
Kenneth et al,2014 1 166 0.0060 [0.0002; 0.0331] 3.7%
Cianflone et al, 2012 5 195 0.0256 [0.0084; 0.0588] 3.8%
Random effects model 361 0.0145 [0.0015; 0.0403] 7.5%
Heterogeneity: $I^2$ = 60%, $\tau^2$ = 0.0021, $p$ = 0.12
Random effects model 9055 0.0155 [0.0069; 0.0275] 100.0%
Heterogeneity: $I^2$ = 94%, $\tau^2$ = 0.0107, $p$ < 0.01
Residual heterogeneity: $I^2$ = 82%, $p$ < 0.01
0 0.05 0.1 0.15

**Figure S22. Forest plot of estimated pooled HEV RNA positive rate among HIV population based on different countries.**

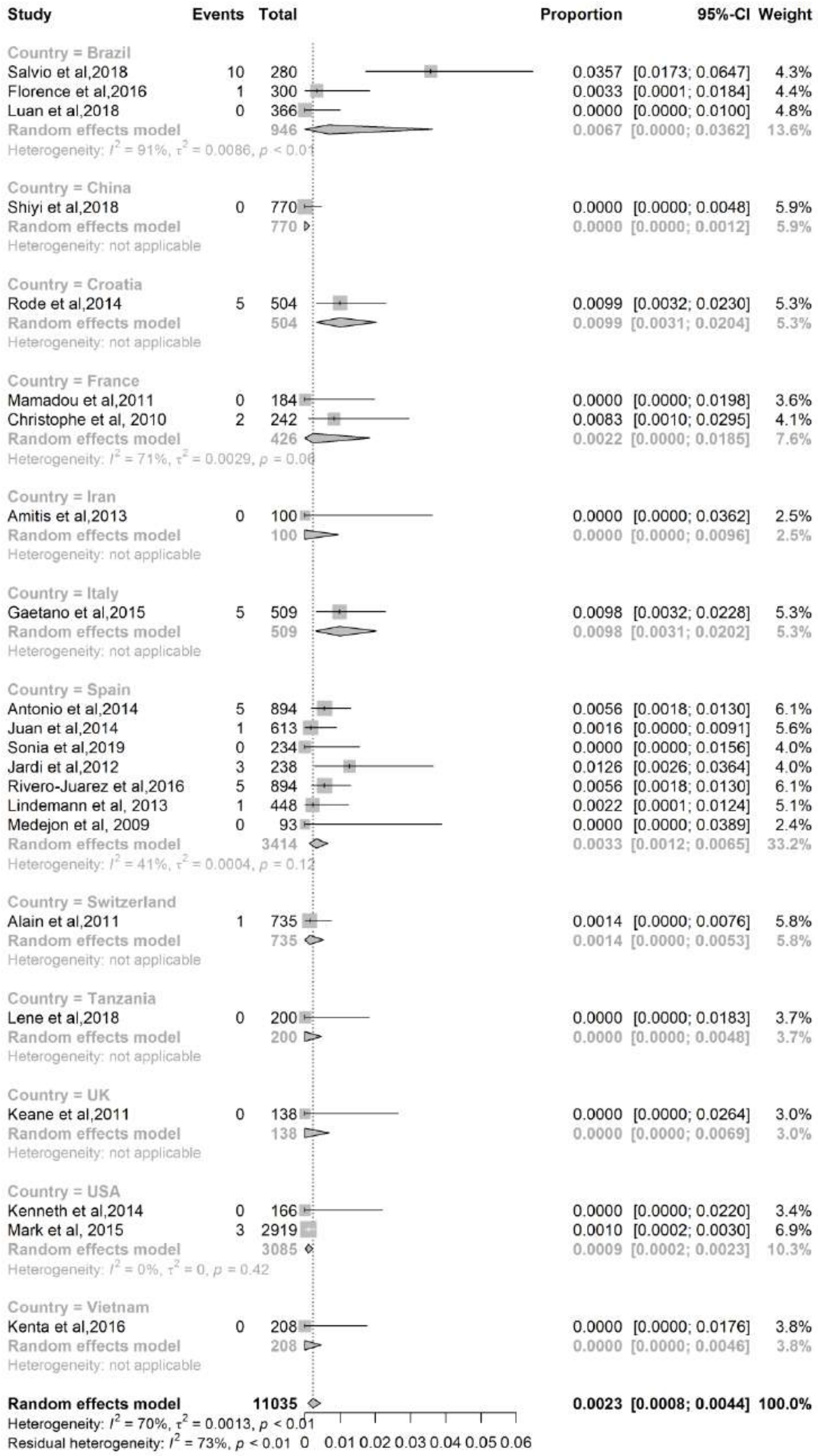

**Figure S23. Forest plot of estimated pooled seroprevalence of anti-HEV IgG among transplant population based on different countries.**

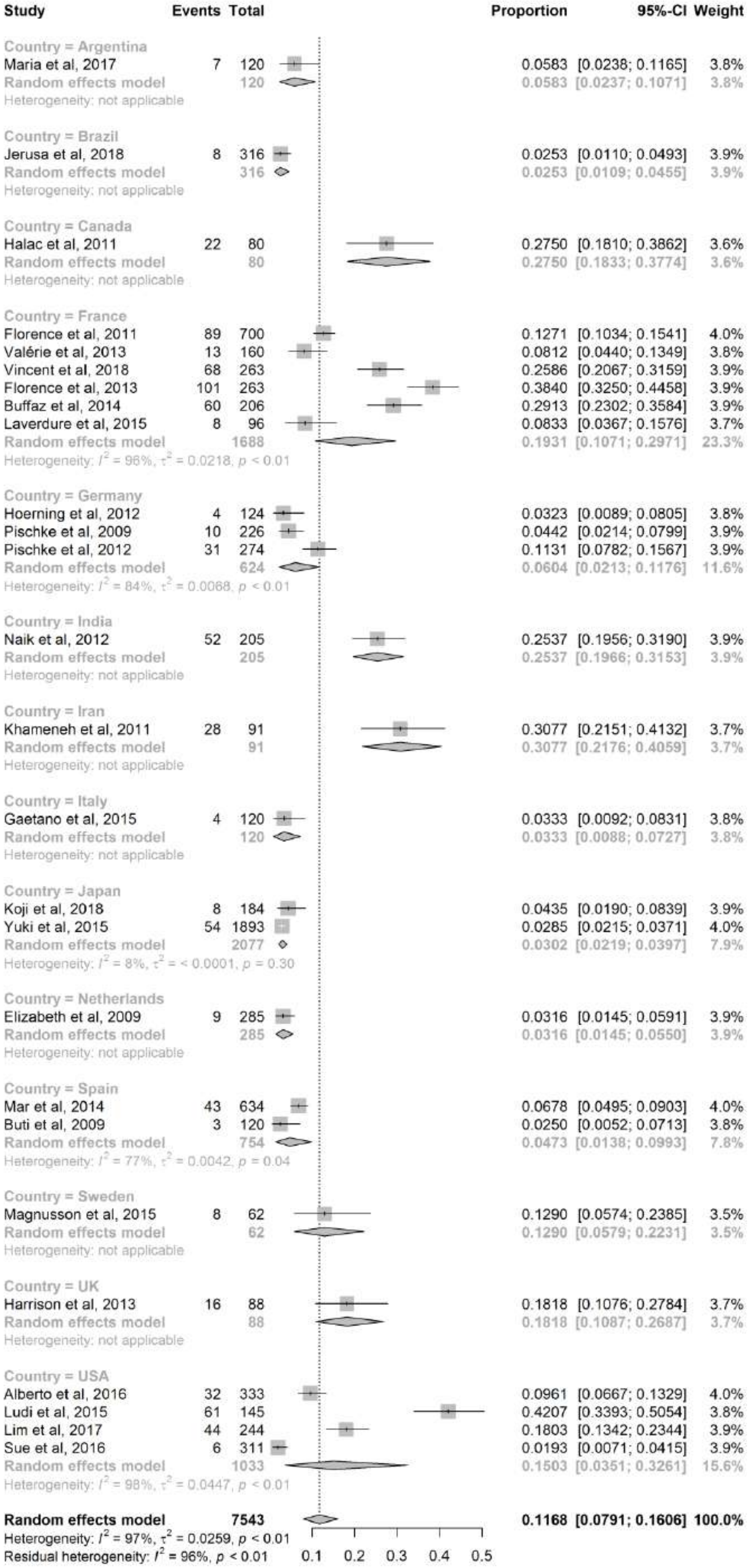

**Figure S24. Forest plot of estimated pooled seroprevalence of anti-HEV IgM among transplant population based on different countries.**

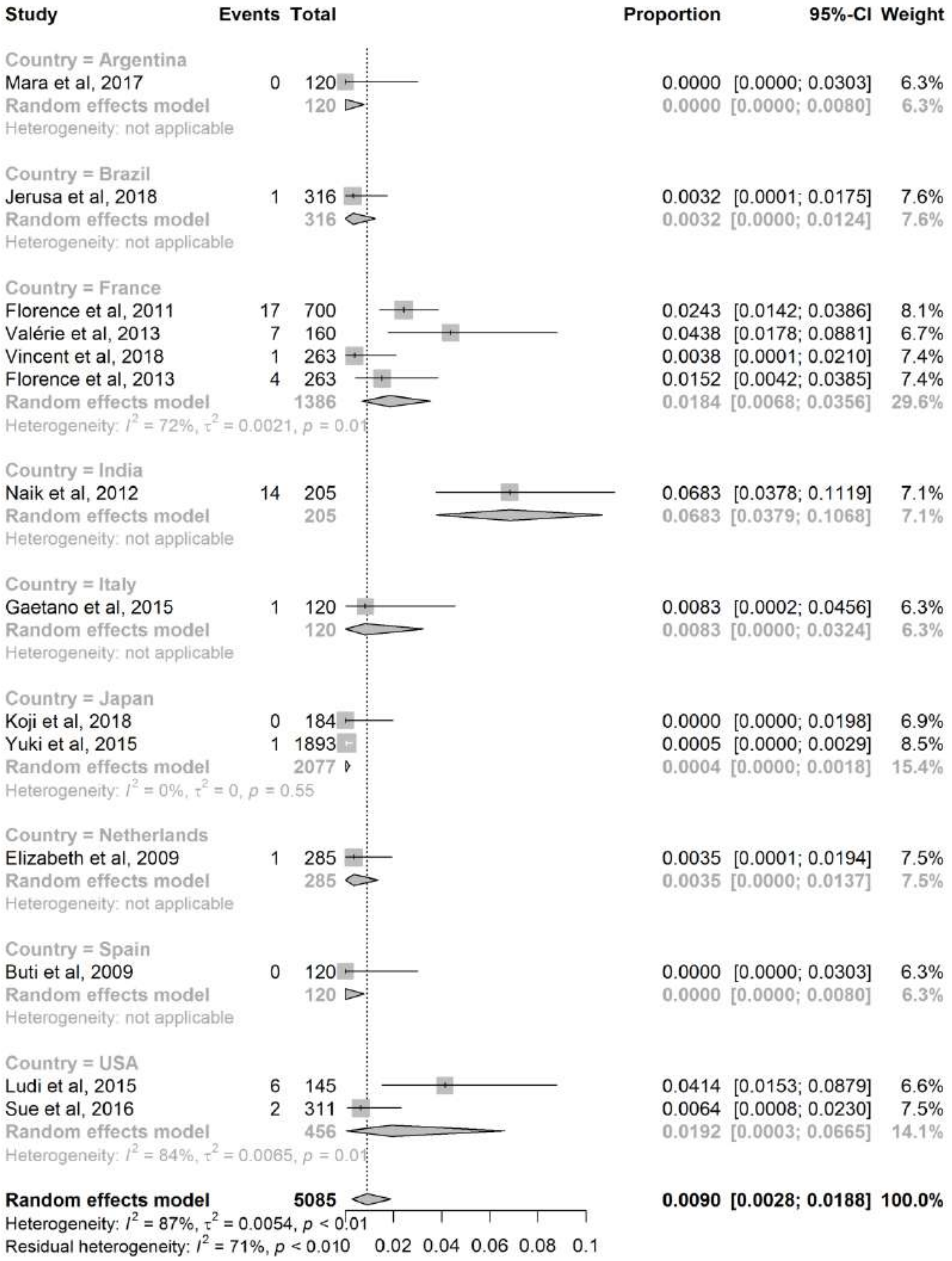

**Figure S25. Forest plot of estimated pooled HEV RNA positive rate among transplant population based on different countries.**

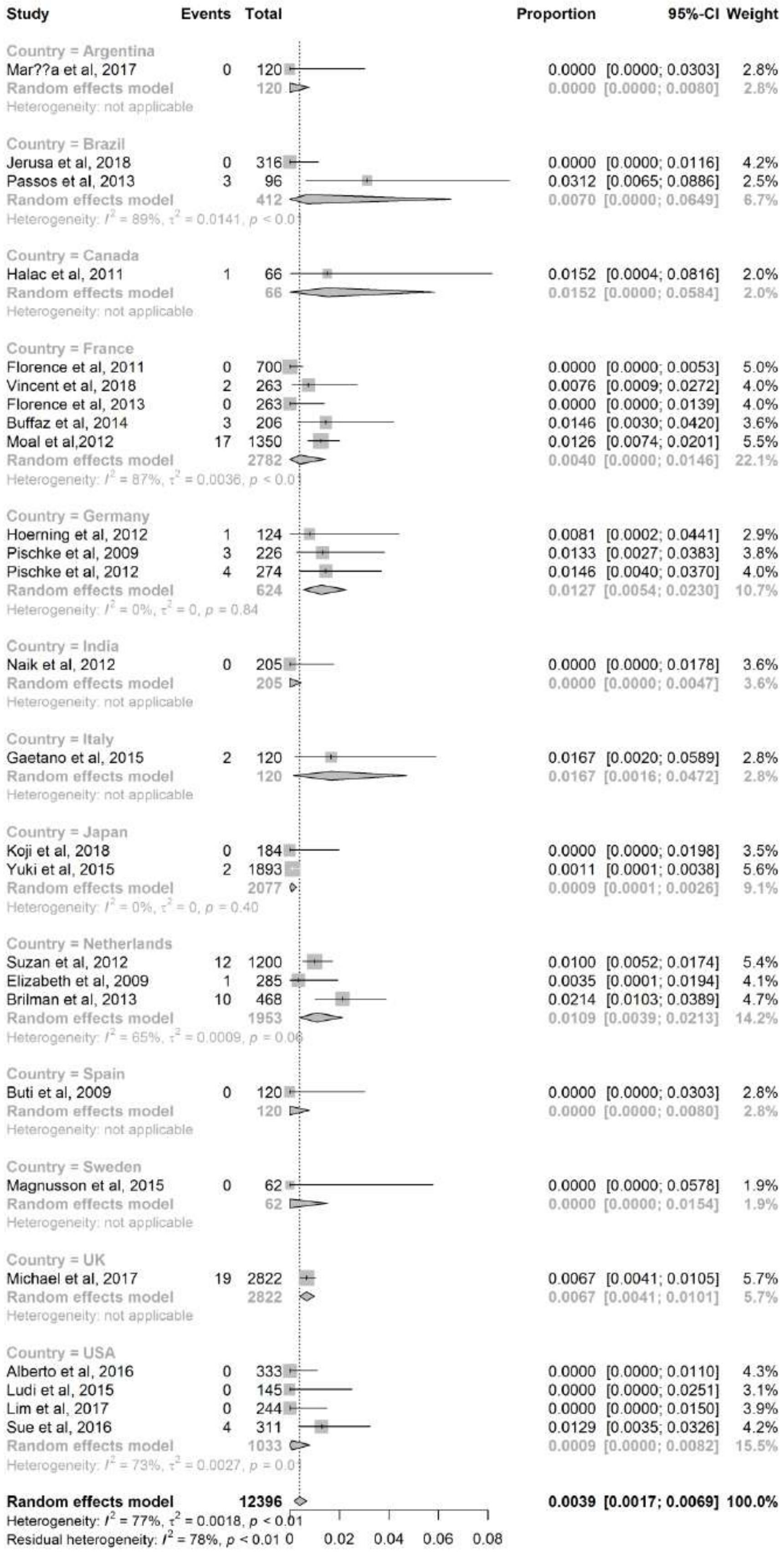

**Figure S26. Forest plot of estimated pooled seroprevalence of anti-HEV IgG among hemodialysis population based on different countries.**

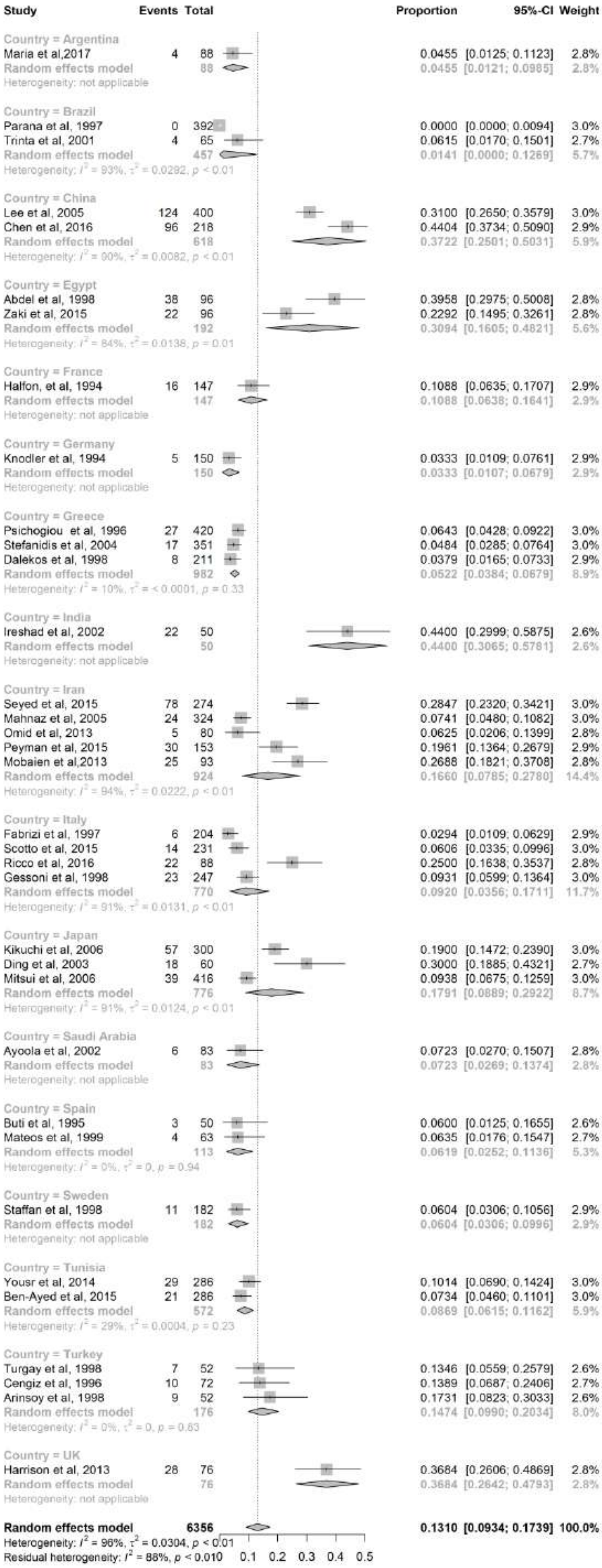

**Figure S27. Forest plot of estimated pooled seroprevalence of anti-HEV IgM among hemodialysis population based on different countries.**

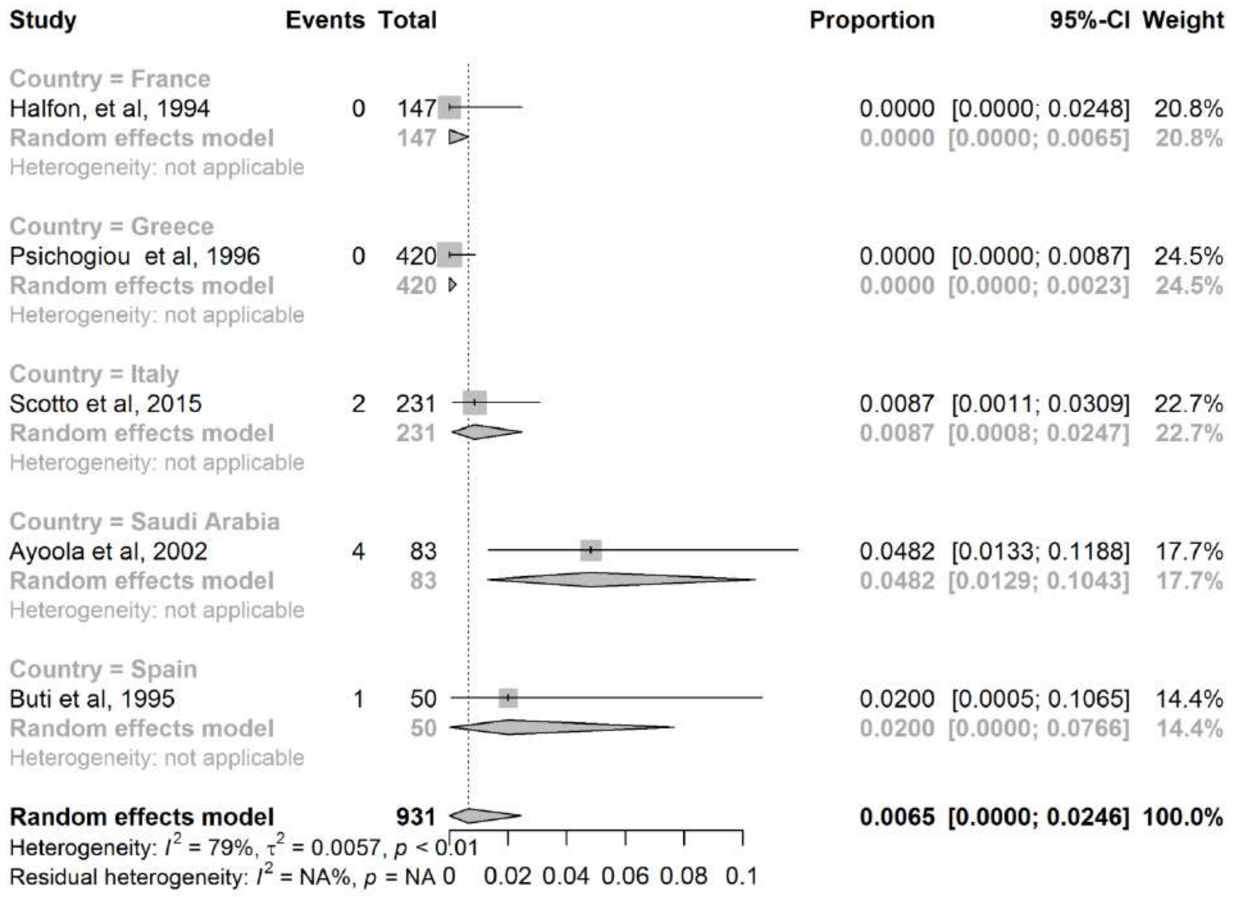

**Figure S28. Pooled Odd Ratios of anti-HEV IgG seroprevalence to investigate the risk factors for HEV infection among general population.**

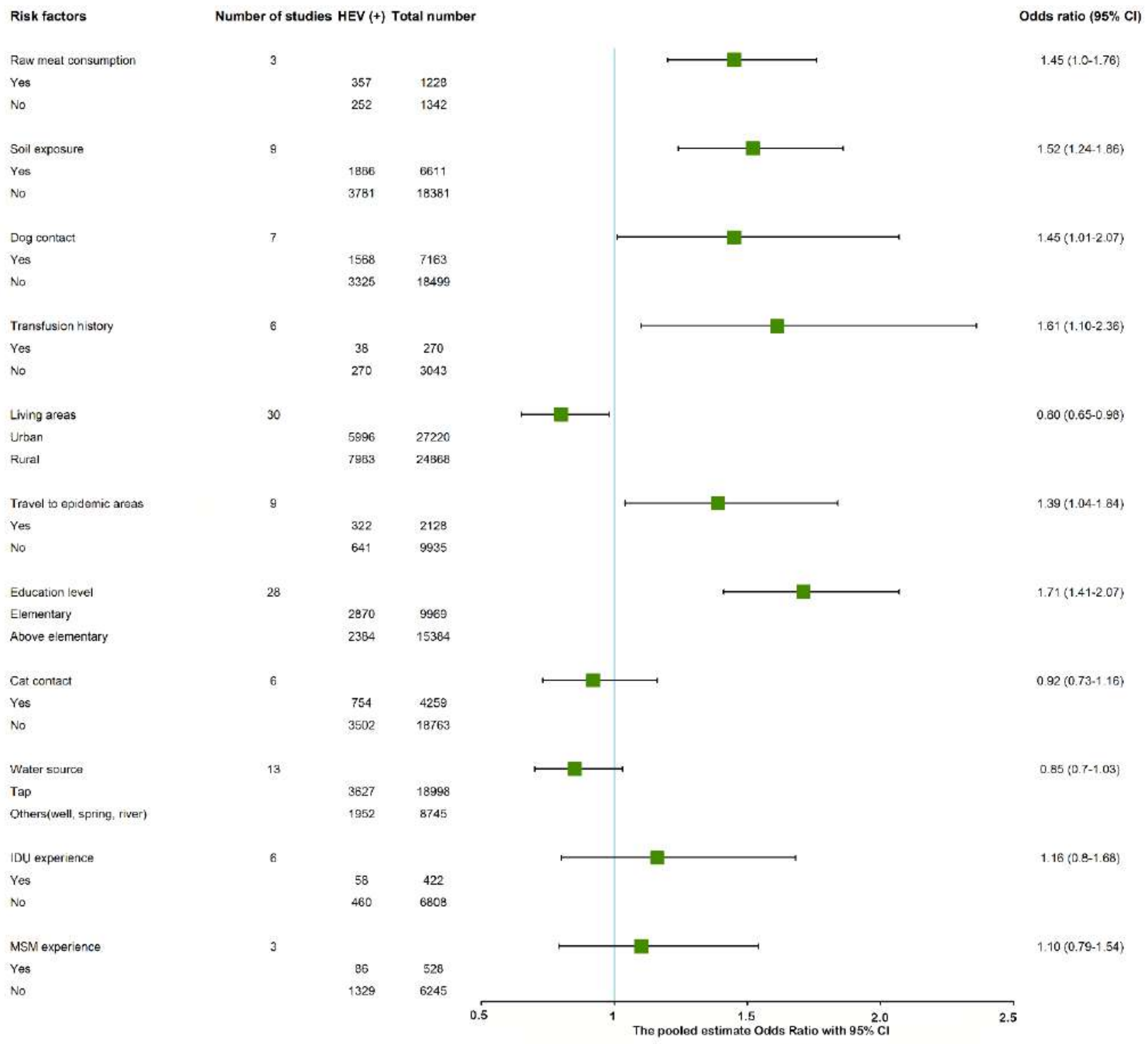

**Figure S29. Odds ratio analysis of anti-HEV seroprevalence for people contacting dogs or not in general population.**

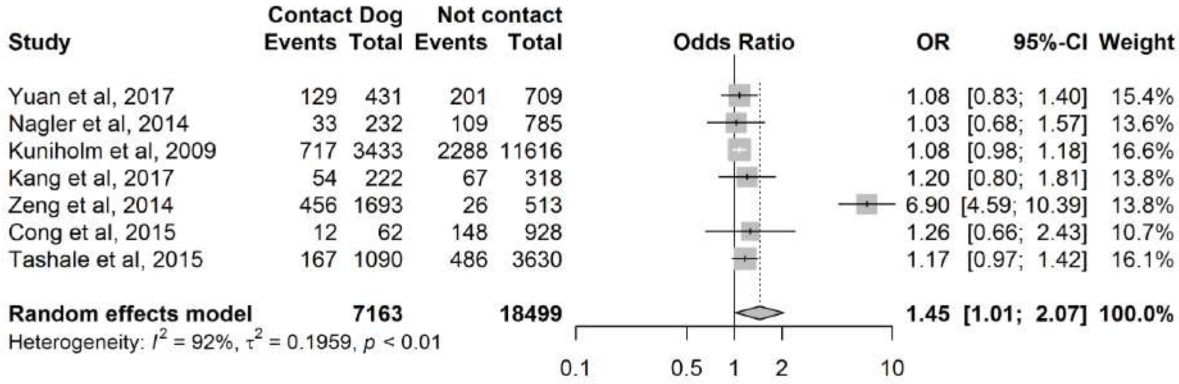


**Figure S30. Odds ratio analysis of anti-HEV IgG seroprevalence for people contacting cats or not in general population.**

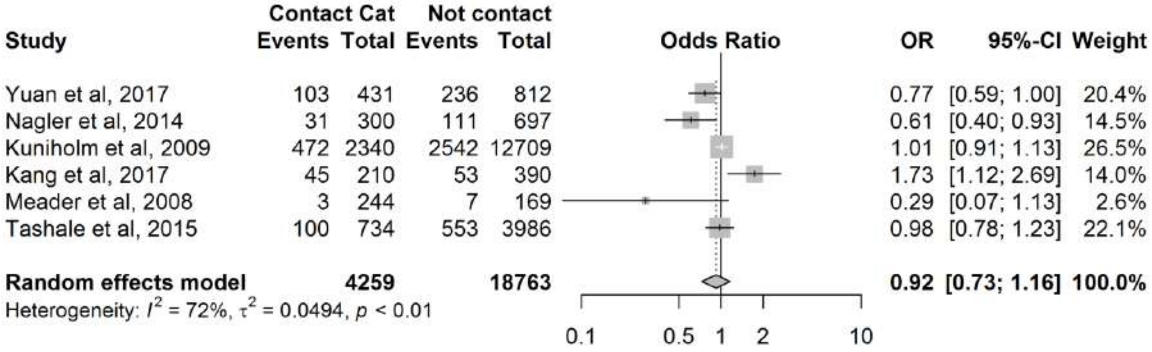


**Figure S31. Odds ratio analysis of anti-HEV IgG seroprevalence for people with or without blood transfusion in general population.**

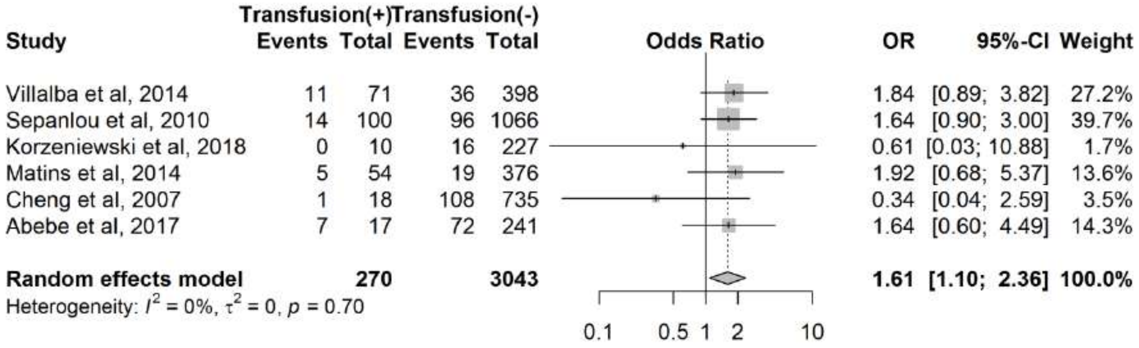

**Figure S32. Odds ratio analysis of anti-HEV IgG seroprevalence for people with or without consumption of raw meat in general population.**

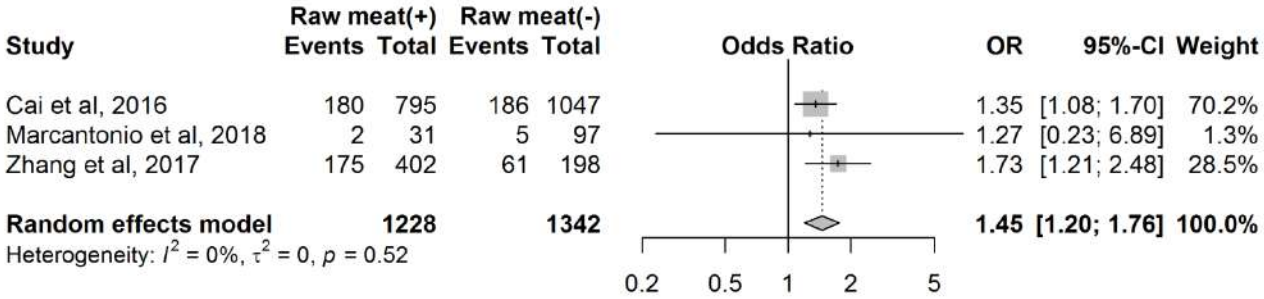


**Figure S33. Odds ratio analysis of anti-HEV IgG seroprevalence for people frequently exposed to soil or unexposed to soil in general population.**

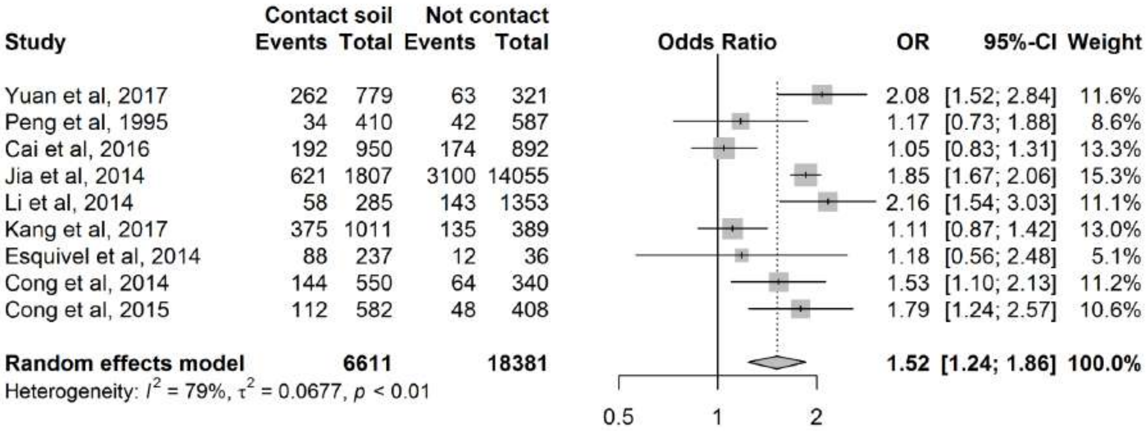


**Figure S34. Odds ratio analysis of anti-HEV IgG seroprevalence for people with or without travelling to endemic areas in general population.**

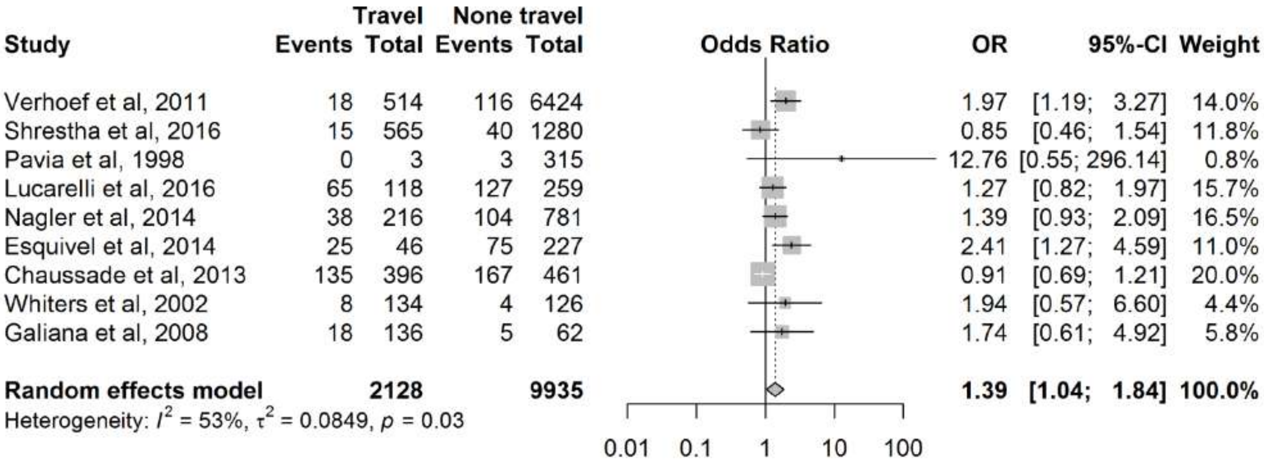

**Figure S35. Odds ratio analysis of anti-HEV IgG seroprevalence for urban residents or rural residents in general population.**

| Study | Urban Events | Urban Total | Rural Events | Rural Total | OR | 95%-CI | Weight |
|---|---|---|---|---|---|---|---|
| Yuan et al, 2017 | 74 | 372 | 256 | 828 | 0.55 | [0.41; 0.74] | 5.0% |
| Hassan et al, 2013 | 71 | 475 | 5 | 55 | 1.76 | [0.68; 4.56] | 2.5% |
| Ahn et al, 2005 | 1 | 280 | 14 | 288 | 0.07 | [0.01; 0.54] | 0.9% |
| Abe et al, 2006 | 1 | 280 | 14 | 288 | 0.07 | [0.01; 0.54] | 0.9% |
| Yoon et al, 2014 | 90 | 1937 | 54 | 513 | 0.41 | [0.29; 0.59] | 4.8% |
| Yun et al, 2011 | 124 | 201 | 151 | 234 | 0.89 | [0.60; 1.31] | 4.7% |
| Vivek et al, 2010 | 226 | 1135 | 104 | 1144 | 2.49 | [1.94; 3.19] | 5.2% |
| Bortoliero et al, 2006 | 21 | 915 | 2 | 58 | 0.66 | [0.15; 2.88] | 1.4% |
| Cai et al, 2016 | 155 | 1080 | 211 | 762 | 0.44 | [0.35; 0.55] | 5.2% |
| Ataei et al, 2007 | 26 | 634 | 5 | 182 | 1.51 | [0.57; 4.00] | 2.5% |
| Hickey et al, 2016 | 13 | 100 | 3 | 98 | 4.73 | [1.30; 17.17] | 1.7% |
| Sepanlou et al, 2010 | 95 | 1172 | 32 | 251 | 0.60 | [0.39; 0.92] | 4.5% |
| Pujol et al, 1994 | 3 | 184 | 8 | 204 | 0.41 | [0.11; 1.55] | 1.6% |
| Lucarelli et al, 2016 | 69 | 149 | 84 | 164 | 0.82 | [0.53; 1.28] | 4.4% |
| Liang et al, 2014 | 74 | 193 | 55 | 114 | 0.67 | [0.42; 1.07] | 4.3% |
| Marcantonio et al, 2018 | 5 | 64 | 2 | 64 | 2.63 | [0.49; 14.07] | 1.2% |
| Jia et al, 2014 | 1781 | 7842 | 1918 | 8010 | 0.93 | [0.87; 1.00] | 5.6% |
| Kuniholm et al, 2009 | 2453 | 6636 | 4254 | 8415 | 0.57 | [0.54; 0.61] | 5.6% |
| Kang et al, 2017 | 94 | 470 | 316 | 1030 | 0.56 | [0.43; 0.73] | 5.1% |
| Zhang et al, 2017 | 134 | 355 | 102 | 245 | 0.85 | [0.61; 1.19] | 4.9% |
| Cong et al, 2014 | 154 | 624 | 54 | 266 | 1.29 | [0.91; 1.83] | 4.8% |
| Serkan et al, 2005 | 18 | 257 | 9 | 129 | 1.00 | [0.44; 2.30] | 2.9% |
| Zakieh et al, 2013 | 5 | 73 | 0 | 63 | 10.20 | [0.55; 188.15] | 0.5% |
| Niguse et al, 2018 | 176 | 356 | 183 | 490 | 1.64 | [1.24; 2.16] | 5.1% |
| Caron et al, 2008 | 28 | 207 | 11 | 173 | 2.30 | [1.11; 4.78] | 3.3% |
| Cevrioglu et al, 2004 | 14 | 153 | 17 | 92 | 0.44 | [0.21; 0.95] | 3.2% |
| Cong et al, 2015 | 82 | 549 | 78 | 441 | 0.82 | [0.58; 1.15] | 4.9% |
| Adjei et al, 2009 | 8 | 21 | 29 | 47 | 0.38 | [0.13; 1.10] | 2.2% |
| Alizadeh et al, 2016 | 1 | 246 | 9 | 75 | 0.03 | [0.00; 0.24] | 0.8% |
| Trinta et al,2001 | 0 | 260 | 3 | 145 | 0.08 | [0.00; 1.52] | 0.4% |
| **Random effects model** | | **27220** | | **24868** | **0.80** | **[0.65; 0.98]** | **100.0%** |

Heterogeneity: $I^2 = 91\%$, $\tau^2 = 0.1933$, $p < 0.01$

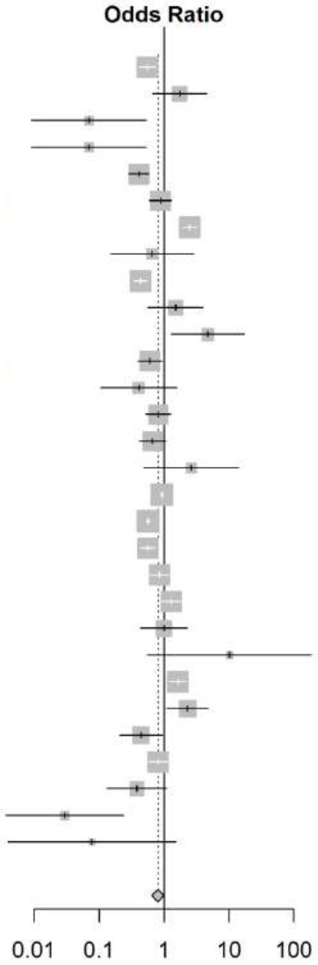

**Figure S36. Odds ratio analysis of anti-HEV IgG seroprevalence for people receiving water source of tap or none tap in general population.**

| Study | Tap water Events | Tap water Total | None tap Events | None tap Total | OR | 95%-CI | Weight |
|---|---|---|---|---|---|---|---|
| Yuan et al, 2017 | 226 | 868 | 105 | 332 | 0.76 | [0.58; 1.00] | 11.9% |
| Olcay et al, 2003 | 54 | 869 | 3 | 41 | 0.84 | [0.25; 2.81] | 2.1% |
| Bortoliero et al, 2006 | 23 | 959 | 0 | 14 | 0.73 | [0.04; 12.56] | 0.4% |
| Cai et al, 2016 | 213 | 1271 | 153 | 571 | 0.55 | [0.43; 0.70] | 12.8% |
| Madden et al, 2016 | 11 | 44 | 313 | 1117 | 0.86 | [0.43; 1.72] | 5.1% |
| Kuniholm et al, 2009 | 2391 | 11782 | 326 | 1731 | 1.10 | [0.96; 1.25] | 14.9% |
| Esquivel et al, 2014 | 61 | 190 | 39 | 83 | 0.53 | [0.31; 0.90] | 7.1% |
| Chaussade et al, 2013 | 267 | 772 | 35 | 87 | 0.79 | [0.50; 1.24] | 8.3% |
| Cong et al, 2014 | 144 | 624 | 64 | 266 | 0.95 | [0.68; 1.33] | 10.6% |
| Serkan et al, 2005 | 25 | 305 | 5 | 81 | 1.36 | [0.50; 3.66] | 3.0% |
| Niguse et al, 2018 | 7 | 10 | 352 | 484 | 0.88 | [0.22; 3.43] | 1.7% |
| Cong et al, 2015 | 118 | 740 | 42 | 250 | 0.94 | [0.64; 1.38] | 9.6% |
| Tashale et al, 2015 | 87 | 564 | 515 | 3688 | 1.12 | [0.88; 1.44] | 12.6% |
| **Random effects model** | | **18998** | | **8745** | **0.85** | **[0.70; 1.03]** | **100.0%** |

Heterogeneity: $I^2 = 66\%$, $\tau^2 = 0.0584$, $p < 0.01$

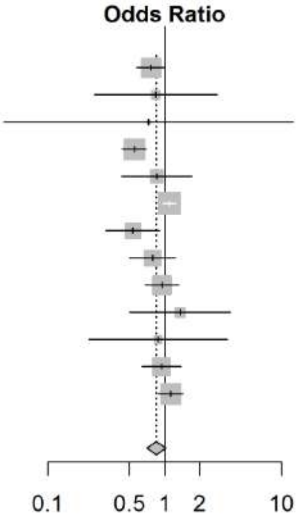

**Figure S37. Odds ratio analysis of anti-HEV IgG seroprevalence for people accepting education of elementary or above elementary in general population.**

| Study | <Elementary Events | <Elementary Total | >Elementary Events | >Elementary Total | OR | 95%-CI | Weight |
|---|---|---|---|---|---|---|---|
| Azarkar et al, 2017 | 58 | 149 | 93 | 417 | 2.22 | [1.49; 3.32] | 4.8% |
| Yoon et al, 2014 | 27 | 132 | 87 | 1094 | 2.98 | [1.85; 4.79] | 4.4% |
| Mooij et al, 2018 | 272 | 780 | 213 | 770 | 1.40 | [1.13; 1.74] | 5.6% |
| Lafeber et al, 2017 | 222 | 609 | 472 | 1813 | 1.63 | [1.34; 1.98] | 5.7% |
| Olcay et al, 2003 | 36 | 570 | 21 | 340 | 1.02 | [0.59; 1.79] | 4.0% |
| Rapicetta et al, 1999 | 11 | 410 | 9 | 505 | 1.52 | [0.62; 3.70] | 2.6% |
| Wong et al, 2004 | 2 | 32 | 183 | 902 | 0.26 | [0.06; 1.11] | 1.4% |
| Taremi et al, 2007 | 3 | 11 | 27 | 388 | 5.01 | [1.26; 20.00] | 1.5% |
| Bortoliero et al, 2006 | 5 | 175 | 18 | 798 | 1.27 | [0.47; 3.48] | 2.3% |
| Feng et al, 2018 | 1045 | 1459 | 292 | 453 | 1.39 | [1.11; 1.74] | 5.6% |
| Ataei et al, 2007 | 15 | 382 | 15 | 416 | 1.09 | [0.53; 2.27] | 3.2% |
| Hesamizadeh et al, 2015 | 18 | 85 | 27 | 429 | 4.00 | [2.09; 7.66] | 3.6% |
| Sepanlou et al, 2010 | 66 | 468 | 44 | 698 | 2.44 | [1.63; 3.64] | 4.8% |
| Zuin et al, 2016 | 135 | 674 | 245 | 2872 | 2.69 | [2.13; 3.38] | 5.5% |
| Lucarelli et al, 2016 | 48 | 93 | 105 | 220 | 1.17 | [0.72; 1.90] | 4.3% |
| Labrique et al, 2009 | 189 | 776 | 83 | 358 | 1.07 | [0.79; 1.43] | 5.3% |
| Matins et al, 2014 | 21 | 339 | 3 | 92 | 1.96 | [0.57; 6.72] | 1.7% |
| Jahanbakhsh et al, 2017 | 74 | 205 | 59 | 340 | 2.69 | [1.80; 4.01] | 4.8% |
| Esquivel et al, 2014 | 97 | 265 | 2 | 6 | 1.15 | [0.21; 6.42] | 1.0% |
| Cui et al, 2016 | 61 | 227 | 167 | 798 | 1.39 | [0.99; 1.95] | 5.1% |
| Serkan et al, 2005 | 26 | 268 | 3 | 118 | 4.12 | [1.22; 13.89] | 1.8% |
| Zakieh et al, 2013 | 4 | 102 | 1 | 29 | 1.14 | [0.12; 10.64] | 0.7% |
| Niguse et al, 2018 | 262 | 638 | 97 | 208 | 0.80 | [0.58; 1.09] | 5.2% |
| Cevrioglu et al, 2004 | 27 | 177 | 4 | 68 | 2.88 | [0.97; 8.57] | 2.0% |
| Farshadpour et al, 2018 | 66 | 537 | 18 | 794 | 6.04 | [3.54; 10.30] | 4.1% |
| Adjei et al, 2009 | 23 | 82 | 21 | 75 | 1.00 | [0.50; 2.01] | 3.3% |
| Alizadeh et al, 2016 | 7 | 179 | 3 | 142 | 1.89 | [0.48; 7.43] | 1.5% |
| Abebe et al, 2017 | 50 | 145 | 72 | 241 | 1.24 | [0.80; 1.92] | 4.6% |
| **Random effects model** | | **9969** | | **15384** | **1.71** | **[1.41; 2.07]** | **100.0%** |

Heterogeneity: $I^2 = 78\%$, $\tau^2 = 0.1587$, $p < 0.01$

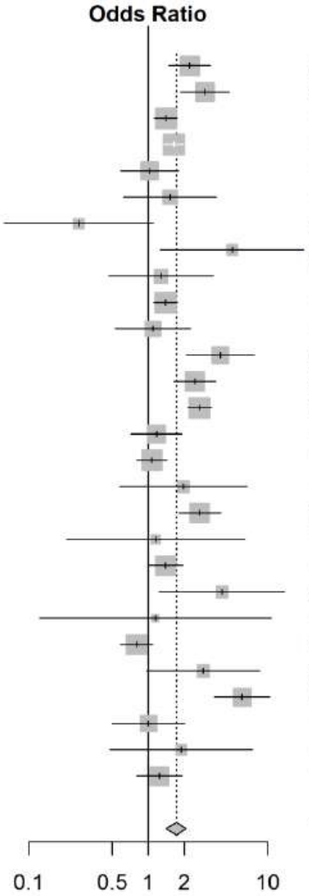

**Figure S38. Odds ratio analysis of anti-HEV IgG seroprevalence for people with or without MSM experience in general population.**

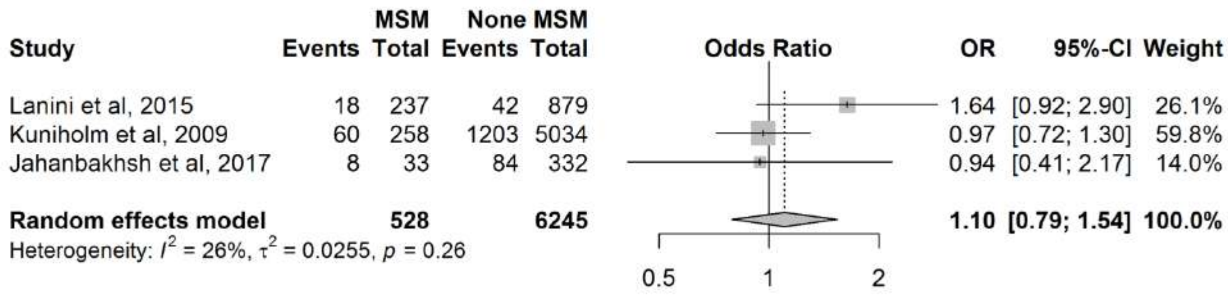


**Figure S39. Odds ratio analysis of anti-HEV IgG seroprevalence for people with or without IDU experience in general population.**

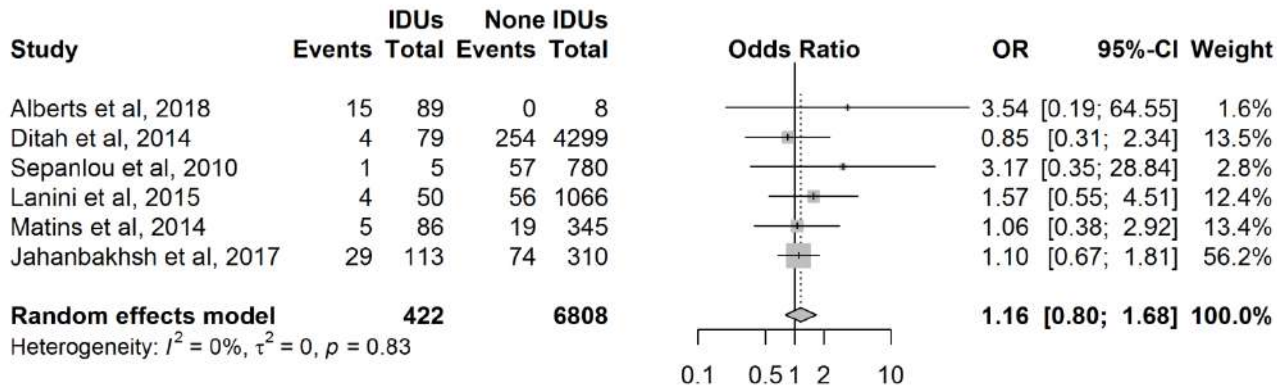

**Figure S40. Forest plot of estimated pooled anti-HEV IgG seroprevalence among general population based on different ELISA manufactures.**

Note: Those own in-house assays and uncommon commercial kits are classified as ‘Others’

| Study | Events | Total | Proportion | 95%-CI | Weight |
|---|---|---|---|---|---|
| Company = Abbott Laboratories | | | | | |
| Corwin et al, 1996 | 26 | 187 | 0.1390 | [0.0929; 0.1970] | 0.3% |
| Rapicetta et al, 1999 | 33 | 2233 | 0.0148 | [0.0102; 0.0207] | 0.4% |
| Talarmin et al, 1997 | 64 | 996 | 0.0643 | [0.0498; 0.0813] | 0.3% |
| Santos et al, 2002 | 17 | 699 | 0.0243 | [0.0142; 0.0387] | 0.3% |
| Miller et al, 1998 | 1 | 403 | 0.0025 | [0.0001; 0.0137] | 0.3% |
| Bortoliero et al, 2006 | 23 | 996 | 0.0231 | [0.0147; 0.0344] | 0.3% |
| Gessoni et al, 1996 | 49 | 1889 | 0.0259 | [0.0193; 0.0341] | 0.4% |
| Goncales et al, 2000 | 18 | 357 | 0.0504 | [0.0302; 0.0785] | 0.3% |
| Arif et al, 1994 | 160 | 1418 | 0.1128 | [0.0968; 0.1305] | 0.4% |
| Bernal et al, 1995 | 36 | 1757 | 0.0205 | [0.0144; 0.0283] | 0.4% |
| Pujol et al, 1994 | 23 | 611 | 0.0376 | [0.0240; 0.0559] | 0.3% |
| Mateos et al, 1998 | 34 | 905 | 0.0376 | [0.0262; 0.0521] | 0.3% |
| Mansuy et al, 2004 | 46 | 431 | 0.1067 | [0.0792; 0.1398] | 0.3% |
| Kamel et al, 1995 | 214 | 1259 | 0.1700 | [0.1496; 0.1919] | 0.4% |
| Langer et al, 1997 | 3 | 553 | 0.0054 | [0.0011; 0.0158] | 0.3% |
| Lavanchy et al, 1994 | 14 | 317 | 0.0442 | [0.0244; 0.0730] | 0.3% |
| Ibarra et al, 1994 | 82 | 469 | 0.1748 | [0.1415; 0.2123] | 0.3% |
| Jeggli et al, 2015 | 25 | 752 | 0.0332 | [0.0216; 0.0487] | 0.3% |
| Lachish et al, 2013 | 19 | 4970 | 0.0038 | [0.0023; 0.0060] | 0.4% |
| Alecci et al, 1997 | 3 | 244 | 0.0123 | [0.0025; 0.0355] | 0.3% |
| Olsen et al, 2006 | 10 | 108 | 0.0926 | [0.0453; 0.1637] | 0.3% |
| Gandolfo et al, 2003 | 4 | 435 | 0.0092 | [0.0025; 0.0234] | 0.3% |
| Lin et al, 2004 | 86 | 2588 | 0.0332 | [0.0267; 0.0409] | 0.4% |
| Hau et al, 1999 | 18 | 646 | 0.0279 | [0.0166; 0.0437] | 0.3% |
| Fix et al, 2000 | 4283 | 10026 | 0.4272 | [0.4175; 0.4369] | 0.4% |
| Rey et al, 1997 | 95 | 5196 | 0.0183 | [0.0148; 0.0223] | 0.4% |
| Munoz et al, 1999 | 374 | 3549 | 0.1054 | [0.0955; 0.1160] | 0.4% |
| Perez et al, 1996 | 17 | 399 | 0.0426 | [0.0250; 0.0673] | 0.3% |
| Bartoloni et al, 1999 | 36 | 490 | 0.0735 | [0.0520; 0.1003] | 0.3% |
| Leon et al, 1999 | 64 | 318 | 0.2013 | [0.1586; 0.2496] | 0.3% |
| Trinta et al,2001 | 3 | 405 | 0.0074 | [0.0015; 0.0215] | 0.3% |
| Random effects model | | 45606 | 0.0527 | [0.0207; 0.0984] | 10.8% |
| Heterogeneity: $I^2 = 100\%$, $\tau^2 = 0.0612$, $p = 0$ | | | | | |
| Company = Dia.Pro | | | | | |
| Hassan et al, 2013 | 76 | 530 | 0.1434 | [0.1147; 0.1762] | 0.3% |
| Fatemeh et al, 2014 | 235 | 510 | 0.4608 | [0.4169; 0.5051] | 0.3% |
| Cainelli et al, 2018 | 11 | 199 | 0.0553 | [0.0279; 0.0968] | 0.3% |
| Dell'Amico et al, 2011 | 12 | 123 | 0.0976 | [0.0514; 0.1642] | 0.3% |
| Yun et al, 2011 | 46 | 489 | 0.0941 | [0.0697; 0.1235] | 0.3% |
| Straková et al, 2016 | 13 | 230 | 0.0565 | [0.0304; 0.0947] | 0.3% |
| Taremi et al, 2007 | 31 | 399 | 0.0777 | [0.0534; 0.1085] | 0.3% |
| Mohebbi et al, 2012 | 51 | 551 | 0.0926 | [0.0697; 0.1199] | 0.3% |
| Parsa et al, 2016 | 50 | 700 | 0.0714 | [0.0535; 0.0931] | 0.3% |
| Teoharov et al, 2014 | 67 | 741 | 0.0904 | [0.0708; 0.1134] | 0.3% |
| Ataei et al, 2007 | 31 | 816 | 0.0380 | [0.0260; 0.0535] | 0.3% |
| Hesamizadeh et al, 2015 | 45 | 514 | 0.0875 | [0.0646; 0.1154] | 0.3% |
| Jahromi et al, 2013 | 26 | 477 | 0.0545 | [0.0359; 0.0788] | 0.3% |
| Sepanlou et al, 2010 | 105 | 1423 | 0.0738 | [0.0607; 0.0886] | 0.4% |
| Adesina et al, 2009 | 25 | 186 | 0.1344 | [0.0889; 0.1920] | 0.3% |
| Zuin et al, 2016 | 380 | 3546 | 0.1072 | [0.0972; 0.1178] | 0.4% |
| Scotto et al, 2012 | 2 | 151 | 0.0132 | [0.0016; 0.0470] | 0.3% |
| Lanini et al, 2015 | 60 | 1116 | 0.0538 | [0.0413; 0.0687] | 0.4% |
| Jahanbakhsh et al, 2017 | 136 | 562 | 0.2420 | [0.2071; 0.2796] | 0.3% |
| Esquivel et al, 2014 | 100 | 273 | 0.3663 | [0.3090; 0.4265] | 0.3% |
| Ghorbani et al, 2007 | 9 | 800 | 0.0112 | [0.0052; 0.0212] | 0.3% |
| Terzic et al, 2009 | 24 | 400 | 0.0600 | [0.0388; 0.0880] | 0.3% |
| Bayhan et al, 2016 | 17 | 408 | 0.0417 | [0.0245; 0.0659] | 0.3% |
| Saffar et al, 2009 | 25 | 1080 | 0.0231 | [0.0150; 0.0340] | 0.3% |
| Maria et al, 2010 | 38 | 1040 | 0.0365 | [0.0260; 0.0498] | 0.3% |
| Zakieh et al, 2013 | 5 | 136 | 0.0368 | [0.0120; 0.0837] | 0.3% |
| Cosme et al, 2014 | 25 | 439 | 0.0569 | [0.0372; 0.0829] | 0.3% |
| Farshadpour et al, 2018 | 83 | 1331 | 0.0624 | [0.0500; 0.0767] | 0.4% |
| Paschale et al, 2016 | 62 | 278 | 0.2230 | [0.1755; 0.2766] | 0.3% |
| Alizadeh et al, 2016 | 10 | 321 | 0.0312 | [0.0150; 0.0565] | 0.3% |
| Traore et al, 2012 | 56 | 178 | 0.3146 | [0.2472; 0.3883] | 0.3% |
| Esquivel et al, 2015 | 10 | 150 | 0.0667 | [0.0324; 0.1192] | 0.3% |
| Esquivel et al, 2015 | 25 | 439 | 0.0569 | [0.0372; 0.0829] | 0.3% |
| Wassaf et al,2014 | 19 | 433 | 0.0439 | [0.0266; 0.0677] | 0.3% |
| Munne et al, 2014 | 31 | 202 | 0.1535 | [0.1067; 0.2107] | 0.3% |
| Random effects model | | 21171 | 0.0889 | [0.0668; 0.1138] | 12.2% |
| Heterogeneity: $I^2 = 97\%$, $\tau^2 = 0.0149$, $p < 0.01$ | | | | | |

| Study | Events | Total | Proportion | 95% CI | Weight |
|---|---|---|---|---|---|
| **Company = Genelabs Diagnostics** | | | | | |
| Ahn et al, 2005 | 138 | 1030 | 0.1340 | [0.1138; 0.1563] | 0.3% |
| Boutrouille et al, 2007 | 64 | 1998 | 0.0320 | [0.0248; 0.0407] | 0.4% |
| Moaven et al, 1995 | 17 | 550 | 0.0309 | [0.0181; 0.0490] | 0.3% |
| Minuk et al, 2007 | 11 | 393 | 0.0280 | [0.0141; 0.0495] | 0.3% |
| Olcay et al, 2003 | 57 | 910 | 0.0626 | [0.0478; 0.0804] | 0.3% |
| Wong et al, 2004 | 176 | 934 | 0.1884 | [0.1638; 0.2150] | 0.3% |
| Mitsui et al, 2005 | 6 | 266 | 0.0226 | [0.0083; 0.0484] | 0.3% |
| Poovorawan et al, 1996 | 61 | 1038 | 0.0588 | [0.0452; 0.0749] | 0.3% |
| Lee et al, 1993 | 43 | 984 | 0.0437 | [0.0318; 0.0584] | 0.3% |
| Gambel et al, 1998 | 209 | 981 | 0.2130 | [0.1878; 0.2400] | 0.3% |
| Whiters et al, 2002 | 3 | 125 | 0.0240 | [0.0050; 0.0685] | 0.3% |
| Cheng et al, 2007 | 109 | 754 | 0.1446 | [0.1202; 0.1717] | 0.3% |
| Chow et al, 1996 | 30 | 219 | 0.1370 | [0.0944; 0.1897] | 0.3% |
| Colak et al, 2002 | 3 | 338 | 0.0089 | [0.0018; 0.0257] | 0.3% |
| Kaya et al, 2007 | 2 | 589 | 0.0034 | [0.0004; 0.0122] | 0.3% |
| Random effects model | | 11109 | 0.0622 | [0.0342; 0.0977] | 5.2% |
| Heterogeneity: $I^2 = 98\%$, $\tau^2 = 0.0165$, $p < 0.01$ | | | | | |
| **Company = Mikrogen** | | | | | |
| Nascimento et al, 2016 | 270 | 1656 | 0.1630 | [0.1456; 0.1817] | 0.4% |
| Vitral et al, 2014 | 50 | 388 | 0.1289 | [0.0972; 0.1663] | 0.3% |
| Fogeda et al, 2012 | 25 | 2305 | 0.0108 | [0.0070; 0.0160] | 0.4% |
| Krumbholz et al, 2011 | 18 | 116 | 0.1552 | [0.0946; 0.2341] | 0.3% |
| Krumbholz et al, 2014 | 65 | 537 | 0.1210 | [0.0947; 0.1517] | 0.3% |
| Kantala et al, 2015 | 37 | 385 | 0.0961 | [0.0686; 0.1300] | 0.3% |
| Korzeniewski et al, 2018 | 13 | 253 | 0.0514 | [0.0276; 0.0863] | 0.3% |
| Matins et al, 2014 | 24 | 431 | 0.0557 | [0.0360; 0.0817] | 0.3% |
| Schielke et al, 2015 | 26 | 126 | 0.2063 | [0.1394; 0.2875] | 0.3% |
| Todys et al, 2015 | 206 | 1027 | 0.2006 | [0.1765; 0.2264] | 0.3% |
| Teixeira et al, 2017 | 106 | 804 | 0.1318 | [0.1092; 0.1572] | 0.3% |
| Galiana et al, 2008 | 4 | 97 | 0.0412 | [0.0113; 0.1022] | 0.3% |
| Krumbholz et al, 2014 | 16 | 1646 | 0.0097 | [0.0056; 0.0157] | 0.4% |
| Oliveira et al, 2017 | 4 | 352 | 0.0114 | [0.0031; 0.0288] | 0.3% |
| Kyvernitakis et al, 2015 | 12 | 115 | 0.1043 | [0.0551; 0.1752] | 0.3% |
| Random effects model | | 10238 | 0.0860 | [0.0465; 0.1362] | 5.2% |
| Heterogeneity: $I^2 = 98\%$, $\tau^2 = 0.0242$, $p < 0.01$ | | | | | |
| **Company = MP Diagnostics** | | | | | |
| Verhoef et al, 2011 | 134 | 7072 | 0.0189 | [0.0159; 0.0224] | 0.4% |
| Vivek et al, 2010 | 330 | 2279 | 0.1448 | [0.1306; 0.1599] | 0.4% |
| Anita et al, 2014 | 22 | 148 | 0.1486 | [0.0956; 0.2164] | 0.3% |
| Houcine et al, 2012 | 37 | 687 | 0.0539 | [0.0382; 0.0735] | 0.3% |
| Jia et al, 2014 | 3721 | 15862 | 0.2346 | [0.2280; 0.2413] | 0.4% |
| Kaufmann et al, 2011 | 27 | 550 | 0.0491 | [0.0326; 0.0706] | 0.3% |
| Meader et al, 2008 | 10 | 413 | 0.0242 | [0.0117; 0.0441] | 0.3% |
| Chaussade et al, 2013 | 83 | 322 | 0.2578 | [0.2109; 0.3092] | 0.3% |
| Widasari et al, 2013 | 16 | 277 | 0.0578 | [0.0334; 0.0921] | 0.3% |
| Pathak et al, 2018 | 341 | 1369 | 0.2491 | [0.2264; 0.2729] | 0.4% |
| Bawazir et al, 2010 | 38 | 356 | 0.1067 | [0.0767; 0.1436] | 0.3% |
| Random effects model | | 29335 | 0.1075 | [0.0455; 0.1915] | 3.8% |
| Heterogeneity: $I^2 = 100\%$, $\tau^2 = 0.0400$, $p = 0$ | | | | | |
| **Company = Others** | | | | | |
| Maria et al, 2006 | 96 | 1280 | 0.0750 | [0.0612; 0.0908] | 0.4% |
| Edward et al, 1997 | 571 | 757 | 0.7543 | [0.7220; 0.7846] | 0.3% |
| Azarkar et al, 2017 | 139 | 554 | 0.2509 | [0.2153; 0.2892] | 0.3% |
| Christensen et al,2018 | 293 | 906 | 0.3234 | [0.2930; 0.3549] | 0.3% |
| Abe et al, 2006 | 1720 | 5233 | 0.3287 | [0.3160; 0.3416] | 0.4% |
| Assarehzadegan et al, 2007 | 46 | 400 | 0.1150 | [0.0854; 0.1504] | 0.3% |
| Takahashi et al, 2004 | 28 | 249 | 0.1124 | [0.0760; 0.1584] | 0.3% |
| Takahashi et al, 2010 | 1167 | 22027 | 0.0530 | [0.0501; 0.0560] | 0.4% |
| Zhuang et al, 2014 | 113 | 486 | 0.2325 | [0.1956; 0.2727] | 0.3% |
| Takeda et al, 2010 | 431 | 12600 | 0.0342 | [0.0311; 0.0375] | 0.4% |
| Petrovi et al, 2014 | 30 | 200 | 0.1500 | [0.1035; 0.2072] | 0.3% |
| Tripathy et al, 2018 | 433 | 2447 | 0.1770 | [0.1620; 0.1927] | 0.4% |
| Quintana et al, 2005 | 11 | 209 | 0.0526 | [0.0266; 0.0922] | 0.3% |
| Sakata et al, 2008 | 45 | 1389 | 0.0324 | [0.0237; 0.0431] | 0.4% |
| Mor et al, 2015 | 77 | 729 | 0.1056 | [0.0843; 0.1302] | 0.3% |
| Villalba et al, 2014 | 47 | 469 | 0.1002 | [0.0746; 0.1310] | 0.3% |
| Toyoda et al, 2008 | 60 | 592 | 0.1014 | [0.0782; 0.1285] | 0.3% |
| Mohammad et al, 2017 | 139 | 450 | 0.3089 | [0.2665; 0.3538] | 0.3% |
| Yamada et al, 2015 | 160 | 868 | 0.1843 | [0.1591; 0.2118] | 0.3% |
| Peng et al, 1995 | 76 | 997 | 0.0762 | [0.0605; 0.0945] | 0.3% |
| Ren et al, 2013 | 2945 | 10741 | 0.2742 | [0.2658; 0.2827] | 0.4% |
| Pittaras et al, 2014 | 25 | 265 | 0.0943 | [0.0620; 0.1361] | 0.3% |
| Jahromi et al, 2013 | 26 | 477 | 0.0545 | [0.0359; 0.0788] | 0.3% |
| Fukuda et al, 2007 | 168 | 3185 | 0.0527 | [0.0452; 0.0611] | 0.4% |
| Cangin et al, 2018 | 377 | 7656 | 0.0492 | [0.0445; 0.0543] | 0.4% |

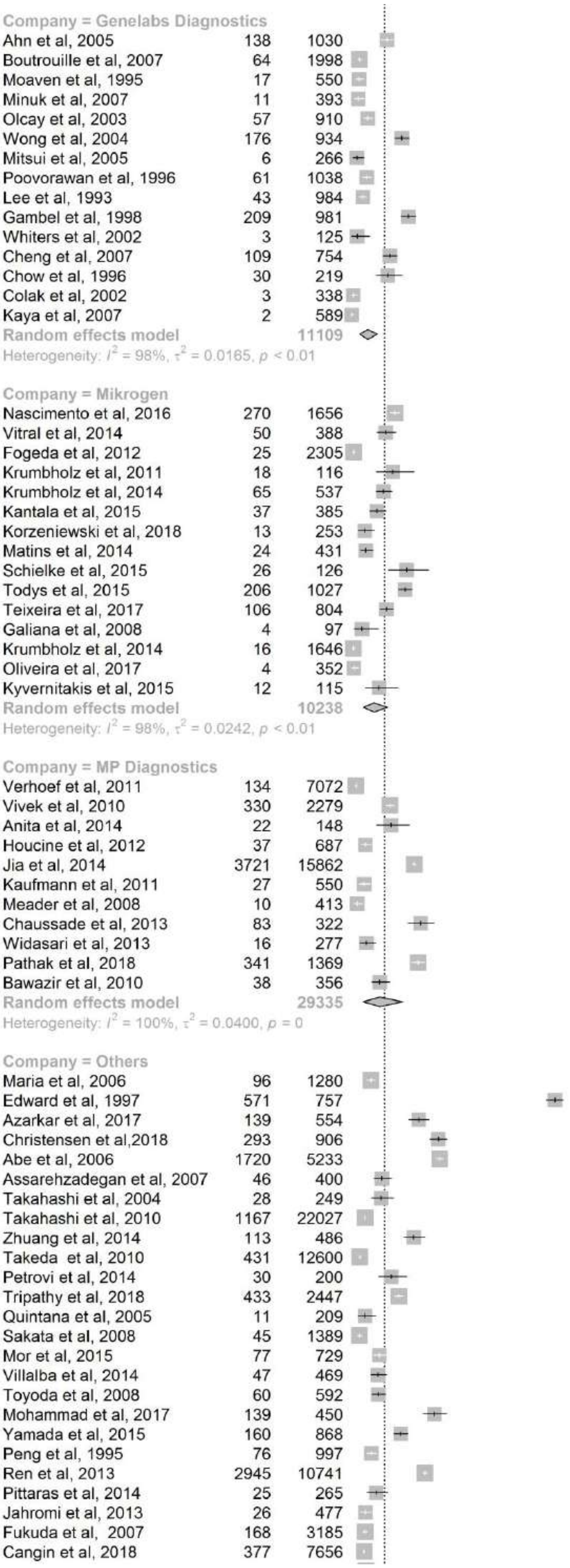

| | | | | | |
|---|---|---|---|---|---|
| Ditah et al, 2014 | 490 | 8814 | 0.0556 | [0.0509; 0.0606] | 0.4% |
| Paula et al, 2001 | 10 | 255 | 0.0392 | [0.0190; 0.0709] | 0.3% |
| Chiu et al, 2013 | 129 | 450 | 0.2867 | [0.2453; 0.3309] | 0.3% |
| Ding et al, 2003 | 15 | 533 | 0.0281 | [0.0158; 0.0460] | 0.3% |
| Chan et al, 2017 | 492 | 1539 | 0.3197 | [0.2964; 0.3436] | 0.4% |
| Abdelaal et al, 1995 | 100 | 593 | 0.1686 | [0.1394; 0.2012] | 0.3% |
| Amarapurkar et al, 2008 | 416 | 1163 | 0.3577 | [0.3301; 0.3860] | 0.4% |
| Grabow et al, 1994 | 16 | 782 | 0.0205 | [0.0117; 0.0330] | 0.3% |
| Ricco et al, 2016 | 9 | 199 | 0.0452 | [0.0209; 0.0841] | 0.3% |
| Puttini et al, 2015 | 12 | 132 | 0.0909 | [0.0479; 0.1534] | 0.3% |
| Pavia et al, 1998 | 3 | 360 | 0.0083 | [0.0017; 0.0242] | 0.3% |
| Raoofi et al, 2012 | 31 | 400 | 0.0775 | [0.0533; 0.1082] | 0.3% |
| Rahman et al, 2014 | 90 | 300 | 0.3000 | [0.2487; 0.3553] | 0.3% |
| Sabato et al, 2017 | 4 | 253 | 0.0158 | [0.0043; 0.0400] | 0.3% |
| Chen et al, 2012 | 2793 | 14208 | 0.1966 | [0.1901; 0.2032] | 0.4% |
| Love et al, 2018 | 6 | 291 | 0.0206 | [0.0076; 0.0443] | 0.3% |
| Lopes et al, 2017 | 78 | 300 | 0.2600 | [0.2113; 0.3135] | 0.3% |
| Karetny et al, 1995 | 37 | 1416 | 0.0261 | [0.0185; 0.0358] | 0.4% |
| Kotwal et al, 2014 | 712 | 4175 | 0.1705 | [0.1592; 0.1823] | 0.4% |
| Karetnyi et al, 1999 | 36 | 734 | 0.0490 | [0.0346; 0.0673] | 0.3% |
| Johargy et al, 2013 | 168 | 900 | 0.1867 | [0.1617; 0.2137] | 0.3% |
| Konomi et al, 1999 | 93 | 574 | 0.1620 | [0.1328; 0.1948] | 0.3% |
| Intharasongkroh et al, 2019 | 0 | 1019 | 0.0000 | [0.0000; 0.0036] | 0.3% |
| Nagler et al, 2014 | 143 | 997 | 0.1434 | [0.1222; 0.1667] | 0.3% |
| Liu et al, 2019 | 3419 | 10256 | 0.3334 | [0.3242; 0.3426] | 0.4% |
| Kuniholm et al, 2009 | 3916 | 18695 | 0.2095 | [0.2037; 0.2154] | 0.4% |
| Khounvisith et al, 2018 | 38 | 210 | 0.1810 | [0.1313; 0.2398] | 0.3% |
| Labrique et al, 2009 | 255 | 1134 | 0.2249 | [0.2009; 0.2503] | 0.4% |
| Kaba et al, 2010 | 57 | 490 | 0.1163 | [0.0893; 0.1481] | 0.3% |
| Chironna et al, 1999 | 20 | 526 | 0.0380 | [0.0234; 0.0581] | 0.3% |
| Rapicetta et al,1999 | 113 | 973 | 0.1161 | [0.0967; 0.1380] | 0.3% |
| Nübling et al, 2002 | 20 | 511 | 0.0391 | [0.0241; 0.0598] | 0.3% |
| Pilakasiri et al, 2009 | 44 | 381 | 0.1155 | [0.0852; 0.1519] | 0.3% |
| Bura et al, 2018 | 2 | 138 | 0.0145 | [0.0018; 0.0514] | 0.3% |
| Dremsek et al, 2012 | 132 | 853 | 0.1547 | [0.1311; 0.1808] | 0.3% |
| Eick et al, 2010 | 16 | 1500 | 0.0107 | [0.0061; 0.0173] | 0.4% |
| Bouwknegt et al, 2008 | 12 | 644 | 0.0186 | [0.0097; 0.0323] | 0.3% |
| Drobeniuc et al, 2001 | 63 | 255 | 0.2471 | [0.1954; 0.3047] | 0.3% |
| Sheikh et al, 2002 | 164 | 273 | 0.6007 | [0.5400; 0.6593] | 0.3% |
| Abe et al, 2004 | 62 | 341 | 0.1818 | [0.1423; 0.2269] | 0.3% |
| Aboulata et al, 2005 | 26 | 100 | 0.2600 | [0.1774; 0.3573] | 0.3% |
| Achwan et al, 2007 | 34 | 581 | 0.0585 | [0.0409; 0.0808] | 0.3% |
| Atabek et al, 2004 | 12 | 210 | 0.0571 | [0.0299; 0.0977] | 0.3% |
| Buti et al, 2008 | 57 | 1249 | 0.0456 | [0.0347; 0.0587] | 0.4% |
| Cevahir et al, 2013 | 23 | 185 | 0.1243 | [0.0805; 0.1807] | 0.3% |
| Davaalkham et al, 2009 | 3 | 520 | 0.0058 | [0.0012; 0.0168] | 0.3% |
| Ekanem et al, 2015 | 31 | 402 | 0.0771 | [0.0530; 0.1077] | 0.3% |
| Goto et al, 2006 | 9 | 344 | 0.0262 | [0.0120; 0.0491] | 0.3% |
| Hasan et al, 2016 | 33 | 123 | 0.2683 | [0.1924; 0.3557] | 0.3% |
| Mathur et al, 2001 | 545 | 2070 | 0.2633 | [0.2444; 0.2828] | 0.4% |
| Mohanavalli et al, 2002 | 16 | 185 | 0.0865 | [0.0502; 0.1366] | 0.3% |
| Sencan et al, 2003 | 34 | 476 | 0.0714 | [0.0500; 0.0984] | 0.3% |
| Shamsizadeh et al, 2009 | 48 | 566 | 0.0848 | [0.0632; 0.1109] | 0.3% |
| Sidal et al, 2001 | 19 | 909 | 0.0209 | [0.0126; 0.0324] | 0.3% |
| Bira et al, 2007 | 5 | 717 | 0.0070 | [0.0023; 0.0162] | 0.3% |
| Dong et al, 2007 | 2073 | 12052 | 0.1720 | [0.1653; 0.1789] | 0.4% |
| Ijaz et al, 2009 | 362 | 2731 | 0.1326 | [0.1201; 0.1458] | 0.4% |
| Héctor et al, 2017 | 1 | 127 | 0.0079 | [0.0002; 0.0431] | 0.3% |
| Serkan et al, 2005 | 27 | 386 | 0.0699 | [0.0466; 0.1001] | 0.3% |
| Dorcas et al, 2018 | 48 | 393 | 0.1221 | [0.0914; 0.1587] | 0.3% |
| Sonia et al, 2006 | 2046 | 2428 | 0.8427 | [0.8276; 0.8569] | 0.4% |
| Caron et al, 2008 | 119 | 840 | 0.1417 | [0.1188; 0.1671] | 0.3% |
| Caron et al, 2012 | 16 | 243 | 0.0658 | [0.0381; 0.1047] | 0.3% |
| Taha et al, 2015 | 80 | 397 | 0.2015 | [0.1631; 0.2444] | 0.3% |
| Cevrioglu et al, 2004 | 31 | 245 | 0.1265 | [0.0876; 0.1748] | 0.3% |
| Gad et al, 2011 | 68 | 116 | 0.5862 | [0.4910; 0.6769] | 0.3% |
| Adjei et al, 2009 | 16 | 157 | 0.1019 | [0.0594; 0.1602] | 0.3% |
| Huang et al, 2013 | 30 | 293 | 0.1024 | [0.0702; 0.1429] | 0.3% |
| Aubry et al, 1997 | 18 | 129 | 0.1395 | [0.0848; 0.2115] | 0.3% |
| Gambel et al, 1998 | 14 | 112 | 0.1250 | [0.0701; 0.2008] | 0.3% |
| Zhang et al, 2015 | 1352 | 6262 | 0.2159 | [0.2058; 0.2263] | 0.4% |
| Tashale et al, 2015 | 1081 | 13851 | 0.0780 | [0.0736; 0.0826] | 0.4% |
| Stramer et al, 2015 | 27 | 200 | 0.1350 | [0.0909; 0.1903] | 0.3% |
| Meng et al, 2001 | 73 | 400 | 0.1825 | [0.1459; 0.2239] | 0.3% |
| Lemos et al, 2000 | 16 | 1149 | 0.0139 | [0.0080; 0.0225] | 0.4% |
| **Random effects model** | | 203881 | 0.1243 | [0.1017; 0.1489] | 34.8% |

Heterogeneity: $I^2 = 100\%$, $\tau^2 = 0.0327$, $p = 0$

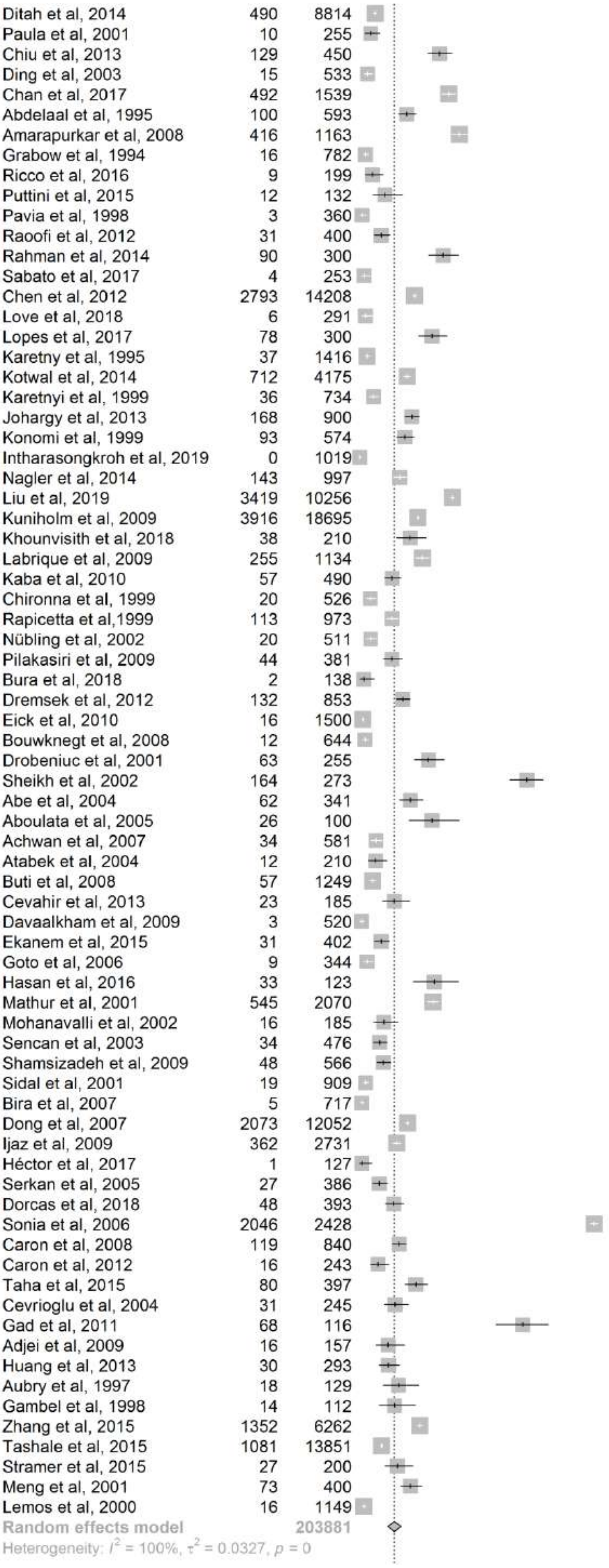

| Study | Events | Total | Proportion | 95%-CI | Weight |
|---|---|---|---|---|---|
| Company = Wantai | | | | | |
| Chang et al, 2009 | 522 | 2572 | 0.2030 | [0.1876; 0.2190] | 0.4% |
| Geng et al, 2011 | 538 | 2682 | 0.2006 | [0.1856; 0.2163] | 0.4% |
| Yuan et al, 2017 | 268 | 1200 | 0.2233 | [0.2001; 0.2480] | 0.4% |
| Chen et al, 2019 | 799 | 4044 | 0.1976 | [0.1854; 0.2102] | 0.4% |
| Guo et al, 2009 | 14610 | 44816 | 0.3260 | [0.3217; 0.3304] | 0.4% |
| Halliday et al, 2014 | 120 | 1048 | 0.1145 | [0.0959; 0.1354] | 0.3% |
| Baylis et al, 2012 | 1 | 165010 | 0.0000 | [0.0000; 0.0000] | 0.4% |
| Yoon et al, 2014 | 144 | 2450 | 0.0588 | [0.0498; 0.0688] | 0.4% |
| O'Riordan et al, 2016 | 57 | 1076 | 0.0530 | [0.0404; 0.0681] | 0.3% |
| Spada et al, 2018 | 869 | 10011 | 0.0868 | [0.0814; 0.0925] | 0.4% |
| Nasrallah et al, 2017 | 1198 | 5854 | 0.2046 | [0.1944; 0.2152] | 0.4% |
| Nouhin et al, 2016 | 85 | 301 | 0.2824 | [0.2322; 0.3369] | 0.3% |
| Castilho et al, 2017 | 49 | 500 | 0.0980 | [0.0734; 0.1275] | 0.3% |
| Mooij et al, 2018 | 648 | 2100 | 0.3086 | [0.2889; 0.3288] | 0.4% |
| Lafeber et al, 2017 | 666 | 2422 | 0.2750 | [0.2573; 0.2932] | 0.4% |
| Nouhin et al, 2018 | 829 | 2004 | 0.4137 | [0.3920; 0.4356] | 0.4% |
| Slot et al, 2013 | 1401 | 5239 | 0.2674 | [0.2555; 0.2796] | 0.4% |
| Meldal et al, 2013 | 11 | 239 | 0.0460 | [0.0232; 0.0809] | 0.3% |
| Wenzel et al, 2014 | 929 | 2184 | 0.4254 | [0.4045; 0.4464] | 0.4% |
| Wang et al, 2017 | 2682 | 9069 | 0.2957 | [0.2864; 0.3052] | 0.4% |
| Castilho et al, 2016 | 30 | 300 | 0.1000 | [0.0685; 0.1397] | 0.3% |
| Zhu et al, 2008 | 1289 | 4654 | 0.2770 | [0.2641; 0.2901] | 0.4% |
| Sadik et al, 2016 | 459 | 1199 | 0.3828 | [0.3552; 0.4110] | 0.4% |
| Yang et al, 2014 | 473 | 2028 | 0.2332 | [0.2150; 0.2523] | 0.4% |
| Zhu et al, 2014 | 152061 | 400162 | 0.3800 | [0.3785; 0.3815] | 0.4% |
| Thom et al, 2017 | 104 | 2311 | 0.0450 | [0.0369; 0.0543] | 0.4% |
| Shrestha et al, 2016 | 773 | 1845 | 0.4190 | [0.3963; 0.4419] | 0.4% |
| Sauleda et al, 2014 | 1996 | 9998 | 0.1996 | [0.1918; 0.2076] | 0.4% |
| Schreuder et al, 2016 | 25 | 600 | 0.0417 | [0.0271; 0.0609] | 0.3% |
| Niederhauser et al, 2017 | 737 | 3609 | 0.2042 | [0.1912; 0.2177] | 0.4% |
| Hewitt et al, 2018 | 98 | 1013 | 0.0967 | [0.0792; 0.1166] | 0.3% |
| Alberts et al, 2018 | 190 | 7065 | 0.0269 | [0.0232; 0.0309] | 0.4% |
| Grabarczyk et al, 2018 | 1340 | 3079 | 0.4352 | [0.4176; 0.4529] | 0.4% |
| Fischer et al, 2014 | 163 | 1203 | 0.1355 | [0.1166; 0.1561] | 0.4% |
| Feng et al, 2018 | 1273 | 1912 | 0.6658 | [0.6441; 0.6869] | 0.4% |
| Fearon et al, 2017 | 241 | 4102 | 0.0588 | [0.0517; 0.0664] | 0.4% |
| Harvala et al, 2018 | 42 | 480 | 0.0875 | [0.0638; 0.1164] | 0.3% |
| Cleland et al, 2013 | 73 | 1559 | 0.0468 | [0.0369; 0.0585] | 0.4% |
| Cai et al, 2016 | 1842 | 3337 | 0.5520 | [0.5349; 0.5690] | 0.4% |
| Dimeglio et al, 2018 | 23 | 300 | 0.0767 | [0.0492; 0.1128] | 0.3% |
| Hardtke et al, 2018 | 91 | 408 | 0.2230 | [0.1836; 0.2666] | 0.3% |
| Hogema et al, 2016 | 11 | 45 | 0.2444 | [0.1288; 0.3954] | 0.3% |
| Hickey et al, 2016 | 16 | 198 | 0.0808 | [0.0469; 0.1279] | 0.3% |
| Madden et al, 2016 | 324 | 1161 | 0.2791 | [0.2534; 0.3058] | 0.4% |
| Mansuy et al, 2016 | 2371 | 10569 | 0.2243 | [0.2164; 0.2324] | 0.4% |
| Lucarelli et al, 2016 | 153 | 313 | 0.4888 | [0.4322; 0.5457] | 0.3% |
| Lange et al, 2016 | 177 | 1263 | 0.1401 | [0.1215; 0.1605] | 0.4% |
| Liang et al, 2014 | 129 | 307 | 0.4202 | [0.3644; 0.4776] | 0.3% |
| Marcantonio et al, 2018 | 10 | 198 | 0.0505 | [0.0245; 0.0909] | 0.3% |
| Ma et al, 2010 | 407 | 2090 | 0.1947 | [0.1780; 0.2124] | 0.4% |
| Mansuy et al, 2015 | 1310 | 3353 | 0.3907 | [0.3741; 0.4074] | 0.4% |
| Izopet et al, 2015 | 1083 | 2538 | 0.4267 | [0.4074; 0.4462] | 0.4% |
| Li et al, 2011 | 69 | 173 | 0.3988 | [0.3253; 0.4759] | 0.3% |
| Li et al, 2014 | 197 | 1638 | 0.1203 | [0.1049; 0.1370] | 0.4% |
| Juhl et al, 2017 | 70 | 449 | 0.1559 | [0.1236; 0.1928] | 0.3% |
| Katiyar et al, 2018 | 383 | 633 | 0.6051 | [0.5658; 0.6434] | 0.3% |
| Juhl et al, 2014 | 69 | 1019 | 0.0677 | [0.0531; 0.0849] | 0.3% |
| Mahrt et al, 2018 | 679 | 3000 | 0.2263 | [0.2115; 0.2417] | 0.4% |
| Kang et al, 2017 | 121 | 600 | 0.2017 | [0.1703; 0.2360] | 0.3% |
| Li et al, 2006 | 3154 | 7284 | 0.4330 | [0.4216; 0.4445] | 0.4% |
| Jablonka et al, 2017 | 122 | 604 | 0.2020 | [0.1707; 0.2363] | 0.3% |
| Azman et al, 2017 | 154 | 206 | 0.7476 | [0.6825; 0.8054] | 0.3% |
| Cui et al, 2016 | 228 | 1028 | 0.2218 | [0.1967; 0.2485] | 0.3% |
| Tabili et al, 2013 | 23 | 103 | 0.2233 | [0.1471; 0.3160] | 0.3% |
| Zeng et al, 2014 | 26 | 513 | 0.0507 | [0.0334; 0.0734] | 0.3% |
| Yu et al, 2009 | 841 | 3994 | 0.2106 | [0.1980; 0.2235] | 0.4% |
| Zhang et al, 2017 | 236 | 600 | 0.3933 | [0.3540; 0.4337] | 0.3% |
| Dalton et al, 2017 | 11 | 464 | 0.0237 | [0.0119; 0.0420] | 0.3% |
| Cong et al, 2014 | 208 | 890 | 0.2337 | [0.2063; 0.2629] | 0.3% |
| Meng et al, 2015 | 174 | 1500 | 0.1160 | [0.1002; 0.1333] | 0.4% |
| Toole et al, 2006 | 132 | 426 | 0.3099 | [0.2662; 0.3562] | 0.3% |
| Lu et al, 2009 | 1547 | 8762 | 0.1766 | [0.1686; 0.1847] | 0.4% |
| Li et al, 2019 | 139 | 946 | 0.1469 | [0.1250; 0.1711] | 0.3% |
| Lee et al, 2013 | 2 | 100 | 0.0200 | [0.0024; 0.0704] | 0.3% |
| Christophe et al, 2004 | 24 | 315 | 0.0762 | [0.0494; 0.1112] | 0.3% |
| Rui et al, 2018 | 49 | 225 | 0.2178 | [0.1657; 0.2775] | 0.3% |
| Niguse et al, 2018 | 359 | 846 | 0.4243 | [0.3908; 0.4585] | 0.3% |
| Abebe et al, 2017 | 122 | 386 | 0.3161 | [0.2700; 0.3650] | 0.3% |
| Xu et al,2013 | 364 | 1939 | 0.1877 | [0.1706; 0.2058] | 0.4% |
| Katrina et al,2018 | 104 | 1714 | 0.0607 | [0.0498; 0.0730] | 0.4% |
| Random effects model | | 778377 | 0.2072 | [0.1523; 0.2680] | 27.9% |
| Heterogeneity: $I^2$ = 100%, $\tau^2$ = 0.1062, $p$ = 0 | | | | | |
| **Random effects model** | | **1099717** | **0.1247** | **[0.1042; 0.1467]** | **100.0%** |
| Heterogeneity: $I^2$ = 100%, $\tau^2$ = 0.0769, $p$ = 0 | | | | | |
| Residual heterogeneity: $I^2$ = 100%, $p$ = 0 | | | | | |

0 0.2 0.4 0.6 0.8

**Figure S41. Funnel plot for anti-HEV IgG seroprevalence among general population.**

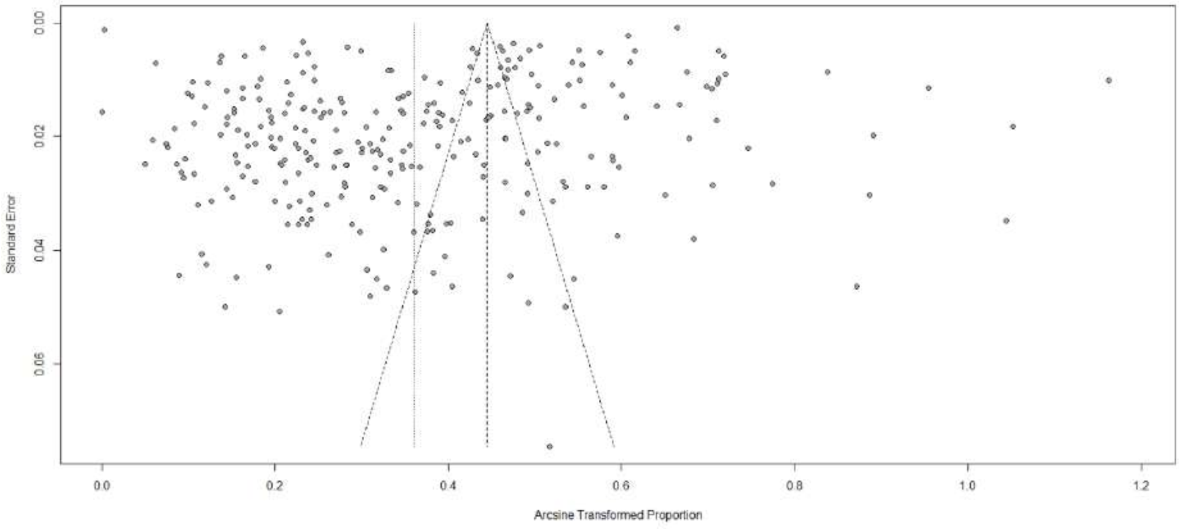


**Figure S42. Egger plot for anti-HEV IgG seroprevalence among general population.**

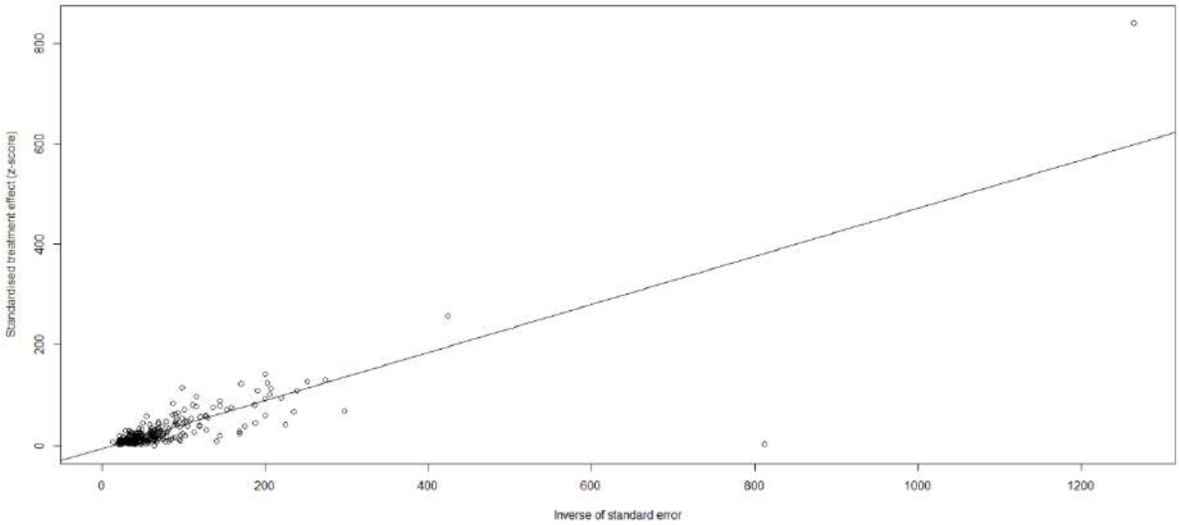

**Table S1. Score of studies evaluated by JBI Critical Appraisal Tools.**

1. Was the sample frame appropriate to address the target population?

2. Were study participants sampled in an appropriate way?

3. Was the sample size adequate?

4. Were the study subjects and the setting described in detail?

5. Was the data analysis conducted with sufficient coverage of the identified sample?

6. Were valid methods used for the identification of the condition?

7. Was the condition measured in a standard, reliable way for all participants?

8. Was there appropriate statistical analysis?

9. Was the response rate adequate, and if not, was the low response rate managed appropriately?

| Study | 1 | 2 | 3 | 4 | 5 | 6 | 7 | 8 | 9 | Score |
|---|---|---|---|---|---|---|---|---|---|---|
| Legrand-Abravanel et al,2011 | ✓ | ✓ | ✓ | ✓ | ✓ | ✓ | ? | ✓ | ✓ | 8 |
| Haagsma et al,2009 | ✓ | ✓ | ✓ | ✓ | ✓ | ✓ | ? | ✓ | ✓ | 8 |
| Hoerning et al,2012 | ✓ | ? | ✓ | ? | ✓ | ✓ | ? | ✓ | ✓ | 6 |
| Pischke et al,2010 | ✓ | ✓ | ✓ | ? | ✓ | ✓ | ✓ | ✓ | ✓ | 8 |
| Halac et al,2012 | ✓ | ✓ | ✓ | ✓ | ✓ | ✓ | ? | ✓ | ✓ | 8 |
| Rostamzadeh et al,2011 | ✓ | ✓ | ✓ | ✓ | ✓ | ✓ | ? | ✓ | ✓ | 8 |
| Pischke et al, 2012 | ✓ | ✓ | ✓ | ✓ | ✓ | ✓ | ? | ✓ | ✓ | 8 |
| Scotto et al,2015 | ✓ | ? | ✓ | ✓ | ✓ | ✓ | ? | ✓ | ✓ | 7 |
| Pisano et al,2017 | ✓ | ✓ | ✓ | ✓ | ✓ | ✓ | ? | ✓ | ✓ | 7 |
| Nanmoku et al,2019 | ✓ | ✓ | ✓ | ✓ | ✓ | ✓ | ? | ✓ | ✓ | 8 |
| Unzueta et al,2016 | ✓ | ✓ | ✓ | ✓ | ✓ | ✓ | ? | ✓ | ✓ | 8 |
| Naik et al,2013 | ✓ | ? | ✓ | - | ✓ | ✓ | ? | ✓ | ✓ | 6 |
| Moal et al,2013 | ✓ | ✓ | ✓ | ✓ | ✓ | ✓ | ? | ✓ | ✓ | 8 |
| Koning et al,2015 | ✓ | ✓ | ✓ | ✓ | ✓ | ✓ | ✓ | ✓ | ✓ | 9 |
| Inagaki et al,2015 | ✓ | ✓ | ✓ | ✓ | ✓ | ✓ | ✓ | ✓ | ✓ | 9 |
| Mallet et al,2018 | ✓ | ✓ | ✓ | ✓ | ✓ | ✓ | ✓ | ✓ | ✓ | 9 |
| de Oliveira et al,2018 | ✓ | ✓ | ✓ | ✓ | ✓ | ✓ | ✓ | ✓ | ✓ | 9 |
| Buffaz et al, 2014 | ✓ | ? | ✓ | ✓ | ✓ | ✓ | ? | ✓ | ✓ | 7 |
| Abravanel et al,2014 | ✓ | ✓ | ✓ | ✓ | ✓ | ✓ | ? | ✓ | ✓ | 8 |
| Pas et al,2012 | ✓ | ✓ | ✓ | ✓ | ✓ | ✓ | ✓ | ✓ | ✓ | 9 |
| Riezebos-Brilman et al,2013 | ✓ | ✓ | ✓ | - | ✓ | ✓ | ? | ✓ | ✓ | 7 |
| Passos et al,2013 | ✓ | ? | ✓ | ✓ | ✓ | ✓ | ? | ✓ | ✓ | 7 |
| Ankcorn et al,2018 | ✓ | ✓ | ✓ | ✓ | ✓ | ✓ | ? | ✓ | ✓ | 8 |
| Kamar et al,2008 | ✓ | ✓ | ✓ | ✓ | ✓ | ✓ | ? | ✓ | ✓ | 8 |
| Moal et al,2013 | ✓ | ✓ | ✓ | ✓ | ✓ | ✓ | ? | ✓ | ✓ | 8 |
| Lim et al,2018 | ✓ | ✓ | ✓ | ✓ | ✓ | ✓ | ? | ✓ | ✓ | 8 |
| Buti et al,2010 | ✓ | ✓ | ✓ | ✓ | ✓ | ✓ | ? | ✓ | ✓ | 8 |
| Laverdure et al, 2015 | ✓ | ✓ | ✓ | ✓ | ✓ | ✓ | ? | ✓ | ✓ | 8 |
| Magnusson et al,2015 | ✓ | ✓ | ✓ | ✓ | ✓ | ✓ | ? | ✓ | ✓ | 8 |
| Lee et al,2005 | ✓ | ✓ | ✓ | ✓ | ✓ | ✓ | ✓ | ✓ | ✓ | 9 |
| Psichogiou et al,1996 | ✓ | ✓ | ✓ | - | ✓ | ✓ | ✓ | ✓ | ✓ | 8 |
| Dalekos et al,1998 | ✓ | ? | ✓ | × | ✓ | ✓ | ✓ | ✓ | ✓ | 7 |
| Abdel et al,1998 | ✓ | ✓ | ✓ | ✓ | ✓ | ✓ | ✓ | ✓ | ✓ | 9 |
| Harrison et al,2013 | ✓ | ✓ | ✓ | ✓ | ✓ | ✓ | ✓ | ✓ | ✓ | 9 |
| Ding et al,2003 | ✓ | ✓ | ✓ | ✓ | ✓ | ✓ | ✓ | ✓ | ✓ | 9 |
| Mitsui et al,2006 | ✓ | ✓ | ✓ | ✓ | ✓ | ✓ | ✓ | ✓ | ✓ | 9 |
| Ben-Ayed et al,2015 | ✓ | ✓ | ✓ | ✓ | ✓ | ✓ | ? | ✓ | ✓ | 8 |
| Ricco et al,2016 | ✓ | ✓ | ✓ | ✓ | ✓ | ✓ | ? | ✓ | ✓ | 8 |
| Chen et al,2016 | ✓ | ✓ | ✓ | ✓ | ✓ | ✓ | ✓ | ✓ | ✓ | 9 |
| Knodler et al,1994 | ✓ | ✓ | ✓ | - | ✓ | ✓ | ? | ✓ | ✓ | 7 |
| Buti et al,1995 | ✓ | ✓ | ✓ | ✓ | ✓ | ✓ | ? | ✓ | ✓ | 8 |
| Cengiz et al,1996 | ✓ | ✓ | ✓ | ✓ | ✓ | ✓ | ? | ✓ | ✓ | 8 |
| Parana et al,1997 | ✓ | ✓ | ✓ | ✓ | ✓ | ✓ | ? | ✓ | ✓ | 8 |

| | | | | | | | | | | |
|---|---|---|---|---|---|---|---|---|---|---|
| Arinsoy et al,1998 | ✓ | ? | ✓ | - | ✓ | ✓ | ? | ✓ | ✓ | 7 |
| Mateos et al,1999 | ✓ | ? | ✓ | ✓ | ✓ | ✓ | ? | ✓ | ✓ | 7 |
| Trinta et al,2001 | ✓ | ✓ | ✓ | × | ✓ | ✓ | ? | ✓ | ✓ | 7 |
| Alavian et al, 2015 | ✓ | ✓ | ✓ | ✓ | ✓ | ✓ | ? | ✓ | ✓ | 8 |
| Halfon et al,1994 | ✓ | ✓ | ✓ | ✓ | ✓ | ✓ | ? | ✓ | ✓ | 8 |
| Kikuchi et al, 2006 | ✓ | ✓ | ✓ | ✓ | ✓ | ✓ | ✓ | ✓ | ✓ | 9 |
| Sylvan et al,1998 | ✓ | ✓ | ✓ | ✓ | ✓ | ✓ | ✓ | ✓ | ✓ | 9 |
| Taremi et al,2005 | ✓ | ✓ | ✓ | ✓ | ✓ | ✓ | ✓ | ✓ | ✓ | 9 |
| Zekavat et al,2013 | ✓ | ✓ | ✓ | ✓ | ✓ | ✓ | ✓ | ✓ | ✓ | 9 |
| Ayoola et al,2015 | ✓ | ✓ | ✓ | ✓ | ✓ | ✓ | ? | ✓ | ✓ | 8 |
| Javani et al,2015 | ✓ | ✓ | ✓ | ✓ | ✓ | ✓ | ? | ✓ | ✓ | 8 |
| Fabrizi et al,1997 | ✓ | ✓ | ✓ | ✓ | ✓ | ✓ | ✓ | ✓ | ✓ | 9 |
| Irshad et al,2002 | ✓ | ✓ | ✓ | ✓ | ✓ | ✓ | ? | ✓ | ✓ | 8 |
| Gessoni et al,1998 | ✓ | ✓ | ✓ | ✓ | ✓ | ✓ | ? | ✓ | ✓ | 8 |
| Stefanidis et al,2004 | ✓ | ✓ | ✓ | ✓ | ✓ | ✓ | ? | ✓ | ✓ | 8 |
| Mobaien et al,2013 | ✓ | ✓ | ✓ | × | ✓ | ✓ | ? | ✓ | ✓ | 7 |
| Yong et al,2014 | ✓ | ✓ | ✓ | ✓ | ✓ | ✓ | ? | ✓ | ✓ | 8 |
| Maylin et al,2012 | ✓ | ✓ | ✓ | ✓ | ✓ | ✓ | ? | ✓ | ✓ | 8 |
| Politou et al,2015 | ✓ | ✓ | ✓ | ✓ | ✓ | ✓ | ? | ✓ | ✓ | 8 |
| Kenfak-Foguena et al,2011 | ✓ | ✓ | ✓ | ✓ | ✓ | ✓ | ? | ✓ | ✓ | 8 |
| Keane et al,2012 | ✓ | ✓ | ✓ | ✓ | ✓ | ✓ | ? | ✓ | ✓ | 8 |
| Ng et al,2000 | ✓ | ? | ✓ | ✓ | ✓ | ✓ | ? | ✓ | ✓ | 7 |
| Feldt et al,2013 | ✓ | ✓ | ✓ | ✓ | ✓ | ✓ | ? | ✓ | ✓ | 8 |
| Ethakovic et al,2014 | ✓ | ✓ | ✓ | - | ✓ | ✓ | ? | ✓ | ✓ | 7 |
| Renou et al, 2010 | ✓ | ✓ | ✓ | ✓ | ✓ | ✓ | ? | ✓ | ✓ | 8 |
| Mateos-Lindemann et al,2014 | ✓ | ✓ | ✓ | ✓ | ✓ | ✓ | ? | ✓ | ✓ | 8 |
| Caron et al,2012 | ✓ | ✓ | ✓ | ✓ | ✓ | ✓ | ? | ✓ | ✓ | 8 |
| Riveiro-Barciela et al,2014 | ✓ | ? | ✓ | ✓ | ✓ | ✓ | ? | ✓ | ✓ | 7 |
| Sellier et al,2011 | ✓ | ✓ | ✓ | ✓ | ✓ | ✓ | ? | ✓ | ✓ | 8 |
| Hassing et al,2014 | ✓ | ✓ | ✓ | ✓ | ✓ | ✓ | ? | ✓ | ✓ | 8 |
| Nouhin et al,2015 | ✓ | ✓ | ✓ | ✓ | ✓ | ✓ | ✓ | ✓ | ✓ | 9 |
| Crum-Cianflone et al,2012 | ✓ | ✓ | ✓ | - | ✓ | ✓ | ✓ | ✓ | ✓ | 8 |
| Fainboim et al,1999 | ✓ | ✓ | ✓ | ✓ | ✓ | ✓ | ✓ | ✓ | ✓ | 9 |
| Zeng et al,2017 | ✓ | ✓ | ✓ | ✓ | ✓ | ✓ | ✓ | ✓ | ✓ | 9 |
| Shrestha et al,2017 | ✓ | ✓ | ✓ | ✓ | ✓ | ✓ | ✓ | ✓ | ✓ | 9 |
| Kaba et al,2011 | ✓ | ✓ | ✓ | ✓ | ✓ | ✓ | ✓ | ✓ | ✓ | 9 |
| Rivero-Juarez et al, 2015 | ✓ | ✓ | ✓ | ✓ | ✓ | ✓ | ✓ | ✓ | ✓ | 9 |
| Scotto et al,2015 | ✓ | ✓ | ✓ | ✓ | ✓ | ✓ | ✓ | ✓ | ✓ | 9 |
| Joulaei et al,2015 | ✓ | ✓ | ✓ | ✓ | ✓ | ✓ | ✓ | ✓ | ✓ | 9 |
| Pineda et al,2014 | ✓ | ✓ | ✓ | ✓ | ✓ | ✓ | ✓ | ✓ | ✓ | 9 |
| Sherman et al,2014 | ✓ | ✓ | ✓ | ✓ | ✓ | ✓ | ✓ | ✓ | ✓ | 9 |
| Vazquez-Moron et al,2019 | ✓ | ✓ | ✓ | ✓ | ✓ | ✓ | ✓ | ✓ | ✓ | 9 |
| Jardi et al,2012 | ✓ | ✓ | ✓ | ✓ | ✓ | ✓ | ✓ | ✓ | ✓ | 9 |
| Shimizu et al, 2016 | ✓ | ✓ | ✓ | ✓ | ✓ | ✓ | ✓ | ✓ | ✓ | 9 |

| | | | | | | | | | | |
|---|---|---|---|---|---|---|---|---|---|---|
| Zhou et al,2018 | ✓ | × | ✓ | ✓ | ✓ | ✓ | ? | ✓ | ✓ | 7 |
| Rivero-Juarez et al,2017 | ✓ | ✓ | ✓ | ✓ | ✓ | ✓ | ? | ✓ | ✓ | 8 |
| Ramezani et al,2013 | ✓ | ✓ | ✓ | ✓ | ✓ | ✓ | ✓ | ✓ | ✓ | 9 |
| Pischke et al,2010 | ✓ | ✓ | ✓ | ✓ | ✓ | ✓ | ? | ✓ | ✓ | 8 |
| Balayan et al,1997 | ✓ | ✓ | ✓ | ✓ | ✓ | ✓ | ? | ✓ | ✓ | 8 |
| Boon et al,2018 | ✓ | ? | ✓ | ✓ | ✓ | ✓ | ? | ✓ | ✓ | 7 |
| Ferreira et al,2018 | ✓ | ✓ | ✓ | ✓ | ✓ | ✓ | ? | ✓ | ✓ | 8 |
| Harritshoj et al,2018 | ✓ | ✓ | ✓ | ✓ | ✓ | ✓ | ? | ✓ | ✓ | 8 |
| Bura et al,2017 | ✓ | ✓ | ✓ | ✓ | ✓ | ✓ | ? | ✓ | ✓ | 8 |
| Abravanel et al,2017 | ✓ | ✓ | ✓ | ✓ | ✓ | ✓ | ? | ✓ | ✓ | 8 |
| Bezerra et al,2019 | ✓ | ✓ | ✓ | ✓ | ✓ | ✓ | ? | ✓ | ✓ | 8 |
| Madejon et al, 2009 | ✓ | ✓ | ✓ | ✓ | ✓ | ✓ | ? | ✓ | ✓ | 8 |
| Salvio et al,2018 | ✓ | ✓ | ✓ | ✓ | ✓ | ✓ | ✓ | ✓ | ✓ | 9 |
| Pischke et al,2015 | ✓ | ✓ | ✓ | ✓ | ✓ | ✓ | ✓ | ✓ | ✓ | 9 |
| Kuniholm et al,2016 | ✓ | ✓ | ✓ | ✓ | ✓ | ✓ | ✓ | ✓ | ✓ | 9 |
| Demi et al,2018 | ✓ | ✓ | × | ✓ | ✓ | ✓ | ✓ | ✓ | ✓ | 8 |
| Enayatollah et al,2018 | ✓ | ✓ | ✓ | ✓ | ✓ | ✓ | ✓ | ✓ | ✓ | 9 |
| Oladipo et al,2015 | ✓ | ✓ | ✓ | ✓ | ✓ | ✓ | ✓ | ✓ | ✓ | 9 |
| Kantala et al, 2009 | ✓ | ✓ | ✓ | ✓ | ✓ | ✓ | ? | ✓ | ✓ | 8 |
| Khuroo et al,1994 | ✓ | ? | ✓ | ✓ | ✓ | ✓ | ? | ✓ | ✓ | 7 |
| Yang et al, 2019 | ✓ | ✓ | ✓ | ✓ | ✓ | ✓ | ✓ | ✓ | ✓ | 9 |
| Pischke et al,2014 | ✓ | ✓ | ✓ | ✓ | ✓ | ✓ | ? | ✓ | ✓ | 8 |
| Psichogiou et al,1995 | ✓ | ✓ | ✓ | ✓ | ✓ | ✓ | ? | ✓ | ✓ | 8 |
| Siripanyaphinyo et al,2014 | ✓ | × | ✓ | ✓ | ✓ | ✓ | ? | ✓ | ✓ | 7 |
| Schulz et al,2016 | ✓ | ✓ | ✓ | × | ✓ | ✓ | ? | ✓ | ✓ | 7 |
| Ramalingam et al,2013 | ✓ | ✓ | ✓ | ✓ | ✓ | ✓ | ? | ✓ | ✓ | 8 |
| Divizia et al,1999 | ✓ | ✓ | ✓ | ✓ | ✓ | ✓ | ? | ✓ | ✓ | 8 |
| Al-Knawy et al,1997 | ✓ | ✓ | ✓ | ✓ | ✓ | ✓ | ? | ✓ | ✓ | 8 |
| Cacciola et al,2011 | ✓ | ✓ | ✓ | ✓ | ✓ | ✓ | ? | ✓ | ✓ | 8 |
| Bayram et al,2007 | ✓ | ✓ | ✓ | ✓ | ✓ | ✓ | ? | ✓ | ✓ | 8 |
| Chandra et al, 2014 | ✓ | ✓ | ✓ | ✓ | ✓ | ✓ | ? | ✓ | ✓ | 8 |
| Freitas et al,2016 | ✓ | ✓ | ✓ | ✓ | ✓ | ✓ | ? | ✓ | ✓ | 8 |
| Goumba et al,2011 | ✓ | ? | ✓ | × | ✓ | ✓ | ✓ | ✓ | ✓ | 7 |
| Gupta et al,2018 | ✓ | ✓ | ✓ | ✓ | ✓ | ✓ | ? | ✓ | ✓ | 8 |
| Hyams et al,1996 | ✓ | ✓ | ✓ | ✓ | ✓ | ✓ | ? | ✓ | ✓ | 8 |
| Coursaget et al,1998 | ✓ | ✓ | ✓ | ✓ | ✓ | ✓ | ? | ✓ | ✓ | 8 |
| Shrestha et al,2003 | ✓ | ✓ | ✓ | ✓ | ✓ | ✓ | ? | ✓ | ✓ | 8 |
| Mohammad et al,2007 | ✓ | ✓ | ✓ | ✓ | ✓ | ✓ | ? | ✓ | ✓ | 8 |
| Guilherme et al,2018 | ✓ | ✓ | ✓ | ✓ | ✓ | ✓ | ? | ✓ | ✓ | 8 |
| Perez-Gracia et al, 2004 | ✓ | ✓ | ✓ | ✓ | ✓ | ✓ | ? | ✓ | ✓ | 8 |
| J.-F. TSAI et al,1994 | ✓ | ✓ | ✓ | ✓ | ✓ | ✓ | ? | ✓ | ✓ | 8 |
| Tsatsralt-Od et al,2016 | ✓ | ✓ | ✓ | ✓ | ✓ | ✓ | ? | ✓ | ✓ | 8 |
| Chandra et al, 2012 | ✓ | ✓ | ✓ | × | ✓ | ✓ | ✓ | ✓ | ✓ | 8 |
| Fridmana et al,2016 | ✓ | ✓ | ✓ | ✓ | ✓ | ✓ | ✓ | ✓ | ✓ | 9 |

| | | | | | | | | | | |
|---|---|---|---|---|---|---|---|---|---|---|
| Albetkova et al,2007 | ✓ | ✓ | ✓ | ✓ | ✓ | ✓ | ? | ✓ | ✓ | 8 |
| Koot et al,2015 | ✓ | ? | ✓ | ✓ | ✓ | ✓ | ? | ✓ | ✓ | 7 |
| Kumar et al,2007 | ✓ | ✓ | ✓ | ✓ | ✓ | ✓ | ✓ | ✓ | ✓ | 9 |
| Joon et al,2015 | ✓ | ✓ | ✓ | ✓ | ✓ | ✓ | ? | ✓ | ✓ | 8 |
| Kantala et al,2017 | ✓ | ✓ | ✓ | ✓ | ✓ | ✓ | ? | ✓ | ✓ | 8 |
| Kang et al,2017 | ✓ | ✓ | ✓ | ✓ | ✓ | ✓ | ? | ✓ | ✓ | 8 |
| Meader et al,2010 | ✓ | ✓ | ✓ | ✓ | ✓ | ✓ | ? | ✓ | ✓ | 8 |
| Engle et al, 2002 | ✓ | ✓ | ✓ | ✓ | ✓ | ✓ | ? | ✓ | ✓ | 8 |
| Meng et al,2002 | ✓ | ✓ | ✓ | ✓ | ✓ | ✓ | ? | ✓ | ✓ | 8 |
| Zheng et al,2006 | ✓ | ✓ | ✓ | ? | ✓ | ✓ | ? | ✓ | ✓ | 7 |
| Hinjoy et al,2013 | ✓ | ✓ | ✓ | ✓ | ✓ | ✓ | ? | ✓ | ✓ | 8 |
| Utsumi et al,2011 | ✓ | ✓ | ✓ | ✓ | ✓ | ✓ | ? | ✓ | ✓ | 8 |
| Silva et al,2012 | ✓ | ✓ | ✓ | ✓ | ✓ | ✓ | ? | ✓ | ✓ | 8 |
| Traore et al,2015 | ✓ | ✓ | ✓ | ✓ | ✓ | ✓ | ? | ✓ | ✓ | 8 |
| Ivanova et al,2015 | ✓ | ? | ✓ | × | ✓ | ✓ | ✓ | ✓ | ✓ | 7 |
| Caruso et al,2017 | ✓ | ✓ | ✓ | ✓ | ✓ | ✓ | ? | ✓ | ✓ | 8 |
| Masia et al, 2009 | ✓ | ✓ | ✓ | ✓ | ✓ | ✓ | ? | ✓ | ✓ | 8 |
| Mughini-Gras et al,2017 | ✓ | ✓ | ✓ | ✓ | ✓ | ✓ | ? | ✓ | ✓ | 8 |
| Vivek et al,2011 | ✓ | ✓ | ✓ | ✓ | ✓ | ✓ | ? | ✓ | ✓ | 8 |
| Lee et al,2013 | ✓ | ✓ | ✓ | ✓ | ✓ | ✓ | ? | ✓ | ✓ | 8 |
| De Sabato et al,2017 | ✓ | ✓ | ✓ | ✓ | ✓ | ✓ | ? | ✓ | ✓ | 8 |
| Zhang et al,2017 | ✓ | ✓ | ✓ | ✓ | ✓ | ✓ | ? | ✓ | ✓ | 8 |
| Yu et al,2013 | ✓ | ✓ | ✓ | ✓ | ✓ | ✓ | ? | ✓ | ✓ | 8 |
| Chaussade et al,2013 | ✓ | ✓ | ✓ | ✓ | ✓ | ✓ | ? | ✓ | ✓ | 8 |
| Widasari 2013 | ✓ | ✓ | ✓ | ✓ | ✓ | ✓ | ? | ✓ | ✓ | 8 |
| Drobeniuc et al,2001 | ✓ | ✓ | ✓ | ✓ | ✓ | ✓ | ? | ✓ | ✓ | 8 |
| Bouwknegt et al,2008 | ✓ | ✓ | ✓ | ✓ | ✓ | ✓ | ? | ✓ | ✓ | 8 |
| Galiana et al,2008 | ✓ | ✓ | ✓ | ✓ | ✓ | ✓ | ? | ✓ | ✓ | 8 |
| Olsen et al,2006 | ✓ | ✓ | ✓ | ✓ | ✓ | ✓ | ? | ✓ | ✓ | 8 |
| Withers et al,2002 | ✓ | ✓ | ✓ | ? | ✓ | ✓ | ? | ✓ | ✓ | 7 |
| Lucarelli et al, 2016 | ✓ | ✓ | ✓ | ✓ | ✓ | ✓ | ? | ✓ | ✓ | 8 |
| Verhoef et al,2012 | ✓ | ✓ | ✓ | ✓ | ✓ | ✓ | ? | ✓ | ✓ | 8 |
| Lafeber et al, 2017 | ✓ | ✓ | ✓ | ✓ | ✓ | ✓ | ? | ✓ | ✓ | 8 |
| Love et al,2018 | ✓ | ✓ | ✓ | ✓ | ✓ | ✓ | ✓ | ✓ | ✓ | 9 |
| Lange et al,2017 | ✓ | ✓ | ✓ | ✓ | ✓ | ✓ | ✓ | ✓ | ✓ | 9 |
| Liang et al, 2014 | ✓ | ✓ | ✓ | ✓ | ✓ | ✓ | ✓ | ✓ | ✓ | 9 |
| Krumbholz et al,2012 | ✓ | ✓ | ✓ | ✓ | ✓ | ✓ | ✓ | ✓ | ✓ | 9 |
| Tritz et al,2018 | ✓ | ✓ | ✓ | ✓ | ✓ | ✓ | ? | ✓ | ✓ | 8 |
| Krumbholz et al,2014 | ✓ | ✓ | ✓ | ✓ | ✓ | ✓ | ? | ✓ | ✓ | 8 |
| Tabibi et al,2013 | ✓ | ✓ | ✓ | ✓ | ✓ | ✓ | ? | ✓ | ✓ | 8 |
| Alvarado-Esquivel et al,2014 | ✓ | ✓ | ✓ | ✓ | ✓ | ✓ | ? | ✓ | ✓ | 8 |
| Cong et al,2014 | ✓ | ? | ✓ | × | ✓ | ✓ | ? | ✓ | ✓ | 6 |
| Abebe et al,2017 | ✓ | ✓ | ✓ | ✓ | ✓ | ✓ | ? | ✓ | ✓ | 8 |

| | | | | | | | | | |
|---|---|---|---|---|---|---|---|---|---|
| Rey et al,1997 | ✓ | ✓ | ✓ | ✓ | ✓ | ✓ | ? | ✓ | ✓ | 8 |
| Martinez et al, 2014 | ✓ | ✓ | ✓ | ✓ | ✓ | ✓ | ? | ✓ | ✓ | 8 |
| Munne et al,2014 | ✓ | ✓ | ✓ | ✓ | ✓ | ✓ | ? | ✓ | ✓ | 8 |
| Moaven et al,1995 | ✓ | ✓ | ✓ | ✓ | ✓ | ✓ | ? | ✓ | ✓ | 8 |
| Lagler et al,2014 | ✓ | ✓ | ✓ | ✓ | ✓ | ✓ | ? | ✓ | ✓ | 8 |
| Fischer et al, 2015 | ✓ | ✓ | ✓ | ✓ | ✓ | ✓ | ? | ✓ | ✓ | 8 |
| Rahman et al,2014 | ✓ | ✓ | ✓ | ✓ | ✓ | ✓ | ? | ✓ | ✓ | 8 |
| Labrique et al,2009 | ✓ | ✓ | ✓ | ✓ | ✓ | ✓ | ? | ✓ | ✓ | 8 |
| Sheikh et al, 2002 | ✓ | ✓ | ✓ | ✓ | ✓ | ✓ | ? | ✓ | ✓ | 8 |
| De Paschale et al, 2016 | ✓ | ✓ | ✓ | ✓ | ✓ | ✓ | ? | ✓ | ✓ | 8 |
| Dell'Amico et al,2011 | ✓ | ? | ✓ | ✓ | ✓ | ✓ | ? | ✓ | ✓ | 7 |
| Konomi et al,1999 | ✓ | ✓ | ✓ | × | ✓ | ✓ | ? | ✓ | ✓ | 7 |
| Gandolfo et al,2003 | ✓ | ✓ | ✓ | ✓ | ✓ | ✓ | ? | ✓ | ✓ | 8 |
| Bartoloni et al,1999 | ✓ | ✓ | ✓ | ✓ | ✓ | ✓ | ? | ✓ | ✓ | 8 |
| Leon et al, 1999 | ✓ | ✓ | ✓ | ✓ | ✓ | ✓ | ? | ✓ | ✓ | 8 |
| de Paula et al,2001 | ✓ | ✓ | ✓ | ✓ | ✓ | ✓ | ? | ✓ | ✓ | 8 |
| Passos-Castilho et al,2016 | ✓ | ✓ | ✓ | ✓ | ✓ | ✓ | ? | ✓ | ✓ | 8 |
| Goncales et al,2000 | ✓ | ✓ | ✓ | ✓ | ✓ | ✓ | ? | ✓ | ✓ | 8 |
| Vitral et al,2014 | ✓ | ✓ | ✓ | ✓ | ✓ | ✓ | ✓ | ✓ | ✓ | 9 |
| Santos et al,2002 | ✓ | ✓ | ✓ | ✓ | ✓ | ✓ | ✓ | ✓ | ✓ | 9 |
| Hardtke et al,2018 | ✓ | ✓ | ✓ | ✓ | ✓ | ✓ | ✓ | ✓ | ✓ | 9 |
| Martins et al,2014 | ✓ | ✓ | ✓ | ? | ✓ | ✓ | ? | ✓ | ✓ | 7 |
| Bortoliero et al,2006 | ✓ | ✓ | ✓ | ✓ | ✓ | ✓ | ✓ | ✓ | ✓ | 9 |
| Pavel et al,2014 | ✓ | ✓ | ✓ | ✓ | ✓ | ✓ | ? | ✓ | ✓ | 8 |
| Traore et al,2012 | ✓ | ✓ | ✓ | ✓ | ✓ | ✓ | ✓ | ✓ | ✓ | 9 |
| Aubry et al,1997 | ✓ | ✓ | ✓ | ✓ | ✓ | ✓ | ? | ✓ | ✓ | 8 |
| Fearon et al, 2017 | ✓ | ✓ | ✓ | ✓ | ✓ | ✓ | ✓ | ✓ | ✓ | 9 |
| Nouhin et al,2016 | ✓ | ✓ | ✓ | ✓ | ✓ | ✓ | ✓ | ✓ | ✓ | 9 |
| Nouhin et al,2018 | ✓ | ✓ | ✓ | ? | ✓ | ✓ | ✓ | ✓ | ✓ | 8 |
| Yamada et al,2015 | ✓ | ✓ | ✓ | ✓ | ✓ | ✓ | ? | ✓ | ✓ | 8 |
| Wong et al,2004 | ✓ | ✓ | ✓ | ✓ | ✓ | ✓ | ? | ✓ | ✓ | 8 |
| Zhuang et al,2014 | ✓ | ✓ | ✓ | ✓ | ✓ | ✓ | ? | ✓ | ✓ | 9 |
| Wang et al,2017 | ✓ | ✓ | ✓ | ✓ | ✓ | ✓ | ? | ✓ | ✓ | 9 |
| Chiu et al,2013 | ✓ | ✓ | ✓ | ✓ | ✓ | ✓ | ? | ✓ | ✓ | 9 |
| Chang et al,2009 | ✓ | ? | ✓ | ✓ | ✓ | ✓ | ? | ✓ | ✓ | 7 |
| Chen et al,2019 | ✓ | ✓ | ✓ | ✓ | ✓ | ✓ | ? | ✓ | ✓ | 8 |
| Guo et al,2010 | ✓ | ✓ | ✓ | ✓ | ✓ | ✓ | ? | ✓ | ✓ | 8 |
| Yang et al,2014 | ✓ | ✓ | ✓ | ✓ | ✓ | ✓ | ? | ✓ | ✓ | 8 |
| Peng et al,1995 | ✓ | ✓ | ✓ | ✓ | ✓ | ✓ | ✓ | ✓ | ✓ | 9 |
| Jia et al,2014 | ✓ | ✓ | ✓ | ✓ | ✓ | ✓ | ✓ | ✓ | ✓ | 9 |
| Li et al,2011 | ✓ | ✓ | ✓ | ✓ | ✓ | ✓ | ✓ | ✓ | ✓ | 9 |
| Li et al,2014 | ✓ | ✓ | ✓ | ✓ | ✓ | ✓ | ? | ✓ | ✓ | 8 |
| Lee et al,1994 | ✓ | ✓ | ✓ | ✓ | ✓ | ✓ | ? | ✓ | ✓ | 8 |
| Liu et al,2019 | ✓ | ✓ | ✓ | ✓ | ✓ | ✓ | ? | ✓ | ✓ | 8 |

| | | | | | | | | | | |
|---|---|---|---|---|---|---|---|---|---|---|
| Cui et al,2016 | ✓ | ✓ | ✓ | ✓ | ✓ | ✓ | ? | ✓ | ✓ | 8 |
| Zeng et al, 2017 | ✓ | ✓ | ✓ | ✓ | ✓ | ✓ | ? | ✓ | ✓ | 8 |
| Cheng et al,2007 | ✓ | ✓ | ✓ | ✓ | ✓ | ✓ | ? | ✓ | ✓ | 8 |
| Meng et al,2015 | ✓ | ✓ | ✓ | - | ✓ | ✓ | ? | ✓ | ✓ | 7 |
| Toole et al,2006 | ✓ | ✓ | ✓ | ✓ | ✓ | ✓ | ? | ✓ | ✓ | 8 |
| Dong et al,2007 | ✓ | ✓ | ✓ | ✓ | ✓ | ✓ | ? | ✓ | ✓ | 8 |
| Geng et al,2011 | ✓ | ✓ | ✓ | ✓ | ✓ | ✓ | ? | ✓ | ✓ | 8 |
| Zhu et al,2008 | ✓ | ✓ | ✓ | ✓ | ✓ | ✓ | ✓ | ✓ | ✓ | 9 |
| Zhu et al, 2014 | ✓ | ✓ | ✓ | - | ✓ | ✓ | ✓ | ✓ | ✓ | 8 |
| Ren et al,2014 | ✓ | ✓ | ✓ | ✓ | ✓ | ✓ | ✓ | ✓ | ✓ | 9 |
| Cai et al,2017 | ✓ | ✓ | ✓ | ✓ | ✓ | ✓ | ✓ | ✓ | ✓ | 9 |
| Feng et al,2018 | ✓ | ✓ | ✓ | ✓ | ✓ | ✓ | ? | ✓ | ✓ | 8 |
| Chan et al,2017 | ✓ | ✓ | ✓ | ✓ | ✓ | ✓ | ✓ | ✓ | ✓ | 9 |
| Chen et al,2012 | ✓ | ✓ | ✓ | ✓ | ✓ | ✓ | ✓ | ✓ | ✓ | 9 |
| Ma et al,2006 | ✓ | ✓ | ✓ | ✓ | ✓ | ✓ | ? | ✓ | ✓ | 8 |
| Li et al,2006 | ✓ | ✓ | ✓ | ✓ | ✓ | ✓ | ? | ✓ | ✓ | 8 |
| Lu et al, 2009 | ✓ | ✓ | ✓ | ✓ | ✓ | ✓ | ? | ✓ | ✓ | 8 |
| Li et al,2019 | ✓ | ✓ | ✓ | ✓ | ✓ | ✓ | ✓ | ✓ | ✓ | 9 |
| Zhilian et al,2018 | ✓ | ✓ | ✓ | ✓ | ✓ | ✓ | ✓ | ✓ | ✓ | 9 |
| Huang et al, 2013 | ✓ | ✓ | ✓ | ✓ | ✓ | ✓ | ✓ | ✓ | ✓ | 9 |
| Ibarra et al,1994 | ✓ | ✓ | ✓ | ✓ | ✓ | ✓ | ✓ | ✓ | ✓ | 9 |
| Strakova et al,2014 | ✓ | ✓ | ✓ | ✓ | ✓ | ✓ | ? | ✓ | ✓ | 8 |
| Quintana et al,2005 | ✓ | ✓ | ✓ | - | ✓ | ✓ | ? | ✓ | ✓ | 7 |
| Villalba et al,2010 | ✓ | ✓ | ✓ | ✓ | ✓ | ✓ | ? | ✓ | ✓ | 8 |
| Lemos et al,2000 | ✓ | ✓ | ✓ | ✓ | ✓ | ✓ | ? | ✓ | ✓ | 8 |
| Christensen et al,2008 | ✓ | ✓ | ✓ | ✓ | ✓ | ✓ | ? | ✓ | ✓ | 8 |
| Langer et al,1997 | ✓ | ✓ | ✓ | ✓ | ✓ | ✓ | ? | ✓ | ✓ | 8 |
| Dimeglio et al, 2018 | ✓ | ? | ✓ | - | ✓ | ✓ | ? | ✓ | ✓ | 6 |
| Boutrouille et al,2007 | ✓ | ? | ✓ | ✓ | ✓ | ✓ | ? | ✓ | ✓ | 7 |
| Mansuy et al,2016 | ✓ | ✓ | ✓ | ✓ | ✓ | ✓ | ? | ✓ | ✓ | 8 |
| Mansuy et al,2015 | ✓ | ✓ | ✓ | ✓ | ✓ | ✓ | ✓ | ✓ | ✓ | 9 |
| Kaba et al,2010 | ✓ | ✓ | ✓ | ✓ | ✓ | ✓ | ✓ | ✓ | ✓ | 9 |
| Takahashi et al,2004 | ✓ | ✓ | ✓ | ✓ | ✓ | ✓ | ✓ | ✓ | ✓ | 9 |
| Takeda et al,2010 | ✓ | ✓ | ✓ | ✓ | ✓ | ✓ | ✓ | ✓ | ✓ | 9 |
| Sakata et al,2008 | ✓ | ✓ | ✓ | ✓ | ✓ | ✓ | ✓ | ✓ | ✓ | 9 |
| Toyoda et al,2008 | ✓ | ✓ | ✓ | ✓ | ✓ | ✓ | ✓ | ✓ | ✓ | 9 |
| Mitsui et al,2005 | ✓ | ✓ | ✓ | ✓ | ✓ | ✓ | ✓ | ✓ | ✓ | 9 |
| Goto et al,2006 | ✓ | ✓ | ✓ | ✓ | ✓ | ✓ | ? | ✓ | ✓ | 8 |
| Fukuda et al, 2007 | ✓ | ✓ | ✓ | ✓ | ✓ | ✓ | ? | ✓ | ✓ | 8 |
| Gambel et al,1998 | ✓ | ✓ | ✓ | ✓ | ✓ | ✓ | ? | ✓ | ✓ | 8 |
| Kamel et al,1995 | ✓ | ✓ | ✓ | ✓ | ✓ | ✓ | ? | ✓ | ✓ | 8 |
| Aboulata et al, 2005 | ✓ | ✓ | ✓ | ? | ✓ | ✓ | ? | ✓ | ✓ | 7 |
| Hasan et al,2016 | ✓ | ✓ | ✓ | ✓ | ✓ | ✓ | ? | ✓ | ✓ | 8 |
| Fix et al,2000 | ✓ | ✓ | ✓ | ✓ | ✓ | ✓ | ? | ✓ | ✓ | 8 |
| Stoszek et al,2006 | ✓ | ✓ | ✓ | ✓ | ✓ | ✓ | ? | ✓ | ✓ | 8 |
| Gad et al, 2011 | ✓ | ✓ | ✓ | ✓ | ✓ | ✓ | ? | ✓ | ✓ | 8 |

| | | | | | | | | | | |
|---|---|---|---|---|---|---|---|---|---|---|
| Wenzel et al,2014 | ✓ | ✓ | ✓ | ✓ | ✓ | ✓ | ✓ | ✓ | ✓ | 9 |
| Juhl et al,2018 | ✓ | ✓ | ✓ | ✓ | ✓ | ✓ | ✓ | ✓ | ✓ | 9 |
| Mahrt et al,2018 | ✓ | ? | ✓ | ✓ | ✓ | ✓ | ✓ | ✓ | ✓ | 8 |
| Jablonka et al,2015 | ✓ | ✓ | ✓ | ✓ | ✓ | ✓ | ✓ | ✓ | ✓ | 9 |
| Schielke et al,2015 | ✓ | ✓ | ✓ | ✓ | ✓ | ✓ | ✓ | ✓ | ✓ | 9 |
| Dremsek et al, 2012 | ✓ | ✓ | ✓ | ✓ | ✓ | ✓ | ✓ | ✓ | ✓ | 9 |
| Meldal et al,2013 | ✓ | ✓ | ✓ | ✓ | ✓ | ✓ | ✓ | ✓ | ✓ | 9 |
| Adjei et al,2009 | ✓ | ✓ | ✓ | ✓ | ✓ | ✓ | ✓ | ✓ | ✓ | 9 |
| Talarmin et al,1997 | ✓ | ✓ | ✓ | ✓ | ✓ | ✓ | ✓ | ✓ | ✓ | 9 |
| Tripathy et al,2019 | ✓ | ✓ | ✓ | ? | ✓ | ✓ | ✓ | ✓ | ✓ | 8 |
| Vivek et al,2010 | ✓ | ✓ | ✓ | ✓ | ✓ | ✓ | ✓ | ✓ | ✓ | 9 |
| Kotwal et al,2014 | ✓ | ✓ | ✓ | ✓ | ✓ | ✓ | ✓ | ✓ | ✓ | 9 |
| Katiyar et al, 2018 | ✓ | ✓ | ✓ | ✓ | ✓ | ✓ | ✓ | ✓ | ✓ | 9 |
| Pathak et al,2017 | ✓ | ✓ | ✓ | ✓ | ✓ | ✓ | ✓ | ✓ | ✓ | 9 |
| Mathur et al,2001 | ✓ | ? | ✓ | ✓ | ✓ | ✓ | ✓ | ✓ | ✓ | 8 |
| Achwan et al,2007 | ✓ | ✓ | ✓ | ✓ | ✓ | ✓ | ✓ | ✓ | ✓ | 9 |
| Ehteram et al,2013 | ✓ | ✓ | ✓ | ✓ | ✓ | ✓ | ✓ | ✓ | ✓ | 9 |
| Farshadpour et al,2015 | ✓ | ✓ | ✓ | × | ✓ | ✓ | ✓ | ✓ | ✓ | 8 |
| Assarehzadegan et al,2008 | ✓ | ✓ | ✓ | ✓ | ✓ | ✓ | ? | ✓ | ✓ | 8 |
| Mohebbi et al,2012 | ✓ | ? | ✓ | ✓ | ✓ | ✓ | ? | ✓ | ✓ | 7 |
| Sotoodeh et al,2013 | ✓ | ✓ | ✓ | ✓ | ✓ | ✓ | ? | ✓ | ✓ | 8 |
| Ataei et al, 2009 | ✓ | ✓ | ✓ | ✓ | ✓ | ✓ | ? | ✓ | ✓ | 8 |
| Hesamizadeh et al,2016 | ✓ | ✓ | ✓ | ✓ | ✓ | ✓ | ? | ✓ | ✓ | 8 |
| Sepanlou et al,2010 | ✓ | ? | ✓ | ✓ | ✓ | ✓ | ? | ✓ | ✓ | 7 |
| Raoofi et al, 2012 | ✓ | ? | ✓ | ✓ | ✓ | ✓ | ? | ✓ | ✓ | 7 |
| Jahanbakhsh et al,2017 | ✓ | ? | ✓ | ✓ | × | ✓ | ? | ✓ | ✓ | 6 |
| Saffar et al,2009 | ✓ | ✓ | ✓ | ✓ | ✓ | ✓ | ? | ✓ | ✓ | 8 |
| Hickey et al,2016 | ✓ | ✓ | ✓ | ✓ | ✓ | ✓ | ✓ | ✓ | ✓ | 9 |
| O'Riordan et al,2016 | ✓ | ✓ | ✓ | ✓ | ✓ | ✓ | ? | ✓ | ✓ | 8 |
| Karetnyi et al, 1995 | ✓ | ✓ | ✓ | ✓ | ✓ | ✓ | ? | ✓ | ✓ | 8 |
| Lachish et al, 2013 | ✓ | ✓ | ✓ | ✓ | ✓ | ✓ | ? | ✓ | ✓ | 8 |
| Mor et al, 2015 | ✓ | ✓ | ✓ | ✓ | ✓ | ✓ | ? | ✓ | ✓ | 8 |
| Spada et al,2018 | ✓ | ✓ | ✓ | ✓ | ✓ | ✓ | ? | ✓ | ✓ | 8 |
| Gessoni et al, 1996 | ✓ | ✓ | ✓ | ✓ | ✓ | ✓ | ? | ✓ | ✓ | 8 |
| Puttini et al,2015 | ✓ | ✓ | ✓ | ✓ | ✓ | ✓ | ? | ✓ | ✓ | 8 |
| Pavia et al,1998 | ✓ | ✓ | ✓ | ✓ | ✓ | ✓ | ? | ✓ | ✓ | 8 |
| Zuin et al, 2017 | ✓ | ✓ | ✓ | ✓ | ✓ | ✓ | ? | ✓ | ✓ | 8 |
| Scotto et al, 2012 | ✓ | ✓ | ✓ | ✓ | ✓ | ✓ | ✓ | ✓ | ✓ | 9 |
| Marcantonio et al,2019 | ✓ | ✓ | ✓ | ✓ | ? | ✓ | ✓ | ✓ | ✓ | 8 |
| Lanini et al,2015 | ✓ | ✓ | ✓ | ✓ | ✓ | ✓ | ? | ✓ | ✓ | 8 |
| Rapicetta et al,1999 | ✓ | ✓ | ✓ | ✓ | ✓ | ✓ | ? | ✓ | ✓ | 8 |
| Alecci et al,1997 | ✓ | ✓ | ✓ | ✓ | ✓ | ✓ | ? | ✓ | ✓ | 8 |
| Obaidat et al,2018 | ✓ | ✓ | ✓ | ✓ | ✓ | ✓ | ? | ✓ | ✓ | 8 |
| Cainelli et al, 2018 | ✓ | ✓ | ✓ | ✓ | ✓ | ✓ | ? | ✓ | ✓ | 8 |
| Ahn et al,2005 | ✓ | ✓ | ✓ | ✓ | ✓ | ✓ | ? | ✓ | ✓ | 8 |

| | | | | | | | | | |
|---|---|---|---|---|---|---|---|---|---|
| Yoon et al,2014 | ✓ | ✓ | ✓ | ✓ | ✓ | ✓ | ? | ✓ | ✓ | 8 |
| Yun et al, 2011 | ✓ | ✓ | ✓ | ✓ | ✓ | ✓ | ? | ✓ | ✓ | 8 |
| Chironna et al,2001 | ✓ | ✓ | ✓ | ✓ | ✓ | ✓ | ? | ✓ | ✓ | 8 |
| Khounvisith et al,2018 | ✓ | ✓ | ✓ | ✓ | ✓ | ✓ | ✓ | ✓ | ✓ | 9 |
| Alvarez-Munoz et al,1999 | ✓ | ✓ | ✓ | ✓ | ✓ | ✓ | ✓ | ✓ | ✓ | 9 |
| Baptista-Gonzalez et al,2017 | ✓ | ✓ | ✓ | ✓ | ✓ | ✓ | ✓ | ✓ | ✓ | 9 |
| Alvarado-Esquivel et al,2015 | ✓ | ✓ | ✓ | ✓ | ✓ | ✓ | ✓ | ✓ | ✓ | 9 |
| Davaalkham et al,2009 | ✓ | ✓ | ✓ | ✓ | ✓ | ✓ | ✓ | ✓ | ✓ | 9 |
| Tsatsralt-Od et al,2007 | ✓ | ✓ | ✓ | - | ✓ | ✓ | ✓ | ✓ | ✓ | 9 |
| Terzic et al, 2015 | ✓ | ✓ | ✓ | ✓ | ✓ | ✓ | ✓ | ✓ | ✓ | 9 |
| Taha et al,2015 | ✓ | ✓ | ✓ | ✓ | ✓ | ✓ | ✓ | ✓ | ✓ | 9 |
| Halliday et al,2014 | ✓ | ✓ | ✓ | ✓ | ✓ | ✓ | ? | ✓ | ✓ | 8 |
| Abe et al, 2006 | ✓ | ✓ | ✓ | ✓ | ✓ | ✓ | ? | ✓ | ✓ | 8 |
| Baylis et al,2012 | ✓ | ✓ | ✓ | ✓ | ✓ | ✓ | ? | ✓ | ✓ | 8 |
| Minuk et al, 2007 | ✓ | ✓ | ✓ | ✓ | ✓ | ✓ | ? | ✓ | ✓ | 8 |
| Izopet et al,2015 | ✓ | ✓ | ✓ | ✓ | ✓ | ✓ | ? | ✓ | ✓ | 8 |
| Schreuder et al, 2016 | ✓ | ✓ | ✓ | ✓ | ✓ | ✓ | ? | ✓ | ✓ | 8 |
| Clayson et al,1997 | ✓ | ✓ | ✓ | ✓ | ✓ | ✓ | ? | ✓ | ✓ | 8 |
| Shrestha et al,2016 | ✓ | ✓ | ✓ | ✓ | ✓ | ✓ | ? | ✓ | ✓ | 8 |
| Mooij et al,2018 | ✓ | ✓ | ✓ | ✓ | ✓ | ✓ | ? | ✓ | ✓ | 8 |
| Slot et al, 2013 | ✓ | ✓ | ✓ | ✓ | ✓ | ✓ | ✓ | ✓ | ✓ | 9 |
| Sadik et al,2016 | ✓ | ✓ | ✓ | × | ✓ | ✓ | ✓ | ✓ | ✓ | 8 |
| Hogema et al,2016 | ✓ | ✓ | ✓ | ✓ | ✓ | ✓ | ✓ | ✓ | ✓ | 9 |
| Alberts et al, 2018 | ✓ | ? | ✓ | ✓ | ✓ | ✓ | ✓ | ✓ | ✓ | 8 |
| Hewitt et al,2018 | ✓ | ✓ | ✓ | ✓ | ✓ | ✓ | ✓ | ✓ | ✓ | 9 |
| Ekanem et al,2015 | ✓ | ✓ | ✓ | ✓ | ✓ | ✓ | ? | ✓ | ✓ | 8 |
| O. A Adesina et al,2009 | ✓ | ? | ✓ | ✓ | ✓ | ✓ | ? | ✓ | ✓ | 7 |
| Perez 1996 | ✓ | ✓ | ✓ | ✓ | ✓ | ✓ | ? | ✓ | ✓ | 8 |
| Grabarczyk et al,2018 | ✓ | ✓ | ✓ | ✓ | ✓ | ✓ | ? | ✓ | ✓ | 8 |
| Korzeniewski et al,2018 | ✓ | ✓ | ✓ | ✓ | ✓ | ✓ | ? | ✓ | ✓ | 8 |
| Sadkowska-Todys et al,2015 | ✓ | ✓ | ✓ | ✓ | ✓ | ✓ | ? | ✓ | ✓ | 8 |
| Bura et al,2018 | ✓ | ✓ | ✓ | ✓ | ✓ | ✓ | ? | ✓ | ✓ | 8 |
| Nascimento et al,2018 | ✓ | ✓ | ✓ | ✓ | ✓ | ✓ | ? | ✓ | ✓ | 8 |
| Teixeira et al,2017 | ✓ | ✓ | ✓ | ✓ | ✓ | ✓ | ? | ✓ | ✓ | 8 |
| Oliveira et al,2017 | ✓ | ✓ | ✓ | ✓ | ✓ | ✓ | ? | ✓ | ✓ | 8 |
| Nasrallah et al, 2017 | ✓ | ✓ | ✓ | ✓ | ✓ | ✓ | ? | ✓ | ✓ | 8 |
| Abe et al,2004 | ✓ | ✓ | ✓ | ✓ | ✓ | ✓ | ? | ✓ | ✓ | 8 |
| Anita et al,2014 | ✓ | ✓ | ✓ | ✓ | ✓ | ✓ | ? | ✓ | ✓ | 8 |
| Arif et al,1994 | ✓ | ✓ | ✓ | ✓ | ✓ | ✓ | ? | ✓ | ✓ | 8 |
| Johargy et al, 2013 | ✓ | ? | ✓ | ✓ | ✓ | ✓ | ? | ✓ | ✓ | 7 |
| Abdelaal et al,1998 | ✓ | ✓ | ✓ | ✓ | ✓ | ✓ | ? | ✓ | ✓ | 8 |
| Petrovic et al,2014 | ✓ | ✓ | ✓ | ✓ | ✓ | ✓ | ? | ✓ | ✓ | 8 |

| | | | | | | | | | | |
|---|---|---|---|---|---|---|---|---|---|---|
| Chow et al,1997 | ✓ | ✓ | ✓ | ✓ | ✓ | ✓ | ? | ✓ | ✓ | 8 |
| Grabow et al, 1994 | ✓ | ✓ | ✓ | ✓ | ✓ | ✓ | ? | ✓ | ✓ | 8 |
| Madden et al,2016 | ✓ | ✓ | ✓ | ✓ | ✓ | ✓ | ? | ✓ | ✓ | 8 |
| Lopes et al,2017 | ✓ | ✓ | ✓ | ✓ | ✓ | ✓ | ? | ✓ | ✓ | 8 |
| Azman et al,2017 | ✓ | ✓ | ✓ | - | ✓ | ✓ | ? | ✓ | ✓ | 7 |
| Fogeda et al,2012 | ✓ | ✓ | ✓ | ✓ | ✓ | ✓ | ? | ✓ | ✓ | 8 |
| Bernal 1995 | ✓ | ✓ | ✓ | ✓ | ✓ | ✓ | ? | ✓ | ✓ | 8 |
| Mateos et al,1998 | ✓ | ✓ | ✓ | ✓ | ✓ | ✓ | ? | ✓ | ✓ | 8 |
| Buti et al,2013 2008 | ✓ | ✓ | ✓ | ? | ✓ | ✓ | ? | ✓ | ✓ | 7 |
| Buti et al,2006 | ✓ | ✓ | ✓ | ✓ | ✓ | ✓ | ? | ✓ | ✓ | 8 |
| Sauleda et al, 2015 | ✓ | ✓ | ✓ | ✓ | ✓ | ✓ | ✓ | ✓ | ✓ | 9 |
| Kaufmann et al,2011 | ✓ | ✓ | ✓ | ✓ | ✓ | ✓ | ✓ | ✓ | ✓ | 9 |
| Lavanchy et al,1994 | ✓ | ✓ | ✓ | - | ✓ | ✓ | ✓ | ✓ | ✓ | 8 |
| Jeggli et al,2004 | ✓ | ✓ | ✓ | ✓ | ✓ | ✓ | ? | ✓ | ✓ | 8 |
| Niederhauser et al,2018 | ✓ | ? | ✓ | ✓ | ✓ | ✓ | ✓ | ✓ | ✓ | 8 |
| Poovorawan et al,1996 | ✓ | ✓ | ✓ | ✓ | ✓ | ✓ | ✓ | ✓ | ✓ | 9 |
| Intharasongkroh et al,2019 | ✓ | ✓ | ✓ | ✓ | ✓ | ✓ | ✓ | ✓ | ✓ | 9 |
| Pilakasiri et al,2009 | ✓ | ✓ | ✓ | ✓ | ✓ | ✓ | ✓ | ✓ | ✓ | 9 |
| Mohanavalli et al,2003 | ✓ | ✓ | ✓ | ✓ | ✓ | ✓ | ✓ | ✓ | ✓ | 9 |
| Houcine et al,2012 | ✓ | ? | ✓ | ✓ | ✓ | ✓ | ? | ✓ | ✓ | 7 |
| Colak et al,2002 | ✓ | ✓ | ✓ | ✓ | ✓ | ✓ | ? | ✓ | ✓ | 8 |
| Bayhan et al,2016 | ✓ | ✓ | ✓ | ✓ | ✓ | ✓ | ? | ✓ | ✓ | 8 |
| Atabek et al,2004 | ✓ | ✓ | ✓ | ✓ | ✓ | ✓ | ? | ✓ | ✓ | 8 |
| Kaya et al,2008 | ✓ | ✓ | ✓ | ✓ | ✓ | ✓ | ? | ✓ | ✓ | 8 |
| Sencan et al,2004 | ✓ | ✓ | ✓ | ✓ | ✓ | ✓ | ? | ✓ | ✓ | 8 |
| Sidal et al,2001 | ✓ | ✓ | ✓ | ✓ | ✓ | ✓ | ? | ✓ | ✓ | 8 |
| Dilek et al,2003 | ✓ | ✓ | ✓ | ✓ | ✓ | ✓ | ? | ✓ | ✓ | 8 |
| Nural et al,2013 | ✓ | ✓ | ✓ | ✓ | ✓ | ✓ | ? | ✓ | ✓ | 8 |
| Oncu et al,2006 | ✓ | ✓ | ✓ | ✓ | ✓ | ✓ | ✓ | ✓ | ✓ | 9 |
| Cevrioglu et al,2004 | ✓ | ✓ | ✓ | ✓ | ✓ | ✓ | ✓ | ✓ | ✓ | 9 |
| Miller et al,1998 | ✓ | ✓ | ✓ | ✓ | ✓ | ✓ | ✓ | ✓ | ✓ | 9 |
| Cangin et al, 2019 | ✓ | ✓ | ✓ | ✓ | ✓ | ✓ | ✓ | ✓ | ✓ | 9 |
| Karetnyi et al,1999 | ✓ | ✓ | ✓ | × | ✓ | ✓ | ✓ | ✓ | ✓ | 8 |
| Kuniholm et al,2009 | ✓ | ✓ | ✓ | ✓ | ✓ | ✓ | ✓ | ✓ | ✓ | 9 |
| Eick et al,2010 | ✓ | ✓ | ✓ | ✓ | ✓ | ✓ | ✓ | ✓ | ✓ | 9 |
| Ditah et al,2014 | ✓ | ✓ | ✓ | ✓ | ✓ | ✓ | ✓ | ✓ | ✓ | 9 |
| Zhang et al,2015 | ✓ | ✓ | ✓ | ✓ | ✓ | ✓ | ✓ | ✓ | ✓ | 9 |
| Kyvernitakis 2015 | ✓ | ✓ | ✓ | ✓ | ✓ | ✓ | ? | ✓ | ✓ | 8 |
| Xu et al,2013 | ✓ | ✓ | ✓ | ✓ | ✓ | ✓ | ? | ✓ | ✓ | 8 |
| Stramer et al,2016 | ✓ | ✓ | ✓ | ✓ | ✓ | ✓ | ? | ✓ | ✓ | 8 |
| Teshale et al, 2015 | ✓ | ✓ | ✓ | ✓ | ✓ | ✓ | ? | ✓ | ✓ | 8 |
| Cleland et al, 2013 | ✓ | ✓ | ✓ | ✓ | ✓ | ✓ | ? | ✓ | ✓ | 8 |
| Thom et al,2018 | ✓ | ✓ | ✓ | ✓ | ✓ | ✓ | ? | ✓ | ✓ | 8 |
| Dalton et al,2017 | ✓ | ✓ | ✓ | ✓ | ✓ | ✓ | ? | ✓ | ✓ | 8 |
| Harvala et al,2019 | ✓ | ✓ | ✓ | ✓ | ✓ | ✓ | ? | ✓ | ✓ | 8 |
| Ijaz et al,2009 | ✓ | ✓ | ✓ | ✓ | ✓ | ✓ | ? | ✓ | ✓ | 8 |

| | | | | | | | | | | |
|---|---|---|---|---|---|---|---|---|---|---|
| Pujol et al,1994 | ✓ | ✓ | ✓ | ✓ | ✓ | ✓ | ✓ | ✓ | ✓ | 9 |
| Corwin et al,1996 | ✓ | ✓ | ✓ | ✓ | ✓ | ✓ | ✓ | ✓ | ✓ | 9 |
| Hau et al,1999 | ✓ | ✓ | ✓ | ✓ | ✓ | ✓ | ✓ | ✓ | ✓ | 9 |
| Bawazir et al, 2010 | ✓ | ✓ | ✓ | ✓ | ✓ | ✓ | ? | ✓ | ✓ | 8 |
| Maitrey et al,2014 | ✓ | ✓ | ✓ | ✓ | ✓ | ✓ | ? | ✓ | ✓ | 8 |
| Gupta et al,2015 | ✓ | ✓ | ✓ | ✓ | ✓ | ✓ | ? | ✓ | ✓ | 8 |
| Sadaf et al,2015 | ✓ | ✓ | ✓ | ? | - | ✓ | ? | ✓ | ✓ | 6 |
| Ifeorah et al, 2017 | ✓ | ✓ | ✓ | ✓ | ✓ | ✓ | ? | ✓ | ✓ | 8 |
| Adeola et al,2018 | ✓ | ✓ | ✓ | ✓ | ✓ | ✓ | ? | ✓ | ✓ | 8 |
| Kumar et al,2001 | ✓ | ✓ | ✓ | ✓ | ✓ | ✓ | ? | ✓ | ✓ | 8 |
| Hoad et al,2017 | ✓ | ✓ | ✓ | ✓ | ✓ | ✓ | ? | ✓ | ✓ | 8 |
| Harritshoj et al, 2016 | ✓ | ✓ | ✓ | ✓ | ✓ | ✓ | ? | ✓ | ✓ | 8 |
| Vollmer et al,2012 | ✓ | ✓ | ✓ | ✓ | ✓ | ✓ | ✓ | ✓ | ✓ | 9 |
| Arankalle et al,2002 | ✓ | ✓ | ✓ | ✓ | ✓ | ✓ | ✓ | ✓ | ✓ | 9 |
| La Fauci et al,2017 | ✓ | ✓ | ✓ | ✓ | ✓ | ✓ | ✓ | ✓ | ✓ | 9 |
| Minagi et al,2016 | ✓ | ✓ | ✓ | ✓ | ✓ | ✓ | ✓ | ✓ | ✓ | 9 |
| Riezebos-Brilman et al,2013 | ✓ | ✓ | ✓ | ✓ | ✓ | ✓ | ✓ | ✓ | ✓ | 9 |
| Rivero-Juarez et al,2019 | ✓ | ✓ | ✓ | ✓ | ✓ | ✓ | ✓ | ✓ | ✓ | 9 |
| Hewitt et al,2014 | ✓ | ✓ | ✓ | ✓ | ✓ | ✓ | ✓ | ✓ | ✓ | 9 |

**Meaning of symbol** ✓ Yes × No ? Not clear -Not applicable

**Table S2. Distribution of HEV genotypes 1-4 in different countries.**

| Name | Country | HEV-1 | HEV-2 | HEV-3 | HEV-4 | Subgroup |
|---|---|---|---|---|---|---|
| Fischer et al, 2015 | Austria | | | 7 | | General population |
| Dell'Amico et al, 2011 | Bolivia | | | 4 | | General population |
| Passos et al, 2013 | Brazil | | | 3 | | Transplant |
| Nouhin et al, 2016 | Cambodia | | | 1 | | General population |
| Yamada et al, 2015 | Cambodia | | | 1 | 1 | General population |
| Halac et al, 2012 | Canada | | | 5 | | Transplant |
| Zhu et al, 2008 | China | | | | 15 | General population |
| Ren et al, 2014 | China | | | | 3 | General population |
| Ma et al, 2010 | China | | | | 23 | General population |
| Li et al, 2006 | China | 1 | | | 23 | General population |
| Li et al, 2019 | China | | | | 1 | General population |
| Rui et al, 2018 | China | | | | 4 | General population |
| Yu et al, 2009 | China | | | | 30 | General population |
| Chen et al, 2019 | China | | | | 4 | General population |
| Yang et al, 2014 | China | | | | 12 | General population |
| Li et al, 2011 | China | | | | 1 | General population |
| Li et al, 2014 | China | | | | 10 | General population |
| Dong et al, 2007 | China | | | | 25 | General population |
| Zhu et al, 2014 | China | 5 | | | 72 | General population |
| Zheng et al, 2006 | China | 4 | | 27 | | Occupational |
| Vallalba et al, 2012 | Cuba | | | 3 | | Occupational |
| Harritshoj et al, 2016 | Denmark | | | 2 | | General population |
| Kantala et al, 2009 | Finland | 5 | | | | Hepatitis |
| Mansuy et al, 2004 | France | | | 16 | | General population |
| Kaba et al, 2010 | France | | | 1 | | General population |
| Kaba et al, 2011 | France | | | 1 | | HIV |
| Moal et al, 2012 | France | | | 17 | | Transplant |
| Dremsek et al, 2012 | Germany | | | 4 | | Occupational population |
| Schielke et al, 2015 | Germany | | | 1 | | Occupational population |
| Hoerning et al, 2012 | Germany | | | 3 | | Transplant |
| Pischke et al, 2010 | Germany | | | 3 | | Transplant |
| Voller et al, 2012 | Germany | | | 13 | | General population |
| Tripathy et al, 2019 | India | 2 | | | | General population |
| Pathak et al, 2017 | India | 22 | | | | General population |
| Persa et al, 2016 | Iran | 7 | | | | General population |
| O'Riordan et al,2016 | Ireland | | | 3 | | General population |
| Lucarelli et al, 2016 | Italy | | | 3 | | General population |
| Marcantonio et al, 2019 | Italy | | | 1 | | General population |
| Inagaki et al, 2015 | Japan | | | 2 | | Transplant |
| Fukuda et al, 2007 | Japan | | | 8 | 3 | General population |
| Sakata et al, 2008 | Japan | | | 19 | 4 | General population |
| Takahashi et al, 2010 | Japan | | | 3 | | General population |
| Minagi et al, 2016 | Japan | | | 36 | | General population |
| Ahn et al, 2005 | Korea | | | 14 | | General population |
| Tsatsralt et al, 2016 | Mongolia | | | | 12 | Hepatitis |
| Abe et al, 2006 | Nepal | 3 | | | | General population |
| Shrestha et al, 2003 | Nepal | 48 | | | | Hepatitis |
| Slot et al, 2013 | Netherlands | | | 16 | | General population |
| Hogema et al, 2016 | Netherlands | | | 17 | | General population |
| Suzan et al, 2012 | Netherlands | | | 11 | | Transplant |
| Brilman et al, 2012 | Netherlands | | | 10 | | Transplant |
| Koot et al, 2015 | Netherlands | 4 | | 50 | | Hepatitis |
| Grabarczyk et al, 2018 | Poland | | | 3 | | General population |

| | | | | |
|---|---|---|---|---|
| Anita et al, 2014 | Romania | | 6 | General population |
| Sauleda et al, 2014 | Spain | | 3 | General population |
| Jardi et al, 2012 | Spain | | 3 | HIV |
| Foguena et al, 2011 | Switzerland | | 1 | HIV |
| Intharasongkroh et al, 2018 | Thailand | | 3 | General population |
| Siripanyaphinyo et al, 2014 | Thailand | | 5 | Hepatitis |
| Albetkova et al, 2007 | Turkmenistan | 25 | | Hepatitis |
| Harvala et al, 2018 | UK | | 479 | General population |
| Hewitt et al, 2014 | UK | | 79 | General population |
| Cleland et al, 2013 | UK | | 1 | General population |
| Thom et al, 2018 | UK | | 10 | General population |
| Ramalingam et al, 2013 | UK | | 7 | Hepatitis |
| Mark et al, 2015 | USA | | 3 | HIV |

**Table S3. Sensitivity analysis in seroepidemiology of anti-HEV IgG in general population**

| Study | Prevalence | Lower | Upper |
|---|---|---|---|
| Chang et al, 2009 | 0.1242 | 0.1037 | 0.1464 |
| Geng et al, 2011 | 0.1242 | 0.1037 | 0.1464 |
| Yuan et al, 2017 | 0.1242 | 0.1036 | 0.1463 |
| Maria et al, 2006 | 0.1247 | 0.1041 | 0.1468 |
| Chen et al, 2019 | 0.1242 | 0.1037 | 0.1464 |
| Edward et al, 1997 | 0.1229 | 0.1025 | 0.1449 |
| Corwin et al, 1996 | 0.1244 | 0.1039 | 0.1466 |
| Hassan et al, 2013 | 0.1244 | 0.1039 | 0.1465 |
| Fatemeh et al, 2014 | 0.1236 | 0.1031 | 0.1456 |
| Guo et al, 2009 | 0.1239 | 0.1030 | 0.1464 |
| Halliday et al, 2014 | 0.1245 | 0.1039 | 0.1467 |
| Ahn et al, 2005 | 0.1244 | 0.1039 | 0.1466 |
| Azarkar et al, 2017 | 0.1241 | 0.1036 | 0.1462 |
| Boutrouille et al, 2007 | 0.1249 | 0.1043 | 0.1471 |
| Cainelli et al, 2018 | 0.1248 | 0.1042 | 0.1469 |
| Christensen et al,2018 | 0.1239 | 0.1034 | 0.1460 |
| Abe et al, 2006 | 0.1239 | 0.1033 | 0.1460 |
| Baylis et al, 2012 | 0.1254 | 0.1096 | 0.1420 |
| Assarehzadegan et al, 2007 | 0.1245 | 0.1039 | 0.1466 |
| Dell’Amico et al, 2011 | 0.1246 | 0.1040 | 0.1467 |
| Yoon et al, 2014 | 0.1247 | 0.1042 | 0.1469 |
| Takahashi et al, 2004 | 0.1245 | 0.1040 | 0.1467 |
| Takahashi et al, 2010 | 0.1248 | 0.1041 | 0.1471 |
| O’Riordan et al, 2016 | 0.1248 | 0.1042 | 0.1469 |
| Spada et al, 2018 | 0.1246 | 0.1040 | 0.1469 |
| Nasrallah et al, 2017 | 0.1242 | 0.1036 | 0.1464 |
| Moaven et al, 1995 | 0.1249 | 0.1043 | 0.1471 |
| Zhuang et al, 2014 | 0.1241 | 0.1036 | 0.1463 |
| Nascimento et al, 2016 | 0.1243 | 0.1038 | 0.1465 |
| Takeda et al, 2010 | 0.1249 | 0.1043 | 0.1471 |
| Minuk et al, 2007 | 0.1249 | 0.1043 | 0.1471 |
| Nouhin et al, 2016 | 0.1240 | 0.1035 | 0.1461 |
| Verhoef et al, 2011 | 0.1250 | 0.1044 | 0.1472 |
| Castilho et al, 2017 | 0.1246 | 0.1040 | 0.1467 |
| Mooij et al, 2018 | 0.1239 | 0.1034 | 0.1461 |
| Lafeber et al, 2017 | 0.1240 | 0.1035 | 0.1462 |
| Yun et al, 2011 | 0.1246 | 0.1040 | 0.1467 |
| Olcay et al, 2003 | 0.1247 | 0.1041 | 0.1469 |
| Rapicetta et al, 1999 | 0.1250 | 0.1044 | 0.1472 |
| Nouhin et al, 2018 | 0.1237 | 0.1032 | 0.1458 |
| Slot et al, 2013 | 0.1240 | 0.1035 | 0.1462 |
| Straková et al, 2016 | 0.1248 | 0.1042 | 0.1469 |
| Wong et al, 2004 | 0.1243 | 0.1037 | 0.1464 |
| Meldal et al, 2013 | 0.1248 | 0.1042 | 0.1470 |
| Wenzel et al, 2014 | 0.1237 | 0.1032 | 0.1457 |
| Petrovi et al, 2014 | 0.1244 | 0.1038 | 0.1465 |
| Wang et al, 2017 | 0.1240 | 0.1034 | 0.1462 |
| Tripathy et al, 2018 | 0.1243 | 0.1037 | 0.1465 |
| Quintana et al, 2005 | 0.1248 | 0.1042 | 0.1469 |
| Taremi et al, 2007 | 0.1247 | 0.1041 | 0.1468 |
| Castilho et al, 2016 | 0.1246 | 0.1040 | 0.1467 |
| Vivek et al, 2010 | 0.1244 | 0.1038 | 0.1466 |
| Zhu et al, 2008 | 0.1240 | 0.1035 | 0.1462 |
| Sakata et al, 2008 | 0.1249 | 0.1043 | 0.1471 |
| Sadik et al, 2016 | 0.1238 | 0.1033 | 0.1458 |
| Mohebbi et al, 2012 | 0.1246 | 0.1040 | 0.1467 |
| Yang et al, 2014 | 0.1241 | 0.1036 | 0.1463 |
| Zhu et al, 2014 | 0.1238 | 0.1052 | 0.1436 |
| Thom et al, 2017 | 0.1248 | 0.1042 | 0.1470 |
| Mor et al, 2015 | 0.1245 | 0.1040 | 0.1467 |
| Villalba et al, 2014 | 0.1246 | 0.1040 | 0.1467 |
| Toyoda et al, 2008 | 0.1246 | 0.1040 | 0.1467 |
| Shrestha et al, 2016 | 0.1237 | 0.1032 | 0.1458 |
| Mohammad et al, 2017 | 0.1239 | 0.1034 | 0.1460 |
| Yamada et al, 2015 | 0.1243 | 0.1037 | 0.1464 |
| Vitral et al, 2014 | 0.1245 | 0.1039 | 0.1466 |
| Peng et al, 1995 | 0.1247 | 0.1041 | 0.1468 |
| Talarmin et al, 1997 | 0.1247 | 0.1041 | 0.1469 |
| Sauleda et al, 2014 | 0.1242 | 0.1036 | 0.1465 |
| Ren et al, 2013 | 0.1240 | 0.1034 | 0.1463 |
| Santos et al, 2002 | 0.1249 | 0.1044 | 0.1471 |
| Miller et al, 1998 | 0.1252 | 0.1046 | 0.1474 |
| Parsa et al, 2016 | 0.1247 | 0.1041 | 0.1468 |

| | | | |
|---|---|---|---|
| Pittaras et al, 2014 | 0.1246 | 0.1040 | 0.1467 |
| Schreuder et al, 2016 | 0.1248 | 0.1042 | 0.1470 |
| Jahromi et al, 2013 | 0.1248 | 0.1042 | 0.1469 |
| Niederhauser et al, 2017 | 0.1242 | 0.1036 | 0.1464 |
| Teoharov et al, 2014 | 0.1246 | 0.1040 | 0.1467 |
| Bortoliero et al, 2006 | 0.1250 | 0.1044 | 0.1471 |
| Hewitt et al, 2018 | 0.1246 | 0.1040 | 0.1467 |
| Alberts et al, 2018 | 0.1249 | 0.1043 | 0.1471 |
| Grabarczyk et al, 2018 | 0.1236 | 0.1031 | 0.1457 |
| Gessoni et al, 1996 | 0.1249 | 0.1043 | 0.1471 |
| Fukuda et al,  2007 | 0.1248 | 0.1042 | 0.1469 |
| Fogeda et al, 2012 | 0.1251 | 0.1045 | 0.1472 |
| Fischer et al, 2014 | 0.1244 | 0.1039 | 0.1466 |
| Feng et al, 2018 | 0.1231 | 0.1027 | 0.1451 |
| Fearon et al, 2017 | 0.1247 | 0.1041 | 0.1469 |
| Harvala et al, 2018 | 0.1246 | 0.1040 | 0.1468 |
| Cleland et al, 2013 | 0.1248 | 0.1042 | 0.1470 |
| Cangin et al, 2018 | 0.1248 | 0.1042 | 0.1470 |
| Ditah et al, 2014 | 0.1248 | 0.1041 | 0.1470 |
| Paula et al, 2001 | 0.1248 | 0.1043 | 0.1470 |
| Goncales et al, 2000 | 0.1248 | 0.1042 | 0.1469 |
| Cai et al, 2016 | 0.1234 | 0.1029 | 0.1454 |
| Chiu et al, 2013 | 0.1240 | 0.1035 | 0.1461 |
| Dimeglio et al, 2018 | 0.1247 | 0.1041 | 0.1468 |
| Ding et al, 2003 | 0.1249 | 0.1043 | 0.1471 |
| Chan et al, 2017 | 0.1239 | 0.1034 | 0.1460 |
| Arif et al, 1994 | 0.1245 | 0.1039 | 0.1467 |
| Abdelaal et al, 1995 | 0.1243 | 0.1038 | 0.1465 |
| Amarapurkar et al, 2008 | 0.1238 | 0.1033 | 0.1459 |
| Anita et al, 2014 | 0.1244 | 0.1038 | 0.1465 |
| Grabow et al, 1994 | 0.1250 | 0.1044 | 0.1471 |
| Hardtke et al,  2018 | 0.1242 | 0.1036 | 0.1463 |
| Ataei et al, 2007 | 0.1249 | 0.1043 | 0.1470 |
| Hesamizadeh et al, 2015 | 0.1246 | 0.1040 | 0.1468 |
| Hogema et al, 2016 | 0.1241 | 0.1036 | 0.1462 |
| Hickey et al, 2016 | 0.1246 | 0.1041 | 0.1468 |
| Houcine et al, 2012 | 0.1248 | 0.1042 | 0.1469 |
| Jahromi et al, 2013 | 0.1248 | 0.1042 | 0.1469 |
| Sepanlou et al, 2010 | 0.1247 | 0.1041 | 0.1468 |
| Mitsui et al, 2005 | 0.1250 | 0.1044 | 0.1471 |
| Adesina et al, 2009 | 0.1244 | 0.1039 | 0.1466 |
| Bernal et al, 1995 | 0.1250 | 0.1044 | 0.1471 |
| Pujol et al, 1994 | 0.1249 | 0.1043 | 0.1470 |
| Ricco et al, 2016 | 0.1248 | 0.1042 | 0.1470 |
| Puttini et al, 2015 | 0.1246 | 0.1040 | 0.1467 |
| Pavia et al, 1998 | 0.1251 | 0.1045 | 0.1473 |
| Zuin et al, 2016 | 0.1245 | 0.1039 | 0.1467 |
| Scotto et al, 2012 | 0.1250 | 0.1044 | 0.1472 |
| Raoofi et al, 2012 | 0.1247 | 0.1041 | 0.1468 |
| Rahman et al, 2014 | 0.1240 | 0.1034 | 0.1461 |
| Poovorawan et al, 1996 | 0.1247 | 0.1042 | 0.1469 |
| Sabato et al, 2017 | 0.1250 | 0.1044 | 0.1472 |
| Chen et al, 2012 | 0.1242 | 0.1035 | 0.1466 |
| Madden et al, 2016 | 0.1240 | 0.1035 | 0.1461 |
| Love et al, 2018 | 0.1250 | 0.1044 | 0.1471 |
| Lopes et al, 2017 | 0.1241 | 0.1035 | 0.1462 |
| Mansuy et al, 2016 | 0.1242 | 0.1035 | 0.1464 |
| Karetny et al, 1995 | 0.1249 | 0.1043 | 0.1471 |
| Lucarelli et al, 2016 | 0.1235 | 0.1030 | 0.1456 |
| Lange et al, 2016 | 0.1244 | 0.1039 | 0.1466 |
| Kotwal et al, 2014 | 0.1243 | 0.1037 | 0.1465 |
| Liang et al, 2014 | 0.1237 | 0.1032 | 0.1458 |
| Mateos et al, 1998 | 0.1249 | 0.1043 | 0.1470 |
| Marcantonio et al, 2018 | 0.1248 | 0.1042 | 0.1469 |
| Karetnyi et al, 1999 | 0.1248 | 0.1042 | 0.1469 |
| Lanini et al, 2015 | 0.1248 | 0.1042 | 0.1469 |
| Johargy et al, 2013 | 0.1243 | [0.1037 | 0.1464 |
| Jia et al, 2014 | 0.1241 | 0.1034 | 0.1465 |
| Ma et al, 2010 | 0.1243 | 0.1037 | 0.1464 |
| Kaufmann et al, 2011 | 0.1248 | 0.1042 | 0.1469 |
| Mansuy et al, 2004 | 0.1245 | 0.1040 | 0.1467 |
| Mansuy et al, 2015 | 0.1237 | 0.1032 | 0.1458 |
| Izopet et al, 2015 | 0.1237 | 0.1032 | 0.1457 |
| Li et al, 2011 | 0.1237 | 0.1032 | 0.1458 |
| Konomi et al, 1999 | 0.1244 | 0.1038 | 0.1465 |
| Li et al, 2014 | 0.1245 | 0.1039 | 0.1466 |
| Juhl et al, 2017 | 0.1244 | 0.1038 | 0.1465 |
| Katiyar et al, 2018 | 0.1232 | 0.1028 | 0.1453 |
| Intharasongkroh et al, 2019 | 0.1253 | 0.1047 | 0.1475 |

| | | | |
|---|---|---|---|
| Juhl et al, 2014 | 0.1247 | 0.1041 | 0.1469 |
| Kamel et al, 1995 | 0.1243 | 0.1038 | 0.1465 |
| Nagler et al, 2014 | 0.1244 | 0.1038 | 0.1465 |
| Langer et al, 1997 | 0.1251 | 0.1045 | 0.1473 |
| Lee et al, 1993 | 0.1248 | 0.1042 | 0.1470 |
| Lavanchy et al, 1994 | 0.1248 | 0.1042 | 0.1470 |
| Ibarra et al, 1994 | 0.1243 | 0.1038 | 0.1464 |
| Liu et al, 2019 | 0.1239 | 0.1033 | 0.1461 |
| Mahrt et al, 2018 | 0.1242 | 0.1036 | 0.1463 |
| Kuniholm et al, 2009 | 0.1242 | 0.1034 | 0.1466 |
| Krumbholz et al, 2011 | 0.1244 | 0.1038 | 0.1465 |
| Khounvisith et al, 2018 | 0.1243 | 0.1037 | 0.1464 |
| Krumbholz et al, 2014 | 0.1245 | 0.1039 | 0.1466 |
| Jeggli et al, 2015 | 0.1249 | 0.1043 | 0.1470 |
| Kantala et al, 2015 | 0.1246 | 0.1040 | 0.1467 |
| Kang et al, 2017 | 0.1242 | 0.1037 | 0.1463 |
| Korzeniewski et al, 2018 | 0.1248 | 0.1042 | 0.1469 |
| Li et al, 2006 | 0.1236 | 0.1031 | 0.1457 |
| Labrique et al, 2009 | 0.1242 | 0.1036 | 0.1463 |
| Jablonka et al, 2017 | 0.1242 | 0.1037 | 0.1463 |
| Matins et al, 2014 | 0.1248 | 0.1042 | 0.1469 |
| Jahanbakhsh et al, 2017 | 0.1241 | 0.1036 | 0.1462 |
| Kaba et al, 2010 | 0.1245 | 0.1039 | 0.1466 |
| Chironna et al, 1999 | 0.1249 | 0.1043 | 0.1470 |
| Rapicetta et al,1999 | 0.1245 | 0.1039 | 0.1466 |
| Azman et al, 2017 | 0.1229 | 0.1025 | 0.1449 |
| Lachish et al, 2013 | 0.1252 | 0.1046 | 0.1473 |
| Esquivel et al, 2014 | 0.1238 | 0.1033 | 0.1459 |
| Cui et al, 2016 | 0.1242 | 0.1036 | 0.1463 |
| Schielke et al, 2015 | 0.1242 | 0.1037 | 0.1463 |
| Nübling et al, 2002 | 0.1248 | 0.1043 | 0.1470 |
| Todys et al, 2015 | 0.1242 | 0.1037 | 0.1464 |
| Meader et al, 2008 | 0.1249 | 0.1044 | 0.1471 |
| Tabili et al, 2013 | 0.1242 | 0.1036 | 0.1463 |
| Pilakasiri et al, 2009 | 0.1245 | 0.1039 | 0.1466 |
| Ghorbani et al, 2007 | 0.1251 | 0.1045 | 0.1472 |
| Bura et al, 2018 | 0.1250 | 0.1044 | 0.1472 |
| Dremsek et al, 2012 | 0.1244 | 0.1038 | 0.1465 |
| Eick et al, 2010 | 0.1251 | 0.1045 | 0.1472 |
| Gambel et al, 1998 | 0.1242 | 0.1037 | 0.1463 |
| Alecci et al, 1997 | 0.1251 | 0.1045 | 0.1472 |
| Chaussade et al, 2013 | 0.1241 | 0.1035 | 0.1462 |
| Whiters et al, 2002 | 0.1249 | 0.1044 | 0.1471 |
| Zeng et al, 2014 | 0.1248 | 0.1042 | 0.1469 |
| Yu et al, 2009 | 0.1242 | 0.1036 | 0.1464 |
| Teixeira et al, 2017 | 0.1244 | 0.1039 | 0.1466 |
| Widasari et al, 2013 | 0.1247 | 0.1042 | 0.1469 |
| Olsen et al, 2006 | 0.1246 | 0.1040 | 0.1467 |
| Zhang et al, 2017 | 0.1237 | 0.1032 | 0.1458 |
| Bouwknegt et al, 2008 | 0.1250 | 0.1044 | 0.1472 |
| Drobeniuc et al, 2001 | 0.1241 | 0.1036 | 0.1462 |
| Galiana et al, 2008 | 0.1248 | 0.1042 | 0.1470 |
| Dalton et al, 2017 | 0.1250 | 0.1044 | 0.1471 |
| Cheng et al, 2007 | 0.1244 | 0.1038 | 0.1465 |
| Pathak et al, 2018 | 0.1241 | 0.1036 | 0.1462 |
| Sheikh et al, 2002 | 0.1233 | 0.1028 | 0.1453 |
| Chow et al, 1996 | 0.1244 | 0.1039 | 0.1466 |
| Colak et al, 2002 | 0.1251 | 0.1045 | 0.1473 |
| Cong et al, 2014 | 0.1241 | 0.1036 | 0.1463 |
| Terzic et al, 2009 | 0.1247 | 0.1042 | 0.1469 |
| Bayhan et al, 2016 | 0.1248 | 0.1042 | 0.1470 |
| Abe et al, 2004 | 0.1243 | 0.1037 | 0.1464 |
| Aboulata et al, 2005 | 0.1241 | 0.1036 | 0.1462 |
| Achwan et al, 2007 | 0.1247 | 0.1042 | 0.1469 |
| Atabek et al, 2004 | 0.1247 | 0.1042 | 0.1469 |
| Buti et al, 2008 | 0.1248 | 0.1042 | 0.1470 |
| Cevahir et al, 2013 | 0.1245 | 0.1039 | 0.1466 |
| Davaalkham et al, 2009 | 0.1251 | 0.1045 | 0.1473 |
| Ekanem et al, 2015 | 0.1247 | 0.1041 | 0.1468 |
| Gandolfo et al, 2003 | 0.1251 | 0.1045 | 0.1473 |
| Goto et al, 2006 | 0.1249 | 0.1043 | 0.1471 |
| Hasan et al, 2016 | 0.1241 | 0.1035 | 0.1462 |
| Kaya et al, 2007 | 0.1252 | 0.1046 | 0.1473 |
| Krumbholz et al, 2014 | 0.1251 | 0.1045 | 0.1472 |
| Lin et al, 2004 | 0.1249 | 0.1043 | 0.1471 |
| Mathur et al, 2001 | 0.1241 | 0.1035 | 0.1462 |
| Meng et al, 2015 | 0.1245 | 0.1039 | 0.1467 |
| Mohanavalli et al, 2002 | 0.1246 | 0.1040 | 0.1468 |
| Oliveira et al, 2017 | 0.1251 | 0.1045 | 0.1472 |

| | | | |
|---|---|---|---|
| Sencan et al, 2003 | 0.1247 | 0.1041 | 0.1468 |
| Shamsizadeh et al, 2009 | 0.1246 | 0.1041 | 0.1468 |
| Sidal et al, 2001 | 0.1250 | 0.1044 | 0.1471 |
| Bira et al, 2007 | 0.1251 | 0.1045 | 0.1473 |
| Hau et al, 1999 | 0.1249 | 0.1043 | 0.1471 |
| Toole et al, 2006 | 0.1239 | 0.1034 | 0.1460 |
| Saffar et al, 2009 | 0.1250 | 0.1044 | 0.1471 |
| Bawazir et al, 2010 | 0.1245 | 0.1040 | 0.1467 |
| Dong et al, 2007 | 0.1243 | 0.1036 | 0.1466 |
| Lu et al, 2009 | 0.1243 | 0.1037 | 0.1465 |
| Fix et al, 2000 | 0.1237 | 0.1031 | 0.1458 |
| Ijaz et al, 2009 | 0.1244 | 0.1039 | 0.1466 |
| Rey et al, 1997 | 0.1250 | 0.1044 | 0.1472 |
| Munoz et al, 1999 | 0.1245 | 0.1039 | 0.1467 |
| Héctor et al, 2017 | 0.1251 | 0.1045 | 0.1473 |
| Li et al, 2019 | 0.1244 | 0.1038 | 0.1465 |
| Lee et al, 2013 | 0.1250 | 0.1044 | 0.1471 |
| Serkan et al, 2005 | 0.1247 | 0.1041 | 0.1468 |
| Christophe et al, 2004 | 0.1247 | 0.1041 | 0.1468 |
| Dorcas et al, 2018 | 0.1245 | 0.1039 | 0.1466 |
| Rui et al, 2018 | 0.1242 | 0.1037 | 0.1463 |
| Maria et al, 2010 | 0.1249 | 0.1043 | 0.1470 |
| Zakieh et al, 2013 | 0.1249 | 0.1043 | 0.1470 |
| Sonia et al, 2006 | 0.1226 | 0.1023 | 0.1444 |
| Niguse et al, 2018 | 0.1237 | 0.1032 | 0.1457 |
| Caron et al, 2008 | 0.1244 | 0.1039 | 0.1466 |
| Caron et al, 2012 | 0.1247 | 0.1041 | 0.1469 |
| Sandro et al, 2017 | 0.1247 | 0.1041 | 0.1468 |
| Cosme et al, 2014 | 0.1248 | 0.1042 | 0.1469 |
| Cevrioglu et al, 2004 | 0.1245 | 0.1039 | 0.1466 |
| Farshadpour et al, 2018 | 0.1247 | 0.1041 | 0.1469 |
| Gad et al, 2011 | 0.1233 | 0.1028 | 0.1454 |
| Adjei et al, 2009 | 0.1246 | 0.1040 | 0.1467 |
| Huang et al, 2013 | 0.1246 | 0.1040 | 0.1467 |
| Paschale et al, 2016 | 0.1242 | 0.1036 | 0.1463 |
| Alizadeh et al, 2016 | 0.1249 | 0.1043 | 0.1471 |
| Abebe et al, 2017 | 0.1239 | 0.1034 | 0.1460 |
| Traore et al, 2012 | 0.1239 | 0.1034 | 0.1460 |
| Aubry et al, 1997 | 0.1244 | 0.1039 | 0.1465 |
| Gambel et al, 1998 | 0.1245 | 0.1039 | 0.1466 |
| Zhang et al, 2015 | 0.1242 | 0.1036 | 0.1464 |
| Kyvernitakis et al, 2015 | 0.1245 | 0.1040 | 0.1467 |
| Tashale et al, 2015 | 0.1247 | 0.1040 | 0.1469 |
| Xu et al,2013 | 0.1243 | 0.1037 | 0.1464 |
| Stramer et al, 2015 | 0.1244 | 0.1039 | 0.1466 |
| Meng et al, 2001 | 0.1243 | 0.1037 | 0.1464 |
| Esquivel et al, 2015 | 0.1247 | 0.1041 | 0.1468 |
| Esquivel et al, 2015 | 0.1248 | 0.1042 | 0.1469 |
| Perez et al, 1996 | 0.1248 | 0.1042 | 0.1470 |
| Wassaf et al,2014 | 0.1248 | 0.1042 | 0.1470 |
| Munne et al, 2014 | 0.1244 | 0.1038 | 0.1465 |
| Bartoloni et al, 1999 | 0.1247 | 0.1041 | 0.1468 |
| Leon et al, 1999 | 0.1242 | 0.1037 | 0.1463 |
| Trinta et al,2001 | 0.1251 | 0.1045 | 0.1473 |
| Lemos et al, 2000 | 0.1250 | 0.1044 | 0.1472 |

**Table S4. Manufactures of ELISA assays used in general population.**

| Study | year | manufactures |
|---|---|---|
| Chang et al | 2009 | Wantai |
| Geng et al | 2011 | wantai |
| Yuan et al | 2017 | Wantai |
| Maria et al | 2006 | Biokit |
| Chen et al | 2019 | Wantai |
| Edward et al | 1997 | Diagnostic Biotechnology |
| Corwin et al | 1996 | Abbott Laboratories |
| Hassan et al | 2013 | Dia.Pro |
| Fatemeh et al | 2014 | Dia.Pro |
| Guo et al | 2009 | Wantai |
| Halliday et al | 2014 | Wantai |
| Ahn et al | 2005 | Genelabs Diagnostics |
| Azarkar et al | 2017 | Delavara Company |
| Boutrouille et al | 2007 | Genelabs Diagnostics |
| Cainelli et al | 2018 | Dia.Pro |
| Christensen et al | 2018 | Unknown |
| Abe et al | 2006 | In-house assay |
| Baylis et al | 2012 | Wantai |
| Assarehzadegan et al | 2007 | Biokit |
| Dell’Amico et al | 2011 | Dia.Pro |
| Yoon et al | 2014 | Wantai |
| Takahashi et al | 2004 | In-house assay |
| Takahashi et al | 2010 | In-house assay |
| O’Riordan et al | 2016 | Wantai |
| Spada et al | 2018 | Wantai |
| Nasrallah et al | 2017 | Wantai |
| Moaven et al | 1995 | Genelabs Diagnostics |
| Zhuang et al | 2014 | Pierce |
| Nascimento et al | 2016 | Mikrogen |
| Takeda et al | 2010 | Cosmic corporation |
| Minuk et al | 2007 | Genelabs Diagnostics |
| Nouhin et al | 2016 | Wantai |
| Verhoef et al | 2011 | MP Diagnostics |
| Castilho et al | 2017 | Wantai |
| Mooij et al | 2018 | Wantai |
| Lafeber et al | 2017 | Wantai |
| Yun et al | 2011 | Dia.Pro |
| Olcay et al | 2003 | Genelabs Diagnostics |
| Rapicetta et al | 1999 | Abbott Laboratories |
| Nouhin et al | 2018 | Wantai |
| Slot et al | 2013 | Wantai |
| Straková et al | 2016 | Dia.Pro |
| Wong et al | 2004 | GeneLabs Diagnostics |
| Meldal et al | 2013 | Wantai |
| Wenzel et al | 2014 | Wantai |
| Petrovi et al | 2014 | In-house assay |
| Wang et al | 2017 | Wantai |
| Tripathy et al | 2018 | Genelabs Diagnostic/Wantai |
| Quintana et al | 2005 | Unknown |
| Taremi et al | 2007 | Dia.Pro |
| Castilho et al | 2016 | Wantai |
| Vivek et al | 2010 | MP Bio |
| Zhu et al | 2008 | Wantai |
| Sakata et al | 2008 | Cosmic Corporation |
| Sadik et al | 2016 | Wantai |
| Mohebbi et al | 2012 | Dia.Pro |
| Yang et al | 2014 | Wantai |
| Zhu et al | 2014 | Wantai |
| Thom et al | 2017 | Wantai |
| Mor et al | 2015 | Diagnostic Systems |
| Villalba et al | 2014 | Cavendish |
| Toyoda et al | 2008 | In-house assay |
| Shrestha et al | 2016 | Wantai |
| Mohammad et al | 2017 | Fortress Diagnostics |
| Yamada et al | 2015 | Institute of Immunology Company |
| Vitral et al | 2014 | Mikrogen |
| Peng et al | 1995 | Diagnostic Biotechnolgy |
| Talarmin et al | 1997 | Abbott Laboratories |
| Sauleda et al | 2014 | Wantai |
| Ren et al | 2013 | Diagnostic Biotechnology |
| Santos et al | 2002 | Abbott Laboratories |
| Miller et al | 1998 | Abbott Laboratories |

| | | |
|---|---|---|
| Parsa et al | 2016 | Dia.Pro |
| Pittaras et al | 2014 | Adaltis |
| Schreuder et al | 2016 | Wantai |
| Jahromi et al | 2013 | Unknown |
| Niederhauser et al | 2017 | Wantai |
| Teoharov et al | 2014 | Dia.Pro |
| Bortoliero et al | 2006 | Abbott Laboratories |
| Hewitt et al | 2018 | Wantai |
| Alberts et al | 2018 | Wantai |
| Grabarczyk et al | 2018 | Wantai |
| Gessoni et al | 1996 | Abbott Laboratories |
| Fukuda et al | 2007 | Unknown |
| Fogeda et al | 2012 | Mikrogen GmbH |
| Fischer et al | 2014 | Wantai |
| Feng et al | 2018 | Wantai |
| Fearon et al | 2017 | Wantai |
| Harvala et al | 2018 | Wantai |
| Cleland et al | 2013 | Wantai |
| Cangin et al | 2018 | Soronno |
| Ditah et al | 2014 | Soronno |
| Paula et al | 2001 | Organon Teknika |
| Goncales et al | 2000 | Abbott Laboratories |
| Cai et al | 2016 | Wantai |
| Chiu et al | 2013 | Biotec Laboratories |
| Dimeglio et al | 2018 | Wantai |
| Ding et al | 2003 | In-house assay |
| Chan et al | 2017 | Unknown |
| Arif et al | 1994 | Abbott Laboratories |
| Abdelaal et al | 1995 | Unknown |
| Amarapurkar et al | 2008 | Unknown |
| Anita et al | 2014 | MP Biomedicals |
| Grabow et al | 1994 | Unknown |
| Hardtke et al | 2018 | Wantai |
| Ataei et al | 2007 | Dia.Pro |
| Hesamizadeh et al | 2015 | Dia.Pro |
| Hogema et al | 2016 | Wantai |
| Hickey et al | 2016 | Wantai |
| Houcine et al | 2012 | MP Diagnostics |
| Jahromi et al | 2013 | Dia.Pro |
| Sepanlou et al | 2010 | Dia.Pro |
| Mitsui et al | 2005 | Genelabs Diagnostics |
| Adesina et al | 2009 | Dia.Pro |
| Bernal et al | 1995 | Abbott Laboratories |
| Pujol et al | 1994 | Abbott Laboratories |
| Ricco et al | 2016 | Wantai/ Dia.Pro |
| Puttini et al | 2015 | Adaltis |
| Pavia et al | 1998 | International Immunodiagnostics |
| Zuin et al | 2016 | Dia.Pro |
| Scotto et al | 2012 | Dia.Pro |
| Raoofi et al | 2012 | Unknown |
| Rahman et al | 2014 | Institute of Immunology |
| Poovorawan et al | 1996 | Genelabs Diagnostics |
| Sabato et al | 2017 | Bio-Chain Institute |
| Chen et al | 2012 | In-house assay |
| Madden et al | 2016 | Wantai |
| Love et al | 2018 | Wantai/Dia.Pro |
| Lopes et al | 2017 | Fortress Diagnostics |
| Mansuy et al | 2016 | Wantai |
| Karetny et al | 1995 | Unknown |
| Lucarelli et al | 2016 | Wantai |
| Lange et al | 2016 | Wantai |
| Kotwal et al | 2014 | Unknown |
| Liang et al | 2014 | Wantai |
| Mateos et al | 1998 | Abbott Laboratories |
| Marcantonio et al | 2018 | Wantai |
| Karetnyi et al | 1999 | In-house assay |
| Lanini et al | 2015 | Dia.Pro |
| Johargy et al | 2013 | Bioelisa |
| Jia et al | 2014 | MP Diagnostics |
| Ma et al | 2010 | Wantai |
| Kaufmann et al | 2011 | MP Biomedicals |
| Mansuy et al | 2004 | Abbott Laboratories |
| Mansuy et al | 2015 | Wantai |
| Izopet et al | 2015 | Wantai |
| Li et al | 2011 | Wantai |
| Konomi et al | 1999 | In-house assay |
| Li et al | 2014 | Wantai |
| Juhl et al | 2017 | Wantai |

| | | |
|---|---|---|
| Katiyar et al | 2018 | Wantai |
| Intharasongkroh et al | 2019 | Euroimmun/Wantai |
| Juhl et al | 2014 | Wantai |
| Kamel et al | 1995 | Abbott Laboratories |
| Nagler et al | 2014 | Fortress Diagnostics |
| Langer et al | 1997 | Abbott Laboratories |
| Lee et al | 1993 | Genelabs Diagnostics |
| Lavanchy et al | 1994 | Abbott Laboratories |
| Ibarra et al | 1994 | Abbott Laboratories |
| Liu et al | 2019 | Unknown |
| Mahrt et al | 2018 | Wantai |
| Kuniholm et al | 2009 | In-house assay |
| Krumbholz et al | 2011 | Mikrogen GmbH |
| Khounvisith et al | 2018 | Euroimmun |
| Krumbholz et al | 2014 | Mikrogen GmbH |
| Jeggli et al | 2015 | Abbott Laboratories |
| Kantala et al | 2015 | Mikrogen GmbH |
| Kang et al | 2017 | Wantai |
| Korzeniewski et al | 2018 | Mikrogen |
| Li et al | 2006 | Wantai |
| Labrique et al | 2009 | In-house assay |
| Jablonka et al | 2017 | Wantai |
| Matins et al | 2014 | Mikrogen GmbH |
| Jahanbakhsh et al | 2017 | Dia.Pro |
| Kaba et al | 2010 | Adaltis |
| Chironna et al | 1999 | Nuclear Laser Medicine |
| Rapicetta et al | 1999 | DSI |
| Azman et al | 2017 | Wantai |
| Lachish et al | 2013 | Abbott Laboratories |
| Esquivel et al | 2014 | Dia.Pro |
| Cui et al | 2016 | Wantai |
| Schielke et al | 2015 | Mikrogen GmbH |
| Nübling et al | 2002 | Unknown |
| Todys et al | 2015 | Microgen |
| Meader et al | 2008 | MP Biomedical |
| Tabili et al | 2013 | Wantai |
| Pilakasiri et al | 2009 | In-house assay |
| Ghorbani et al | 2007 | Dia.Pro |
| Bura et al | 2018 | Euroimmun |
| Dremsek et al | 2012 | In-house assay |
| Eick et al | 2010 | WRAIR |
| Gambel et al | 1998 | Genelabs Diagnostics |
| Alecci et al | 1997 | Abbott Laboratories |
| Chaussade et al | 2013 | MP Biomedicals |
| Whiters et al | 2002 | Genelabs Diagnostics |
| Zeng et al | 2014 | Wantai |
| Yu et al | 2009 | Wantai |
| Teixeira et al | 2017 | Mikrogen |
| Widasari et al | 2013 | MP Biomedicals |
| Olsen et al | 2006 | Abbott Laboratories |
| Zhang et al | 2017 | Wantai |
| Bouwknegt et al | 2008 | Abbott/Genelabs Diagnostics |
| Drobeniuc et al | 2001 | Unknown |
| Galiana et al | 2008 | Mikrogen |
| Dalton et al | 2017 | Wantai |
| Cheng et al | 2007 | Genelabs Diagnostics |
| Pathak et al | 2018 | MP Diagnostics |
| Sheikh et al | 2002 | In-house assay |
| Chow et al | 1996 | Genelabs Diagnostics |
| Colak et al | 2002 | Genelabs Diagnostics |
| Cong et al | 2014 | Wantai |
| Terzic et al | 2009 | Dia.Pro |
| Bayhan et al | 2016 | Dia.Pro |
| Abe et al | 2004 | In-house assay |
| Aboulata et al | 2004 | Unknown |
| Achwan et al | 2007 | In-house assay |
| Atabek et al | 2004 | Unknown |
| Buti et al | 2008 | Biokit |
| Cevahir et al | 2013 | BLK diagnostics |
| Davaalkham et al | 2009 | In-house assay |
| Ekanem et al | 2015 | Springfield Township |
| Gandolfo et al | 2003 | Abbott Laboratories |
| Goto et al | 2006 | Cosmic Corporation |
| Hasan et al | 2016 | Adaltis/MP Diagnostics |
| Kaya et al | 2007 | Genelabs Diagnostic |
| Krumbholz et al | 2014 | Mikrogen |
| Lin et al | 2004 | Abbott Laboratories |
| Mathur et al | 2001 | unknown |

| | | |
|---|---|---|
| Meng et al | 2015 | Wantai |
| Mohanavalli et al | 2002 | Millipore |
| Oliveira et al | 2017 | Mikrogen |
| Sencan et al | 2003 | Giuliana Diagnostica |
| Shamsizadeh et al | 2009 | Biokit |
| Sidal et al | 2001 | Unknown |
| Bira et al | 2007 | In-house assay |
| Hau et al | 1999 | Abbott Laboratories |
| Toole et al | 2006 | Wantai |
| Saffar et al | 2009 | Dia.Pro |
| Bawazir et al | 2010 | MP Diagnostic |
| Dong et al | 2007 | In-house assay |
| Lu et al | 2009 | Wantai |
| Fix et al | 2000 | Abbott Laboratories |
| Ijaz et al | 2009 | Fortress Diagnostics |
| Rey et al | 1997 | Abbott Laboratories |
| Munoz et al | 1999 | Abbott Laboratories |
| Héctor et al | 2017 | Euroimmun Medizinische |
| Li et al | 2019 | Wantai |
| Lee et al | 2013 | Wantai |
| Serkan et al | 2005 | Global Diagnostics |
| Christophe et al | 2004 | Wantai |
| Dorcas et al | 2018 | Innovita |
| Rui et al | 2018 | Wantai |
| Maria et al | 2010 | Dia.Pro |
| Zakieh et al | 2013 | Dia.Pro |
| Sonia et al | 2006 | NIH in-house EIA |
| Niguse et al | 2018 | Wantai |
| Caron et al | 2008 | Gebelabs Diagnostic |
| Caron et al | 2012 | Gebelabs Diagnostic |
| Sandro et al | 2017 | Dia.Pro |
| Cosme et al | 2014 | Dia.Pro |
| Cevrioglu et al | 2004 | Virotech GmbH |
| Farshadpour et al | 2018 | Dia.Pro |
| Gad et al | 2011 | Gebelabs Diagnostic |
| Adjei et al | 2009 | International Immunodiagnostics |
| Huang et al | 2013 | Unknown |
| Paschale et al | 2016 | Dia.Pro |
| Alizadeh et al | 2016 | Dia.Pro |
| Abebe et al | 2017 | Wantai |
| Traore et al | 2012 | Dia.Pro |
| Aubry et al | 1997 | Unknown |
| Gambel et al | 1998 | Unknown |
| Zhang et al | 2015 | Unknown |
| Kyvernitakis et al | 2015 | Mikrogen |
| Tashale et al | 2015 | DSI |
| Xu et al | 2013 | Wantai |
| Stramer et al | 2015 | Unknown |
| Meng et al | 2001 | In-house assay |
| Esquivel et al | 2015 | Dia.Pro |
| Esquivel et al | 2015 | Dia.Pro |
| Perez et al | 1996 | Abbott Laboratories |
| Wassaf et al | 2014 | Dia.Pro |
| Munne et al | 2014 | Dia.Pro |
| Bartoloni et al | 1999 | Abbott Laboratories |
| Leon et al | 1999 | Abbott Laboratories |
| Trinta et al | 2001 | Abbott Laboratories |
| Lemos et al | 2000 | Unknown |

**Table S5. HEV prevalence among occupational cohort and special cohort.**

| | | Occupational cohort | | | | | Special Cohort | | | | | | | | | | | | | | | | | | | |
|---|---|---|---|---|---|---|---|---|---|---|---|---|---|---|---|---|---|---|---|---|---|---|---|---|---|---|
| | | Anti-HEV IgG | | | | | HIV | | | | | Hemodialysis | | | | | Transplant | | | | | Hepatitis | | | | |
| Continent | Country | No· of studies | Events | Tested (n) | Prevalence (%) | 95% CI | No. of studies | Events | Tested (n) | Prevalence (%) | 95% CI | No. of studies | Events | Tested (n) | Prevalence (%) | 95% CI | No. of studies | Events | Tested (n) | Prevalence (%) | 95% CI | No. of studies | Events | Tested (n) | Prevalence (%) | 95% CI |
| Asia (22) | China | 7 | 1211 | 3064 | 41.93 | 29.97; 54.40 | 2 | 594 | 1409 | 41.98 | 37.15; 46.89 | 2 | 220 | 618 | 37.22 | 25.01; 50.31 | | | | | | 1 | 63 | 1751 | 3.60 | 2.78; 4.52 |
| | Thailand | 1 | 39 | 171 | 22.81 | 16.85; 29.38 | | | | | | | | | | | | | | | | 1 | 212 | 614 | 34.53 | 30.82; 38.33 |
| | Israel | | | | | | | | | | | | | | | | | | | | | | | | | |
| | Saudi Arabia | | | | | | | | | | | 1 | 6 | 83 | 7.23 | 2.69; 13.74 | | | | | | 1 | 12 | 132 | 9.09 | 4.80; 14.57 |
| | Japan | | | | | | | | | | | 3 | 124 | 776 | 17.91 | 8.89; 29.22 | 2 | 62 | 2077 | 3.02 | 2.19; 3.97 | | | | | |
| | Jordan | | | | | | | | | | | | | | | | | | | | | | | | | |
| | Kazakhstan | | | | | | | | | | | | | | | | | | | | | | | | | |
| | Korea | | | | | | 1 | 99 | 208 | 47.60 | 40.85; 54.38 | | | | | | | | | | | | | | | |
| | Vietnam | | | | | | | | | | | | | | | | | | | | | | | | | |
| | Yemen | | | | | | | | | | | | | | | | | | | | | | | | | |
| | Bangladesh | | | | | | | | | | | | | | | | | | | | | | | | | |
| | India | 1 | 32 | 34 | 94.12 | 83.89; 99.41 | | | | | | 1 | 22 | 50 | 44.00 | 30.65; 57.81 | 1 | 52 | 205 | 25.37 | 19.66; 31.53 | 2 | 154 | 512 | 27.63 | 6.63; 56.09 |
| | Indonesia | 2 | 38 | 289 | 14.80 | 0.0607; 26.50 | | | | | | | | | | | | | | | | | | | | |
| | Mongolia | | | | | | | | | | | | | | | | | | | | | | | | | |
| | Iran | 1 | 1 | 28 | 3.57 | 0.00; 13.44 | 3 | 62 | 331 | 19.43 | 8.31; 33.81 | 5 | 162 | 924 | 16.60 | 7.85; 27.80 | 1 | 28 | 91 | 30.77 | 21.76; 40.59 | 1 | 55 | 200 | 27.50 | 21.55; 33.88 |
| | Cambodia | | | | | | 1 | 247 | 825 | 29.94 | 26.86; 33.11 | | | | | | | | | | | | | | | |
| | Singapore | | | | | | | | | | | | | | | | | | | | | | | | | |
| | Laos | 1 | 57 | 139 | 41.01 | 32.99; 49.27 | | | | | | | | | | | | | | | | | | | | |
| | Nepal | | | | | | 1 | 181 | 459 | 39.43 | 35.01; 43.94 | | | | | | | | | | | 2 | 160 | 364 | 48.46 | 0.94; 98.38 |
| | Malaysia | | | | | | 1 | 15 | 145 | 10.34 | 5.93; 15.80 | | | | | | | | | | | | | | | |
| | Qatar | | | | | | | | | | | | | | | | | | | | | | | | | |
| | United Arab Emirates | | | | | | | | | | | | | | | | | | | | | | | | | |
| Africa (17) | Nigeria | | | | | | | | | | | | | | | | | | | | | | | | | |
| | Burkina Faso | 1 | 76 | 100 | 76 | 67.19; 83.82 | | | | | | | | | | | | | | | | | | | | |
| | Burundi | | | | | | | | | | | | | | | | | | | | | | | | | |
| | Djibouti | | | | | | | | | | | | | | | | | | | | | 1 | 46 | 111 | 3.60 | 2.78; 4.52 |
| | Egypt | | | | | | | | | | | 2 | 60 | 192 | 30.94 | 16.05; 48.21 | | | | | | 1 | 90 | 202 | 44.55 | 37.77; 51.44 |
| | Ethiopia | 1 | 36 | 104 | 34.62 | 25.81; 43.99 | | | | | | | | | | | | | | | | | | | | |
| | Ghana | | | | | | 1 | 182 | 402 | 45.27 | 40.44; 50.15 | | | | | | | | | | | | | | | |
| | Benin | | | | | | | | | | | | | | | | | | | | | | | | | |
| | Malawi | | | | | | | | | | | | | | | | | | | | | | | | | |
| | South Africa | | | | | | | | | | | | | | | | | | | | | | | | | |
| | South Sudan | | | | | | | | | | | | | | | | | | | | | | | | | |
| | Tanzania | | | | | | 1 | 16 | 200 | 8.00 | 4.65; 12.15 | | | | | | | | | | | | | | | |
| | Tunisia | | | | | | | | | | | 2 | 50 | 572 | 8.69 | 6.15; 11.62 | | | | | | | | | | |
| | Cameroon | | | | | | 1 | 41 | 289 | 14.19 | 10.41; 18.44 | | | | | | | | | | | | | | | |
| | Uganda | | | | | | 1 | 229 | 494 | 46.36 | 41.98; 50.76 | | | | | | | | | | | | | | | |
| Europe (29) | Finland | 1 | 31 | 220 | 14.09 | 9.82; 18.99 | | | | | | | | | | | | | | | | 1 | 26 | 105 | 24.76 | 17.02; 33.43 |
| | France | 1 | 134 | 306 | 43.79 | 38.28; 49.38 | 4 | 38 | 798 | 4.28 | 1.56; 8.26 | 1 | 16 | 147 | 10.88 | 6.38; 16.41 | 6 | 339 | 1688 | 19.31 | 10.71; 29.71 | | | | | |
| | Kosovo | | | | | | | | | | | | | | | | | | | | | | | | | |
| | Germany | 2 | 84 | 408 | 22.37 | 13.17; 22.37 | 1 | 64 | 246 | 26.02 | 20.74; 31.67 | 1 | 5 | 150 | 3.33 | 1.07; 6.79 | 3 | 45 | 624 | 6.04 | 2.13; 11.76 | 2 | 80 | 612 | 12.30 | 1.61; 30.88 |
| | Moldova | 1 | 135 | 264 | 51.14 | 45.11; 57.14 | | | | | | | | | | | | | | | | | | | | |
| | Greece | | | | | | 1 | 18 | 243 | 7.41 | 4.46; 11.03 | 3 | 52 | 982 | 5.22 | 3.84; 6.79 | | | | | | 1 | 15 | 198 | 7.58 | 4.31; 11.66 |
| | Turkey | | | | | | | | | | | 3 | 26 | 176 | 14.74 | 9.90; 20.34 | | | | | | 1 | 120 | 364 | 32.97 | 28.24; 37.88 |
| | UK | 1 | 2 | 75 | 2.67 | 0.26; 7.49 | 1 | 13 | 138 | 9.42 | 5.13; 14.83 | 1 | 28 | 76 | 36.84 | 26.42; 47.93 | | | | | | 1 | 16 | 377 | 4.24 | 2.45; 6.51 |
| | Iceland | 1 | 1 | 21 | 4.76 | 0.00; 17.67 | | | | | | | | | | | | | | | | | | | | |
| | Ireland | | | | | | | | | | | | | | | | | | | | | | | | | |
| | Italy | 7 | 67 | 557 | 13.04 | 4.12; 25.95 | 1 | 34 | 509 | 6.68 | 4.68; 9.01 | 4 | 65 | 770 | 9.20 | 3.56; 17.11 | 1 | 4 | 120 | 3.33 | 0.88; 7.27 | 1 | 16 | 375 | 4.27 | 2.46; 6.54 |
| | Czech | | | | | | | | | | | | | | | | | | | | | | | | | |
| | Denmark | | | | | | | | | | | | | | | | | | | | | | | | | |
| | Austria | | | | | | | | | | | | | | | | | | | | | | | | | |
| | Bulgaria | | | | | | | | | | | | | | | | | | | | | | | | | |
| | Montenegro | | | | | | | | | | | | | | | | | | | | | | | | | |
| | Netherlands | 3 | 168 | 856 | 13.21 | 0.29; 40.48 | 2 | 50 | 354 | 15.34 | 7.97; 24.58 | | | | | | 1 | 9 | 285 | 3.16 | 1.45; 5.50 | | | | | |
| | Norway | 1 | 10 | 46 | 21.74 | 11.16; 34.67 | | | | | | | | | | | | | | | | | | | | |
| | Poland | | | | | | 1 | 124 | 244 | 50.82 | 44.56; 57.07 | | | | | | | | | | | | | | | |
| | Portugal | 1 | 35 | 114 | 30.70 | 22.61; 39.45 | | | | | | | | | | | | | | | | | | | | |
| | Romania | | | | | | | | | | | | | | | | | | | | | | | | | |
| | Russia | | | | | | | | | | | | | | | | | | | | | | | | | |
| | San Marino | | | | | | | | | | | | | | | | | | | | | | | | | |
| | Serbia | | | | | | | | | | | | | | | | | | | | | | | | | |
| | Spain | | | | | | 7 | 569 | 3559 | 14.36 | 9.48; 20.05 | 2 | 7 | 113 | 6.19 | 2.52; 11.36 | 2 | 46 | 754 | 4.73 | 1.38; 9.93 | | | | | |
| | Sweden | 2 | 34 | 216 | 15.68 | 10.48; 21.70 | | | | | | 1 | 11 | 182 | 6.04 | 3.06; 9.96 | | | | | | | | | | |
| | Switzerland | | | | | | 1 | 19 | 735 | 2.59 | 1.56; 3.86 | | | | | | | | | | | | | | | |
| | Estonia | 1 | 9 | 67 | 13.43 | 6.39; 22.56 | | | | | | | | | | | | | | | | | | | | |
| | Croatia | | | | | | 1 | 51 | 504 | 10.12 | 7.64; 12.90 | | | | | | | | | | | | | | | |
| Oceania (2) | New Zealand | | | | | | | | | | | | | | | | | | | | | | | | | |
| | Australia | | | | | | 1 | 12 | 191 | 6.28 | 3.29; 10.15 | | | | | | | | | | | | | | | |
| North America (5) | Cuba | | | | | | | | | | | | | | | | | | | | | | | | | |
| | Mexico | 2 | 100 | 324 | 24.58 | 4.90; 52.88 | | | | | | | | | | | | | | | | | | | | |
| | USA | 2 | 96 | 460 | 18.14 | 5.66; 35.60 | 2 | 39 | 360 | 10.04 | 0.41; 30.09 | | | | | | 4 | 143 | 1033 | 15.03 | 3.51; 32.61 | | | | | |
| | Canada | | | | | | | | | | | | | | | | 1 | 22 | 80 | 27.50 | 18.33; 37.74 | | | | | |
| | Nicaragua | | | | | | | | | | | | | | | | | | | | | | | | | |
| South America (7) | Chile | | | | | | | | | | | | | | | | | | | | | | | | | |
| | Argentina | | | | | | 1 | 32 | 484 | 6.61 | 4.57; 8.99 | | | | | | 1 | 7 | 120 | 5.83 | 2.37; 10.71 | | | | | |
| | Venezuela | | | | | | | | | | | | | | | | | | | | | | | | | |
| | Bolivia | | | | | | | | | | | | | | | | | | | | | | | | | |
| | Brazil | 1 | 26 | 310 | 8.39 | 5.57; 11.72 | 3 | 169 | 1020 | 15.36 | 1.85; 38.45 | 2 | 4 | 457 | 1.41 | 0.00; 12.69 | 1 | 8 | 316 | 2.53 | 1.09; 4.55 | 2 | 83 | 997 | 7.62 | 3.51; 13.15 |
| | Guiana | | | | | | | | | | | | | | | | | | | | | | | | | |
| | Peru | | | | | | | | | | | | | | | | | | | | | 1 | 24 | 747 | 3.21 | 2.07; 4.60 |
| Multiple | Multiple country | 1 | 58 | 603 | 9.62 | 7.40; 12.10 | | | | | | | | | | | | | | | | | | | | |
| Total | | 40 | 2480 | 8776 | 24.04 | 18.55; 29.99 | 44 | 3112 | 15050 | 16.91 | 12.64; 21.67 | 35 | 852 | 6356 | 13.10 | 9.34; 17.39 | 26 | 789 | 7543 | 11.68 | 7.91; 16.06 | 20 | 1499 | 8072 | 21.49 | 12.65; 31.92 |
